# Electrochemical synthesis of 2-oxa-bicyclo[2.1.1]hexanes by anodic oxidation-cyclization relay strategy

**DOI:** 10.1039/d6sc03129c

**Published:** 2026-05-14

**Authors:** Andrea Brunetti, Giulia Monda, Alessandro Mazza, Magda Monari, Carlos Silva López, Giulio Bertuzzi, Marco Bandini

**Affiliations:** a Dipartimento di Chimica “Giacomo Ciamician”, Alma Mater Studiorum – Università di Bologna Via P. Gobetti 85 40129 Bologna Italy giulio.bertuzzi2@unibo.it marco.bandini@unibo.it; b Center for Chemical Catalysis – C^3^, Alma Mater Studiorum – Università di Bologna Via P. Gobetti 85 40129 Bologna Italy; c Departamento de Química Orgánica, Universidade de Vigo AS Lagoas (Marcosende) s/n 36310 Vigo Spain csilval@uvigo.gal

## Abstract

A general electrosynthetic strategy for the preparation and direct functionalization of 2-oxa-bicyclo[2.1.1]hexanes (*i.e.* 2-oxa-BCHs) from hydroxymethyl-substituted bicyclo[1.1.0]butanes is reported. The method relies on the anodic generation of electrophilic heteroatom-centred species, including TEMPO-derived oxy-cations, halogen radicals, and thiyl radicals, rendering C(4) functionalized 2-oxa-BCHs selectively in good to excellent yields. The protocol operates under mild conditions, avoids stoichiometric oxidants, and displays broad substrate scope (41 examples). The synthetic utility of the resulting scaffolds is further demonstrated through late-stage functionalization, bio-conjugation, and telescoped synthesis from commercially available precursors. Mechanistic studies (cyclovoltammetry as well as DFT computations) support a pathway involving anodic oxidation for the formation of the electrophilic trigger followed by C–C bond capture and transannular intramolecular cyclization. Overall, this work establishes electrosynthesis as a powerful platform for the modular construction and diversification of 2-oxa-BCHs with potential relevance in drug discovery and expanding the chemical space of benzene bioisosteres.

## Abstract 2

The electrochemical synthesis/functionalization of 2-oxa bicyclo[2.1.1]hexanes is realized from readily available bicyclo[1.1.0]butanes by means of green electrons as stoichiometric oxidants.

## Graphical abstract

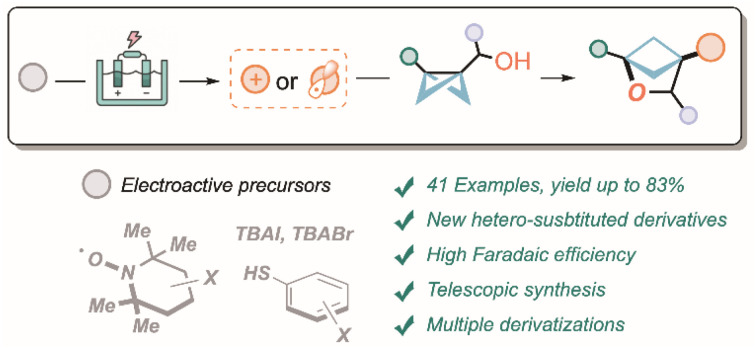

Benzene rings are highly prevalent in biologically active and pharmaceutical molecules, largely due to the wide array of synthetic strategies developed for their modification.^1,2^ Although the aromatic ring can directly influence biological activity through, for example, π–π interactions,^3–6^ it often acts mainly as a structural platform for functional groups placement. In such cases, substituting benzene with alternative motifs, known as isosteres, can address limitations such as poor solubility/physiologic availability, low metabolic stability, and reduced selectivity. These targeted substitutions may also help to overcome patent barriers and provide more efficient synthetic pathways to key APIs (*i.e.* Active Pharmaceutical Ingredients).^7–18^ The “escape from flatland” concept proposed by Lovering in 2009, embodies this design philosophy, supporting the replacement of planar aromatic systems with three-dimensional, saturated fragments to enhance solubility, molecular diversity, and ultimately, clinical success.^19–21^ Within these frameworks, 2-oxa-bicyclo[2.1.1]hexanes (*i.e.* 2-oxa-BCHs) have recently been addressed as one of the most promising scaffolds to explore such strategy.

Following the (re)discovery of this motif by the group of Mykhailiuk in 2020,^22^ a crescent interest can be traced in literature, as demonstrated by the diversified methodologies recently developed targeting its preparation.^23^ Even more importantly, the validation of 2-oxa-BCHs as mimic of *ortho-* or *meta*-disubstituted benzenes in drugs and agrochemicals^24^ has risen a wide attention on the industrial perspective, resulting in a large number of patent families (>60) over the last five years (Fig. 1).

**Fig. 1:**
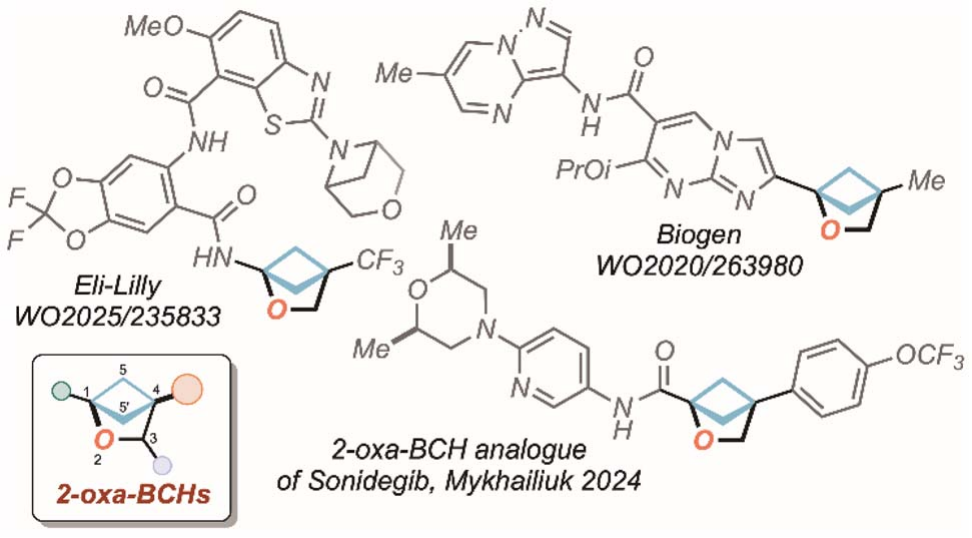
2-Oxa-BCHs in pharmaceutical industry: patented candidate drugs and bio-isosteres of existing APIs.

A literature search identifies three main existing concepts aimed at the formation of 2-oxa-BCHs,^25–27^ differing in their subsequent decoration possibilities and the pool of starting materials involved (Scheme 1A): the intermolecular iodo-cyclization of alkylidene-cyclobutanes;^22,24^ the intramolecular [2π + 2π] cycloaddition of allyl–vinyl ethers;^28–33^ and the intramolecular [2σ + 2π] cycloaddition between bicyclo[1.1.0]butanes (BCBs) and aldehydes.^34–37^ Despite the undoubted synthetic elegance and effectiveness characterizing these approaches, they are still constrained by important limitations, such as pre-functionalization of the starting materials, difficult access to C(3)-unsubstituted derivatives and the sometimes mandatory presence of EWG-groups at position C(4). Therefore, the challenge towards the identification of more reliable and sustainable synthetic approaches to expand the chemical space of 2-oxa-BCHs is still ongoing.^38,39^

**Scheme 1:**
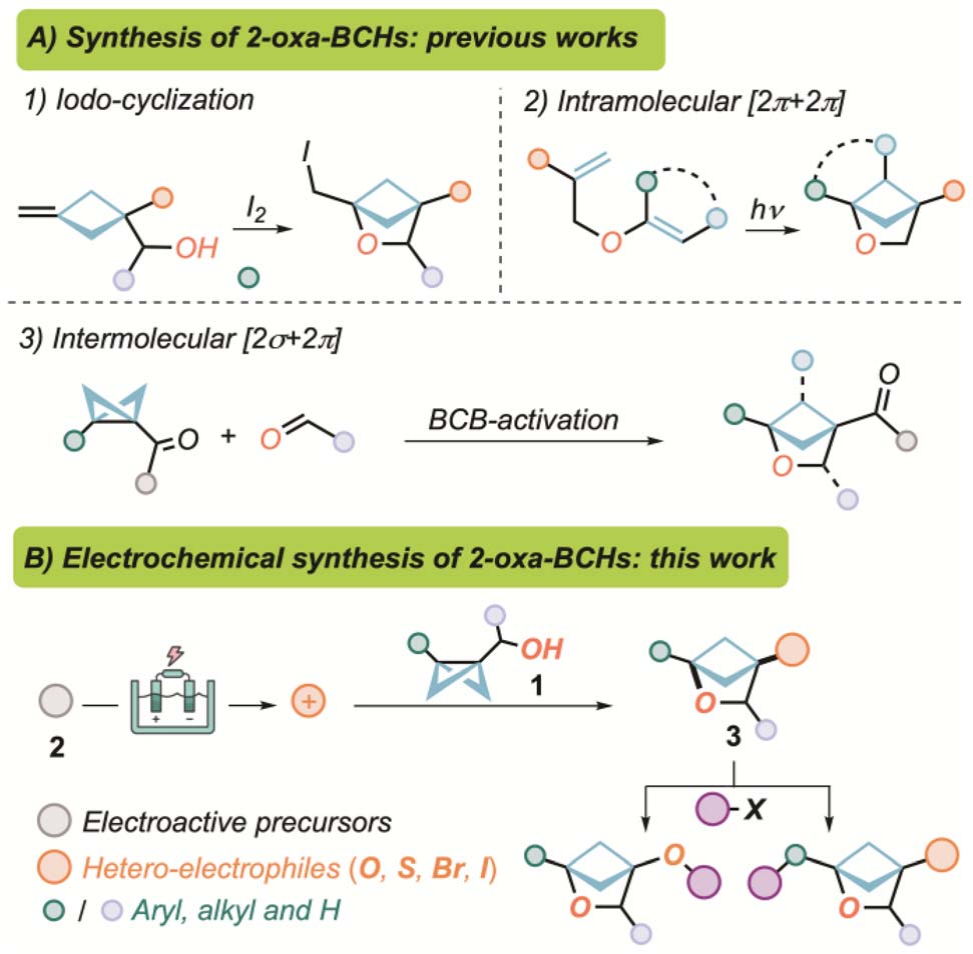
(A) Previously reported strategies for the preparation of 2-oxa-BCHs. (B) This work: electrochemical synthesis/functionalization of 2-oxa-BCHs.

Currently, the pharmaceutical industry is devoting growing efforts towards the implementation of greener energy inputs into complex reaction manifolds.^40–42^ Following this approach, electrochemistry in gaining particular attention due to the possibility to achieve redox processes avoiding the use of stoichiometric oxidants/reductants and, at the same time, providing mild and highly tuneable reaction machineries otherwise cumbersome to unlock.^43–48^

Surprisingly, the preparation of benzene bio-isosteric motifs by electrochemical techniques has not yet been recognized as a consolidated strategy,^49^ generating a gap in the combinatorial, academic as well as industrial interest in both fields. Within the context of our recent research interests focused on electrochemical mediated organic reactions,^50–55^ we therefore aimed at disclosing an electrosynthetic route towards 2-oxa-BCHs, paving the ways to the use of electricity in the preparation of benzene bio-isosteres through a new concept for the simultaneous generation and functionalization of such motifs (Scheme 1B).

In order to develop the proposed machinery, we targeted a new class of BCB-derivatives^56–60^ bearing a pendant hydroxymethyl group (1), easily obtainable from the corresponding esters or ketones. Compounds 1 are expected to feature a transannular C–C bond with a greater nucleophilic profile than the parent EWG-substituted analogues. Therefore, their reaction with an external electrophile 2, electrochemically generated by a chemoselective anodic event,^61–66^ would then enable functionalization at C(4), and subsequent ring-closure to the 2-oxa-bicyclo[2.1.1]hexane 3, with the pre-installed hydroxyl group.^66^ Concerning the electrochemically generated derivatizing species, we targeted candidates pivoting about heteroatoms such as oxygen, sulphur and halogens, that would not only provide a strategic handle for further functionalization of position C(4), but would also allow the exploration of a new family of 2-oxa-BCHs, as potential bio-isosteres of *meta*-substituted benzene derivatives.

We initially postulated that TEMPO (2,2,6,6-tetramethylpiperidine 1-oxyl) radical 2a, electrochemically oxidized to highly electrophilic oxy-cations,^67,68^ would be an expedite platform to realize the proposed strategy to give access to 4-hydroxy-2-oxa-bicyclo[2.1.1]hexane 3aa from model alcohol 1a. Our investigation began by subjecting 1a and TEMPO 2a (2 equiv) to constant current electrolysis (CCE, I = 2 mA) in the presence of LiClO_4_ as an electrolyte, a Ni cathode and a graphite (C) anode (CH_3_CN, rt, undivided cell).

**Table 1:** Optimization of the reaction conditions^a^

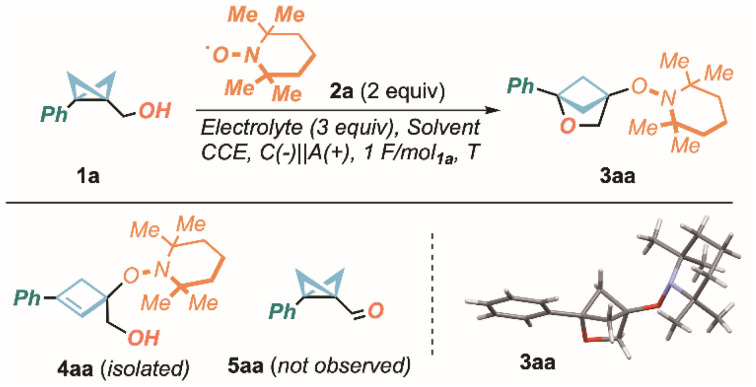					
Entry	Electrolytic conditions	Solvent	*T* (°C)	3aa : 4aa ratio^b^	Yield 3aa^c^ (%)
1	Ni(−)‖C(+), LiClO_4_ 2 mA	ACN	25	2.0 : 1	50
2	Ni(−)‖C(+), LiClO_4_ 6 mA	ACN	25	2.2 : 1	55
3	Ni(−)‖C(+), LiClO_4_ 10 mA	ACN	25	2.4 : 1	57
4	Ni(−)‖C(+), LiBF_4_ 10 mA	ACN	25	2.3 : 1	52
5	Ni(−)‖C(+), TBAClO_4_ 10 mA	ACN	25	2.0 : 1	48
6	Ni(−)‖C(+), TEABF_4_ 10 mA	ACN	25	1.6 : 1	39
7	Ni(−)‖SS(+), LiClO_4_ 10 mA	ACN	25	—	Traces
8	Ni(−)‖GC(+), LiClO_4_ 10 mA	ACN	25	2.3 : 1	47
9	Ni(−)‖C(+), LiClO_4_ 10 mA	DMF	25	8.8 : 1	44
10	Ni(−)‖C(+), LiClO_4_ 10 mA	DMSO	25	13 : 1	50
11	Ni(−)‖C(+), LiClO_4_ 10 mA	ACN	0	4.1 : 1	65
12	Ni(−)‖C(+), LiClO_4_ 10 mA	ACN	−30	5 : 1	70
13^d^	Ni(−)‖C(+), LiClO_4_ 10 mA	THF/ACN	−30	18 : 1	75

a All reactions were carried under air in the Electrasyn 2.0 apparatus, [1a] = 0.033 M.

b Determined on the ^1^H NMR spectrum of the crude mixture.

c Isolated yield after FC purification.

d THF/ACN = 2 : 1.

Importantly, a stoichiometric amount of electrons (1 F mol^−1^) was sufficient to achieve complete conversion of 1a.^69^ The feasibility of the proposed strategy was demonstrated by the successful isolation of the desired product 3aa in 50% yield, whose structure was also confirmed by single-crystal X-ray analysis.^70^ Nevertheless, cyclobutene 4aa (*vide infra*) was also obtained in 25% yield (entry 1, Table 1). Therefore, our efforts were directed towards the maximization of the reaction efficiency, as well as the suppression of 4aa. Increasing the current value from 2 mA (entry 2) to 6 mA (entry 3) and 10 mA (entry 4), progressively, showed a slight improvement in both isolated yield and 3aa : 4aa ratio (up to 57% and 2.4 : 1, respectively), besides providing a more convenient, faster reaction. On the other hand, the use of electrolytes different from LiClO_4_ such as LiBF_4_ (entry 4), TBAClO_4_ (entry 5) and TEABF_4_ (entry 6), or anodes different from C such as stainless steel (SS, entry 7)^71^ and glassy carbon (GC, entry 8) resulted in diminished yields and lower selectivity. Changing solvent from ACN to DMF (entry 9) or DMSO (entry 10) increased the 3aa : 4aa ratio up to 13 : 1, suggesting the polarity of the medium as a pivotal factor to direct the selectivity of the final stage of the process. However, these resulted in lower isolated yields, with ACN being kept as optimal, at this stage. Gratifyingly, by lowering the temperature to 0 °C (entry 11) and −30 °C (entry 12) both the yield and the selectivity improved (up to 70% and 5 : 1). Finally, switching the solvent to a binary mixture of THF and ACN (2 : 1), largely suppressed the formation of cyclobutene 4aa and yielded the desired product in optimal 75% yield (entry 13). Importantly, in all cases, by-product 5aa, deriving from the foreseeable TEMPO^+^ mediated oxidation of alcohols to aldehydes, of which electrochemical implementations exist,^72^ was never observed in the reaction crudes, highlighting higher reactivity of the Cα–Cγ σ-bond of BCB 1a with respect to the hydroxy moiety.

The robustness of the process was first assessed by conducting the model reaction on 1 mmol-scale, yielding product 3aa in 70% yield (Scheme 2).

Then, a range of primary and secondary BCB-alcohols 1b–w was subjected to the optimized conditions. Here, hydroxymethyl bicyclobutanes carrying γ-aryl substituents (1b–n) proved competent in the electrochemical synthesis regardless position as well as type of the chemical decoration. As a matter of fact, the presence of extended arenes (3ba–ca), alkyl (3da, 3fa, 3ga, 3ja), aryl (3ia) and alkoxy groups (3ha, 3ka), as well as of halogens (3ea, 3la–na) at the *ortho*, *meta* or *para* positions was very well tolerated, rendering the desired products 3 in high yields (up to 83%). In all cases, the reactions proved highly selective with no formation of cyclobutenes 4 or aldehydes 5, indicating the robustness of the disclosed methodology. A similar trend was recorded also in the presence of the alkyl-substituted precursor 1o that delivered the corresponding 2-oxa-BCH 3oa in 54% isolated yield.

**Scheme 2:**
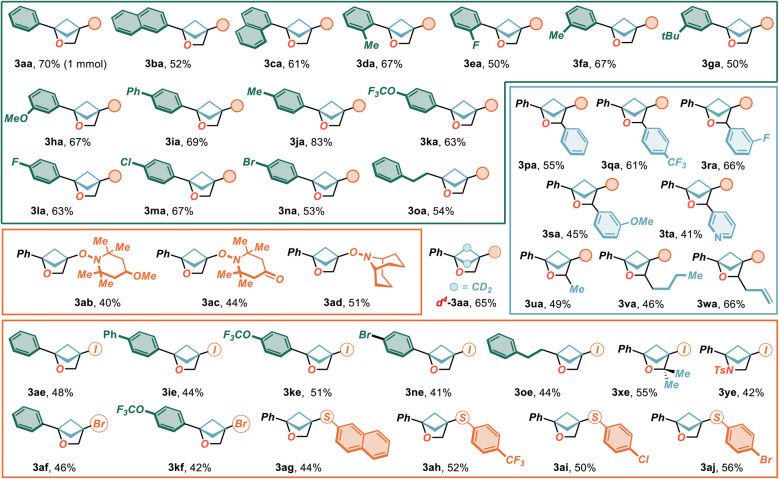
Assessing the generality of the electrochemical synthesis of 2-oxa-BCHs 3. ^*a*^All reactions were carried under air in the Electrasyn 2.0 apparatus, under the conditions reported in Table 1, entry 13, unless otherwise noted. All reported yields refer to isolated products after FC purification. Iodination reactions were carried out in the presence of TBAI (2e), 2.0 F mol_1_^−1^; bromination reactions with TBABr (2f), 3.5 F mol_1_^−1^ and thiolation reactions in the presence of thiophenols 2g–j and 2,6-lutidine (2 equiv), 2 F mol_1a_^−1^.

Then, the possibility of introducing substituents at position C(3) of the oxa-bicyclohexane core was undertaken by employing secondary alcohols 1p–w, deriving from a chemoselective reduction of the corresponding ketones (see SI for details). In this context, aryl (1p–s- yield up to 66%), heteroaryl (*i.e.* 3-pyridyl, 1t, 41% yield), alkyl (1u,v – yield up to 49%) and allyl (1w, 66% yield) groups could be efficiently introduced, providing a wide and faceted range of decorations and highlighting the versatility of this strategy in providing, following the same reaction concept, both C(3)-substituted and unsubstituted 2-oxa-BCHs 3, otherwise difficult to achieve by a unified methodology. The adaptability of the method to substituted TEMPO derivatives 2b,c as well as ABNO (9-aza-bicyclo[3.3.1]nonane *N*-oxyl) radical 2d was also proved. Here, the desired 2-oxa-BCHs 3ab–d were isolated in moderated to good yields (up to 51%).

Then, we targeted the preparation of ***d***^***4***^-1a that, when subjected to the optimized conditions, led to ***d***^***4***^-3aa in high yield (65%). This represents the first entry to a 2-oxa-BCH motif featuring complete deuteration of the 4-membered ring that would meet the current “deuterium switch” strategy in medicinal chemistry.^73,74^

At this stage, we postulated that our strategy was not limited to oxy-cations for the trigger of the intramolecular formation of 2-oxa-BCHs (one-electron process) but could be extended to electrophilic species generated by the electrochemical oxidation of heteroatom-centred anions (two-electron process). To our delight, by subjecting a set of primary (1a,i,k,n,o) and tertiary 1x alcohols to the optimized conditions in the presence of tetrabutylammonium iodide (2e) the corresponding C(4)-iodo compounds 3 were isolated in moderate to good yields (up to 55%). The impact of the tethering group was also investigated by employing *N*-protected aminomethyl bicyclobutane 1y to electro-oxidative cyclization.^.^ Here, synthetic valuable 2-azabicyclo[2.1.1]hexane 3ye was obtained in moderate yield (42%).

Analogously, the procedure turned out to be efficient also for the construction of brominated 2-oxa-BCHs, by using tetrabutylammonium bromide 2f as an electroactive precursor (yield up to 46%). Finally, aromatic thiols (2g–j) were also shown to produce electrophilic species amenable to trigger the desired transformation upon treatment with a base (*i.e.* 2,6-lutidine). The corresponding sulphides 3ag–j were isolated with synthetically useful outcomes (44–56% yield). In all cases, the formation of cyclobutenes 4 was not observed, confirming a completely selective protocol, irrespective of the electrophile of choice. Importantly, the incorporation of halogens and sulphur at this position of 2-oxa-BCHs would contribute to opening new opportunities for accessing derivatives of significant synthetic and applicative relevance.

The use of TEMPO cation as an electrophilic trigger towards the formation of 2-oxa-BCHs 3 finds a high functional relevance due to the oxygenation of C(4), and in the flexibility of the resulting hydroxyl group as a synthetic handle on subsequent derivatization steps to access a new space of structural complexity (Scheme 3). Therefore, after a small set of optimizations, directed towards the minimization of by-product 7 (Grob fragmentation), we successfully achieved alcohol 6 in 57% yield (6 : 7 = 4 : 1) by Zn/AcOH-mediated reductive cleavage of the N–O bond (20% recovered 3aa).

**Scheme 3:**
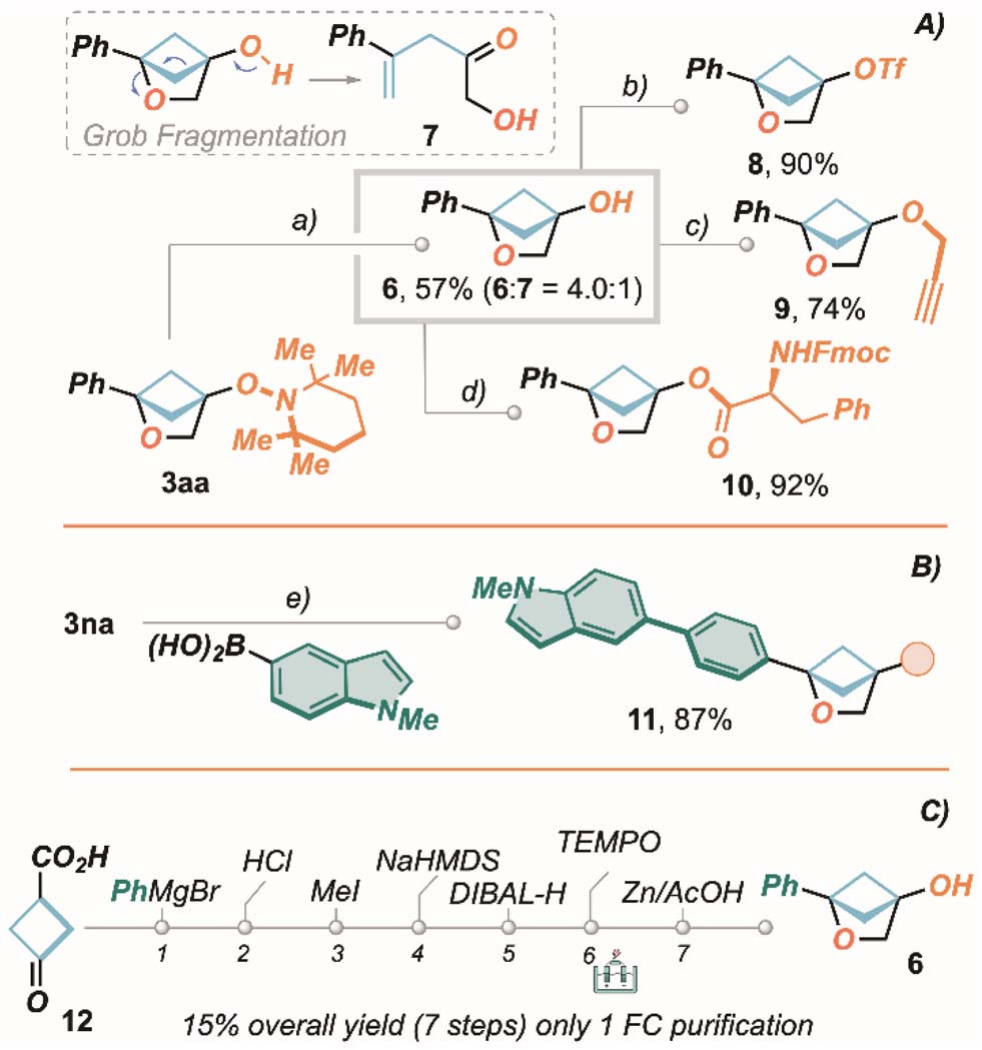
(A) Transformation of products 3aa. (B) Transformation of product 3an. (C) Telescoped synthesis of 6 from commercially available starting materials. Reaction conditions: (a) Zn dust (2.5 equiv), AcOH, rt, 4 h. (b) Tf_2_O (1.1 equiv), pyridine (1.1 equiv), DCM, 1 h. (c) NaH (1.1 equiv), propargyl bromide (1.1 equiv), DMF, 0 °C to rt, 1.5 h. (d) Fmoc-PheOH (1.5 equiv), EDC HCl (1.5 equiv), DMAP (10 mol%), DCM, 0 °C to rt, 3 h. (e) *N*-methyl-5-indolylboronic acid (2.0 equiv), [Pd(PPh_3_)_2_Cl_2_] (5 mol%), Cs_2_CO_3_ (3.0 equiv), THF/H_2_O (10 : 1), reflux, 3 h. For the telescoped synthesis in detail see the SI.

We then moved to demonstrate the utility of this compound by the productive formation of triflate 8 (90% yield), propargyl ether 9 (74% yield) and phenylalanine ester 10, suggesting an excellent potential for bio-conjugations of the 4-hydroxy-2-oxa-BCH 6 (Scheme 3A). Furthermore, to elect also the aromatic ring at position C(1) as a productive site for further modification, Suzuki coupling with *N*-methyl-5-indolyl boronic acid was successfully achieved on derivative 3na towards bi-aryl 11 in 87% yield (Scheme 3B).

Finally, the robustness of the present methodology was underlined by targeting alcohol 6 directly from commercially available ketone 12, *via* a telescopic approach (Scheme 3C). Remarkably, the 7-step procedure resulted in the formation of the desired compound in 76% average yield per-step (15% overall yield), requiring only one final chromatographic purification.

With the aim of better understanding the mechanism of the disclosed protocols, we subjected BCB 1a, TEMPO 2a, TBABr 2f, TBAI 2e and potassium *p*-chlorothiophenolate (*p*-ClPhSK, 2i potassium salt) to voltammetric analysis (Scheme 4A). This showed that oxidation of 1a occurs at higher potential (*E*_p_ = 0.81 V *vs.* Fc/Fc^+^ redox couple) with respect to all other species 2 (*p*-ClPhS^−^2i potassium salt: *E*_p_ = −0.43 V; TEMPO 2a: *E*_1/2_ = 0.20 V, I^−^2e: *E*_p_ = 0.58 V; Br^−^2f: *E*_p_ = 0.79 V *vs.* Fc/Fc^+^ redox couple), indicating that 2 are the effective electroactive species. Therefore, we suggest that the productive process occurs only when the pro-electrophilic species is generated at lower voltages compared to the oxidation of 1a, decomposition of the latter occurring otherwise. In the case of the reversible single-electron oxidation of radical 2a, the reactivity of the anodically generated cation with BCB 1a was substantiated by a “titration” experiment.

**Scheme 4:**
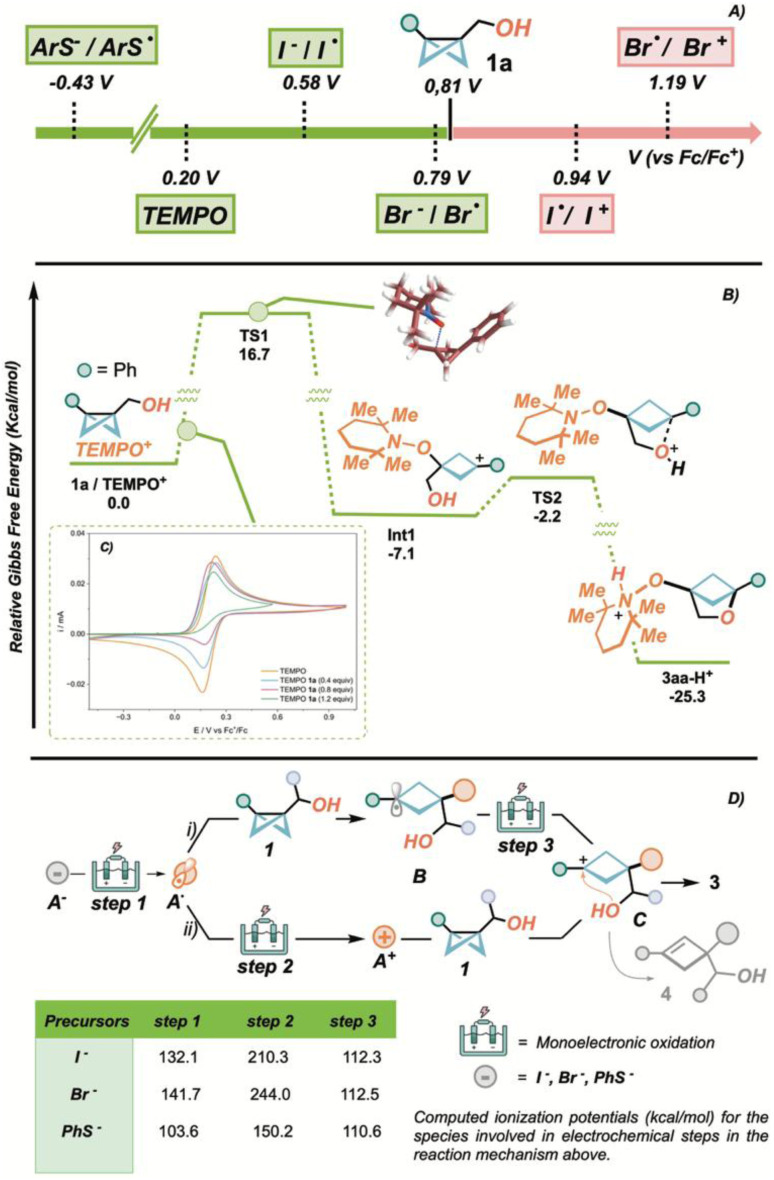
(A) Oxidation potentials of 1a, 2a, 2e, 2f and 2i potassium salt.^*a*^ (B) Mechanistic insights for the TEMPO 2a-mediated pathways.^*b*^ (C) Voltammetric titration experiment.^*c*^ (D) Mechanistic insights for the I^−^, Br^−^ and PhS^−^ precursors.^*d a*^Oxidation potentials collected by CV responses measured in a 3-electrode cell, ACN solvent, [2a, 2e, 2f, 2i potassium salt, 1a] = 2.10 mM, TEABF_4_ electrolyte (0.10 M).^*b*^ Energies in kcal mol^−1^.^*c*^ CV responses measured in a 3-electrode cell, ACN solvent, [2a] = 2.10 mM (all lines), TEABF_4_ electrolyte (0.10 M), [1a] = 0.84 mM (light blue line), [1a] = 1.68 mM (pink line), [1a] = 3.76 mM. See SI for full experimental details.^*d*^ Ionization potentials in the table are reported in kcal mol^−1^.

Here, increasing amounts of 1a were added to a solution of 2a, subjected to voltammetric analyses (Scheme 4C). The progressive disappearance of the return curve (TEMPO^+^/TEMPO˙) indicates a concentration-dependent depletion of TEMPO^+^ from the solution, due to irreversible reaction with 1a.

Based on this preliminary mechanistic evidence and to gain further insight on the TEMPO reaction machinery, we carried out DFT simulations at the wB97xD/Def2TZVP level (see the SI for methodological details). The reaction profile obtained is summarized in Scheme 4B and it involves an initial attack of TEMPO^+^ onto the BCB substrate 1 which is kinetically rate determining; the thus obtained carbocationic intermediate Int1,^24,75^ is rapidly trapped by the internal nucleophilic alcohol through a low lying transition state (with a barrier of only 4.9 kcal mol^−1^) yielding the final 2-oxa-BCH 3.^76^

This mechanistic picture is in good agreement with the measured oxidation potentials for TEMPO 2a and 1a.

The reactivity of the anionic precursors A^−^ (I^−^, Br^−^ and ArS^−^) poses an additional mechanistic interrogative: what species suffer the second electrochemical event after the initial oxidative step of the precursor resulting in the electrophilic radicals A˙ (Scheme 4C)? In this regard we tackled the mechanistic divergency regarding the direct attack of A˙ into the BCB-1a (Scheme 4C upper pathway) or its second SET oxidation resulting in the corresponding highly electrophilic halonium/thionium cations A^+^ (Scheme 4D lower pathway). Voltammetric analyses suggest the unlikeliness of this second hypothesis in the case of iodination and bromination reactions,^77^ where the formation of the halonium ion occurs at a higher potential when compared to the oxidation of BCB 1a (I˙/I^+^: *E*_p_ = 0.94 V; Br˙/Br^+^: *E*_p_ = 0.94 V *vs.* Fc/Fc^+^ redox couple). In these cases, prior to the formation of the cationic species D, a voltage able to anodically decompose 1a would be reached, preventing the desired process to occur. However, as the electrodic processes generating halogen- or sulfur-centered radicals are irreversible—thus preventing a titration experiment analogous to that performed for TEMPO 2a—we resorted to DFT calculations to evaluate the reactivity of these species toward 1 (detailed reaction profiles can be found in the SI). Our simulations on the electrochemical steps involved in these two mechanisms are well aligned with the experimental observations above for the first electrochemical event, bromide, iodide, and thiolate (A^−^) precursors can be oxidized to their corresponding radicals (A˙) with affordable potentials (Scheme 4D, table). The second electrochemical event (A˙/A^+^), however, is more energetically demanding (244.0, 210.3 and 150.2 kcal mol^−1^, respectively) on the precursors than on intermediate B, formed after the attack of the A˙ radicals on BCB 1 (about 110 kcal mol^−1^ to transform radical B into carbocation C, Scheme 4D).

Based on the aforementioned experimental and computational evidence we propose that electrophilic radicals A˙ react with 1, leading to the formation of stable benzyl radicals B that undergo a second anodic oxidation to benzylic cations C. In this context, as well as in the case of cation-based oxygenations, the formation of cyclobutenes 4 is postulated to arise from an undesired quenching of carbocations (C or Int1) by proton elimination, competing with the intramolecular nucleophilic trapping of the hydroxyl group. This serves as direct experimental evidence on the formation of carbocationic species as pivotal intermediates leading to the desired products.

## Conclusions

In conclusion, we have disclosed a general and selective electrochemical strategy for the synthesis of 2-oxa-bicyclo[2.1.1]hexanes (2-oxa-BCHs) as valuable benzene bioisosteres, addressing limitations of existing methodologies. By leveraging the unique reactivity of hydroxymethyl-substituted bicyclo[1.1.0]butanes and electrochemically generated electrophilic species, this approach enables the simultaneous formation and functionalization of the 2-oxa-BCH core under mild, oxidant-free conditions. The method displays broad substrate scope, accommodates both C(3)-substituted and unsubstituted derivatives, and allows the introduction of diverse heteroatom-based functionalities at C(4), including oxygen, halogens, and sulphur, which serve as versatile handles for downstream diversification. Mechanistic studies support a pathway involving anodic generation of electrophilic intermediates and intramolecular trapping to forge the bicyclic scaffold with high selectivity. Taken together, this work not only expands the accessible chemical space of 2-oxa-BCHs but also establishes electrosynthesis as a powerful platform for the preparation of benzene bioisosteres, opening new opportunities at the interface of synthetic methodology, medicinal chemistry, and industrial application.

## Author contributions

MB, GB and CSL directed the research. AB, GM and AM carried out both the synthesis of the starting materials and their determination *via* single-X-ray crystal diffraction. All authors give approval to the final version of the manuscript.

## Conflicts of interest

The authors declare no conflicts of interest.

## Supplementary Material

SC-017-D6SC03129C-s001

SC-017-D6SC03129C-s002

## Data Availability

CCDC 2517247 (3aa) contains the supplementary crystallographic data for this paper. The supporting data has been provided as part of the supplementary information (SI). Supplementary information: NMR spectra and further experimental details. See DOI: https://doi.org/10.1039/d6sc03129c.

## References

[cit1] Taylor R. D., MacCoss M., Lawson A. D. G. (2014). J. Med. Chem..

[cit2] Nilova A., Campeau L. C., Sherer E. C., Stewart D. R. (2020). J. Med. Chem..

[cit3] Meyer E. A., Castellano R. K., Diederich F. (2003). Angew. Chem., Int. Ed..

[cit4] Salonen L. M., Ellermann M., Diederich F. (2011). Angew. Chem., Int. Ed..

[cit5] Schenkel L. B., Olivieri P. R., Boezio A. A., Deak H. L., Emkey R., Graceffa R. F., Gunaydin H., Guzman-Perez A., Lee J. H., Teffera Y., Wang W., Youngblood B. D., Yu V. L., Zhang M., Gavva N. R., Lehto S. G., Geuns-Meyer S. (2016). J. Med. Chem..

[cit6] Gunaydin H., Bartberger M. D. (2016). ACS Med. Chem. Lett..

[cit7] Locke G. M., Bernhard S. S. R., Senge M. O. (2019). Chem.–Eur. J..

[cit8] Mykhailiuk P. K. (2019). Org. Biomol. Chem..

[cit9] Subbaiah M. A. M., Meanwell N. A. (2021). J. Med. Chem..

[cit10] Wiesenfeldt M. P., Rossi-Ashton J. A., Perry I. B., Diesel J., Garry O. L., Bartels F., Coote S. C., Ma X., Yeung C. S., Bennett D. J., MacMillan D. W. C. (2023). Nature.

[cit11] Diepers H. E., Walker J. C. L. (2024). Beilstein J. Org. Chem..

[cit12] Prysiazhniuk K., Datsenko O. P., Polishchuk O., Shulha S., Shablykin O., Nikandrova Y., Horbatok K., Bodenchuk I., Borysko P., Shepilov D., Pishel I., Kubyshkin V., Mykhailiuk P. K. (2024). Angew. Chem., Int. Ed..

[cit13] Tsien N., Hu C., Merchant R. R., Qin T. (2024). Nat. Rev. Chem..

[cit14] Garrido-García P., Quiròs I., Milàn-Rois P., Ortega-Gutièrrez S., Martin-Fontecha M., Campos L. A., Somoza A., Fernàndez I., Rigotti T., Tortosa M. (2025). Nat. Chem..

[cit15] Surendran S., Raju S., Kalyani N., Saini G., Nandha J., Bhowmik H., Rathod A., Choubey S. S., Anandamoorthy B., Sahoo S. K., Gupta Y. K., Vetrichelvan M., Gupta A., Desai S., Mariappan T., Mathur A., Vachaspati P. R., Subbaiah M. A. M. (2025). J. Med. Chem..

[cit16] Xia K. (2025). Chem. Commun..

[cit17] Hernandez-Llado P., Meanwell N. A., Russel A. (2025). J. Med. Chem..

[cit18] Nato P., Colella M., Luisi R. (2025). Chem. Commun..

[cit19] Lovering F., Bikker J., Humblet C. (2009). J. Med. Chem..

[cit20] Lovering F. (2013). Medchemcomm.

[cit21] Wei W., Cherukupalli S., Jing L., Liu X., Zhan P. (2020). Drug Discov. Today.

[cit22] Levertov V. V., Panasyuk Y., Pivnytska V. O., Mykhailiuk P. K. (2020). Angew. Chem., Int. Ed..

[cit23] Whalley D. M., Lorthioir O., Coote S. C., Anderson N. A. (2025). Org. Biomol. Chem..

[cit24] Levterov V. V., Panasiuk Y., Shablykin O., Stashkevych O., Sahun K., Rassokhin A., Sadkova I., Lesyk D., Anisiforova A., Holota Y., Borysko P., Bodenchuk I., Voloshchuk N. M., Mykhailiuk P. K. (2024). Angew. Chem., Int. Ed..

[cit25] Tamura Y., Ishibashi H., Hirai M., Kita Y., Ikeda M. (1975). J. Org. Chem..

[cit26] Kirmse W., Mrotzeck U. (1988). Chem. Ber..

[cit27] Matlin A. R., Turk B. E., McGarvey D. J., Manevich A. A. (1992). J. Org. Chem..

[cit28] Graßl R., Jandl C., Bach T. (2020). J. Org. Chem..

[cit29] Rigotti T., Schwinger D. P., Graßl R., Jandl C., Bach T. (2022). Chem. Sci..

[cit30] Denisenko A., Garbuz P., Voloshchuk N. M., Holota Y., Al-Maali G., Borysko P., Mykhailiuk P. K. (2023). Nat. Chem..

[cit31] Lorthioir O., Anderson N., Boyd S., Carlino L., Davey P., Hodds W., Howard M., Lindhagen M., Proctor K., Putra O. D., Smith T., Turner O., Woodhouse A., Woodward M. (2024). Org. Lett..

[cit32] Whalley D. M., Carlino L., Putra O. D., Anderson N. A., Coote S. C., Lorthioir O. (2024). Chem. Sci..

[cit33] Homon A. A., Hryshchuk O. V., Mykhailenko O. V., Vashchenko B. V., Melnykov K. P., Michurin O. M., Daniliuc C. G., Gerus I. I., Kovtunenko V. O., Kondratov I. S., Grygorenko O. O. (2021). Eur. J. Org Chem..

[cit34] Liang Y., Kleinmans R., Daniliuc C. G., Glorius F. (2022). J. Am. Chem. Soc..

[cit35] Liang Y., Paulus F., Daniliuc C. G., Glorius F. (2023). Angew. Chem., Int. Ed..

[cit36] Li T., Wang Y., Xu Y., Ren H., Lin Z., Li Z., Zheng J. (2024). ACS Catal..

[cit37] Tang S.-Y., Wang Z.-J., Wu J.-J., Xing Z.-X., Du Z.-Y., Huang H.-M. (2025). Chem. Sci..

[cit38] Fawcett A., Biberger T., Aggarwal V. K. (2019). Nat. Chem..

[cit39] Bennett S. H., Fawcett A., Denton E. H., Biberger T., Fasano V., Winter N., Aggarwal V. K. (2020). J. Am. Chem. Soc..

[cit40] Tanbouza N., Ollevier T., Lam K. (2020). iScience.

[cit41] Xia R., Overa S., Jiao F. (2022). JACS Au.

[cit42] Ferretti A. C., Cohen B., Deng L., Diwan M., Frederick M. O., Lehnherr D. (2025). Org. Process Res. Dev..

[cit43] Francke R., Little R. D. (2014). Chem. Soc. Rev..

[cit44] Wiebe A., Gieshoff T., Mçhle S., Rodrigo E., Zirbes M., Waldvogel S. R. (2018). Angew. Chem., Int. Ed..

[cit45] Horn E. J., Rosen B. R., Baran P. S. (2016). ACS Cent. Sci..

[cit46] Novaes L. F. T., Liu J., Shen Y., Lu L., Meinhardt J. M., Lin S. (2021). Chem. Soc. Rev..

[cit47] Zhu C., Ang N. W., Meyer T. H., Qiu Y., Ackermann L. (2021). ACS Cent. Sci..

[cit48] Wang Y., Dana S., Long H., Xu Y., Li Y., Kaplaneris N., Ackermann L. (2023). Chem. Rev..

[cit49] Maddigan-Wyatt J. T., Knyazev D. A., Werz D. B. (2026). Org. Lett..

[cit50] Brunetti A., Kiriakidi S., Garbini M., Monda G., Zanardi C., López C. S., Bertuzzi G., Bandini M. (2025). ACS Catal..

[cit51] Brunetti A., Garbini M., Kub N. G., Monari M., Pedrazzani R., Zanardi C., Bertuzzi G., Bandini M. (2024). Adv. Synth. Catal..

[cit52] Brunetti A., Garbini M., Autuori G., Zanardi C., Bertuzzi G., Bandini M. (2024). Chem.–Eur. J..

[cit53] Garbini M., Brunetti A., Pedrazzani R., Monari M., Marcaccio M., Bertuzzi G., Bandini M. (2024). Chem. Commun..

[cit54] Rapisarda L., Fermi A., Ceroni P., Giovanelli R., Bertuzzi G., Bandini M. (2023). Chem. Commun..

[cit55] Bertuzzi G., Ombrosi G., Bandini M. (2022). Org. Lett..

[cit56] Golfmann N., Walker J. C. L. (2023). Commun. Chem..

[cit57] Kelly C. B., Milligan J. A., Tilley L. J., Sodano T. M. (2022). Chem. Sci..

[cit58] Zhou X., Hu Y., Huang Y., Xiong Y. (2024). Chem. Commun..

[cit59] Hu Q.-Q., Chen J., Yang Y., Yang H., Zhou L. (2024). Tetrahedron Chem.

[cit60] Giovanelli R., Gallorini G., Huang Y., Brunetti A., Nikopoulos K. D., Monari M., Silva López C., Kiriakidi S., Bertuzzi G., Bandini M. (2025). ACS Catal..

[cit61] Jiang D.-B., Wu F.-Y., Cui H.-L. (2023). Org. Biomol. Chem..

[cit62] Yuan Y., Yao A., Zheng Y., Gao M., Zhou Z., Quiao J., Hu J., Ye B., Zhao J., Wen H., Lei A. (2019). iScience.

[cit63] Kim W., Young H., Kim Y., Oh K. (2020). Org. Lett..

[cit64] Kurig N., Palkovits R. (2023). Green Chem..

[cit65] Wu J., Dou Y., Guillot R., Kouklovsky C., Vincent G. (2019). J. Am. Chem. Soc..

[cit66] Li D., Li S., Peng C., Lu L., Wang S., Wang P., Chen Y.-H., Cong H., Lei A. (2019). Chem. Sci..

[cit67] Nutting J. E., Rafiee M., Stahl S. S. (2018). Chem. Rev..

[cit68] Siu J. C., Sauer G. S., Saha A., Macey R. L., Fu N., Chauviré T., Lancaster K. M., Lin S. (2018). J. Am. Chem. Soc..

[cit69] In most cases, complete consumption of **1a** was observed, even in low yielding reactions, probably due to decomposition-polymerization side reactions

[cit70] CCDC 2517247: Experimental Crystal Structure Determination, 2026, 10.5517/ccdc.csd.cc2qhdj5

[cit71] Oxidation of stainless steel to Fe(II) or Fe(III) cations probably led to Lewis-acid catalyzed decomposition of **1a**

[cit72] Schroeder C. M., Politano F., Ohlhorst K. K., Leadbeater N. E. (2023). RSC Adv..

[cit73] Pirali T., Serafini M., Cargnin S., Genazzani A. A. (2019). J. Med. Chem..

[cit74] Chen Y., Du Y. (2024). ChemMedChem.

[cit75] Wang Y., Zheng L., Liu X., Zhang C., Li Y., Li Q., Zhang X., Lin W., Liu C. (2025). Angew. Chem., Int. Ed..

[cit76] This evidence provides an explanation why the reaction intermediate **Int1** could not be captured *via* the addition of external nucleophiles (*i.e.* methanol), since the intramolecular pathway is kinetically very rapid

[cit77] The second oxidation peak showed in the voltammetric analysis of p-ClPhSK was not clearly attributable to the formation of the cationic species

