# Supplementary material for: Electrochemical synthesis of 2-oxa-bicyclo[2.1.1]hexanes by anodic oxidation-cyclization relay strategy
Source: Chem Sci. 2026 May 14;17(26):13055–61. doi: 10.1039/d6sc03129c (PMC13209150; doi:10.1039/d6sc03129c)
Supplement: SC-017-D6SC03129C-s001 [file SC-017-D6SC03129C-s001.pdf]

Supplementary Information (SI) for Chemical Science.
This journal is © The Royal Society of Chemistry 2026

Electrochemical Synthesis of 2-Oxa-Bicyclo[2.1.1]hexanes by Anodic Oxidation-Cyclization Relay Strategy

Supplementary Information

Andrea Brunetti,[a,b] Giulia Monda,[a,b] Alessandro Mazza,[a] Magda Monari,[a] Carlos Silva López,*[c] Giulio Bertuzzi,*[a,b] Marco Bandini*[a,b]

[a] Mr. A. Brunetti, Ms. G. Monda, Dr. G. Bertuzzi, Prof. M. Monari, Prof. M. Bandini, Dipartimento di Chimica "Giacomo Ciamician", Alma Mater Studiorum – Università di Bologna, Via P. Gobetti 85, 40129 Bologna, Italy.

[b] Mr. A. Brunetti, Ms. G. Monda, Dr. G. Bertuzzi, Prof. M. Bandini, Center for Chemical Catalysis – C[3] Alma Mater Studiorum – Università di Bologna, via P. Gobetti 85, 40129 Bologna, Italy.

[c] Departamento de Química Orgánica, Universidade de Vigo, AS Lagoas (Marcosende) s/n, 36310 Vigo, Spain.

E-mail: giulio.bertuzzi2@unibo.it, marco.bandini@unibo.it.

Table of Contents

1. General Information S3

2. Preparation of Starting Materials S5

3. Preparation and Characterization of Products **3** S15

4. Product Elaborations S34

5. Crystallographic Data S43

6. Voltametric Analyses S45

7. Additional Mechanistic Elucidations S51

8. NMR Spectra S55

9. Cartesian Coordinates S142

10. References S194

1. General Information

Instrumentation and Chemicals.

^{1}H NMR, ^{13}C NMR and ^{19}F NMR spectra were recorded on Bruker NeoAvance 600 (600 MHz) or INOVA 600 MR (600 MHz). Data are reported as follows: chemical shift, multiplicity (s = singlet, d = doublet, dd= double doublet, t = triplet, td = triple doublet, dt = double triplet, q = quartet, sext = sextet, sept = septet, p = pseudo, b = broad, m = multiplet), coupling constants (Hz). Chemical shifts are reported in ppm from TMS with the solvent resonance peak as the internal standard.

HRMS spectra were obtained with a G2XS QTof mass spectrometer using ESI ionization technique, as specified case by case.

Melting points were determined with a Büchi Melting Point B-540 apparatus and are not corrected.

Chromatographic purification was done with 240-400 mesh silica gel.

Anhydrous solvents were supplied by Sigma Aldrich in Sureseal® bottles and used without any further purification.

Commercially available chemicals and (non-anhydrous) solvents were purchased from Sigma Aldrich, Fluorochem and TCI Chemicals and used without any further purification.

Starting materials **S1** were prepared by our reported modification of literature procedures.[1] Starting materials **1t**, **1x** and **1y** were prepared following literature procedures (**1t** and **1y** are known compounds).[2]

Computational Methodology

We have used the Density Functional Theory (DFT) in the Kohn-Sham formulation to optimize all the stationary points presented in this manuscript. Geometries of all the stationary points were fully optimized at the ωB97xD[3]/Def2TZVP[4,5] computational level. This combines a density functional corrected to account for dispersion interactions which outperforms most other functionals in countless benchmark studies and a highly flexible and balanced triple-ζ basis set. The effect of solvent (acetonitrile) was modeled using the polarizable continuum model (PCM)[6] with the default parameters implemented in the Gaussian 16 package for this solvent.[7]

All geometry optimizations have been performed using tight convergence criteria in the SCF and requesting a pruned grid with 175 radial shells and 974 angular points for the first row atoms and a finer grid with 250 radial shells and 974 angular points for second row and later elements to guarantee the accuracy of the reported results. Thermochemical calculations were performed assuming a rigid rotor model for the rotation levels and harmonic potentials for the vibrational modes considering 1.0 atm and 298.1 K.

Frequency analysis was used to establish the nature of all optimized structures as either minima or transition structures. For all stationary points, the stability of the wave function was examined.[8,9,10]

2. Preparation of Starting Materials

2.1 General Procedure A for the preparation of primary alcohols **1a-n**

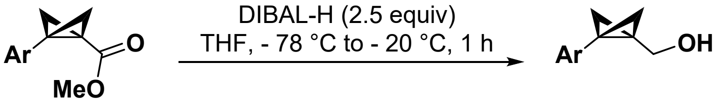

S1a-o **1a-o**

In a heat-gun dried 3-necked round bottom flask, equipped with a magnetic stirring bar and under Ar atmosphere, ester **S1a-o** (0.3 mmol) was dissolved in anhydrous THF (3 mL), cooled to – 78 °C and a solution of DIBAL-H (2.5 equiv, 0.83 mmol, 0.83 mL, 1.0 M in toluene) was added dropwise. After the addition was completed, the resulting solution was stirred for 1 h, during which time the temperature was allowed to rise to – 20 °C. Then, EtOAc (5 mL) was carefully added, followed by a saturated solution of Rochelle's salt (5 mL) The biphasic mixture was then vigorously stirred at room temperature for 1 h, until two clear phases were obtained and the transferred to a separatory funnel. The phases were separated, the aqueous phase was extracted again with EtOAc (2 x 5 mL), the combined organic phases were washed with a saturated solution of Rochelle's salt (2 x 5 mL), dried over Na_2SO_4 and concentrated *in vacuo* to obtain primary alcohols **1a-o**, that were directly used in the electrochemical protocol without further purification.

2.2 General Procedure B for the preparation of secondary alcohols **1o-u**

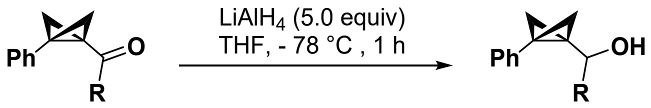

S1p-s, S1u-w **1p-s, 1u-w**

In a heat-gun dried 3-necked round bottom flask, equipped with a magnetic stirring bar and under Ar atmosphere, ketone **S1p-s**, **S1u-w** (0.3 mmol) was dissolved in anhydrous THF (5 mL), cooled to – 78 °C and a solution of $LiAlH_4$ (5.0 equiv, 1.5 mmol, 0.73 mL, 2.3 M in 2-MeTHF) was added dropwise. After the addition was completed, the resulting solution was stirred for 1 h at – 78 °C. Then, EtOAc (5 mL) was carefully added, followed by a saturated solution of Rochelle's salt (5 mL) The biphasic mixture was then vigorously stirred at room temperature for 1 h, until two clear phases were obtained, and the transferred to a separatory funnel. The phases were separated, the aqueous phase was extracted again with EtOAc (2

x 5 mL), the combined organic phases were washed with a saturated solution of Rochelle's salt (2 x 5 mL), dried over Na_2SO_4 and concentrated *in vacuo* to obtain secondary alcohols **1p-s** and **1u-w**, that were directly used in the electrochemical protocol without further purification.

*<u>Note</u>: In an attempted preparation of secondary alcohol **1p** from ketone **S1p**, the use of DIBAL-H led to a mixture of the desired product **1p** and byproduct **S2p**, deriving from the "conjugated" addition of the hydride to the C_α-C_γ bond. The use of $NaBH_4$ led to a mixture of the desired product **1p** and byproduct **S3p**, deriving from reduction of **S2p**. On the contrary, $LiAlH_4$ afforded a completely selective reduction of the carbonyl unit. Therefore, the use of $LiAlH_4$ was extended to other ketones **S1p-s** and **S1u-w** for the preparation of the respective alcohols **1p-s** and **1u-w**.*

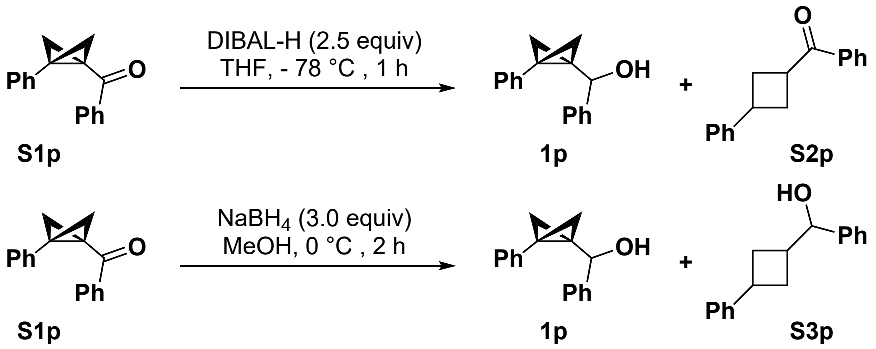

*<u>Note</u>: Alcohols **1** were found to be particularly sensitive to column chromatography on silica gel, with very low recovery, even after fast purifications or passivation of SiO_2 with TEA. Furthermore, they showed to undergo a slow decomposition, probably through polimerization, in the solid state at room temperature. To ensure samples of high purity and long life-time, they were dissolved, straight after preparation, in anhydrous ACN (0.1 M solution) and stored at – 20 °C under Ar.*

2.3 Preparation and Characterization of ***d₄*-S1a**

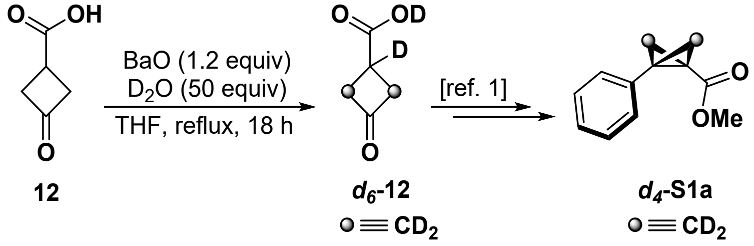

Product ***d*$_4$-1a** (*vide infra*) was obtained by reduction of ***d*$_4$-S1a** following the general procedure. Product ***d*$_4$-S1a** was obtained following the same procedure reported for all other **S1** materials,[1] starting from ***d*$_6$-12**. This was obtained by deuteration of **12** following an unmodified reported procedure.[11]

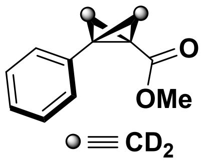

d*$_4$-S1a** White solid. **m.p.** 68 – 80 °C. **^{1}H NMR** (600 MHz, $CDCl_3$) δ 7.29 – 7.24 (m, 4H), 7.22 – 7.19 (m, 1H), 3.45 (s, 3H). **^{13}C NMR** (151 MHz, $CDCl_3$) δ 170.1, 133.6, 128.5 (2C), 127.0, 125.9 (2C), 51.8, 32.7, 23.0, the CD_2 peak was not detected. **HRMS *(ESI) m/z $[M+Na]^+$ calcd. for $C_{12}H_8D_4NaO_2^+$: 215.00981; found 215.0990.

2.4 Characterization of alcohols **1a-s** and **1u-x**.

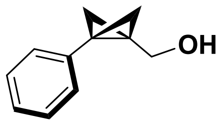

1a. White wax. **Yield** = 97% (0.29 mmol, 46.4 mg). **^{1}H NMR** (600 MHz, $CDCl_3$) δ 7.29 – 7.24 (m, 1H, partially overlapped with residual solvent signal), 7.24 – 7.21 (m, 2H), 7.17 – 7.13 (m, 1H), 3.90 (s, 2H), 2.26 (s, 2H), 1.22 (s, 2H), the –OH signal was not detected in the spectrum. **^{13}C NMR** (151 MHz, $CDCl_3$) δ 135.8, 127.5 (2C), 124.2 (2C), 124.2, 61.2, 32.2 (2C), 22.8, 16.5. **HRMS *(ESI)*** m/z $[M+Na]^+$ calcd. for $C_{11}H_{12}NaO^+$: 183.0780; found 183.0781.

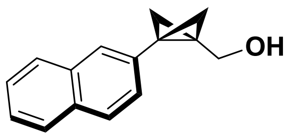

1b White wax. **Yield** = 93% (0.28 mmol, 58.8 mg). **^{1}H NMR** (600 MHz, $CDCl_3$) δ 7.79 (dd, J = 8.23, 1.26 Hz, 1H), 7.78 – 7.74 (m, 2H), 7.68 (d, J = 1.82 Hz, 1H), 7.46 (ddd, J = 8.21, 6.75, 1.43 Hz, 1H), 7.40 (ddd, J = 8.10, 6.78, 1.35 Hz, 1H), 7.35 (dd, J = 8.56, 1.84 Hz, 1H), 3.93 (s, 2H), 2.40 (d, J = 0.76 Hz, 2H), 1.32 (s, 2H), the –OH signal was not detected in the spectrum. **^{13}C NMR** (151 MHz, $CDCl_3$) δ 134.2, 133.3, 131.3, 127.7, 127.3, 126.7, 126.0, 124.6, 123.4, 123.2, 61.8, 33.1 (2C), 24.1, 17.7. **HRMS *(ESI)*** m/z $[M+Na]^+$ calcd. for $C_{15}H_{14}NaO^+$: 233.0937; found 233.0941.

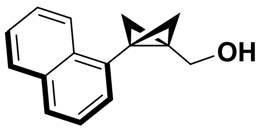

1c. White wax. **Yield** = 90% (0.27 mmol, 56.7 mg). **^{1}H NMR** (600 MHz, $CDCl_3$) δ ^{1}H NMR (600 MHz, $CDCl_3$) δ 8.42 – 8.37 (m, 1H), 7.85 (dd, J = 8.20, 1.38 Hz, 1H), 7.73 (d, J = 8.17 Hz, 1H), 7.53 – 7.46 (m, 2H), 7.40 – 7.36 (m, 1H), 7.25 (dd, J = 7.17, 1.23 Hz, 1H), 4.35 (d, J = 5.70 Hz, 2H), 2.07 (s, 2H), 1.43 (s, 2H), the –OH signal was not detected in the spectrum. **^{13}C NMR** (151 MHz, $CDCl_3$) δ 134.7, 134.2, 133.7, 128.7, 126.9, 126.0, 126.0, 125.7, 125.4, 123.4, 61.6, 36.9 (2C), 20.9, 15.4. **HRMS *(ESI)*** m/z $[M+Na]^+$ calcd. for $C_{15}H_{14}NaO^+$: 233.0937; found 233.0939.

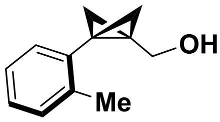

1d. White wax. **Yield** = 90% (0.27 mmol, 47.0 mg). **^{1}H NMR** (600 MHz, $CDCl_3$) δ 7.18 – 7.15 (m, 1H), 7.12 – 7.09 (m, 2H), 7.04 – 7.01 (m, 1H), 4.27 (d, J = 4.73 Hz, 2H), 2.44 (s, 3H), 1.91 (s, 2H), 1.57 (s, 1H), 1.20 (s, 2H), the –OH signal was not detected in the spectrum. **^{13}C NMR** (151 MHz, $CDCl_3$) δ 139.2, 135.0, 130.8, 126.5, 126.4, 125.9, 61.8, 36.1 (2C), 21.0, 20.8, 16.3. **HRMS *(ESI)*** m/z $[M+Na]^+$ calcd. for $C_{12}H_{14}NaO^+$: 197.0937; found 197.0942.

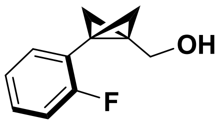

1e. White wax. **Yield** = 90% (0.27 mmol, 48.1 mg). **^{1}H NMR** (600 MHz, $CDCl_3$) δ 7.32 – 7.28 (m, 1H), 7.19 – 7.13 (m, 1H), 7.11 – 7.07 (m, 1H), 7.03 – 6.98 (m, 1H), 4.11 (s, 2H), 2.25 (d, J = 1.4 Hz, 2H), 1.37 (s, 1H), 1.21 (d, J = 1.3 Hz, 2H). **^{13}C NMR** (151 MHz, $CDCl_3$) δ 161.1 (d, J = 245.7 Hz), 130.7 (d, J = 5.1 Hz), 127.0 (d, J = 8.0 Hz), 124.2 (d, J = 13.1 Hz), 124.1 (d, J = 3.7 Hz), 115.5 (d, J = 21.8 Hz), 61.9 (d, J = 1.1 Hz), 34.8, 34.7, 21.8, 14.6. **^{19}F NMR** (565 MHz, CDCl3) δ -115.15 – -115.21 (m, 1H). **HRMS *(ESI)*** m/z $[M+Na]^+$ calcd. for $C_{11}H_{11}FNaO^+$: 201.0686; found 201.0689.

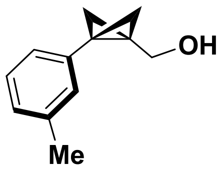

1f. White wax. **Yield** = 87% (0.26 mmol, 45.2 mg). **^{1}H NMR** (600 MHz, $CDCl_3$) δ 7.17 (t, *J* = 7.50 Hz, 1H), 7.07 – 7.00 (m, 2H), 6.97 (d, *J* = 7.54 Hz, 1H), 3.92 (d, *J* = 5.29 Hz, 2H), 2.33 (s, 3H), 2.26 (s, 2H), 1.21 (s, 2H), the –OH signal was not detected in the spectrum. **^{13}C NMR** (151 MHz, $CDCl_3$) δ 138.4, 136.9, 128.7, 126.4, 126.3, 122.6, 62.6, 33.5 (2C), 24.0, 21.8, 17.8. **HRMS *(ESI)*** m/z $[M+Na]^+$ calcd. for $C_{12}H_{14}NaO^+$: 197.0937; found 197.0929.

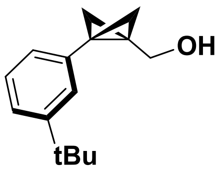

1g. White wax. **Yield** = 97% (0.29 mmol, 62.6 mg). **^{1}H NMR** (600 MHz, $CDCl_3$) δ 7.26 – 7.24 (m, 1H), 7.22 – 7.19 (m, 2H), 7.05 (dt, *J* = 7.17, 1.71 Hz, 1H), 3.92 (d, *J* = 1.93 Hz, 2H), 2.27 (s, 2H), 2.00 (s, 1H), 1.31 (s, 9H), 1.22 (s, 2H).**^{13}C NMR** (151 MHz, $CDCl_3$) δ 150.4, 135.2, 127.2, 121.4, 121.3, 121.3, 61.4, 33.6, 32.3 (2C), 30.3 (3C), 22.4, 16.7. **HRMS *(ESI)*** m/z $[M+Na]^+$ calcd. for $C_{15}H_{20}NaO^+$: 239.1406; found 239.1400.

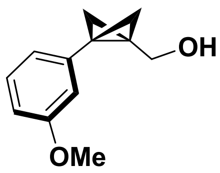

1h. White wax. **Yield** = 90% (0.27 mmol, 51.3 mg). **^{1}H NMR** (600 MHz, $CDCl_3$) δ 7.20 (t, *J* = 7.93 Hz, 1H), 6.82 (ddd, *J* = 7.66, 1.69, 0.92 Hz, 1H), 6.79 – 6.75 (m, 1H), 6.70 (ddd, *J* = 8.20, 2.59, 0.92 Hz, 1H), 3.91 (d, *J* = 3.41 Hz, 2H), 3.80 (s, 3H), 2.26 (s, 2H), 1.63 (s, 1H), 1.22 (s, 2H). **^{13}C NMR** (151 MHz, $CDCl_3$) δ 158.8, 137.5, 128.5, 116.6, 110.3, 109.4, 61.1, 54.1, 32.3 (2C), 23.1, 16.5. **HRMS *(ESI)*** m/z $[M+Na]^+$ calcd. for $C_{12}H_{14}NaO_2^+$: 213.0886; found 213.0888.

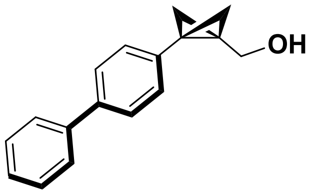

1i. White wax. **Yield** = 93% (0.28 mmol, 66.1 mg). **^{1}H NMR** (600 MHz, $CDCl_3$) δ 7.59 – 7.56 (m, 2H), 7.53 – 7.50 (m, 2H), 7.45 – 7.41 (m, 2H), 7.35 – 7.32 (m, 1H), 7.32 – 7.28 (m, 2H), 3.95 (s, 2H), 2.32 (s, 2H), 1.27 (s, 2H), the –OH signal was not detected in the spectrum; **^{13}C NMR** (151 MHz, $CDCl_3$) δ 140.8, 138.1, 136.0, 128.8 (2C), 127.2 (2C), 127.1, 126.8 (2C), 125.7 (2C), 62.3, 33.4 (2C), 24.3, 17.5. **HRMS *(ESI)*** m/z $[M+Na]^+$ calcd. for $C_{17}H_{16}NaO^+$: 259.1093; found 259.1088.

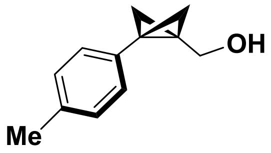

1j. White wax. **Yield** = 93% (0.28 mmol, 48.7 mg). **^{1}H NMR** (600 MHz, $CDCl_3$) δ 7.14 – 7.11 (m, 2H), 7.11 – 7.07 (m, 2H), 3.90 (d, *J* = 5.11 Hz, 2H), 2.32 (s, 3H), 2.24 (s, 2H), 1.20 (s, 2H), the –OH signal was not detected in the spectrum. **^{13}C NMR** (151 MHz, $CDCl_3$) δ 135.0, 133.6, 129.4 (2C), 125.4 (2C), 62.6, 33.4 (2C), 23.3, 21.2, 17.5. **HRMS *(ESI)*** m/z $[M+Na]^+$ calcd. for $C_{12}H_{14}NaO^+$: 197.0937; found 197.0932.

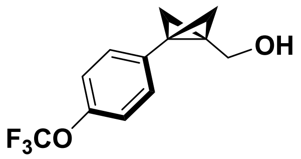

1k. White wax. **Yield** = 90% (0.27 mmol, 65.9 mg). **^{1}H NMR** (600 MHz, $CDCl_3$) δ 7.26 – 7.23 (m, 2H), 7.16 – 7.12 (m, 2H), 3.95 (s, 2H), 2.26 (s, 2H), 1.27 (s, 2H). **^{13}C NMR** (151 MHz, $CDCl_3$) δ 145.9 (q, J = 1.7 Hz), 134.9, 125.5 (2C), 120.2 (2C), 119.5 (q, J = 256 Hz), 60.9, 32.4 (2C), 23.4, 16.2. **^{19}F NMR** (565 MHz, $CDCl_3$) δ -57.96 (s, 3F). **HRMS *(ESI)*** m/z $[M+Na]^+$ calcd. for $C_{12}H_{11}F_3NaO_2^+$: 267.0603; found 267.0607.

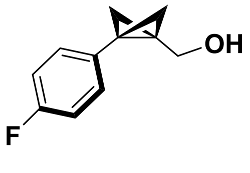

1l. White wax. **Yield** = 93% (0.28 mmol, 49.8 mg). **^{1}H NMR** (600 MHz, $CDCl_3$) δ 7.22 – 7.15 (m, 2H), 7.02 – 6.95 (m, 2H), 3.92 (d, J = 5.2 Hz, 2H), 2.21 (s, 2H), 1.23 (s, 2H). **^{13}C NMR** (151 MHz, $CDCl_3$) δ 160.0 (d, J = 244.0 Hz), 131.4 (d, J = 3.2 Hz), 125.7 (d, J = 7.7 Hz, 2C), 114.4 (d, J = 21.5 Hz, 2C), 61.1, 32.2 (2C), 22.2, 16.1. **^{19}F NMR** (565 MHz, $CDCl_3$) δ -117.89 – -117.95 (m, 1F). **HRMS *(ESI)*** m/z $[M+Na]^+$ calcd. for $C_{11}H_{11}FNaO^+$: 201.0686; found 201.0681.

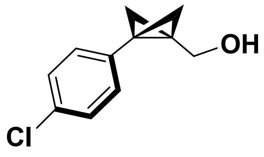

1m. White wax. **Yield** = 90% (0.27 mmol, 52.4 mg). **^{1}H NMR** (600 MHz, $CDCl_3$) δ 7.25 – 7.22 (m, 2H), 7.17 – 7.13 (m, 2H), 3.89 (s, 2H), 2.23 (s, 2H), 1.24 (s, 2H), the –OH signal was not detected in the spectrum. **^{13}C NMR** (151 MHz, $CDCl_3$) δ 135.8, 131.1, 128.8 (2C), 126.8 (2C), 62.1, 33.5 (2C), 24.5, 17.5. **HRMS *(ESI)*** m/z $[M+Na]^+$ calcd. for $C_{11}H_{11}ClNaO^+$: 217.0391; found 217.0395.

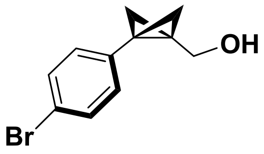

1n. White wax. **Yield** = 93% (0.28 mmol, 66.6 mg). **^{1}H NMR** (600 MHz, $CDCl_3$) δ 7.40 – 7.36 (m, 2H), 7.11 – 7.07 (m, 2H), 3.89 (s, 2H), 2.23 (s, 2H), 1.58 (s, 1H), 1.24 (s, 2H). **^{13}C NMR** (151 MHz, $CDCl_3$) δ 135.9, 131.3 (2C), 126.7 (2C), 118.5, 61.7, 33.1, 24.3, 17.1. **HRMS *(ESI)*** m/z $[M+Na]^+$ calcd. for $C_{11}H_{11}BrNaO^+$: 260.9885; found 260.9892.

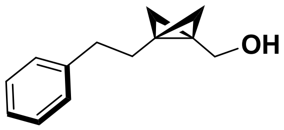

1o. White wax. **Yield** = 94% (0.28 mmol, 53.0 mg). **^{1}H NMR** (600 MHz, $CDCl_3$) δ 7.31 – 7.26 (m, 3H), 7.22 – 7.19 (m, 2H), 3.95 (s, 2H), 2.79 – 2.73 (m, 2H), 2.18 – 2.12 (m, 2H), 1.38 (s, 2H), 0.70 (s, 2H). **^{13}C NMR** (151 MHz, $CDCl_3$) δ 141.9, 128.4 (2C), 128.4 (2C), 125.9, 62.4, 35.6, 33.4 (2C), 28.5, 14.5, 13.3. **HRMS *(ESI)*** m/z $[M+Na]^+$ calcd. for $C_{13}H_{16}NaO^+$: 211.1093; found 211.1101.

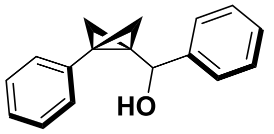

(+/-)-**1p.** White wax. **Yield** = 80% (0.24 mmol, 56.6 mg). **^{1}H NMR** (^{1}H NMR (600 MHz, $CDCl_3$) δ 7.29 – 7.25 (m, 5H), 7.19 – 7.14 (m, 5H), 4.87 (s, 1H), 2.47 (dd, J = 6.57, 0.76 Hz, 1H), 2.06 (dd, J = 6.59, 0.98 Hz, 1H), 1.23 (d, J = 0.94 Hz, 1H), 1.19 (d, J = 1.02 Hz, 1H), the -OH signal was not detected in the spectrum. **^{13}C NMR** (151 MHz, $CDCl_3$) δ 142.1, 136.3, 128.4 (2C), 128.3 (2C), 127.7, 126.5 (2C), 125.5 (2C), 125.3, 72.8, 32.5, 31.6, 30.9, 18.2. **HRMS *(ESI)*** m/z $[M+Na]^+$ calcd. for $C_{17}H_{16}NaO^+$: 259.1093; found 259.1088.

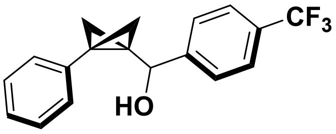

(+/-)-**1q.** White wax. **Yield** = 87% (0.26 mmol, 79.0 mg). **^{1}H NMR** (^{1}H NMR (600 MHz, $CDCl_3$) δ 7.55 – 7.50 (m, 2H), 7.33 – 7.25 (m, 4H, partially overlapped with residual solvent signal), 7.23 – 7.19 (m, 1H), 7.18 – 7.15 (m, 2H), 4.96 (d, J = 2.8 Hz, 1H), 2.47 (dd, J = 6.6, 0.9 Hz, 1H), 2.08 (dd, J = 6.6, 1.1 Hz, 1H), 1.84 (d, J = 3.5 Hz, 1H), 1.25 (s, 1H), 1.23 (s, 1H). **^{13}C NMR** (151 MHz, $CDCl_3$) δ 170.2, 144.7 (q, J = 1.7 Hz), 134.7, 128.7 (q, J = 32.4 Hz), 127.5 (2C), 125.7 (2C), 124.5, 124.4 (2C), 124.1 (q, J = 3.2 Hz, 2C), 123.1 (q, J = 272.0 Hz), 71.2, 59.4, 31.3, 29.8, 26.5. **^{19}F NMR** (565 MHz, $CDCl_3$) δ -62.46 (s, 3F). **HRMS *(ESI)*** m/z $[M+Na]^+$ calcd. for $C_{18}H_{15}F_3NaO^+$: 327.0967; found 327.0961.

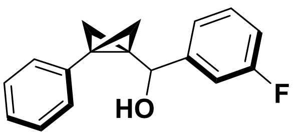

(+/-)-**1r.** White wax. **Yield** = 83% (0.25 mmol, 63.5 mg). **^{1}H NMR** (^{1}H NMR (600 MHz, $CDCl_3$) 7.31 – 7.27 (m, 2H), 7.24 – 7.18 (m, 2H), 7.18 – 7.13 (m, 2H), 6.97 – 6.86 (m, 3H), 4.88 (s, 1H), 2.46 (dd, J = 6.6, 0.9 Hz, 1H), 2.08 (dd, J = 6.6, 1.1 Hz, 1H), 1.23 (s, 1H), 1.21 (s, 1H); **^{13}C NMR** (151 MHz, $CDCl_3$) δ 162.8 (d, J = 246.8 Hz), 144.5 (d, J = 7.1 Hz), 136.0, 129.7 (d, J = 8.2 Hz), 128.5 (2C), 125.5 (3C), 122.0 (d, J = 2.7 Hz), 114.5 (d, J = 21.1 Hz), 113.5 (d, J = 21.9 Hz), 72.2 (d, J = 1.6 Hz), 32.4, 30.8, 27.5, 18.4. **^{19}F NMR** (565 MHz, $CDCl_3$) δ -117.92 – -118.53 (m, 1F). **HRMS *(ESI)*** m/z $[M+Na]^+$ calcd. for $C_{17}H_{15}FNaO^+$: 277.0999; found 277.1115.

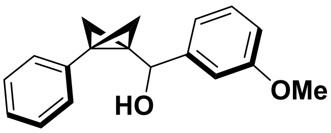

(+/-)-**1s.** White wax. **Yield** = 80% (0.24 mmol, 63.8 mg). **^{1}H NMR** (600 MHz, $CDCl_3$)δ 7.28 – 7.26 (m, 2H), 7.21 – 7.13 (m, 4H), 6.83 – 6.73 (m, 2H), 6.68 (t, *J* = 2.09 Hz, 1H), 4.86 (s, 1H), 3.69 (s, 3H), 2.44 (dd, *J* = 6.56, 0.78 Hz, 1H), 2.06 (dd, *J* = 6.61, 0.96 Hz, 1H), 1.43 (s, 1H), 1.22 (s, 1H), 1.18 (s, 1H). **^{13}C NMR** (151 MHz, $CDCl_3$) δ 159.7, 143.8, 136.5, 129.5, 128.6 (2C), 125.8 (2C), 125.5, 118.9, 113.8, 111.8, 72.9, 55.3, 32.5, 31.2, 28.0, 18.5. **HRMS *(ESI)*** m/z $[M+Na]^+$ calcd. for $C_{18}H_{18}NaO_2^+$: 289.1199; found 289.1204.

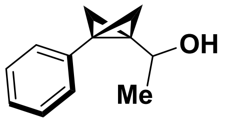

(+/-)-**1u.** White wax. **Yield** = 87% (0.26 mmol, 45.2 mg). **^{1}H NMR** (600 MHz, $CDCl_3$) δ 7.30 – 7.26 (m, 2H), 7.24 – 7.20 (m, 2H), 7.17 – 7.13 (m, 1H), 3.99 (q, *J* = 6.35 Hz, 1H), 2.29 (dd, *J* = 6.63, 0.86 Hz, 1H), 2.23 (dd, *J* = 6.57, 0.98 Hz, 1H), 1.14 (s, 1H), 1.12 (d, *J* = 6.36 Hz, 3H), 1.10 (s, 1H), the –OH signal was not detected in the spectrum. **^{13}C NMR** (151 MHz, $CDCl_3$) δ 136.5, 128.5 (2C), 125.3 (2C), 125.1, 66.8, 31.4, 30.8, 27.8, 21.4, 18.2. **HRMS *(ESI)*** m/z $[M+Na]^+$ calcd. for $C_{12}H_{14}NaO^+$: 197.0937; found 197.0930.

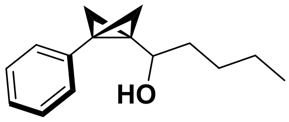

(+/-)-**1v.** White wax. **Yield** = 90% (0.27 mmol, 58.3 mg). **^{1}H NMR** (600 MHz, $CDCl_3$) δ 7.29 – 7.26 (m, 2H), 7.25 – 7.22 (m, 2H), 7.16 – 7.12 (m, 1H), 3.80 (dd, *J* = 7.82, 4.92 Hz, 1H), 2.36 (dd, *J* = 6.54, 0.81 Hz, 1H), 2.19 (dd, *J* = 6.55, 0.95 Hz, 1H), 1.62 – 1.55 (m, 2H), 1.50 – 1.36 (m, 3H), 1.34 – 1.22 (m, 2H), 1.14 (s, 1H), 1.09 (s, 1H), 0.84 (t, *J* = 7.19 Hz, 3H). **^{13}C NMR** (151 MHz, $CDCl_3$) δ 137.1, 128.8 (2C), 125.4 (2C), 125.3, 70.4, 36.5, 32.5, 30.4, 27.8, 27.7, 22.7, 17.5, 14.2. **HRMS *(ESI)*** m/z $[M+Na]^+$ calcd. for $C_{15}H_{20}NaO^+$: 239.1406; found 239.1411.

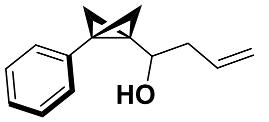

(+/-)-**1w.** Yellow wax. **Yield** = 93% (0.28 mmol, 56.0 mg). **^{1}H NMR** δ 7.30 – 7.19 (m, 4H, partially overlapped with residual solvent signal) , 7.16 – 7.10 (m, 1H), 5.86 – 5.72 (m, 1H), 5.12 – 5.03 (m, 2H), 3.84 (dd, *J* = 7.78, 4.73 Hz, 1H), 2.37 – 2.33 (m, 1H), 2.33 – 2.19 (m, 2H), 2.19 – 2.16 (m, 1H), 1.14 (s, 1H), 1.10 (s, 1H). **^{13}C NMR** (151 MHz, $CDCl_3$) δ 136.7, 134.4, 128.5, 125.4, 125.2, 117.9, 69.3, 41.1, 32.1, 30.6, 26.9, 22.6, 17.6, 14.1. **HRMS *(ESI)*** m/z $[M+Na]^+$ calcd. for $C_{14}H_{16}NaO^+$: 223.1093; found 223.2085.

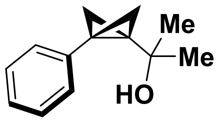

1x.[12] Yellow wax. **Yield** = 88% (0.26 mmol, 49.6 mg). **^{1}H NMR** (600 MHz, $CDCl_3$) δ 7.30 – 7.24 (m, 4H, partially overlapped with the residual solvent signal), 7.16 – 7.12 (m, 1H), 2.27 (s, 2H), 1.33 (s, 1H) 1.17 (s, 6H), 0.97 (s, 2H). **^{13}C NMR** (151 MHz, $CDCl_3$) δ 135.9, 128.2 (2C), 125.4 (2C), 124.8, 70.0, 31.5, 29.3 (2C), 27.7 (2C), 19.6. **HRMS *(ESI)*** m/z $[M+Na]^+$ calcd. for $C_{13}H_{16}NaO^+$: 211.1093; found 211.1099.

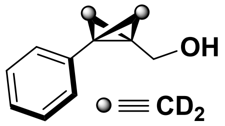

d_4*-1a** White wax. **Yield** = 97% (0.29 mmol, 58.8 mg). **^{1}H NMR** (600 MHz, $CDCl_3$) δ 7.30 – 7.25 (m, 2H, partially overlapped with residual solvent signal), 7.24 – 7.20 (m, 2H), 7.17 – 7.13 (m, 1H), 3.90 (d, *J* = 5.02 Hz, 2H), 1.17 (t, *J* = 6.49 Hz, 1H). **^{13}C NMR** (151 MHz, $CDCl_3$) δ 135.8, 127.5 (2C), 124.2 (2C), 124.2, 61.2, 29.3, 22.5. **HRMS *(ESI) m/z $[M+Na]^+$ calcd. for $C_{11}H_8D_4NaO^+$: 187.1031; found 187.1033.

3. Preparation and Characterization of Products 3.

3.1 General Procedure

The ElectraSyn vial (5 mL), equipped with a stir bar, was charged with the appropriate electroactive precursor **2** (0.20 mmol, 2.0 equiv.) and $LiClO_4$ (0.30 mmol, 31.9 mg). The ElectraSyn vial cap, equipped with anode (C) and cathode (Ni), was inserted into the mixture and closed with a rubber septum. Then THF (2.0 mL) and a solution of alcohol **1** in ACN (0.1 mmol, 1.0 mL, 0.1 M) were added, the mixture stirred until complete dissolution of the solids occurred and then cooled to -30 °C. The reaction mixture was electrolysed at a constant current of 10.0 mA at -30 °C, until the prescribed number of electrons ($F/mol_{\mathbf{1}}$) was reached (see single cases below). Then, the ElectraSyn vial cap was removed, and the electrodes and vial were rinsed with EtOAc (5 mL) and $NH_4Cl_{(aq)}$ (saturated, 5 mL) and water (5 mL), which were combined with the crude mixture in a separatory funnel. Then, the organic layer was separated, and the aqueous layer was extracted with EtOAc (2 x 5 mL). The combined organic layers were washed with $NH_4Cl_{(aq)}$ (0.1 M, 3 x 5 mL), dried over Na_2SO_4 and concentrated in vacuo. The crude product was finally purified by FC on silica gel to afford pure products **3**.

Note A: *The Electrasyn 2.0 electrochemical workstation and all the electrodes for the preparative electrolyses were purchased from IKA. All the electrodes have the shape of a rectangular parallelepiped with 5.3 cm x 0.8 cm x 0.2 cm dimensions resulting in a wet surface area of 1.2 cm^2 in the case of the 0.1 mmol scale reaction and 2.8 cm^2 for the 1.0 mmol scale reaction. The distance between the electrodes in this set-up is fixed and equal to 0.5 cm.*

Note B: *The reaction outcome was not found to be particularly sensitive to stirring. A fixed stirring rate of 520 rpm was adopted in all cases.*

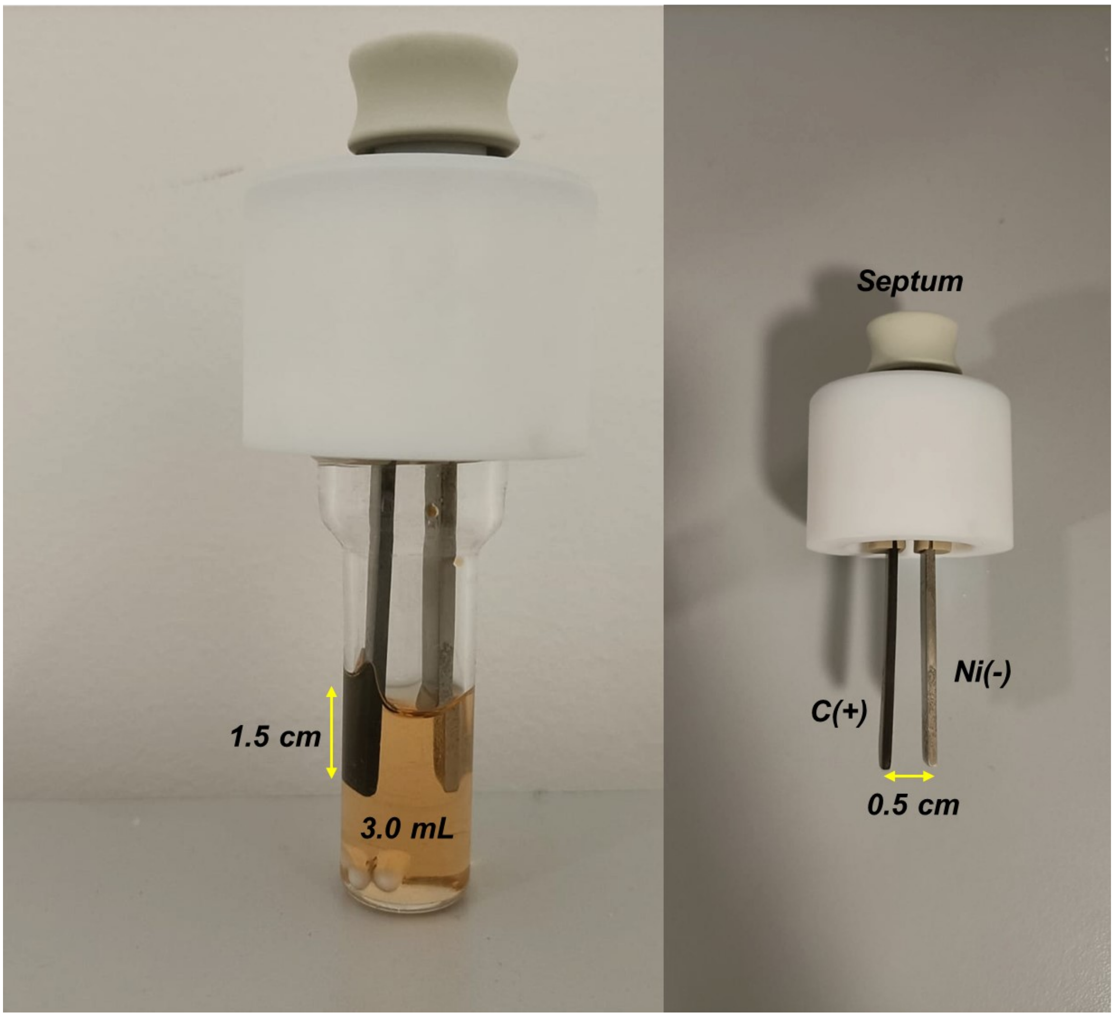

Figure S1. Electrasyn vial cap equipped with C anode and Ni cathode (right). Electrasyn vial (equipped with electrodes and cap) filled with the reaction mixture (left).

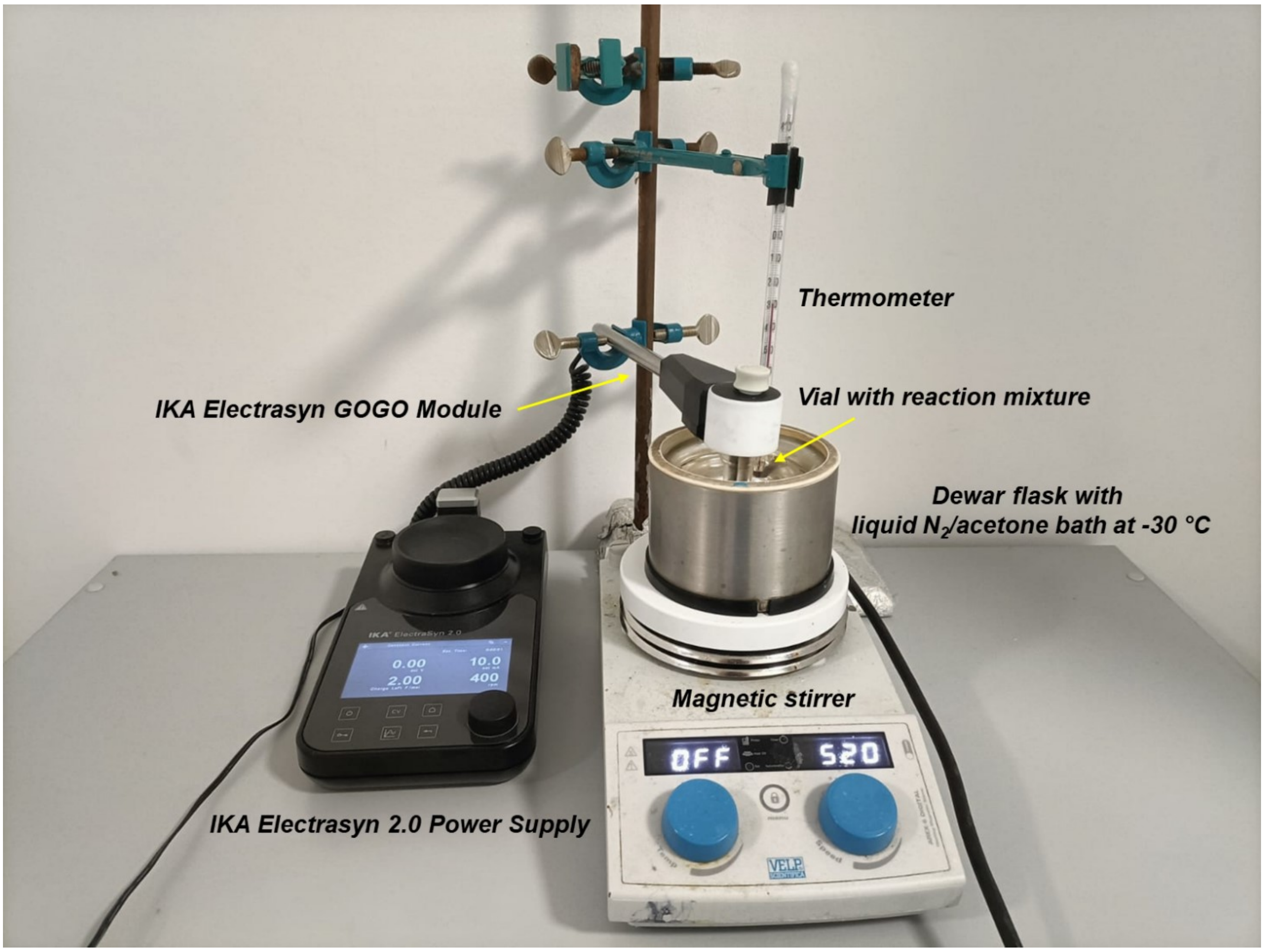

Figure S2. Complete reaction set-up. The IKA Electrasyn GOGO Module is an extension that allows reactions in ElectraSyn 2.0 vials away from the device.

3.2 Characterization of Products **3**

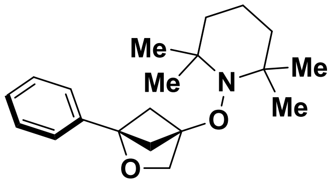

3aa. Obtained from alcohol **1a** and TEMPO **2a** (1.0 F/mol$_{\mathbf{1a}}$). White solid. **m.p.** 101 - 103 °C. **FC eluent**: *n*Hex/EtOAc 50:1. **Yield** = 75% (0.075 mmol, 23.6 mg). **^{1}H NMR** (600 MHz, $CDCl_3$) δ 7.42 – 7.38 (m, 2H), 7.37 – 7.32 (m, 2H), 7.31 – 7.26 (m, 1H), 3.95 (s, 2H), 2.34 – 2.18 (m, 4H), 1.66 – 1.39 (m, 5H), 1.38 – 1.30 (m, 1H), 1.17 (s, 6H), 1.13 (s, 6H). **^{13}C NMR** (151 MHz, $CDCl_3$) δ 138.1, 128.2 (2C), 127.7, 126.0 (2C), 83.4, 81.9, 69.1 (2C), 59.3, 49.9 (2C), 39.8 (2C), 33.8 (2C), 20.4 (2C), 17.1. **HRMS *(ESI)*** m/z [M+Na]$^+$ calcd. for $C_{20}H_{29}NNaO_2^+$: 338.2090; found 338.2095.

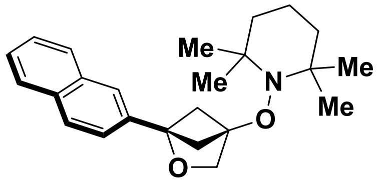

3ba. Obtained from alcohol **1b** and TEMPO **2a** (1.0 F/mol$_{\mathbf{1b}}$). White solid. **m.p.** 109 - 111 °C. **FC eluent**: *n*Hex/EtOAc 50:1. **Yield** = 52% (0.052 mmol, 20.0 mg). **^{1}H NMR** (600 MHz, $CDCl_3$) δ 7.86 (s, 1H), 7.85 – 7.80 (m, 3H), 7.53 – 7.49 (m, 1H), 7.49 – 7.44 (m, 2H), 4.01 (s, 2H), 2.43 – 2.35 (m, 2H), 2.35 – 2.26 (m, 2H), 1.65 – 1.55 (m, 1H), 1.55 – 1.43 (m, 4H), 1.39 – 1.32 (m, 1H), 1.20 (s, 6H), 1.15 (s, 6H). **^{13}C NMR** (151 MHz, $CDCl_3$) δ 135.8, 133.3, 133.1, 128.2, 128.1, 127.8, 126.3, 126.1, 125.1, 124.3, 83.8, 82.3, 69.4, 59.6 (2C), 50.4 (2C), 40.0 (2C), 34.1 (2C), 20.6 (2C), 17.3. **HRMS *(ESI)*** m/z [M+Na]$^+$ calcd. for $C_{24}H_{31}NNaO_2^+$: 388.2247; found 388.2251.

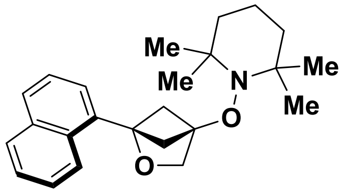

3ca. Obtained from alcohol **1c** and TEMPO **2a** (1.0 F/mol$_{\mathbf{1c}}$). White solid. **m.p.** 105 - 107 °C. **FC eluent**: *n*Hex/EtOAc 50:1. **Yield** = 61% (0.061 mmol, 22.3 mg). **^{1}H NMR** (600 MHz, $CDCl_3$) δ 8.44 (d, *J* = 8.8 Hz, 1H), 7.85 (d, *J* = 7.3 Hz, 1H), 7.82 (dd, *J* = 7.4, 2.1 Hz, 1H), 7.52 (ddd, *J* = 8.4, 6.8, 1.5 Hz, 1H), 7.48 (ddd, *J* = 8.0, 6.8, 1.4 Hz, 1H), 7.44 – 7.39 (m, 2H), 4.09 (s, 2H), 2.68 – 2.61 (m, 2H), 2.44 – 2.37 (m, 2H), 1.66 – 1.57 (m, 1H), 1.56 – 1.44 (m, 4H), 1.39 – 1.33 (m, 1H), 1.23 (s, 6H), 1.16 (s, 6H). **^{13}C NMR** (151 MHz, $CDCl_3$) δ 132.9, 132.6, 130.9, 128.3, 127.4, 125.2, 124.9, 124.7, 124.6, 123.8, 82.1, 81.9, 68.5, 58.4 (2C), 48.7 (2C), 38.8 (2C), 32.9 (2C), 19.4 (2C), 16.1. **HRMS *(ESI)*** m/z [M+Na]$^+$ calcd. for $C_{24}H_{31}NNaO_2^+$: 388.2247; found 388.2242.

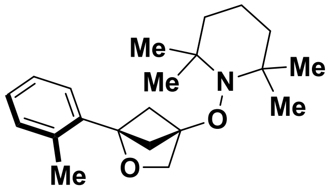

3da. Obtained from alcohol **1d** and TEMPO **2a** (1.0 F/mol$_{\mathbf{1d}}$). White solid. **m.p.** 94 - 96 °C. **FC eluent**: *n*Hex/EtOAc 50:1. **Yield** = 67% (0.067 mmol, 22.0 mg). **^{1}H NMR** (600 MHz, $CDCl_3$) ^{1}H NMR (600 MHz, $CDCl_3$) δ 7.25 – 7.22 (m, 1H), 7.22 – 7.19 (m, 1H), 7.17 – 7.12 (m, 2H), 3.92 (s, 2H), 2.46 (s, 3H), 2.45 – 2.42 (m, 2H), 2.29 – 2.24 (m, 2H), 1.64 – 1.55 (m, 1H), 1.54 – 1.42 (m, 4H), 1.37 – 1.31 (m, 1H), 1.19 (s, 6H), 1.13 (s, 6H). **^{13}C NMR** (151 MHz, $CDCl_3$) δ 138.2, 135.5, 131.1, 128.7, 128.3, 125.7, 83.4, 82.7, 69.4, 59.5 (2C), 49.1 (2C), 40.0 (2C), 34.1 (2C), 20.6 (2C), 20.5, 17.3. **HRMS *(ESI)*** m/z $[M+Na]^+$ calcd. for $C_{21}H_{31}NNaO_2^+$: 352.2247; found 352.2245.

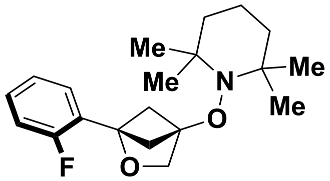

3ea. Obtained from alcohol **1e** and TEMPO **2a** (1.0 F/mol$_{\mathbf{1e}}$). White solid. **m.p.** 108 - 110 °C. **FC eluent**: *n*Hex/EtOAc 50:1. **Yield** = 50% (0.050 mmol, 16.7 mg). **^{1}H NMR** (600 MHz, $CDCl_3$) δ 7.41 (td, *J* = 7.6, 1.8 Hz, 1H), 7.30 – 7.26 (m, 1H), 7.11 (td, *J* = 7.5, 1.2 Hz, 1H), 7.03 (ddd, *J* = 10.9, 8.2, 1.2 Hz, 1H), 3.91 (s, 2H), 2.46 – 2.38 (m, 2H), 2.34 – 2.26 (m, 2H), 1.64 – 1.54 (m, 1H), 1.53 – 1.42 (m, 4H), 1.36 – 1.31 (m, 1H), 1.17 (s, 6H), 1.12 (s, 6H). **^{13}C NMR** (151 MHz, $CDCl_3$) δ 159.9 (d, *J* = 249.1 Hz), 128.7 (d, *J* = 8.3 Hz), 127.9 (d, *J* = 4.1 Hz), 124.0 (d, *J* = 13.1 Hz), 122.8 (d, *J* = 3.7 Hz), 114.6 (d, *J* = 21.8 Hz), 83.0, 77.4, 67.4, 58.3 (2C), 48.5 (d, *J* = 2.1 Hz, 2C), 38.7 (2C), 32.7 (2C), 19.3 , 16.0. **^{19}F NMR** (565 MHz, $CDCl_3$) δ -115.17 – -115.72 (m, 1F). **HRMS *(ESI)*** m/z $[M+Na]^+$ calcd. for $C_{20}H_{28}FNNaO_2^+$: 356.1996; found 256.1999.

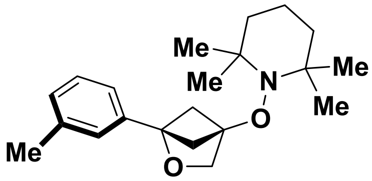

3fa. Obtained from alcohol **1f** and TEMPO **2a** (1.0 F/mol$_{\mathbf{1f}}$). White solid. **m.p.** 115 - 117 °C. **FC eluent**: *n*Hex/EtOAc 50:1. **Yield** = 67% (0.067 mmol, 22.0 mg). **^{1}H NMR** (600 MHz, $CDCl_3$) δ 7.25 – 7.22 (m, 2H), 7.18 (d, *J* = 9.33 Hz, 1H), 7.10 (d, *J* = 7.59 Hz, 1H), 3.94 (s, 2H), 2.35 (s, 3H), 2.31 – 2.26 (m, 2H), 2.25 – 2.20 (m, 2H), 1.64 – 1.55 (m, 1H), 1.54 – 1.42 (m, 4H), 1.37 – 1.31 (m, 1H), 1.17 (s, 6H), 1.13 (s, 6H). **^{13}C NMR** (151 MHz, $CDCl_3$) δ 138.2, 138.0, 128.7, 128.3, 126.9, 123.3, 83.6, 82.1, 69.3, 59.5 (2C), 50.1 (2C), 40.0 (2C), 34.0 (2C), 21.6, 20.6 (2C), 17.3. **HRMS *(ESI)*** m/z $[M+Na]^+$ calcd. for $C_{21}H_{31}NNaO_2^+$: 352.2247; found 352.2244.

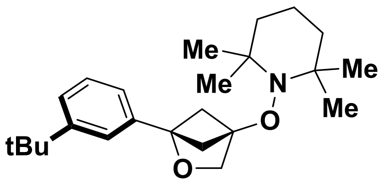

3ga. Obtained from alcohol **1g** and TEMPO **2a** (1.0 F/mol$_{\mathbf{1g}}$). White solid. **m.p.** 107- 109 °C. **FC eluent**: *n*Hex/EtOAc 50:1. **Yield** = 50% (0.050 mmol, 18.6 mg). **^{1}H NMR** (600 MHz, $CDCl_3$) δ 7.42 (t, *J* = 1.9 Hz, 1H), 7.33 (ddd, *J* = 7.9, 2.1, 1.3 Hz, 1H), 7.28 (t, *J* = 7.7 Hz, 1H), 7.19 (dt, *J* = 7.4, 1.4 Hz, 1H), 3.95 (s, 2H), 2.31 – 2.27 (m, 2H), 2.27 – 2.22 (m, 2H), 1.63 – 1.54 (m, 1H), 1.53 – 1.41 (m, 4H), 1.37 – 1.30 (m, 1H) partially overlapped with 1.32 (s, 9H), 1.17 (s, 6H), 1.13 (s, 6H). **^{13}C NMR** (151 MHz, $CDCl_3$) δ 150.1, 136.7, 126.9, 123.9, 122.3, 121.7, 82.4, 81.1, 68.1, 58.3 (2C), 48.9 (2C), 38.7 (2C), 33.7, 32.8 (2C), 30.3 (3C), 19.3 (2C), 16.0. **HRMS** ***(ESI)*** m/z [M+Na]$^+$ calcd. for $C_{24}H_{37}NNaO_2^+$: 394.2717; found 394.2722.

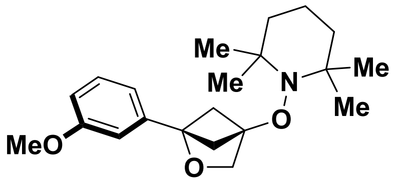

3ha. Obtained from alcohol **1h** and TEMPO **2a** (1.0 F/mol$_{\mathbf{1h}}$). White solid. **m.p.** 98 -100 °C. **FC eluent**: *n*Hex/EtOAc 30:1. **Yield** = 67% (0.067 mmol, 23.1 mg). **^{1}H NMR** (600 MHz, $CDCl_3$) δ 7.27 (t, *J* = 7.9 Hz, 1H), 7.00 – 6.95 (m, 2H), 6.84 (ddd, *J* = 8.2, 2.6, 1.0 Hz, 1H), 3.95 (s, 2H), 3.83 (s, 3H), 2.32 – 2.27 (m, 2H), 2.25 – 2.20 (m, 2H), 1.64 – 1.56 (m, 1H), 1.55 – 1.41 (m, 4H), 1.39 – 1.31 (m, 1H), 1.18 (s, 6H), 1.14 (s, 6H). **^{13}C NMR** (151 MHz, $CDCl_3$) δ 159.6, 139.8, 129.3, 118.4, 113.7, 111.3, 83.4, 81.9, 69.1, 59.4 (2C), 55.3, 50.1 (2C), 39.8 (2C), 33.9 (2C), 20.4 (2C), 17.1. **HRMS** ***(ESI)*** m/z [M+Na]$^+$ calcd. for $C_{21}H_{31}NNaO_3^+$: 368.2196; found 368.2203.

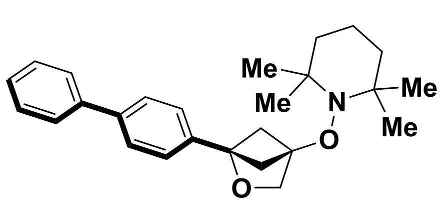

3ia. Obtained from alcohol **1i** and TEMPO **2a** (1.0 F/mol$_{\mathbf{1i}}$). White solid. **m.p.** 112 - 114 °C. **FC eluent**: *n*Hex/EtOAc 50:1. **Yield** = 69% (0.069 mmol, 27.0 mg). **^{1}H NMR** (600 MHz, $CDCl_3$) ^{1}H NMR (600 MHz, $CDCl_3$)δ 7.60 – 7.56 (m, 4H), 7.49 – 7.45 (m, 2H), 7.45 – 7.41 (m, 2H), 7.37 – 7.32 (m, 1H), 3.97 (s, 2H), 2.35 – 2.30 (m, 2H), 2.28 – 2.24 (m, 2H), 1.65 – 1.55 (m, 1H), 1.54 – 1.42 (m, 4H), 1.38 – 1.31 (m, 1H), 1.18 (s, 6H), 1.13 (s, 6H). **^{13}C NMR** (151 MHz, $CDCl_3$) δ 140.7, 140.4, 137.0, 128.5 (2C), 127.1, 126.9 (2C), 126.8 (2C), 126.3 (2C), 83.3, 81.5, 68.9, 59.2 (2C), 49.8 (2C), 39.6 (2C), 33.6 (2C), 20.2 (2C), 16.8. **HRMS** ***(ESI)*** m/z [M+Na]$^+$ calcd. for $C_{26}H_{33}NNaO_2^+$: 414.2404; found 414.2408.

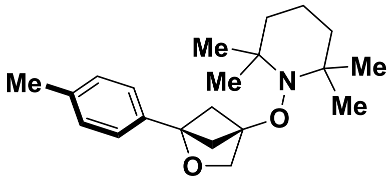

3ja. Obtained from alcohol **1j** and TEMPO **2a** (1.0 F/mol$_{\mathbf{1j}}$). White solid. **m.p.** 81 - 83 °C. **FC eluent**: *n*Hex/EtOAc 50:1. **Yield** = 83% (0.083 mmol, 27.3 mg). **^{1}H NMR** (600 MHz, $CDCl_3$) δ 7.22 – 7.19 (m, 2H), 7.09 – 7.06 (m, 2H), 3.86 (s, 2H), 2.27 (s, 3H), 2.21 – 2.16 (m, 2H), 2.16 – 2.12 (m, 2H), 1.56 – 1.47 (m, 1H), 1.46 – 1.34 (m, 4H), 1.30 – 1.24 (m, 1H), 1.09 (s, 6H), 1.05 (s, 6H). **^{13}C NMR** (151 MHz, $CDCl_3$) δ 136.4, 134.1, 127.8 (2C), 124.9 (2C), 82.4, 80.8, 68.1, 58.3 (2C), 48.8 (2C), 38.7 (2C), 32.8 (2C), 20.2, 19.3 (2C), 16.0. **HRMS *(ESI)*** m/z $[M+Na]^+$ calcd. for $C_{21}H_{31}NNaO_2^+$: 352.2247; found 352.2242.

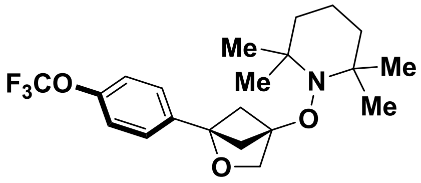

3ka. Obtained from alcohol **1k** and TEMPO **2a** (1.0 F/mol$_{\mathbf{1k}}$). White solid. **m.p.** 77 - 79 °C. **FC eluent**: *n*Hex/EtOAc 50:1. **Yield** = 63% (0.063 mmol, 25.1 mg). **^{1}H NMR** (600 MHz, $CDCl_3$) δ 7.43 – 7.39 (m, 2H), 7.20 – 7.15 (m, 2H), 3.94 (s, 2H), 2.31 – 2.26 (m, 2H), 2.24 – 2.19 (m, 2H), 1.63 – 1.55 (m, 1H), 1.54 – 1.42 (m, 4H), 1.37 – 1.31 (m, 1H), 1.16 (s, 6H), 1.12 (s, 6H). **^{13}C NMR** (151 MHz, $CDCl_3$) δ 148.7, 137.0, 127.6 (2C), 120.8 (2C), 120.5 (q, *J* = 257.2 Hz), 83.4, 81.3, 69.1, 59.4 (2C), 50.1 (2C), 39.8 (2C), 33.8 (2C), 20.4 (2C), 17.1. **^{19}F NMR** (565 MHz, $CDCl_3$) δ -57.86 (s, 3F). **HRMS *(ESI)*** m/z $[M+Na]^+$ calcd. for $C_{21}H_{28}F_3NNaO_3^+$: 422.1913; found $^+$: 422.1911.

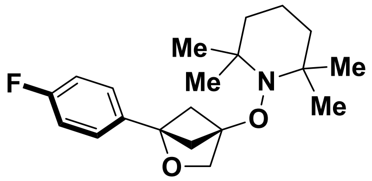

3la. Obtained from alcohol **1l** and TEMPO **2a** (1.0 F/mol$_{\mathbf{1l}}$). White solid. **m.p.** 75 - 77 °C. **FC eluent**: *n*Hex/EtOAc 50:1. **Yield** = 67% (0.067 mmol, 22.3 mg). **^{1}H NMR** (600 MHz, $CDCl_3$) δ 7.39 – 7.32 (m, 2H), 7.05 – 6.99 (m, 2H), 3.93 (s, 2H), 2.29 – 2.24 (m, 2H), 2.24 – 2.19 (m, 2H), 1.63 – 1.54 (m, 1H), 1.53 – 1.41 (m, 4H), 1.37 – 1.30 (m, 1H), 1.16 (s, 6H), 1.12 (s, 6H). **^{13}C NMR** (151 MHz, $CDCl_3$) δ 162.4 (d, *J* = 246.2 Hz), 134.0 (d, *J* = 3.2 Hz), 127.9 (d, *J* = 8.2 Hz, 2C), 115.1 (d, *J* = 21.4 Hz, 2C), 83.4, 81.4, 69.2, 59.4 (2C), 49.9 (2C), 39.8 (2C), 33.8 (2C), 20.4 (2C), 17.1. **^{19}F NMR** (565 MHz, $CDCl_3$) δ -114.46 – -114.54 (m, 1F). **HRMS *(ESI)*** m/z $[M+Na]^+$ calcd. for $C_{20}H_{28}FNNaO_2^+$: 356.1996; found 256.1991.

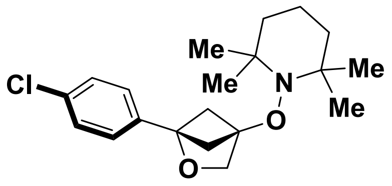

3ma. Obtained from alcohol **1n** and TEMPO **2a** (1.0 F/mol$_{\mathbf{1m}}$). White solid. **m.p.** 85 - 87 °C. **FC eluent**: *n*Hex/EtOAc 50:1. **Yield** = 67% (0.067 mmol, 23.4 mg). **^{1}H NMR** (600 MHz, $CDCl_3$) δ 7.34 – 7.29 (m, 4H), 3.93 (s, 2H), 2.30 – 2.24 (m, 2H), 2.24 – 2.16 (m, 2H), 1.64 – 1.53 (m, 1H), 1.53 – 1.40 (m, 4H), 1.37 – 1.31 (m, 1H), 1.16 (s, 6H), 1.12 (s, 6H). **^{13}C NMR** (151 MHz, $CDCl_3$) δ 136.9, 133.7, 128.5 (2C), 127.7 (2C), 83.6, 81.6, 69.3, 59.6 (2C), 50.3 (2C), 40.0 (2C), 34.0 (2C), 20.6 (2C), 17.2. **HRMS *(ESI)*** m/z $[M+Na]^+$ calcd. for $C_{20}H_{28}ClNNaO_2^+$: 372.1701; found 372.1706.

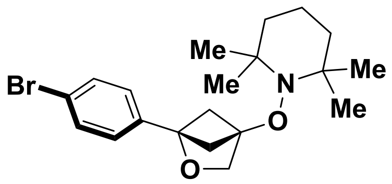

3na. Obtained from alcohol **1n** and TEMPO **2a** (1.0 F/mol$_{\mathbf{1n}}$). White solid. **m.p.** 102 - 104 °C. **FC eluent**: *n*Hex/EtOAc 50:1. **Yield** = 53% (0.053 mmol, 20.9 mg). **^{1}H NMR** (600 MHz, $CDCl_3$) δ 7.47 – 7.45 (m, 2H), 7.27 – 7.24 (m, 2H), 3.93 (s, 2H), 2.29 – 2.25 (m, 2H), 2.22 – 2.18 (m, 2H), 1.62 – 1.54 (m, 1H), 1.53 – 1.40 (m, 4H), 1.37 – 1.29 (m, 1H), 1.15 (s, 6H), 1.12 (s, 6H). **^{13}C NMR** (151 MHz, $CDCl_3$) δ 137.4, 131.5 (2C), 128.0 (2C), 121.9, 83.6, 81.6, 69.3, 59.6 (2C), 50.3 (2C), 40.0 (2C), 34.0 (2C), 20.6 (2C), 17.2. **HRMS *(ESI)*** m/z $[M+Na]^+$ calcd. for $C_{20}H_{28}BrNNaO_2^+$: 416.1196; found 416.1194.

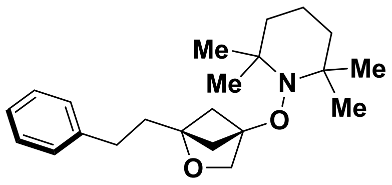

3oa. Obtained from alcohol **1o** and TEMPO **2a** (1.0 F/mol$_{\mathbf{1o}}$). White wax. **FC eluent**: *n*Hex/EtOAc 50:1. **Yield** = 54% (0.054 mmol, 18.9 mg). **^{1}H NMR** (600 MHz, $CDCl_3$) δ 7.30 – 7.26 (m, 2H), 7.22 – 7.16 (m, 3H), 3.79 (s, 2H), 2.73 – 2.67 (m, 2H), 2.06 – 1.99 (m, 2H), 1.92 – 1.82 (m, 4H), 1.62 – 1.53 (m, 1H), 1.51 – 1.39 (m, 4H), 1.36 – 1.30 (m, 1H), 1.12 (s, 6H), 1.09 (s, 6H). **^{13}C NMR** (151 MHz, $CDCl_3$) δ 142.1, 128.4 (2C), 128.3 (2C), 125.8, 83.7, 81.7, 69.0, 59.3 (2C), 47.9 (2C), 39.8 (2C), 33.8 (2C), 33.8, 31.5, 20.4 (2C), 17.1. **HRMS *(ESI)*** m/z $[M+Na]^+$ calcd. for $C_{22}H_{33}NNaO_2^+$: 366.2404; found 366.2407.

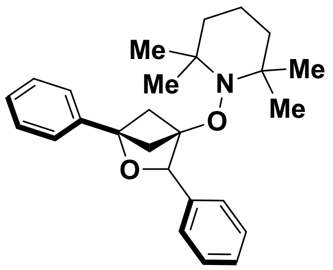

(+/-)-**3pa.** Obtained from alcohol **1p** and TEMPO **2a** (1.0 F/mol$_{\mathbf{1p}}$). White solid. **m.p.** 107 - 109 °C. **FC eluent**: *n*Hex/EtOAc 50:1. **Yield** = 55% (0.055 mmol, 21.5 mg). **^{1}H NMR** (600 MHz, $CDCl_3$) δ 7.59 – 7.55 (m, 2H), 7.54 – 7.51 (m, 2H), 7.41 – 7.37 (m, 2H), 7.37 – 7.34 (m, 2H), 7.34 – 7.30 (m, 1H), 7.30 – 7.27 (m, 1H), 5.10 (s, 1H), 2.72 (d, *J* = 7.0 Hz, 1H), 2.38 (ddd, *J* = 10.2, 7.0, 1.2 Hz, 1H), 2.28 – 2.20 (m, 2H), 1.63 – 1.56 (m, 1H), 1.54 – 1.44 (m, 2H), 1.43 – 1.34 (m, 2H), 1.34 – 1.28 (m, 1H), 1.24 (s, 3H), 1.22 (s, 3H), 0.95 (s, 3H), 0.67 (s, 3H). **^{13}C NMR** (151 MHz, $CDCl_3$) δ 139.9, 138.0, 128.3 (2C), 127.8, 127.5 (2C), 127.1 (2C), 127.1, 126.2 (2C), 85.3, 81.7, 78.7, 59.6, 59.6, 52.6, 45.5, 40.1, 40.0, 34.4, 33.6, 21.0, 20.8, 17.1. **HRMS *(ESI)*** m/z $[M+Na]^+$ calcd. for $C_{26}H_{33}NNaO_2^+$: 414.2404; found 414.2398.

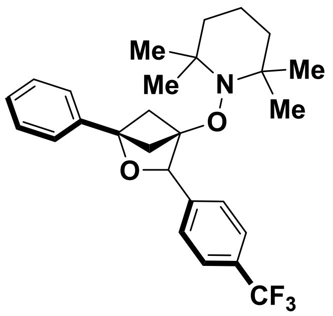

(+/-)-**3qa.** Obtained from alcohol **1q** and TEMPO **2p** (1.0 F/mol$_{\mathbf{1q}}$). White solid. **m.p.** 74 - 76 °C. **FC eluent**: *n*Hex/EtOAc 50:1. **Yield** = 61% (0.061 mmol, 28.0 mg). **^{1}H NMR** (600 MHz, $CDCl_3$) δ 7.72 – 7.68 (m, 2H), 7.64 – 7.60 (m, 2H), 7.53 – 7.48 (m, 2H), 7.43 – 7.37 (m, 2H), 7.36 – 7.32 (m, 1H), 5.13 (s, 1H), 2.75 (d, *J* = 7.1 Hz, 1H), 2.40 (dd, *J* = 10.5, 7.1 Hz, 1H), 2.29 (d, *J* = 7.7 Hz, 1H), 2.13 (dd, *J* = 10.4, 7.7 Hz, 1H), 1.64 – 1.45 (m, 3H), 1.42 – 1.37 (m, 2H), 1.36 – 1.30 (m, 1H), 1.25 (s, 3H), 1.22 (s, 3H), 0.95 (s, 3H), 0.69 (s, 3H). **^{13}C NMR** (151 MHz, $CDCl_3$) δ 144.0, 137.6, 129.3 (q, *J* = 31.9 Hz), 128.3 (2C), 128.0, 127.4 (2C), 126.2 (2C), 124.4 (q, *J* = 3.7 Hz, 2C), 124.4 (q, *J* = 272.2 Hz), 85.4, 82.1, 78.2, 59.7 (2C overlapped), 52.7, 45.5, 40.0, 40.0, 34.3, 33.7, 21.1, 20.9, 17.0. **^{19}F NMR** (565 MHz, $CDCl_3$) δ -62.24 (s, 3F). **HRMS *(ESI)*** m/z $[M+Na]^+$ calcd. for $C_{27}H_{32}F_3NNaO_2^+$: 482.2277; found 482.2275.

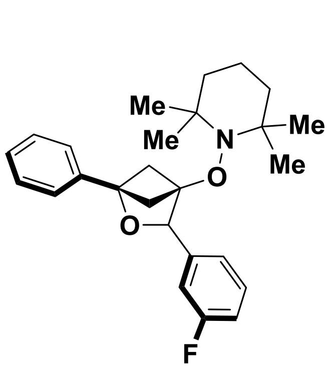

(+/-)-**3ra.** Obtained from alcohol **1r** and TEMPO **2a** (1.0 F/mol$_{\mathbf{1r}}$). White solid. **m.p.** 65 - 67 °C. **FC eluent**: *n*Hex/EtOAc 50:1. **Yield** = 66% (0.066 mmol, 27.0 mg). **^{1}H NMR** (600 MHz, $CDCl_3$) δ 7.53 – 7.49 (m, 2H), 7.42 – 7.38 (m, 2H), 7.37 – 7.29 (m, 4H), 7.00 – 6.96 (m, 1H), 5.08 (s, 1H), 2.73 (d, *J* = 7.1 Hz, 1H), 2.38 (dd, *J* = 10.5, 7.0 Hz, 1H), 2.28 (d, *J* = 7.6 Hz, 1H), 2.18 (dd, *J* = 10.7, 7.5 Hz, 1H), 1.64 – 1.56 (m, 1H), 1.54 – 1.45 (m, 2H), 1.44 – 1.37 (m, 2H), 1.35 – 1.30 (m, 1H), 1.24 (s, 3H), 1.21 (s, 3H), 0.98 (s, 3H), 0.70 (s, 3H). **^{13}C NMR** (151 MHz, $CDCl_3$) δ 161.5 (d,

J = 244.6 Hz), 141.6 (d, *J* = 7.1 Hz), 136.7, 127.9 (d, *J* = 8.0 Hz), 127.3 (2C), 126.9, 125.2 (2C), 121.7 (d, *J* = 2.7 Hz), 113.0 (d, *J* = 22.5 Hz), 112.9 (d, *J* = 21.2 Hz), 84.3, 80.9, 77.1 (d, *J* = 2.1 Hz), 58.6 (d, *J* = 6.3 Hz), 51.6, 44.5, 39.0, 39.0, 33.3, 32.6, 20.0, 19.8, 16.0. **^{19}F NMR** (565 MHz, $CDCl_3$) δ -118.06 – -118.12 (m, 1F). **HRMS *(ESI)*** m/z [M+Na]$^+$ calcd. for $C_{26}H_{32}FNNaO_2^+$: 432.2309; found 432.2313.

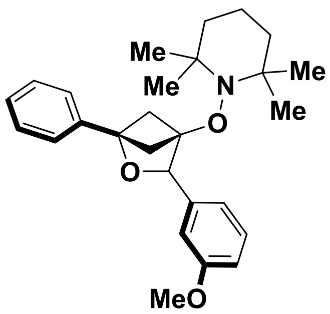

(+/-)-**3sa.** Obtained from alcohol **1s** and TEMPO **2a** (1.0 F/mol$_{\mathbf{1s}}$). White solid. **m.p.** 104 - 106 °C. **FC eluent**: *n*Hex/EtOAc 50:1. **Yield** = 45% (0.045 mmol, 18.9 mg). **^{1}H NMR** (600 MHz, $CDCl_3$) δ 7.53 – 7.50 (m, 2H), 7.40 – 7.37 (m, 2H), 7.33 – 7.30 (m, 1H), 7.28 (t, *J* = 7.8 Hz, 1H), 7.18 (ddt, *J* = 7.6, 1.7, 0.9 Hz, 1H), 7.15 (dt, *J* = 2.3, 1.0 Hz, 1H), 6.83 (ddd, *J* = 7.9, 3.0, 1.0 Hz, 1H), 5.08 (s, 1H), 3.83 (s, 3H), 2.73 (d, *J* = 7.0 Hz, 1H), 2.37 (ddd, *J* = 9.7, 7.0, 1.7 Hz, 1H), 2.29 – 2.21 (m, 2H), 1.64 – 1.55 (m, 1H), 1.54 – 1.47 (m, 2H), 1.42 – 1.37 (m, 2H), 1.32 (ddt, *J* = 13.2, 6.8, 3.3 Hz, 1H), 1.25 (s, 3H), 1.21 (s, 3H), 0.98 (s, 3H), 0.73 (s, 3H). **^{13}C NMR** (151 MHz, $CDCl_3$) δ 159.0, 141.6, 138.0, 128.5, 128.3 (2C), 127.8, 126.2 (2C), 119.6, 112.8, 112.5, 85.3, 81.7, 78.6, 59.6, 59.6, 55.2, 52.5, 45.6, 40.1, 40.0, 34.3, 33.8, 21.0, 20.9, 17.1. **HRMS *(ESI)*** m/z [M+Na]$^+$ calcd. for $C_{27}H_{35}NNaO_3^+$: 444.2509; found 444.2516.

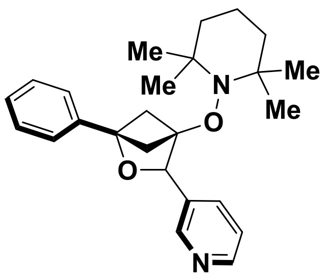

(+/-)-**3ta.** Obtained from alcohol **1t** and TEMPO **2a** (1.0 F/mol$_{\mathbf{1t}}$). White solid. **m.p.** 111 - 113 °C. **FC eluent**: *n*Hex/EtOAc 5:1. **Yield** = 41% (0.041 mmol, 16.1 mg). **^{1}H NMR** (600 MHz, $CDCl_3$) δ 8.83 (d, *J* = 2.2 Hz, 1H), 8.55 (dd, *J* = 4.9, 1.7 Hz, 1H), 7.87 (dt, *J* = 7.9, 2.2 Hz, 1H), 7.52 – 7.48 (m, 2H), 7.41 – 7.38 (m, 2H), 7.35 – 7.32 (m, 1H), 7.30 (dd, *J* = 7.9, 4.8 Hz, 1H), 5.12 (s, 1H), 2.75 (d, *J* = 7.1 Hz, 1H), 2.40 (dd, *J* = 10.4, 7.2 Hz, 1H), 2.30 (d, *J* = 7.8 Hz, 1H), 2.15 (dd, *J* = 10.5, 7.8 Hz, 1H), 1.53 – 1.47 (m, 2H), 1.42 – 1.37 (m, 2H), 1.35 – 1.26 (m, 2H), 1.24 (s, 3H), 1.21 (s, 3H), 0.96 (s, 3H), 0.69 (s, 3H). **^{13}C NMR** (151 MHz, $CDCl_3$) δ 148.8, 148.6, 137.5, 135.3, 134.7, 128.3 (2C), 128.0, 126.2 (2C), 122.6, 85.3, 82.3, 59.7, 59.7, 52.5, 45.5 (2C overlapped), 40.0, 40.0, 34.3, 33.7, 21.0, 20.9, 17.0. **HRMS *(ESI)*** m/z [M+Na]$^+$ calcd. for $C_{25}H_{32}N_2NaO_2^+$: 415.2356; found 415.2350.

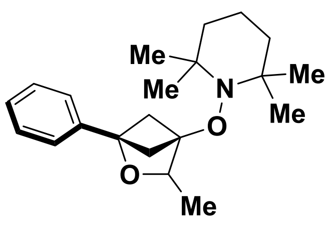

(+/-)-**3ua.** Obtained from alcohol **1u** and TEMPO **2a** (1.0 F/mol$_{1u}$). White solid. **m.p.** 99 - 101 °C. **FC eluent**: *n*Hex/EtOAc 50:1. **Yield** = 49% (0.049 mmol, 16.1 mg). **^{1}H NMR** (600 MHz, $CDCl_3$) δ 7.43 – 7.37 (m, 2H), 7.35 – 7.31 (m, 2H), 7.29 – 7.25 (m, 1H), 4.14 (q, *J* = 6.2 Hz, 1H), 2.52 (d, *J* = 6.6 Hz, 1H), 2.34 (d, *J* = 6.9 Hz, 1H), 2.21 (dd, *J* = 10.4, 6.9 Hz, 1H), 2.16 (dd, *J* = 10.4, 6.7 Hz, 1H), 1.65 – 1.56 (m, 1H), 1.52 – 1.45 (m, 4H), 1.35 (d, *J* = 6.2 Hz, 3H) partially overlapped with 1.34 – 1.30 (m, 1H), 1.14 (app. s, 6H), 1.13 (s, 3H), 1.11 (s, 3H). **^{13}C NMR** (151 MHz, $CDCl_3$) δ 137.1, 127.1 (2C), 126.6, 125.0 (2C), 83.5, 80.5, 74.0, 58.5, 58.4, 51.0, 44.6, 39.0, 38.9, 33.3, 33.1, 19.5, 19.5, 16.2, 16.1. **HRMS *(ESI)*** m/z $[M+Na]^+$ calcd. for $C_{21}H_{31}NNaO_2^+$: 352.2247; found 352.2241.

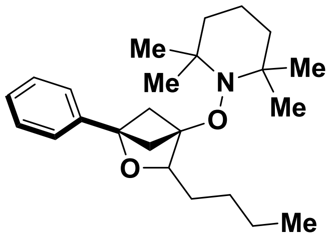

(+/-)-**3va.** Obtained from alcohol **1v** and TEMPO **2a** (1.0 F/mol$_{1v}$). White solid. **m.p.** 66 - 68 °C. **FC eluent**: *n*Hex/EtOAc 50:1. **Yield** = 46% (0.046 mmol, 17.1 mg). **^{1}H NMR** (600 MHz, $CDCl_3$) δ 7.42 – 7.38 (m, 2H), 7.34 – 7.30 (m, 2H), 7.28 – 7.25 (m, 1H), 3.94 (dd, *J* = 8.1, 4.3 Hz, 1H), 2.54 (d, *J* = 6.0 Hz, 1H), 2.32 (d, *J* = 6.3 Hz, 1H), 2.23 – 2.12 (m, 2H), 1.84 – 1.75 (m, 1H), 1.65 – 1.52 (m, 3H), 1.51 – 1.46 (m, 5H), 1.45 – 1.38 (m, 2H), 1.37 – 1.31 (m, 1H), 1.14 (app. s, 6H), 1.13 (s, 3H), 1.11 (s, 3H), 0.94 (t, *J* = 7.3 Hz, 3H). **^{13}C NMR** (151 MHz, $CDCl_3$) δ 137.2, 127.1 (2C), 126.6, 125.1 (2C), 83.8, 80.2, 78.2, 58.5, 58.4, 51.1, 45.3, 39.0, 38.9, 33.4, 33.0, 31.0, 27.3, 22.0, 19.7, 19.6, 16.1, 13.1. **HRMS *(ESI)*** m/z $[M+Na]^+$ calcd. for $C_{24}H_{37}NNaO_2^+$: 394.2717; found 394.2715.

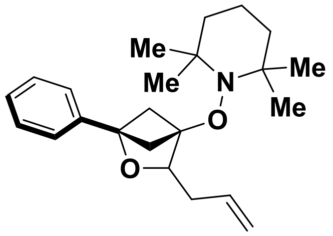

(+/-)-**3wa.** Obtained from alcohol **1w** and TEMPO **2a** (1.0 F/mol$_{1w}$). White solid. **m.p.** 71 - 73 °C. **FC eluent**: *n*Hex/EtOAc 50:1. **Yield** = 66% (0.066 mmol, 23.4 mg). **^{1}H NMR** (600 MHz, $CDCl_3$) δ 7.41 – 7.37 (m, 2H), 7.35 – 7.31 (m, 2H), 7.28 – 7.25 (m, 1H), 6.02 (ddt, *J* = 17.1, 10.2, 6.9 Hz, 1H), 5.19 (dq, *J* = 17.2, 1.6 Hz, 1H), 5.10 (ddt, *J* = 10.2, 2.2, 1.2 Hz, 1H), 4.03 (dd, *J* = 8.3, 4.6 Hz, 1H), 2.60 – 2.54 (m, 2H), 2.40 – 2.33 (m, 2H), 2.22 (dd, *J* = 10.4, 6.8 Hz, 1H), 2.18 (dd, *J* = 10.5, 6.5 Hz, 1H), 1.65 – 1.58 (m, 1H), 1.51 – 1.46 (m, 4H), 1.37 – 1.31 (m, 1H), 1.14 (app. s, 6H), 1.13 (s, 3H), 1.11 (s, 3H). **^{13}C NMR** (151 MHz, $CDCl_3$) δ 138.0, 135.2, 128.2 (2C), 127.7, 126.2 (2C), 116.5, 84.8, 81.6, 78.7, 59.5, 59.5, 52.1, 46.3, 40.1, 40.0, 37.0, 34.4, 34.1, 20.8, 20.7, 17.1. **HRMS *(ESI)*** m/z $[M+Na]^+$ calcd. for $C_{23}H_{33}NNaO_2^+$: 378.2404; found 378.2400.

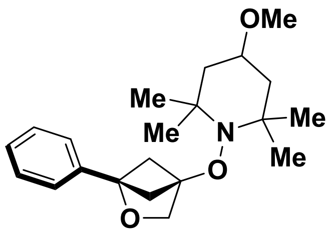

3ab. Obtained from alcohol **1a** and 4-OMeTEMPO **2b** (1.0 F/mol$_{1a}$). White solid. **m.p.** 52 - 54 °C. **FC eluent**: *n*Hex/EtOAc 20:1. **Yield** = 40% (0.040 mmol, 13.8 mg). ^{1}**H NMR** (600 MHz, $CDCl_3$) δ 7.41 – 7.37 (m, 2H), 7.37 – 7.32 (m, 2H), 7.31 – 7.27 (m, 1H), 3.93 (s, 2H), 3.47 (tt, *J* = 11.5, 4.2 Hz, 1H), 3.33 (s, 3H), 2.33 – 2.27 (m, 2H), 2.25 – 2.20 (m, 2H), 1.95 – 1.88 (m, 2H), 1.41 – 1.35 (m, 2H), 1.21 (s, 6H), 1.18 (s, 6H). ^{13}**C NMR** (151 MHz, $CDCl_3$) δ 138.0, 128.2 (2C), 127.8, 126.1 (2C), 83.5, 82.0, 71.6, 69.1, 59.7 (2C), 55.8, 50.0 (2C), 44.7 (2C), 34.1 (2C), 21.3 (2C). **HRMS *(ESI)*** m/z $[M+Na]^+$ calcd. for $C_{21}H_{31}NNaO_3^+$: 368.2196; found 368.2203.

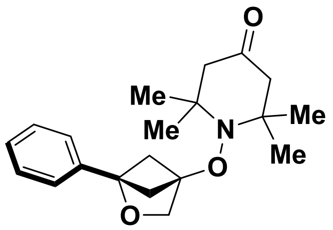

3ac. Obtained from alcohol **1a** and 4-OxoTEMPO **2c** (1.0 F/mol$_{1a}$). White solid. **m.p.** 58 - 60 °C. **FC eluent**: *n*Hex/EtOAc 30:1. **Yield** = 44% (0.044 mmol, 14.5 mg). ^{1}**H NMR** (600 MHz, $CDCl_3$) δ 7.42 – 7.38 (m, 2H), 7.36 (ddd, *J* = 7.9, 6.8, 1.2 Hz, 2H), 7.32 – 7.29 (m, 1H), 3.99 (s, 2H), 2.55 – 2.51 (m, 2H), 2.39 – 2.35 (m, 2H), 2.35 – 2.31 (m, 2H), 2.28 (dd, *J* = 4.4, 1.7 Hz, 2H), 1.31 (s, 6H), 1.22 (s, 6H). ^{13}**C NMR** (151 MHz, $CDCl_3$) δ 207.1, 136.6, 127.3 (2C), 126.9, 125.0 (2C), 82.6, 81.1, 67.9, 61.9 (2C), 51.7 (2C), 49.0 (2C), 31.8 (2C), 22.8 (2C). **HRMS *(ESI)*** m/z $[M+Na]^+$ calcd. for $C_{20}H_{27}NNaO_3^+$: 352.1883; found 352.1885.

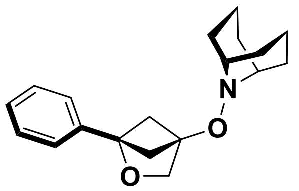

3ad. Obtained from alcohol **1a** and ABNO **2d** (1.0 F/mol$_{1a}$). White solid. **m.p.** 68 - 70 °C. **FC eluent**: *n*Hex/EtOAc 30:1. **Yield** = 51% (0.044 mmol, 15.2 mg). ^{1}**H NMR** (600 MHz, $CDCl_3$) δ 7.43 – 7.39 (m, 2H), 7.37 – 7.33 (m, 2H), 7.31 – 7.27 (m, 1H), 3.81 (s, 2H), 3.19 – 3.13 (m, 2H), 2.33 – 2.22 (m, 4H), 2.16 – 2.10 (m, 2H), 2.10 – 2.02 (m, 2H), 1.97 – 1.82 (m, 2H), 1.81 – 1.74 (m, 2H), 1.63 – 1.58 (m, 1H), 1.48 – 1.44 (m, 1H), 1.36 – 1.30 (m, 2H). ^{13}**C NMR** (151 MHz, $CDCl_3$) δ 138.2, 128.2 (2C), 127.8, 126.1 (2C), 83.7, 80.9, 68.9, 55.3 (2C), 47.1 (2C), 32.1 (4C), 23.0 (2C). **HRMS *(ESI)*** m/z $[M+Na]^+$ calcd. for $C_{19}H_{25}NNaO_2^+$: 322.1777; found 322.1769.

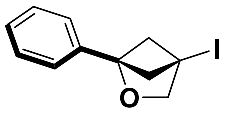

3ae. Obtained from alcohol **1a** and TBAI **2e** (2.0 F/mol$_{\mathbf{1a}}$). Colourless oil. **FC eluent**: *n*Hex/EtOAc 10:1. **Yield** = 48% (0.048 mmol, 13.7 mg). **^{1}H NMR** (600 MHz, $CDCl_3$) δ 7.39 – 7.31 (m, 5H), 4.01 (s, 2H), 2.51 – 2.46 (m, 2H), 2.33 – 2.28 (m, 2H). **^{13}C NMR** (151 MHz, $CDCl_3$) δ 135.9, 128.5 (2C), 128.4, 126.0 (2C), 90.6, 77.4, 53.1 (2C), 18.6. **HRMS** ***(ESI)*** m/z $[M+Na]^+$ calcd. for $C_{11}H_{11}INaO^+$: 308.9747; found 308.9749.

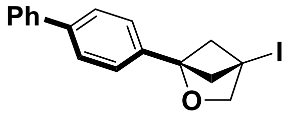

3ie. Obtained from alcohol **1i** and TBAI **2e** (2.0 F/mol$_{\mathbf{1i}}$). Colourless oil. **FC eluent**: *n*Hex/EtOAc 10:1. **Yield** = 44% (0.044 mmol, 15.9 mg). **^{1}H NMR** (600 MHz, $CDCl_3$) δ 7.62 – 7.56 (m, 4H), 7.48 – 7.41 (m, 4H), 7.38 – 7.33 (m, 1H), 4.03 (s, 2H), 2.55 – 2.49 (m, 2H), 2.38 – 2.28 (m, 2H). **^{13}C NMR** (151 MHz, $CDCl_3$) δ 141.4, 140.6, 134.9, 128.8 (2C), 127.5, 127.2 (2C), 127.2 (2C), 126.4 (2C), 90.5, 77.4, 53.2 (2C), 18.6. **HRMS** ***(ESI)*** m/z $[M+Na]^+$ calcd. for $C_{17}H_{15}INaO^+$: 385.0060; found 385.0067.

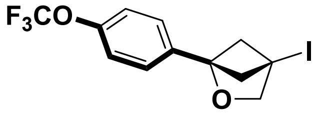

3ke. Obtained from alcohol **1k** and TBAI **2e** (2.0 F/mol$_{\mathbf{1k}}$). Colourless oil. **FC eluent**: *n*Hex/EtOAc 10:1. **Yield** = 51% (0.051 mmol, 18.8 mg). **^{1}H NMR** (600 MHz, $CDCl_3$) δ 7.43 – 7.39 (m, 2H), 7.23 – 7.20 (m, 2H), 4.01 (s, 2H), 2.51 – 2.45 (m, 2H), 2.31 – 2.26 (m, 2H). **^{13}C NMR** (151 MHz, $CDCl_3$) δ 148.1, 133.6, 126.5 (2C), 119.9 (2C), 119.4 (d, J = 257.6 Hz), 88.8, 76.4, 52.1 (2C), 17.0. **^{19}F NMR** (565 MHz, $CDCl_3$) δ -57.9 (s, 3F). **HRMS** ***(ESI)*** m/z $[M+Na]^+$ calcd. for $C_{12}H_{10}F_3INaO_2^+$: 392.9570; found 392.9573.

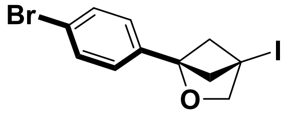

3ne. Obtained from alcohol **1n** and TBAI **2e** (2.0 F/mol$_{\mathbf{1n}}$). Colourless oil. **FC eluent**: *n*Hex/EtOAc 10:1. **Yield** = 41% (0.041 mmol, 14.9 mg). **^{1}H NMR** (600 MHz, $CDCl_3$) δ 7.51 – 7.48 (m, 2H), 7.26 – 7.23 (m, 2H), 4.00 (s, 2H), 2.48 – 2.43 (m, 2H), 2.30 – 2.25 (m, 2H). **^{13}C NMR** (151 MHz, $CDCl_3$) δ 135.0, 131.6 (2C), 127.7 (2C), 122.4, 90.0, 77.4, 53.1 (2C), 18.1. **HRMS** ***(ESI)*** m/z $[M+Na]^+$ calcd. for $C_{11}H_{10}BrINaO^+$: 386.8852; found 386.8851.

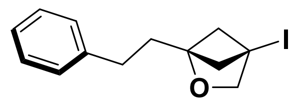

3oe. Obtained from alcohol **1o** and TBAI **2e** (2.0 F/mol$_{\mathbf{1o}}$). Colourless oil. **FC eluent**: *n*Hex/EtOAc 10:1. **Yield** = 44% (0.044 mmol, 13.8 mg). **^{1}H NMR** (600 MHz, $CDCl_3$) δ 7.30 – 7.26 (m, 2H), 7.21 – 7.17 (m, 3H), 3.84 (s, 2H), 2.75 – 2.70 (m, 2H), 2.17 – 2.11 (m, 2H), 2.04 (dd, *J* = 4.72, 1.81 Hz, 2H), 1.92 (dd, *J* = 4.70, 1.82 Hz, 2H). **^{13}C NMR** (151 MHz, $CDCl_3$) δ 141.5, 128.6 (2C), 128.5 (2C), 126.2, 90.6, 77.3 (partially overlapped with residual solvent signal), 51.7 (2C), 33.3, 31.2, 19.2. **HRMS *(ESI)*** m/z $[M+Na]^+$ calcd. for $C_{13}H_{15}INaO^+$: 337.0060; found 337.0053.

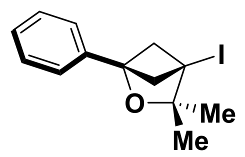

3xe. Obtained from alcohol **1x** and TBAI **2e** (2.0 F/mol$_{\mathbf{1x}}$). Colourless oil. **FC eluent**: *n*Hex/EtOAc 10:1. **Yield** = 55% (0.055 mmol, 17.3 mg). **^{1}H NMR** (600 MHz, $CDCl_3$) δ 7.37 – 7.32 (m, 4H), 7.32 – 7.29 (m, 1H), 2.68 (dd, *J* = 4.83, 1.74 Hz, 2H), 2.39 (dd, *J* = 4.84, 1.76 Hz, 2H), 1.50 (s, 6H). **^{13}C NMR** (151 MHz, $CDCl_3$) δ 137.2, 128.6 (2C), 128.5 (2C), 126.0, 89.9, 82.9, 52.6 (2C), 32.1, 25.1 (2C). **^{1}H NMR** (600 MHz, $CDCl_3$) δ. **^{13}C NMR** (151 MHz, $CDCl_3$) δ. **HRMS *(ESI)*** m/z $[M+Na]^+$ calcd. for $C_{13}H_{15}INaO^+$: 337.0060; found 337.0055.

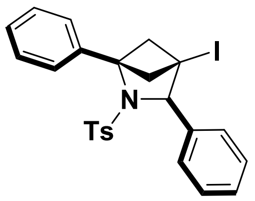

(+/-)-**3ye.** Obtained from amide **1y** and TBAI **2e** (2.0 F/mol$_{\mathbf{1y}}$). Colourless oil. **FC eluent**: *n*Hex/EtOAc 6:1. **Yield** = 42% (0.042 mmol, 21.6 mg). **^{1}H NMR** (600 MHz, $CDCl_3$) δ 7.74 – 7.70 (m, 2H), 7.49 – 7.44 (m, 2H), 7.43 – 7.40 (m, 1H), 7.24 – 7.19 (m, 1H), 7.15 – 7.06 (m, 6H), 6.98 – 6.94 (m, 2H), 5.41 (s, 1H), 3.03 (dd, *J* = 10.2, 7.5 Hz, 1H), 2.91 (dd, *J* = 10.3, 7.9 Hz, 1H), 2.76 (d, *J* = 7.4 Hz, 1H), 2.33 (s, 3H), 2.12 (dd, *J* = 7.9, 1.3 Hz, 1H). **^{13}C NMR** (151 MHz, $CDCl_3$) δ 143.0, 137.5, 137.0, 134.0, 128.9 (2C), 128.9 (2C), 128.4, 128.3, 128.0 (2C), 127.9 (2C), 127.8 (2C + 2C overlapped), 76.7, 70.4, 53.4, 50.3, 25.6, 21.5. **HRMS *(ESI)*** m/z $[M+Na]^+$ calcd. for $C_{24}H_{22}INNaO_2S^+$:538.0308; found 538.0314.

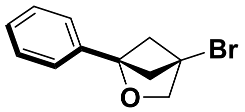

3af. Obtained from alcohol **1a** and TBABr **2f** (3.5 F/mol$_{\mathbf{1a}}$). Colourless oil. **FC eluent**: *n*Hex/EtOAc 10:1. **Yield** = 46% (0.046 mmol, 10.9 mg). **^{1}H NMR** (600 MHz, $CDCl_3$) δ ^{1}H NMR (600 MHz, $CDCl_3$) δ 7.45 – 7.30 (m, 5H), 3.99 (s, 2H), 2.48 – 2.41 (m, 2H), 2.38 – 2.31 (m, 2H). **^{13}C NMR** (151 MHz, $CDCl_3$) δ 136.2, 128.5 (2C), 128.5, 126.0 (2C), 87.6, 74.4, 51.9 (2C), 47.1. **HRMS *(ESI)*** m/z $[M+Na]^+$ calcd. for $C_{11}H_{11}BrNaO^+$: 260.9885; found 260.9888.

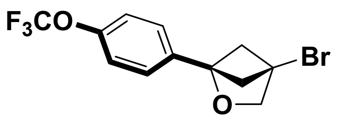

3kf. Obtained from alcohol **1k** and TBABr **2f** (3.5 F/mol$_{\mathbf{1k}}$). Colourless oil. **FC eluent**: *n*Hex/EtOAc 10:1. **Yield** = 42% (0.042 mmol, 13.5 mg). **^{1}H NMR** (600 MHz, $CDCl_3$) δ 7.43 – 7.39 (m, 2H), 7.24 – 7.20 (m, 2H), 3.98 (s, 2H), 2.47 – 2.42 (m, 2H), 2.36 – 2.30 (m, 2H). **^{13}C NMR** (151 MHz, $CDCl_3$) δ 134.9, 128.5, 127.6 (2C), 121.0 (2C), 119.6 (d, *J* = 258.2 Hz), 86.9, 74.3, 52.0, 46.7. **^{19}F NMR** (565 MHz, $CDCl_3$) δ -57.9 (s, 3F). **HRMS *(ESI)*** m/z $[M+Na]^+$ calcd. for $C_{12}H_{10}BrF_3NaO_2^+$: 344.9708; found 344.9699.

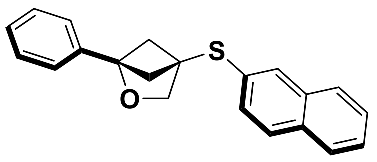

3ag. Obtained from alcohol **1a** and naphthalene-2-thiol **2g** (2.0 F/mol$_{\mathbf{1a}}$) in the presence of 2,6-lutidine (2.0 equiv). Colourless oil. **FC eluent**: *n*Hex/EtOAc 20:1. **Yield** = 44% (0.044 mmol, 14.0 mg). **^{1}H NMR** (600 MHz, $CDCl_3$) δ 8.06 (s, 1H), 7.87 – 7.80 (m, 3H), 7.58 (dd, *J* = 8.5, 1.8 Hz, 1H), 7.54 – 7.50 (m, 2H), 7.35 – 7.27 (m, 5H), 3.90 (s, 2H), 2.17 – 2.11 (m, 4H). **^{13}C NMR** (151 MHz, $CDCl_3$) δ 137.1, 134.3, 133.5, 132.9, 131.7, 128.8, 128.6, 128.3 (2C), 128.2, 127.7, 127.7, 126.8, 126.7, 126.0 (2C), 87.5, 72.9, 52.3, 49.1 (2C). **HRMS *(ESI)*** m/z $[M+Na]^+$ calcd. for $C_{21}H_{18}NaOS^+$: 341.0971; found 341.0975.

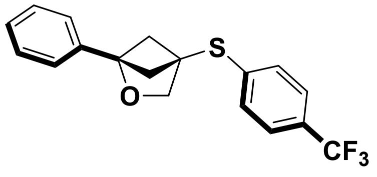

3ah. Obtained from alcohol **1a** and 4-trifluomethylthiophenol **2h** (2.0 F/mol$_{\mathbf{1a}}$) in the presence of 2,6-lutidine (2.0 equiv). Colourless oil. **FC eluent**: *n*Hex/EtOAc 20:1. **Yield** = 52% (0.052 mmol, 17.5 mg). **^{1}H NMR** (600 MHz, $CDCl_3$) δ 7.64 – 7.61 (m, 2H), 7.60 – 7.58 (m, 2H), 7.37 – 7.29 (m, 5H), 3.89 (s, 2H), 2.16 (*pseudo*-s, 4H). **^{13}C NMR** (151 MHz, $CDCl_3$) δ 136.9, 136.8, 134.0 (2C), 130.3 (q, *J* = 32.7 Hz), 128.4 (2C), 128.3, 125.9 (q, *J* = 3.7 Hz, 2C), 126.0 (2C), 123.90 (q, *J* = 272.4 Hz), 87.6, 72.6, 51.5, 49.1

(2C). **^{19}F NMR** (565 MHz, $CDCl_3$) δ -62.7 (s, 3F). **HRMS *(ESI)*** m/z $[M+Na]^+$ calcd. for $C_{18}H_{15}F_3NaOS^+$: 359.0688; found 359.0689.

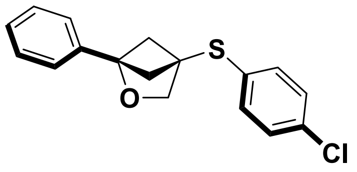

3ai. Obtained from alcohol **1a** and 4-chlorothiophenol **2i** (2.0 F/mol$_{1a}$) in the presence of 2,6-lutidine (2.0 equiv). Colourless oil. **FC eluent**: *n*Hex/EtOAc 20:1. **Yield** = 50% (0.050 mmol, 15.1 mg). **^{1}H NMR** (600 MHz, $CDCl_3$) δ 7.50 – 7.44 (m, 2H), 7.37 – 7.27 (m, 7H), 3.84 (s, 2H), 2.10 – 2.06 (m, 4H). **^{13}C NMR** (151 MHz, $CDCl_3$) δ 136.9, 136.2 (2C), 135.0, 129.9, 129.3 (2C), 128.3 (2C), 128.2, 126.0 (2C), 87.4, 72.7, 52.1, 48.9 (2C). **HRMS *(ESI)*** m/z [M+Na]$^+$ calcd. for $C_{17}H_{15}ClNaOS^+$: 325.0424; found 325.0420.

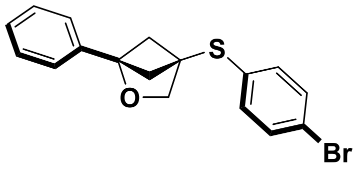

3aj. Obtained from alcohol **1a** and 4-bromothiophenol **2j** (2.0 F/mol$_{1a}$) in the presence of 2,6-lutidine (2.0 equiv). Colourless oil. **FC eluent**: *n*Hex/EtOAc 20:1. **Yield** = 56% (0.056 mmol, 19.4 mg). **^{1}H NMR** (600 MHz, $CDCl_3$) δ 7.50 – 7.45 (m, 2H), 7.43 – 7.37 (m, 2H), 7.35 – 7.32 (m, 4H), 7.31 – 7.28 (m, 1H), 3.85 (s, 2H), 2.11 – 2.07 (m, 4H). **^{13}C NMR** (151 MHz, $CDCl_3$) δ 136.9, 136.4 (2C), 132.2 (2C), 130.6, 128.3 (2C), 128.2, 126.0 (2C), 123.2, 87.5, 72.7, 52.0, 48.9 (2C). **HRMS *(ESI)*** m/z [M+Na]$^+$ calcd. for $C_{17}H_{15}BrNaOS^+$: 368.9919; found 368.9917.

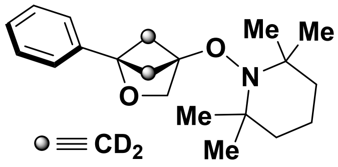

d$_4$*-3aa.** Obtained from alcohol ***d$_4$*-1a** and TEMPO **2a** (1.0 F/mol$_{d4\text{-}1a}$). White solid. **m.p.** 101 -103 °C. **FC eluent**: *n*Hex/EtOAc 50:1. **Yield** = 65% (0.065 mmol, 20.7 mg). **^{1}H NMR** (600 MHz, $CDCl_3$) δ 7.41 – 7.37 (m, 2H), 7.36 – 7.32 (m, 2H), 7.31 – 7.27 (m, 1H), 3.94 (s, 2H), 1.64 – 1.55 (m, 1H), 1.54 – 1.42 (m, 4H), 1.37 – 1.32 (m, 1H), 1.17 (s, 6H), 1.13 (s, 6H). **^{13}C NMR** (151 MHz, $CDCl_3$) δ 137.1, 127.2 (2C), 126.7, 125.0 (2C), 82.1, 80.6, 68.0, 58.3 (2C), 49.4 – 47.3 (m, 2C, CD_2), 38.8 (2C), 32.8 (2C), 19.4 (2C), 16.1. **HRMS *(ESI) m/z [M+Na]$^+$ calcd. for $C_{20}H_{25}D_4NNaO_2^+$: 342.2342; found 342.2345.

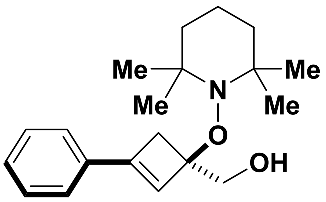

(+/-)-**4aa.** Obtained from alcohol **1a** and TEMPO **2a** in the reaction run under the conditions reported in Table 1, entry 6. **FC eluent**: *n*Hex/EtOAc 3:1. **Yield** = 24% (0.024 mmol, 7.6 mg). **^{1}H NMR** (600 MHz, $CDCl_3$) δ 7.41 – 7.38 (m, 2H), 7.35 (ddd, *J* = 7.8, 6.8, 1.1 Hz, 2H), 7.32 – 7.27 (m, 1H), 6.52 (t, *J* = 1.3 Hz, 1H), 4.60 (s, 1H), 4.03 (s, 2H), 3.14 (dd, *J* = 12.4, 1.4 Hz, 1H), 2.85 (dd, *J* = 12.3, 1.2 Hz, 1H), 1.64 – 1.53 (m, 3H), 1.52 – 1.44 (m, 2H), 1.38 – 1.33 (m, 1H), 1.30 (s, 3H), 1.28 (s, 3H), 1.15 (s, 3H), 1.14 (s, 3H). **^{13}C NMR** (151 MHz, $CDCl_3$) δ 146.1, 133.8, 130.3, 128.5, 128.4 (2C), 125.4 (2C), 81.7, 69.7, 60.2, 59.8, 40.7, 40.3, 40.2, 34.5, 33.8, 20.7 (2C overlapped), 17.1. **HRMS *(ESI)*** m/z $[M+Na]^+$ calcd. for $C_{20}H_{29}NNaO_2^+$: 338.2090; found 338.2096.

3.3 Preparation of **3aa** on 1.0 mmol scale.

The ElectraSyn vial (10 mL), equipped with a stir bar, was charged with TEMPO **2a** (2.0 mmol, 302 mg, 2.0 equiv) and $LiClO_4$ (3.0 mmol, 319 mg). The ElectraSyn vial cap, equipped with anode (C) and cathode (Ni), was inserted into the mixture and closed with a rubber septum. Then THF (6.0 mL) and a solution of alcohol **1** in ACN (1.0 mmol, 3.0 mL, 0.33 M) were added, the mixture stirred until complete dissolution of the solids occurred and then cooled to -30 °C. The reaction mixture was electrolysed at a constant current of 10.0 mA at -30 °C, until a total charge of 1.0 mF (1.0 F/$mol_{\mathbf{1a}}$) was reached. Then, the ElectraSyn vial cap was removed, and the electrodes and vial were rinsed with EtOAc (5 mL) and $NH_4Cl_{(aq)}$ (saturated, 15 mL) and water (15 mL), which were combined with the crude mixture in a separatory funnel. Then, the organic layer was separated, and the aqueous layer was extracted with EtOAc (2 x 15 mL). The combined organic layers were washed with $NH_4Cl_{(aq)}$ (0.1 M, 3 x 15 mL), dried over Na_2SO_4 and concentrated in vacuo. The crude product was finally purified by FC on silica gel (*n*Hex/EtOAc 50:1) to afford pure products **3aa** in 70% yield (0.70 mmol, 217 mg).

3.4 Unproductive experiments

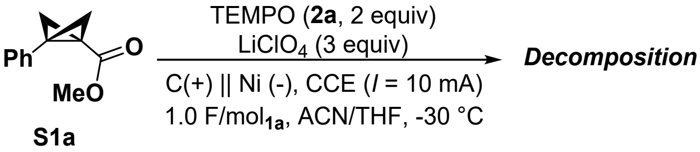

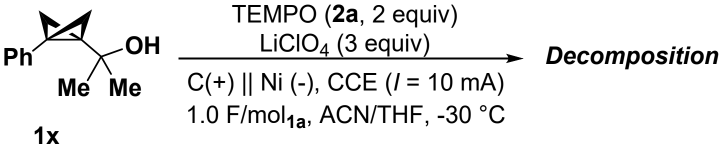

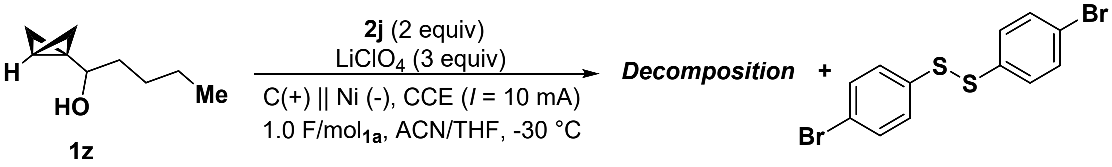

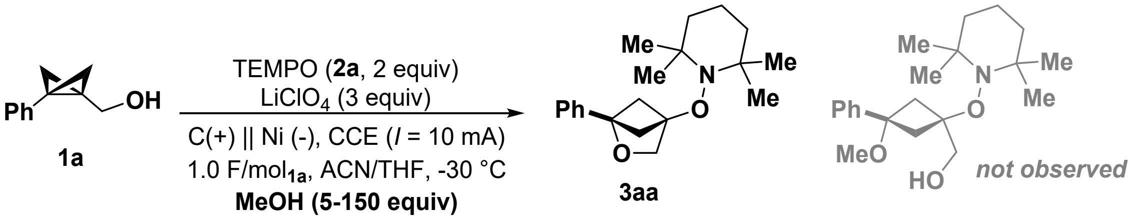

Ester derivatives, such as **S1a**, were found to be unproductive for the desired transformation. Most likely, their reduced nucleophilicity prevented their capture by $TEMPO^+$ cation.

Tertiary alcohol **1x** did not undergo the desired functionalization with TEMPO **2a**, probably due to excessive steric encumbrance, while the desired reaction occurred smoothly with TBAI **2e**.

The reaction with Cγ-unsubstituted BCB **1z** with thiol **2j**, potentially leading to a regiochemical switch in the formation of the radical and cationic intermediates, did not lead to any detectable product. On the other hand, thiol dimerization occurred.

The trap of the putative carbocationic intermediate formed in the reaction between **1a** and **2a** with external nucleophiles such as MeOH was not successful. Computational studies provided an explanation in the very low energy barrier associated with the ring-closure event (see main text).

4. Product Elaborations

4.1 Preparation of Alcohol **6**

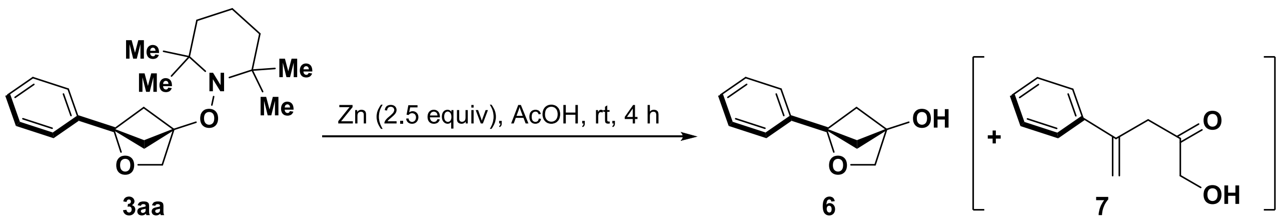

3aa **6** **7**

In a Schlenk flask, equipped with a magnetic stirring bar and under Ar atmosphere, compound **3aa** (0.50 mmol, 158 mg) was dissolved in glacial AcOH (5.0 mL) and Zn dust (1.25 mmol, 2.5 equiv, 83 mg) was added and the resulting suspension was vigorously stirred at room temperature for 4 h. Then, a saturated solution of $NaHCO_3$ (25 mL) was carefully added, followed by EtOAc (10 mL). Then, the biphasic mixture was moved to a separatory funnel, the organic layer was separated, and the aqueous layer was extracted with EtOAc (2 x 10 mL). The combined organic layers were washed with $NaHCO_3$ (saturated, 3 x 10 mL), dried over Na_2SO_4 and concentrated in vacuo. The crude product was finally purified by FC on silica gel (*n*Hex/EtOAc 5:1) to afford pure product **6** as a white waxy solid (50.2 mg, 0.29 mmol, 57% yield) as the third eluting fraction. By-product **7** (12.8 mg, 0.07 mmol, 14% yield, colourless oil) was isolated as the second eluting fraction and recovered starting material **3aa** (31.6 mg, 0.10 mmol, 20% yield, white solid) as the first one.

*Note: The use of higher equivalents of Zn dust (5 equiv) or prolonged reaction times (10 h) ensured full conversion of **3aa** but worsened the **6/7** ratio. Running the reaction at temperatures higher than 45 °C led to a substantial decomposition of **3aa**. Running the reaction in a THF/AcOH mixture, as frequently reported in the literature,[13] afforded no conversion of **3aa** at room temperature and only decomposition products at temperatures higher than 50 °C, irrespective of the amount of Zn dust used (2.5 – 10 equiv).*

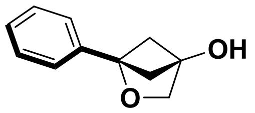

6. **^{1}H NMR** (600 MHz, $CDCl_3$) δ 7.40 – 7.34 (m, 4H), 7.33 – 7.29 (m, 1H), 3.79 (s, 2H), 2.57 (bs, 1H), 2.31 – 2.24 (m, 2H), 2.16 – 2.09 (m, 2H). **^{13}C NMR** (151 MHz, $CDCl_3$) δ 137.5, 128.3 (2C), 128.1, 126.1 (2C), 83.1, 75.2, 70.2, 49.7 (2C). **HRMS *(ESI)*** m/z $[M+Na]^+$ calcd. for $C_{11}H_{12}NaO_2^+$: 199.0730; found 199.0734.

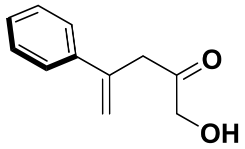

7. **^{1}H NMR** (600 MHz, $CDCl_3$) δ 7.40 – 7.37 (m, 2H), 7.36 – 7.33 (m, 2H), 7.32 – 7.28 (m, 1H), 5.62 (d, *J* = 0.7 Hz, 1H), 5.26 (q, *J* = 1.0 Hz, 1H), 4.27 (s, 2H), 3.65 (s, 2H). **^{13}C NMR** (151 MHz, $CDCl_3$) δ 207.5, 140.0, 139.1, 128.7 (2C), 128.3, 125.8 (2C), 117.5, 67.7, 46.1. **HRMS *(ESI)*** m/z $[M+Na]^+$ calcd. for $C_{11}H_{12}NaO_2^+$: 199.0730; found 199.0736.

4.2 Telescopic 7-step procedure for the preparation of **6** from keto-acid **12**.

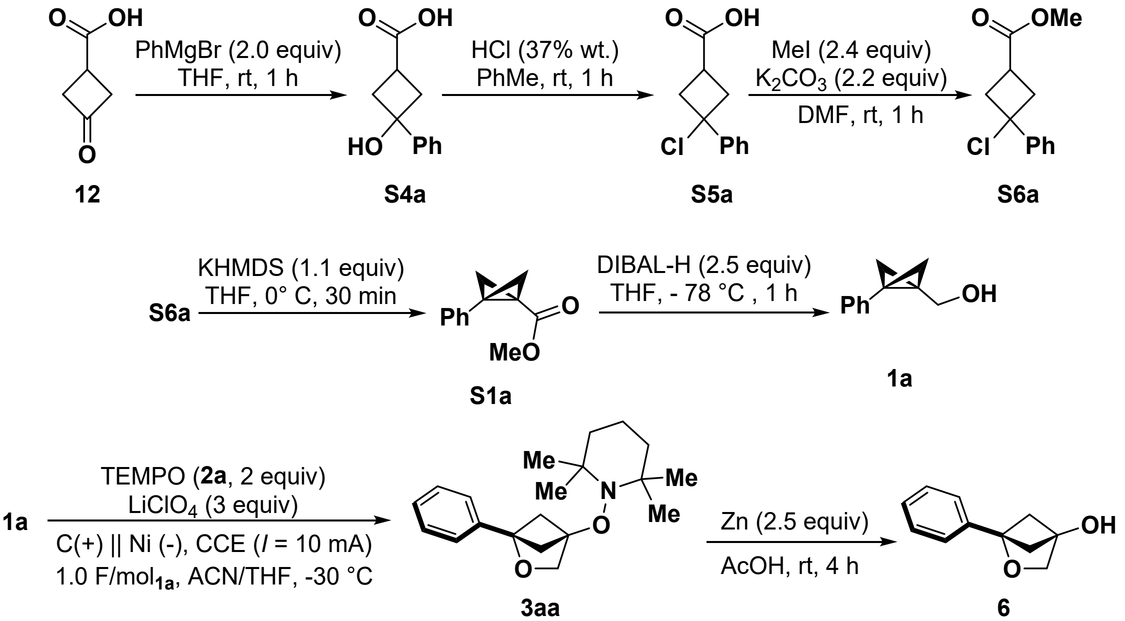

Step 1. In a heat-gun dried 3-necked round bottom flask, equipped with a magnetic stirring bar and under Ar atmosphere, 3-oxocyclobutane carboxylic acid **12** (10 mmol) was dissolved in anhydrous THF (20 mL) and a solution of phenylmagnesium bromide (20 mmol, 2 equiv, 0.7 M, 30 mL) was added dropwise (a slow addition is usually preferred in order to minimize the formation of by-products) at room temperature (a mildly exothermic process occurs) through a dropping funnel. After the addition was completed, the resulting solution was stirred for 30 min, and the conversion was checked by ^{1}H NMR spectroscopy that confirmed complete consumption of the starting material. Then, H_2O (5 mL) was carefully added, followed by HCl (aqueous, 2 M, 20 mL or until a strongly acidic pH was reached) and EtOAc (30 mL). The resulting biphasic mixture was transferred to a separatory funnel. The phases were separated, the aqueous layer was extracted again with EtOAc (2 x 30 mL), the combined organic phases were dried with Na_2SO_4, evaporated in vacuo and the resulting crude alcohol **S4a** (pale-yellow solid) was used in the next step without any purification.[1]

Step 2. In a round-bottom flask equipped with a magnetic stirring bar, **S4a** (10 mmol, from the previous step) was suspended in toluene (20 mL) and HCl (aqueous, 37% w/w, 20 mL) was added. The resulting suspension was vigorously stirred for 1 h and the conversion was checked by ^{1}H NMR spectroscopy, that confirmed complete consumption of the starting material. The biphasic mixture was transferred to a separatory funnel, and the phases were separated. The aqueous layer was diluted with H_2O (60 mL) and extracted again with EtOAc (2 x 30 mL), the combined organic phases were dried with Na_2SO_4, evaporated in vacuo

and the resulting crude chloride **S5a** (pale-yellow solid) was used in the next step without purification. [1]

Step 3. In a round-bottom flask equipped with a magnetic stirring bar, **S5a** (10 mmol, from the previous step) was dissolved in DMF (15 mL), K_2CO_3 (22 mmol, 2.2 equiv) was added under vigorous stirring, followed by MeI (24 mmol, 2.4 equiv, dropwise addition). The resulting suspension was stirred for 1 h and the conversion was checked by TLC (*n*-hexane/EtOAc 7:3 + 1% HCOOH) that usually confirmed complete consumption of the starting material. A saturated aqueous solution of NH_4Cl (10 mL), H_2O (10 mL) and EtOAc (20 mL) were added, and the resulting biphasic mixture was transferred to a separatory funnel. The phases were separated, the aqueous layer was extracted again with EtOAc (2 x 10 mL), the combined organic phases were washed with a 0.1 M aqueous solution of NH_4Cl (3 x 20 mL), dried with Na_2SO_4, evaporated in vacuo and the resulting crude **S6a** (pale-yellow oil) was used in the next step without purification. [1]

Step 4. In a heat-gun dried 3-necked round bottom flask equipped with a magnetic stirring bar and under Ar atmosphere crude **S6a** (10 mmol, from previous step) was dissolved in anhydrous THF (15 mL) and cooled to 0 °C. A solution of KHMDS (11 mmol, 1.1 equiv, 1.0 M in THF) was added dropwise and the resulting red-colored solution was stirred at 0 °C until ^{1}H NMR spectroscopy confirmed complete consumption of the starting material (30 min). A saturated aqueous solution of NH_4Cl (20 mL), H_2O (20 mL) and EtOAc (20 mL) were added, and the resulting biphasic mixture was transferred to a separatory funnel. The phases were separated, the aqueous layer was extracted again with EtOAc (2 x 15 mL), the combined organic phases were dried with Na_2SO_4. Trituration with cold *n*Hex/Et_2O (3:1, 3 x 30 mL) afforded product **S1a** in 47% yield over 4 steps (4.7 mmol, 884 mg, 83% average yield per step).[1]

Step 5. In a heat-gun dried 3-necked round bottom flask, equipped with a magnetic stirring bar and under Ar atmosphere, ester **S1a** (1.0 mmol, from previous step) was dissolved in anhydrous THF (3 mL), cooled to – 78 °C and a solution of DIBAL-H (2.5 equiv, 2.5 mmol, 2.5 mL, 1.0 M in toluene) was added dropwise. After the addition was completed, the resulting solution was stirred for 1 h, during which time the temperature was allowed to rise to – 20 °C. Then, EtOAc (10 mL) was carefully added, followed by a saturated solution of Rochelle's salt (10 mL) The biphasic mixture was then vigorously stirred at room temperature for 1 h, until two clear phases were obtained and the transferred to a separatory funnel. The phases were separated, the aqueous phase was extracted again with EtOAc (2

x 10 mL), the combined organic phases were washed with a saturated solution of Rochelle's salt (2 x 10 mL), dried over Na_2SO_4 and concentrated *in vacuo* to obtain alcohol **1a** (152 mg, 0.95 mmol, 95% yield) that was directly used in the further step without purification.

Step 6. The ElectraSyn vial (10 mL), equipped with a stir bar, was charged with TEMPO **2a** (1.9 mmol, 287 mg, 2.0 equiv) and $LiClO_4$ (3.0 mmol, 319 mg). The ElectraSyn vial cap, equipped with anode (C) and cathode (Ni), was inserted into the mixture and closed with a rubber septum. Then THF (6.0 mL) and a solution of alcohol **1a** in CAN (from previous step, 0.95 mmol, 3.0 mL, 0.32 M) were added, the mixture stirred until complete dissolution of the solids occurred and then cooled to -30 °C. The reaction mixture was electrolysed at a constant current of 10.0 mA at -30 °C, until a total charge of 1.0 mF ($F/mol_{\mathbf{1a}}$) was reached. Then, the ElectraSyn vial cap was removed, and the electrodes and vial were rinsed with EtOAc (5 mL) and $NH_4Cl_{(aq)}$ (saturated, 15 mL) and water (15 mL), which were combined with the crude mixture in a separatory funnel. Then, the organic layer was separated, and the aqueous layer was extracted with EtOAc (2 x 15 mL). The combined organic layers were washed with $NH_4Cl_{(aq)}$ (0.1 M, 3 x 15 mL), dried over Na_2SO_4 and concentrated in vacuo. The crude product was finally purified by FC on silica gel (*n*Hex/EtOAc 50:1) to afford crude product **3aa** that was used directly in the next step without further purification.

Step 7. In a Schlenk flask, equipped with a magnetic stirring bar and under Ar atmosphere, compound **3aa** (0.95 mmol, from previous step) was dissolved in glacial AcOH (10.0 mL) and Zn dust (2.5 mmol, 2.5 equiv, 166 mg) was added and the resulting suspension was vigorously stirred at room temperature for 7 h. Then, a saturated solution of $NaHCO_3$ (50 mL) was carefully added, followed by EtOAc (20 mL). Then, the biphasic mixture was moved to a separatory funnel, the organic layer was separated, and the aqueous layer was extracted with EtOAc (2 x 20 mL). The combined organic layers were washed with $NaHCO_3$ (saturated, 3 x 20 mL), dried over Na_2SO_4 and concentrated in vacuo. The crude product was finally purified by FC on silica gel (*n*Hex/EtOAc 5:1) to afford pure product **6** as a white waxy solid in 34% yield over 2 steps (56.8 mg, 0.32 mmol).

4.3 Preparation of Triflate **8**

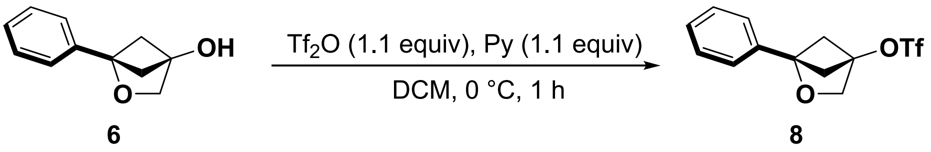

In a Schlenk flask, equipped with a magnetic stirring bar and under Ar atmosphere, compound **6** (0.10 mmol, 17.6 mg) was dissolved in dry DCM (0.5 mL), pyridine (0.11 mmol, 1.1 equiv, 8.9 μL) was added and the solution was cooled to 0 °C. Then, triflic anhydride (0.11 mmol, 1.1 equiv, 18.5 μL) was added and the resulting mixture was stirred at 0 °C for 1 h. A saturated solution of $NaHCO_3$ (3 mL) was carefully added, followed by DCM (3 mL). The biphasic mixture was moved to a separatory funnel, the organic layer was separated, and the aqueous layer was extracted with DCM (2 x 3 mL). The combined organic layers were washed with $NaHCO_3$ (saturated, 3 x 2 mL), dried over Na_2SO_4 and concentrated in vacuo. Pure product **8** was directly obtained as a colourless oil (27.7 mg, 0.090 mmol, 90% yield) without further purification.

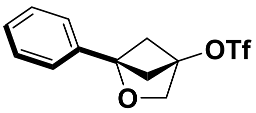

8. ^{1}**H NMR** (600 MHz, $CDCl_3$) δ 7.42 – 7.34 (m, 5H), 4.05 (s, 2H), 2.70 – 2.64 (m, 2H), 2.59 – 2.53 (m, 2H). ^{13}**C NMR** (151 MHz, $CDCl_3$) δ 134.2, 127.8 (2C), 127.5, 125.0 (2C), 117.2 (d, J = 319.6 Hz), 82.1, 81.7, 67.1, 47.9 (2C). ^{19}**F NMR** (565 MHz, $CDCl_3$) δ -75.0 (s, 3F). **HRMS *(ESI)*** m/z $[M+Na]^+$ calcd. for $C_{12}H_{11}F_3NaO_4S^+$: 331.0222; found 331.0219.

4.4 Preparation of Propargyl ether **9**

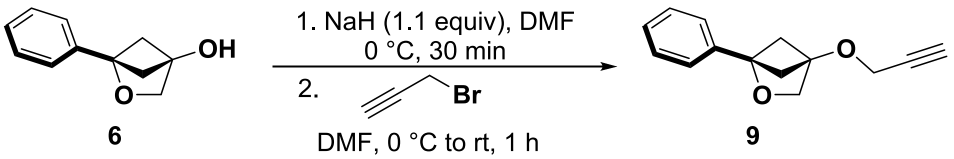

In a Schlenk flask, equipped with a magnetic stirring bar and under Ar atmosphere, compound **6** (0.10 mmol, 17.6 mg) was dissolved in dry DMF (0.5 mL) and the solution was cooled to 0 °C. Then, NaH (0.11 mmol, 1.1 equiv, 3.9 mg, 65% wt. in mineral oil) was added and the resulting mixture was stirred at 0 °C for 30 min. Then, propargyl bromide (0.11 mmol, 1.1 equiv, 8.3 μL) was added and the reaction mixture was stirred at room temperature for 1 h. A saturated solution of NH_4Cl (3 mL) was carefully added, followed by EtOAc (3 mL). The biphasic mixture was moved to a separatory funnel, the organic layer was separated, and the aqueous layer was extracted with EtOAc (2 x 3 mL). The combined organic layers were washed with NaH_4Cl (0.1 M, 3 x 2 mL), dried over Na_2SO_4 and concentrated in vacuo. The crude product was finally purified by FC on silica gel (*n*Hex/EtOAc 20:1) to afford pure product **9** as a colourless oil (15.8 mg, 0.074 mmol, 74% yield)

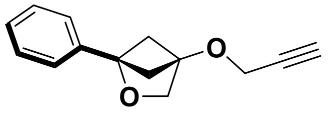

9. ^{1}**H NMR** (600 MHz, $CDCl_3$) δ 7.45 – 7.28 (m, 5H), 4.33 (d, *J* = 2.4 Hz, 2H), 3.88 (s, 2H), 2.49 (t, *J* = 2.4 Hz, 1H), 2.36 – 2.29 (m, 2H), 2.26 – 2.21 (m, 2H). ^{13}**C NMR** (151 MHz, $CDCl_3$) δ 136.4, 127.3 (2C), 127.1, 125.0 (2C), 82.2, 79.2, 79.1, 73.7, 67.8, 54.0, 45.6 (2C). **HRMS *(ESI)*** m/z $[M+Na]^+$ calcd. for $C_{14}H_{14}NaO_2^+$: 237.0886; found 237.0881.

4.5 Preparation of FmocPhe ester ***(S)*-10**

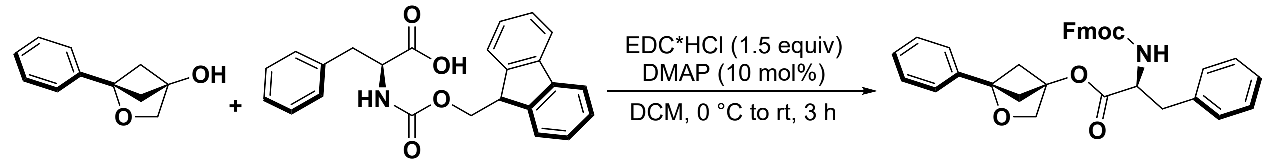

6 **Fmoc-PheOH** **(*S*)-10**

In a Schlenk flask, equipped with a magnetic stirring bar and under Ar atmosphere, compound **6** (0.10 mmol, 17.6 mg) was dissolved in dry DCM (0.5 mL) and the solution was cooled to 0 °C. Then, (*S*)-Fmoc-PheOH (0.15 mmol, 1.5 equiv, 58.1 mg), DMAP (0.01 mmol, 5 mol%, 1.2 mg) and EDC hydrochloride (28.7 mg) were sequentially added. The reaction mixture was stirred at 0 °C for 1 h and at room temperature for 2 h. A saturated solution of NH_4Cl (3 mL) was then added, followed by DCM (3 mL). The biphasic mixture was moved to a separatory funnel, the organic layer was separated, and the aqueous layer was extracted with DCM (2 x 3 mL). The combined organic layers were washed with NaH_4Cl (0.1 M, 3 x 2 mL), dried over Na_2SO_4 and concentrated in vacuo. The crude product was finally purified by FC on silica gel (*n*Hex/EtOAc 3:1) to afford pure product ***(S)*-10** as a white waxy solid (50.1 mg, 0.092 mmol, 92% yield).

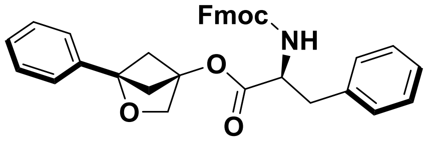

(S)*-10.** **$[\alpha]_D$** = - 44.6 (c = 0.33, $CHCl_3$). **^{1}H NMR** (600 MHz, $CDCl_3$) δ 7.71 (dt, *J* = 7.6, 0.9 Hz, 2H), 7.53 – 7.47 (m, 2H), 7.37 – 7.28 (m, 6H), 7.28 – 7.20 (m, 6H), 7.06 (d, *J* = 6.7 Hz, 2H), 5.20 (d, *J* = 8.3 Hz, 1H), 4.64 (dt, *J* = 8.2, 6.0 Hz, 1H), 4.41 (dd, *J* = 10.7, 7.1 Hz, 1H), 4.31 (dd, *J* = 10.7, 6.9 Hz, 1H), 4.15 (t, *J* = 7.0 Hz, 1H), 3.83 (d, *J* = 4.9 Hz, 1H), 3.81 (d, *J* = 5.0 Hz, 1H), 3.10 (dd, *J* = 13.8, 5.9 Hz, 1H), 3.05 (dd, *J* = 13.9, 6.2 Hz, 1H), 2.39 – 2.29 (m, 4H). **^{13}C NMR** (151 MHz, $CDCl_3$) δ 170.5, 155.5, 143.8 (2C, rotamer), 143.7 (2C, rotamer), 141.4 (2C), 136.6, 135.5, 129.4 (2C), 128.7 (2C), 128.4 (2C), 128.4, 127.8 (2C), 127.4, 127.1 (2C), 126.1 (2C), 125.1 (2C, rotamer), 125.0 (2C, rotamer), 120.1 (2C, rotamer), 120.0 (2C, rotamer), 83.7, 77.0 (overlapped with the $CDCl_3$ peak), 67.4, 67.0, 54.8, 47.9, 47.8, 47.2, 38.4. **HRMS *(ESI) m/z $[M+Na]^+$ calcd. for $C_{35}H_{31}NNaO_5^+$: 568.2094; found 568.2093.

4.6 Suzuki coupling towards product **11**

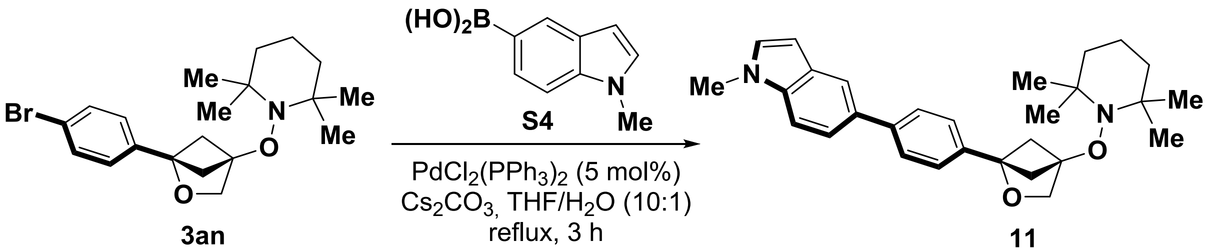

In a Schlenk tube, under an Ar atmosphere and magnetic stirring, bromo-derivative **3an** (39.3 mg, 0.10 mmol), Cs_2CO_3 (0.3 mmol, 81.0 mg) and boronic acid **S4** (26.3 mg, 0.15 mmol) were suspended in a THF/H_2O mixture (10:1, 0.55 mL). The resulting suspension was de-gassed by bubbling Ar for 1 min, then [$PdCl_2(PPh_3)_2$] (0.005 mmol, 3.5 mg, 5 mol%) was added and the resulting mixture was stirred at 70 °C for 3 h. The reaction mixture was cooled to room temperature and a saturated aqueous solution of K_2CO_3 (2 mL), H_2O (5 mL) and EtOAc (5 mL) were added, and the resulting biphasic mixture was transferred to a separatory funnel. The phases were separated, the aqueous layer was extracted again with EtOAc (5 mL), the combined organic phases were washed with a saturated aqueous solution of K_2CO_3 (2 x 5 mL), dried with Na_2SO_4, evaporated *in vacuo* and finally purified by FC on silica gel (*n*Hex/EtOAc 8:1) to afford pure product **11** as a white solid (38.6 mg, 0.087 mmol, 87% yield).

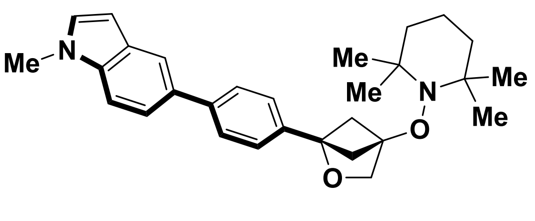

11. m.p. 115 - 117 °C. **^{1}H NMR** (600 MHz, $CDCl_3$) δ 7.84 (dd, *J* = 1.7, 0.7 Hz, 1H), 7.66 – 7.61 (m, 2H), 7.50 – 7.43 (m, 3H), 7.38 (dt, *J* = 8.5, 0.8 Hz, 1H), 7.08 (d, *J* = 3.0 Hz, 1H), 6.53 (dd, *J* = 3.1, 0.9 Hz, 1H), 3.97 (s, 2H), 3.82 (s, 3H), 2.35 – 2.31 (m, 2H), 2.29 – 2.25 (m, 2H), 1.65 – 1.58 (m, 1H), 1.54 – 1.43 (m, 4H), 1.35 (dp, *J* = 13.4, 3.4 Hz, 1H), 1.19 (s, 6H), 1.14 (s, 6H). **^{13}C NMR** (151 MHz, $CDCl_3$) δ 141.1, 135.2, 135.0, 131.4, 128.5, 127.9, 126.1 (2C), 125.3 (2C), 120.3, 118.3, 108.4, 100.3, 82.4, 80.8, 68.1, 58.3 (2C), 49.0 (2C), 38.8 (2C), 32.8 (2C), 31.9, 19.4 (2C), 16.1. **HRMS *(ESI)*** m/z $[M+Na]^+$ calcd. for $C_{29}H_{36}N_2NaO_2^+$: 467.2669; found 467.2676.

5. Crystallographic Data

The X-ray intensity data for **3aa** were collected on a Bruker APEXII CCD diffractometer using Mo−Kα radiation. All data were processed using the Bruker suite of programs[14] and the structures were solved by direct methods and refined with the SHELX program suite.[15] All non-hydrogen atoms were assigned anisotropic displacement parameters. Most of the hydrogen atoms were in the Fourier map, placed in idealized positions and included as riding with constrained isotropic displacement parameters for the aromatic, methylene and methyl protons and refined as riding with U_{iso}(H) = 1.2U_{eq}(C)) or U_{iso}(H) = 1.3U_{eq}(C_{methyl})). Molecular graphics were generated using the program Mercury.[16]

Table S1. Crystal data and experimental details for **3aa**

Compound	**3aa**
Formula	$C_{20}H_{29}NO_2$
Fw	315.44
T, K	296(2)
λ, Å	0.71073
Crystal symmetry	Monoclinic
Space group	*C2/c*
a, Å	26.224(3)
b, Å	6.2897(6)
c, Å	22.147(2))
α, °	90
β,°	98.901(4)
γ, °	90
Cell volume, Å^3	3609.0(6)
Z	8
D_c, Mg m^{-3}	1.161
μ(Mo-$K_α$), mm^{-1}	0.074
F(000)	1376
Crystal size/ mm	0.25 x 0.20 x 0.10

θ limits,°	1.861 to 24.999
Reflections collected	14821
Unique obs. Reflections [F_o >4σ(F_o)]	3092 [R(int) = 0.1142]
Goodness-of-fit-on F^2	1.161
R_1 (F)[a], wR_2 (F^2) [I > 2σ(I)][b]	0.1093, 0.2758
Largest diff. peak and hole, e. $Å^{-3}$	0.308 and -0.381

[a] $R_1 = S||F_o|-|F_c||/S|F_o|$. [b] $wR_2 = [Sw(F_o^2-F_c^2)^2/Sw(F_o^2)^2]^{1/2}$ where $w = 1/[s^2(F_o^2) + (aP)^2 + bP]$ where $P = (F_o^2 + F_c^2)/3$.

Crystallographic data have been deposited with the Cambridge Crystallographic Data Centre (CCDC) as supplementary publication number CCDC 2517247 for **3aa**. Copies of the data can be obtained free of charge via www.ccdc.cam.ac.uk/getstructures .

6. Voltametric Analyses

Cyclic voltammetry (CV) experiments were performed in a 15 mL vial using an Autolab PGSTAT302N potentiostat (Metrohm), with data acquisition controlled by the Nova 2.1.8 software. The analyte of choice was dissolved in 8.0 mL of acetonitrile (CH_3CN, 2.1 mmol L^{-1}). Tetraethylammonium tetrafluoroborate ($TEABF_4$) at a concentration of 0.1 mol L^{-1} was employed as the supporting electrolyte. The measurements were conducted at room temperature at a scan rate of 50 mV s^{-1} using a glassy carbon disk working electrode (3 mm diameter) and a platinum counter electrode (GC||Pt). A silver wire was used as the reference electrode, and all potentials were internally referenced to the ferrocene/ferrocenium (Fc/Fc^+) redox couple. The use of the ACN/THF mixture (employed in the preparative experiments) was avoided due to a reduced electrochemical window that would not allow to clearly discriminate the oxidation reactions occurring at high potentials. The use of $LiClO_4$ electrolyte was avoided for better electrodic stability.

Figure S3 shows the responses of TEMPO **2a** (grey line) and BCB **1a** (orange line), indicating the oxidation of **2a** occurring preferentially. The recorded potentials, over the Fc/Fc^+ redox-couple are $E_{1/2}$ = + 0.20 V for **2a** (reversible event) and E_p = + 0.81 V for **1a** (irreversible event).

Figure S4 shows the titration experiment reported in the main text, Scheme 4. The experiments were run with [**2a**] = 2.10 mM (all lines), [**1a**] = 0.84 mM (light blue line), [**1a**] = 1.68 mM (pink line), [**1a**] = 3.76 mM. This was carried out by adding 50 µL of a 0.1 M solution of **1a** in CAN, progressively in three portions, until a total of 150 µL were reached. The progressive disappearance of the return curve suggests an irreversible reaction of BCB **1a** with $TEMPO^+$, derived from the oxidation of **2a**.

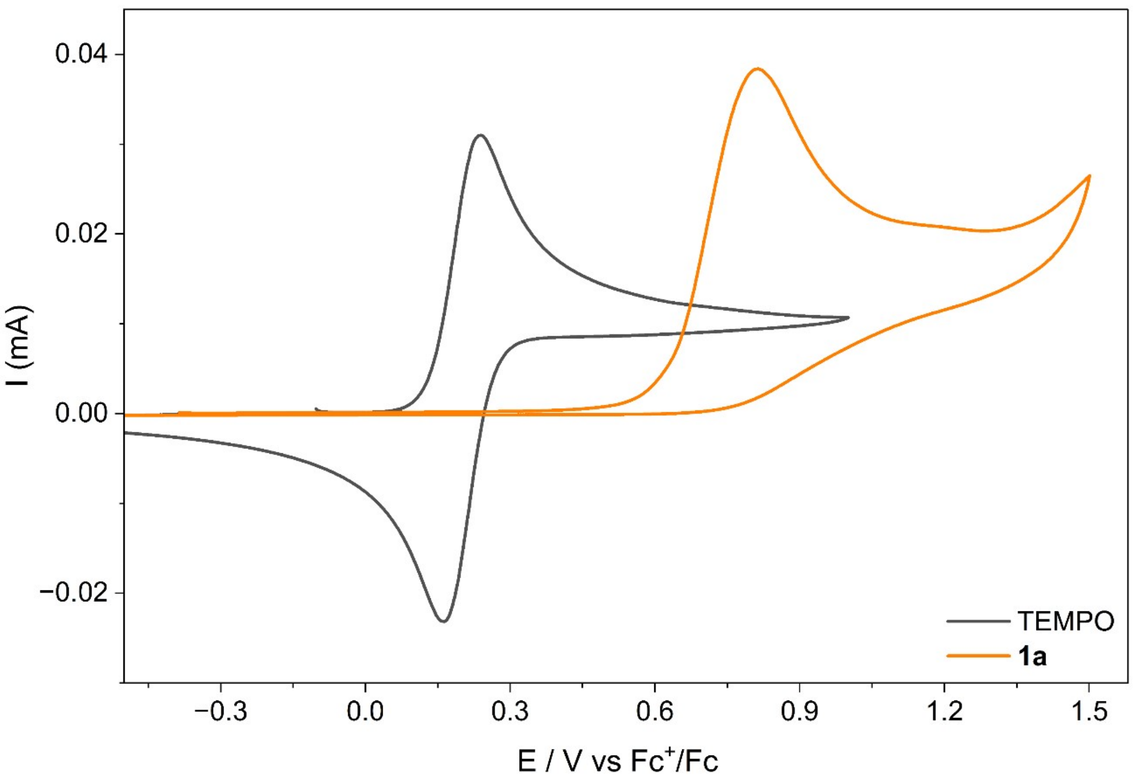

Figure S3. Voltametric responses of **1a** and **2a** plotted following the IUPAC convention.

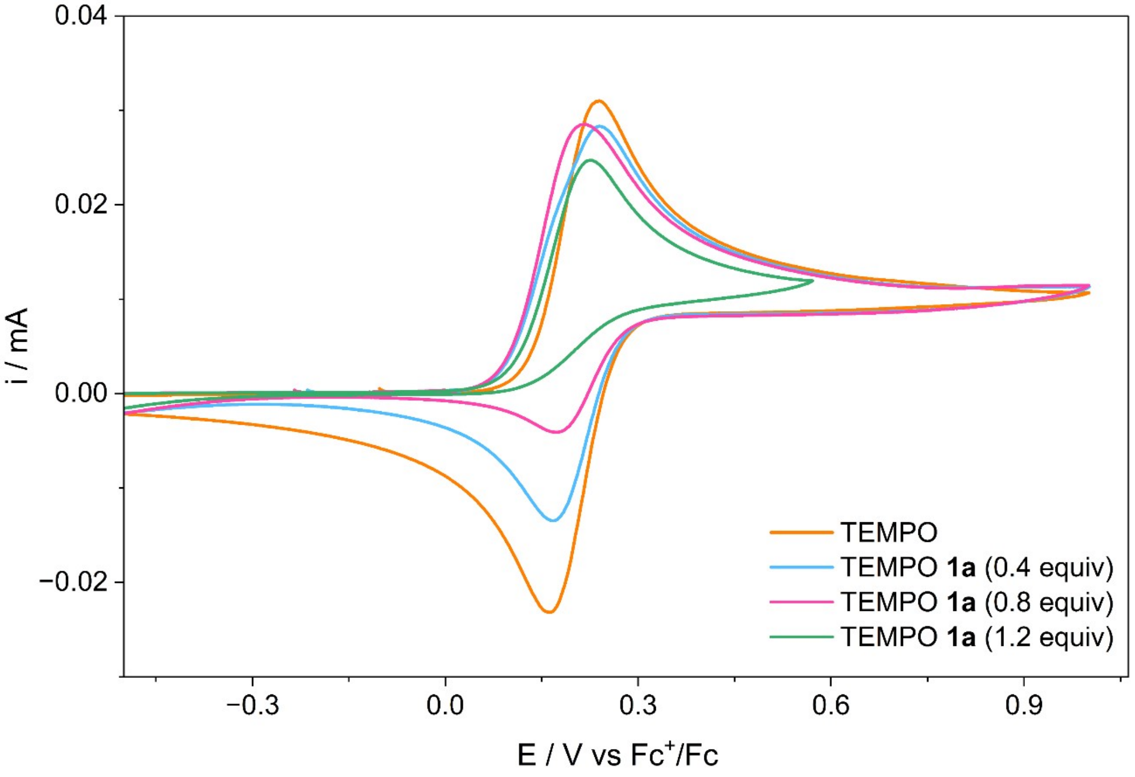

Figure S4. Voltametric responses of the titration experiment between **1a** and **2a** plotted following the IUPAC convention.

Figure S5 shows the response of TBAI **2e** ([**2e**] = 2.10 mM). Two oxidative irreversible events were recorded with E_{p1} = + 0.58 V and E_{p2} = + 1.19 V over the Fc/Fc^{+} redox-couple, respectively (blue line). These are attributed to the single-electron oxidation of the iodide anion to an iodo-radical (E_{p1}) and the single-electron oxidation of the so-formed iodo-radical to the iodonium cation (E_{p2}). While the first event shows a lower potential with respect to BCB **1a**, the second one occurs at a higher voltage. The formation of iodonium ions is thus deemed unlikely in the preparative reactions.

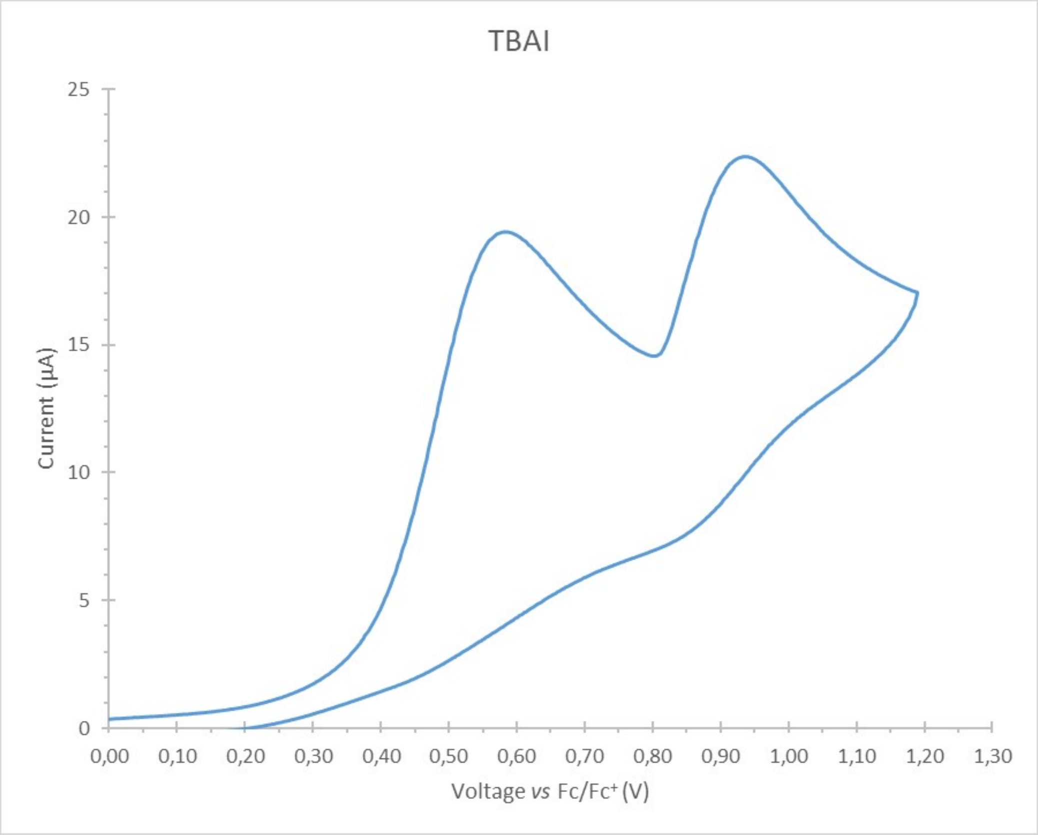

Figure S5. Voltametric response of **2e** plotted following the IUPAC convention.

Figure S6 shows the response of TBABr **2f** ([**2f**] = 2.10 mM). Two oxidative irreversible events were recorded with E_{p1} = + 0.79 V and E_{p2} = + 1.21 V over the Fc/Fc^{+} redox-couple, respectively (orange line). These are attributed to the single-electron oxidation of the bromide anion to a bromo-radical (E_{p1}) and the single-electron oxidation of the so-formed bromo-radical to the bromonium cation (E_{p2}). While the first event shows a lower potential with respect to BCB **1a**, the second one occurs at a higher voltage. The formation of bromonium ions is thus deemed unlikely in the preparative reactions.

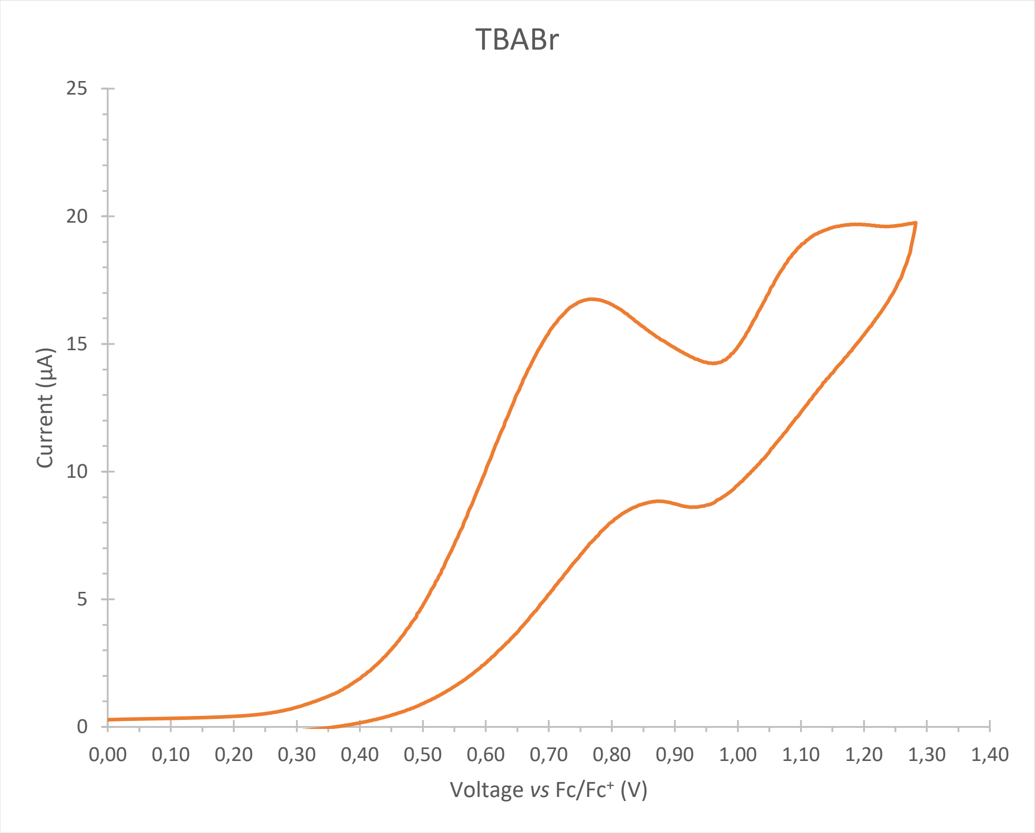

Figure S6. Voltametric response of **2f** plotted following the IUPAC convention.

Figure S7 shows the response of *p*-ClPhSH **2i** ([**2i**] = 2.10 mM). Two close oxidative events were recorded with E_{p1} = + 1.10 V and E_{p2} = + 1.34 V over the Fc/Fc^+ redox-couple, respectively (green line). This shows that any oxidation of protonated **2i** occurs later than the one of **1a**, providing an explanation on the unproductiveness of the preparative processes between thiols **2g-j** and BCB **1a** in the absence of a base. On the contrary, Figure S8 shows the response of the corresponding potassium salt *p*-ClPhSK, prepared *in situ* by mixing equimolar amounts of **2i** and KOtBu ([**2i**] = 2.10 mM, [KOtBu] = 2.10 mM). The curve shows that oxidation of the anionic species to the corresponding radical occurs very easily at E_p = - 0.43 V over the Fc/Fc^+ redox-couple (purple line). This supports the necessity of a base for the productive electrochemical thiolation of **1a** with thiophenols **2g-j**. In order to ascertain that the recorded event is not due to the oxidation of KOtBu, a separate experiment was run on KOtBu alone ([KOtBu] = 2.10 mM), showing no appreciable oxidation event (black line).

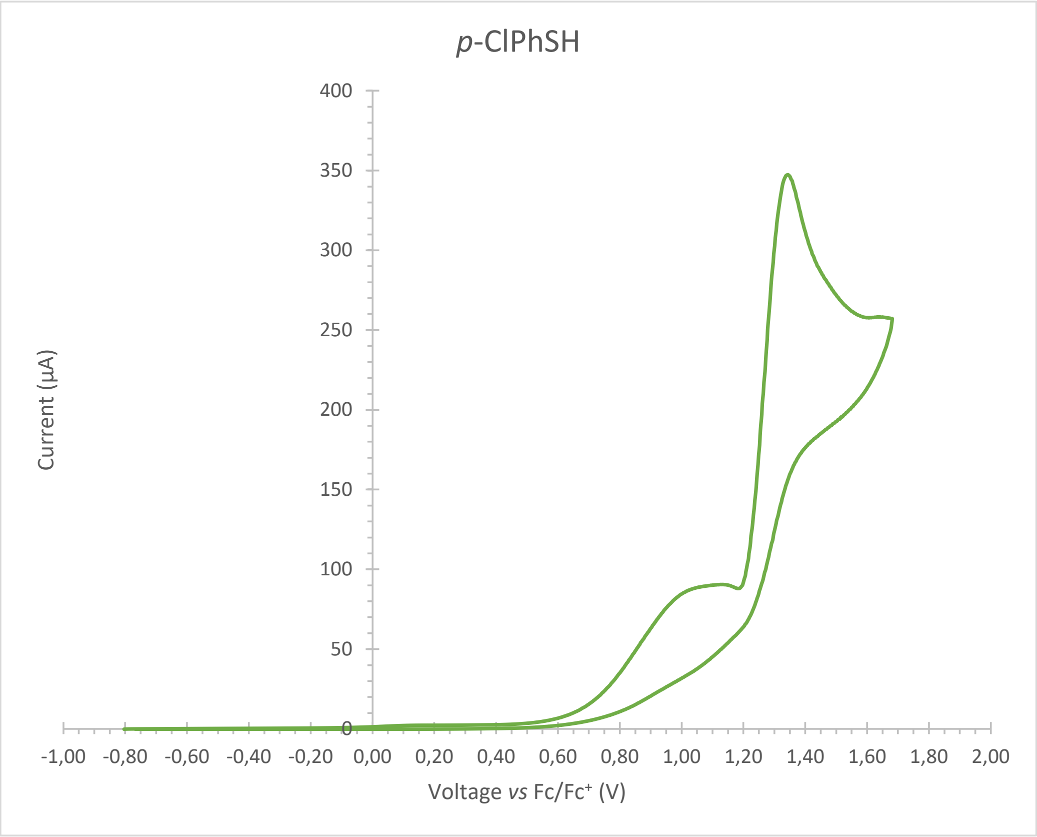

Figure S7. Voltametric response of **2i** plotted following the IUPAC convention.

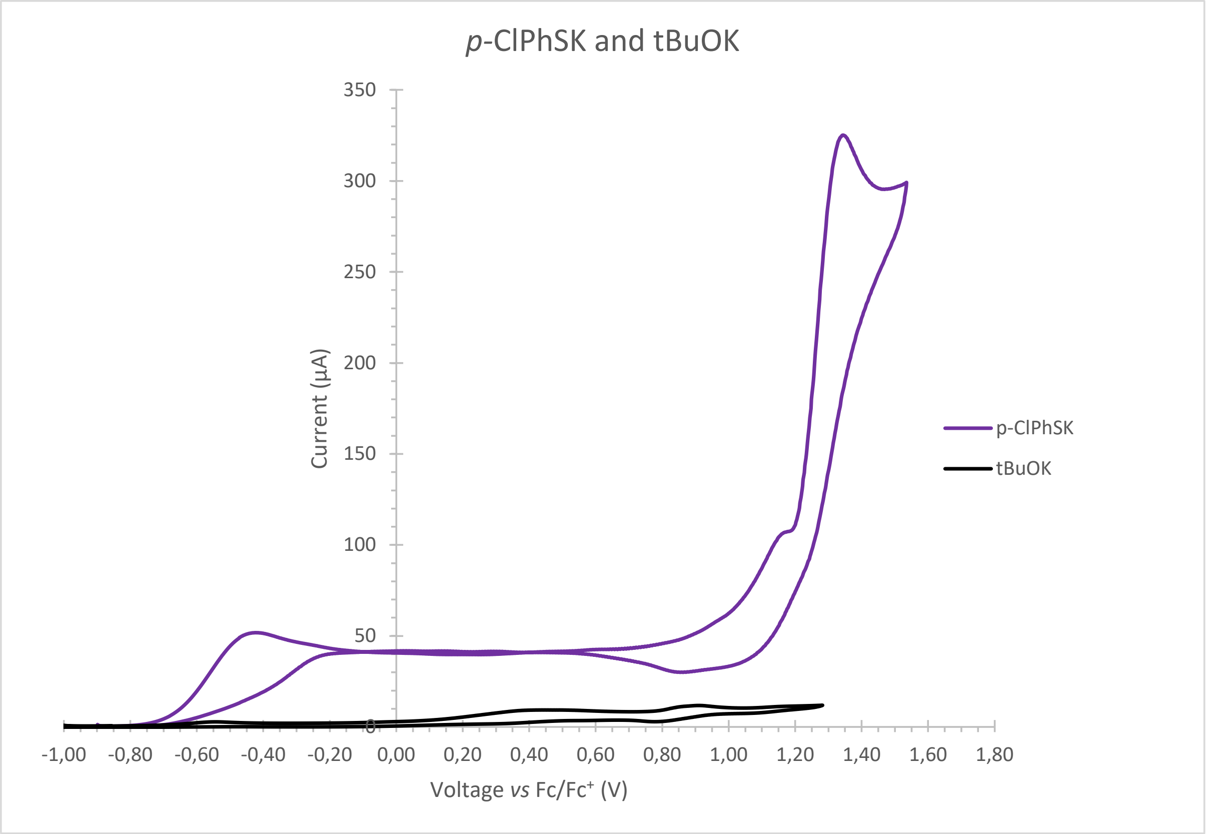

Figure S8. Voltametric responses of *p*-ClPhSK (**2i** + KOtBu) and KOtBu alone plotted following the IUPAC convention.

Note A: The working electrode was polished by brushing with diamond paste on a polyester cloth.

Note B: The solution was de-gassed by bubbling N_2 for 1 min prior to analysis. The N_2 flow was continued, above the solvent surface, during the analysis to prevent oxygen contamination.

*Note C: Initial potential: - 1.10 V vs Fc/Fc^+ for all analyses; direction of initial scan: oxidation; switching potential: + 0.9 V vs Fc/Fc^+ for **2a**, +1.5 V vs Fc/Fc^+ for **1a**, .+ 1.0 V vs Fc/Fc^+ for the titration experiment, + 1.20 V vs Fc/Fc^+ for **2e**, + 1.30 V vs Fc/Fc^+ for **2f**, + 1.80 V vs Fc/Fc^+ for **2i**, + 1.60 V vs Fc/Fc^+ for p-ClPhSK and + 1.30 V for KOtBu.*

7. Additional Mechanistic Elucidations.

7.1 Iodide, bromide and thiolate pathway

Figure S9 depicts the two mechanistic pathways proposed for the reaction of BCB **1a** with pro-electrophilic anions iodide (**2e**), bromide (**2f**) and thiophenolate (simplified model for **2g-j**). Table S2 summarizes the computed energies for the single-electron transfer (SET) oxidations (**a**, **b**, **c**), for the transition states leading to the C-X bond formation (**TS1** for the radical pathway, and **TS1'** for the cationic pathway) and for the transition state leading to the intramolecular capture of the carbocation (**TS2**) to deliver the final compounds **3**. All energies are given in kcal/mol, for the Computational Methodology see Chapter S1.

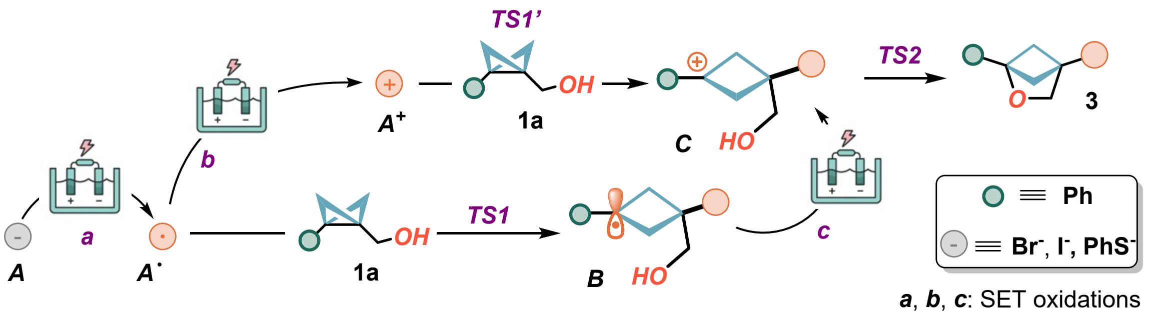

Figure S9. Proposed mechanistic alternatives for the idoide, bromide and thiolate functionalization. Upper line: cationic pathway; lower line: radical pathway.

Table S2. Energies for the SET-oxidations and transition states depicted in Figure S9.

Entry	Precursor	a	b	c	TS1	TS1'	TS2
1	I^-	132.1	210.3	112.3	barrierless	barrierless	4.8
2	Br^-	141.7	244.0	112.5	barrierless	barrierless	5.8
3	PhS^-	103.6	150.2	110.6	12.5	11.0	4.8

Regarding the pathways involving the activation of iodide and bromide anions (entries 1 and 2), both the cationic (**TS1'**) and the radical (**TS1**) transition states leading to the C-X bond forming event were found to be barrierless processes, as they involve very reactive and non-sterically hindered species. In these cases, we propose that the preferred pathway is the one requiring less energy input on the side of the SET oxidations. Indeed, direct capture of radical **A**· renders intermediate **B**, whose oxidation, being easier with respect to the initial formation of radical **A**· itself (compare step **a** and **b**), can be considered a downstream process. It is therefore deemed highly unlikely that the process proceeds *via* the formation

of cationic species **A⁺**, as not only their formation is very energy-demanding, but it is also not necessary for the system to proceed further (barrierless **TS1**).

Similarly, in the case of thiophenolate (entry 3), where the cationic and the radical transition states (**TS1** and **TS1’**) are non-negligible events and very close in energy, we again propose that, in order to dictate which machinery is the operating one, the attention should be turned to the SET oxidation steps (**b** *vs* **c**). Also in this case, the cationic pathway requires higher energy with respect to the radical one.

7.2 TEMPO (**2a**) pathway

Figure S10 depicts the two mechanistic pathways proposed for the reaction of BCB **1a** with pro-electrophilic radical TEMPO **2a**. Table S3 summarizes the computed energies for the single-electron transfer (SET) oxidations (**b**, **c**), for the transition states leading to the C-O bond formation (**TS1** for the radical pathway, and **TS1'** for the cationic pathway) and for the transition state leading to the intramolecular capture of the carbocation (**TS2**) to deliver the final compound **3aa**. All energies are given in kcal/mol, for the Computational Methodology see Chapter S1.

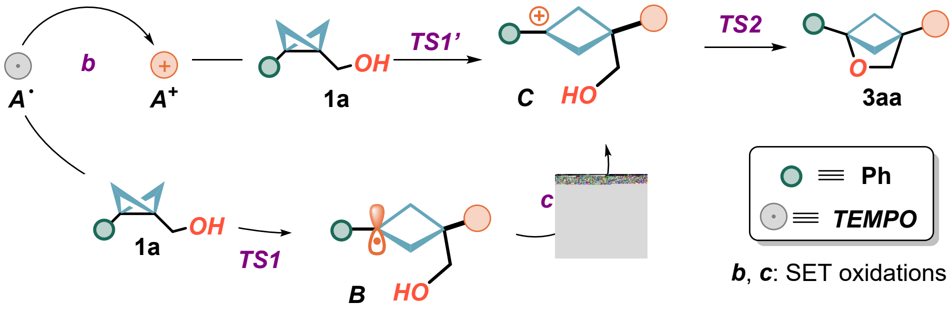

Figure S10. Proposed mechanistic alternatives for TEMPO (**2a**) functionalization. Upper line: cationic pathway; lower line: radical pathway

Table S3. Energies for the SET-oxidations and transition states depicted in Figure S10.

Entry	Precursor	**b**	**c**	**TS1**	**TS1'**	**TS2**
1	TEMPO·	121.8	108.7	36.7	16.7	4.9

In this case, we propose an opposite scenario to the one depicted in the previous examples, with the chosen pathway being dictated by the most accessible C-O forming event (**TS1** *vs* **TS1'**). If on one hand the least demanding machinery on the SET-oxidation point of view would be the radical pathway (easier oxidation of intermediate **B** to intermediate **C** *via* step **c** then of **A**· to **A**+ *via* step **b**), this also requires to overcome a very high energy barrier (**TS1**) for the C-O bond formation. Contrarily, the cationic pathway is much more easily accessed with a low transition state energy (**TS1'**).[17] This proposal can be also easily rationalized considering the different "philicity" of the examined radicals. While iodo, bromo and thio radicals are electrophilic in nature and their capture by nucleophilic **1a** is supposed to be favoured, TEMPO radical **2a** is nucleophilic and a sluggish reaction with mis-matched **1a** is forseeable.

This scenario is also heavily corroborated by the voltametric analyses showing the reactivity of TEMPO cation (**A⁺**) with **1a** (Figure S4) and by the experimental observation that the reaction of TEMPO and **1a**, in the absence of current, does not lead to product formation, nor to appreciable consumption of the starting material (Scheme S1).

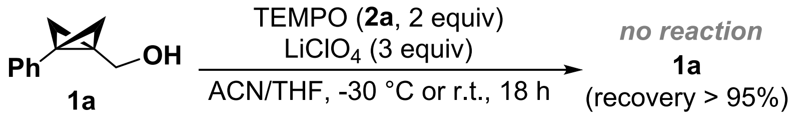

Scheme S1. No reaction is observed between **1a** and **2a** in the absence of electric current.

8. NMR Spectra

***d$_4$*-S1a ^{1}H NMR** (600 MHz, $CDCl_3$)

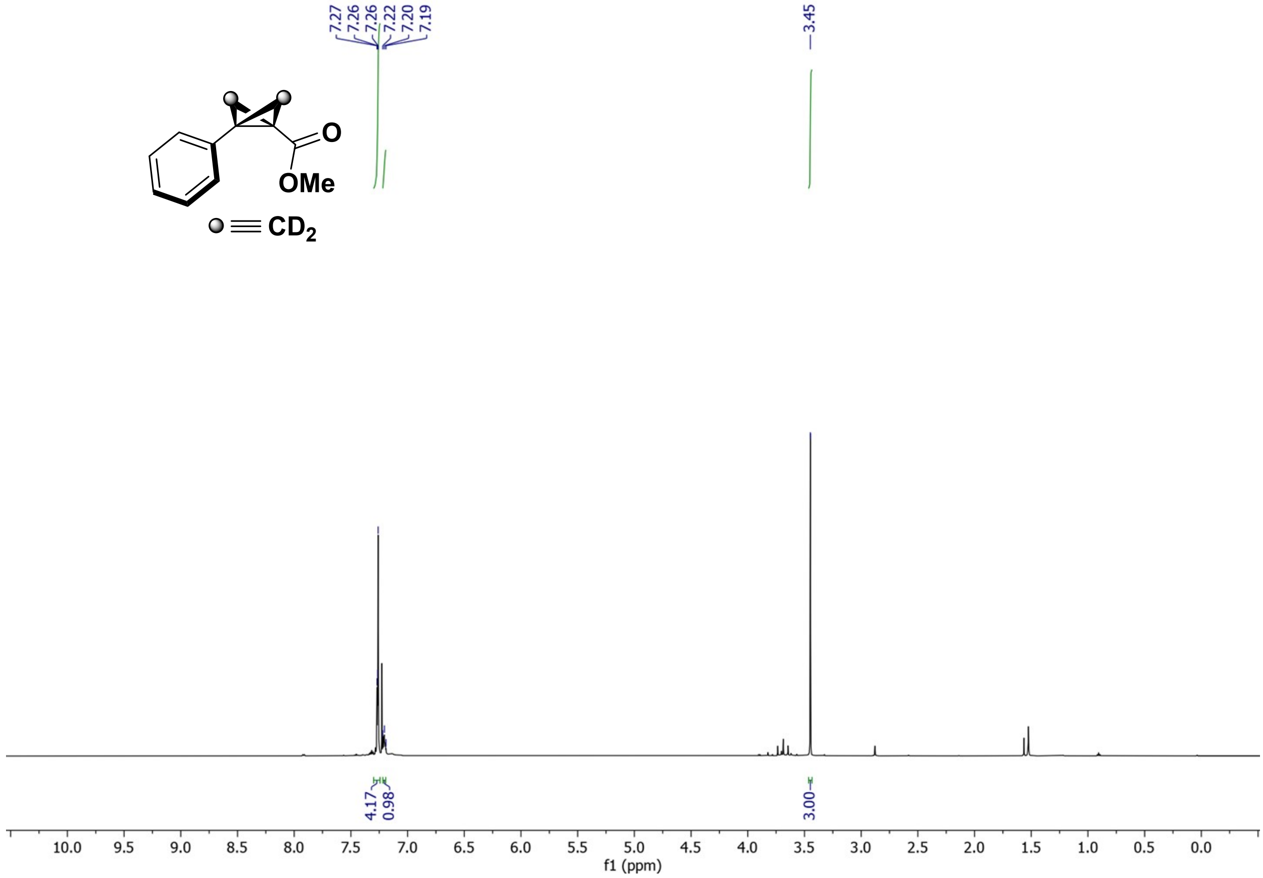

***d$_4$*-S1a ^{13}C NMR** (151 MHz, $CDCl_3$)

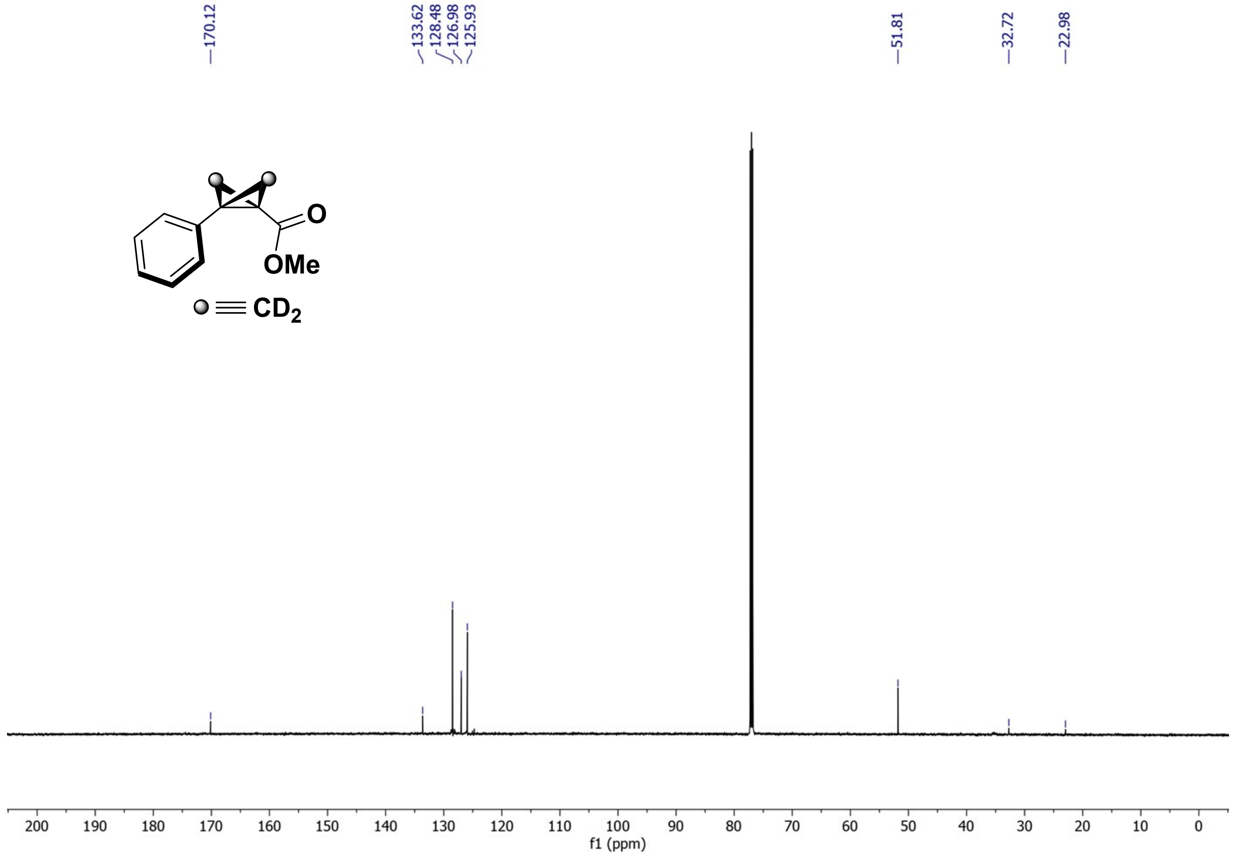

1a ^{1}H NMR (600 MHz, $CDCl_3$)

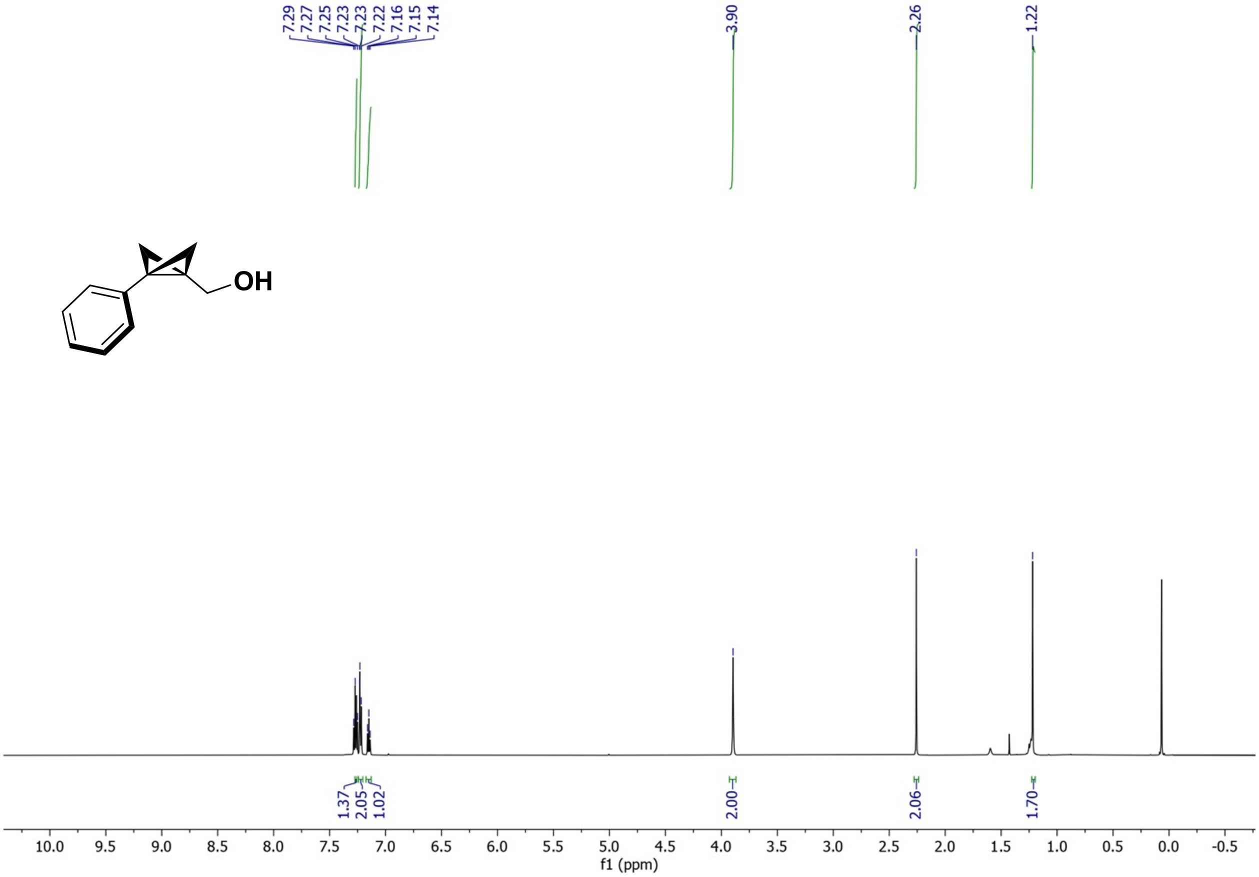

1a ^{13}C NMR (151 MHz, $CDCl_3$)

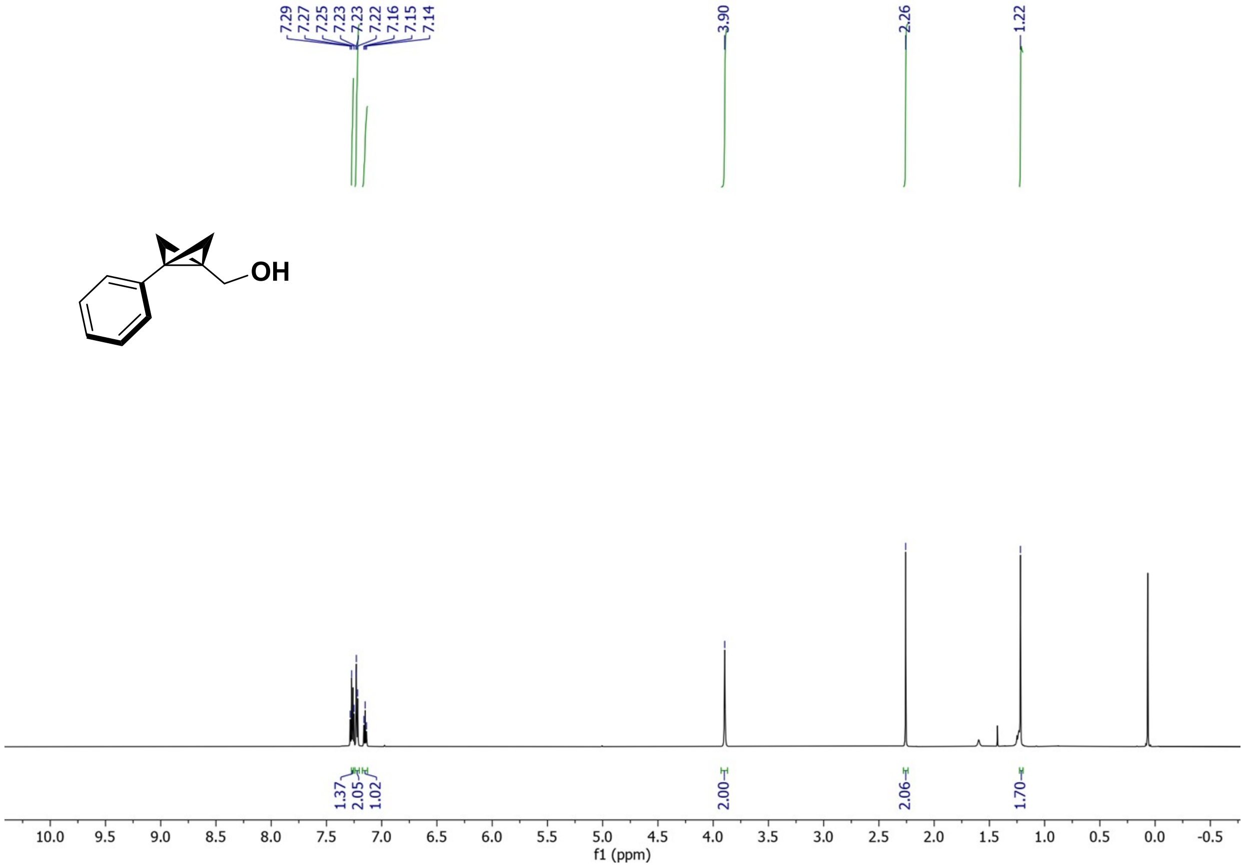

1b ^{1}**H NMR** (600 MHz, CDCl$_3$)

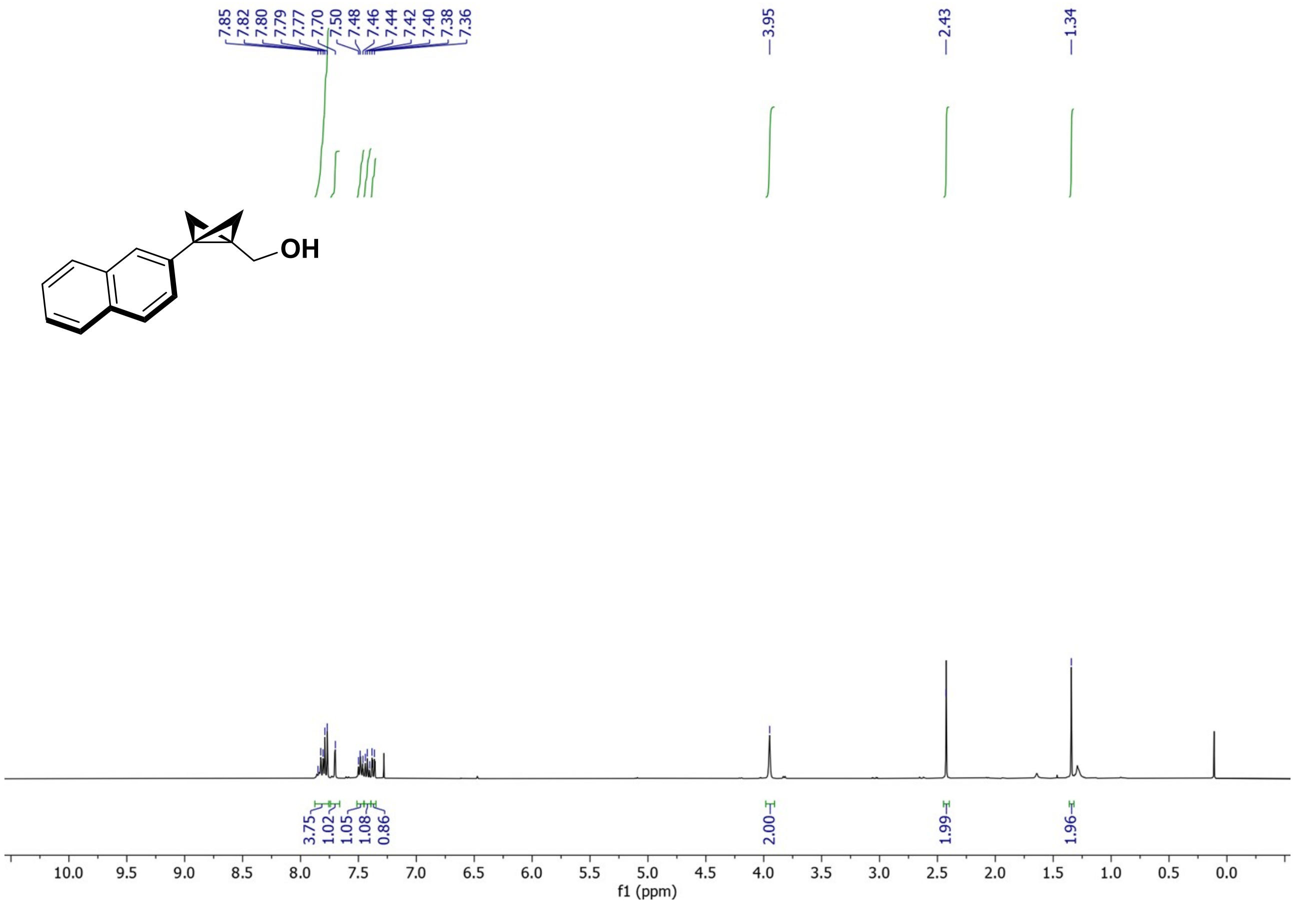

1b ^{13}**C NMR** (151 MHz, CDCl$_3$)

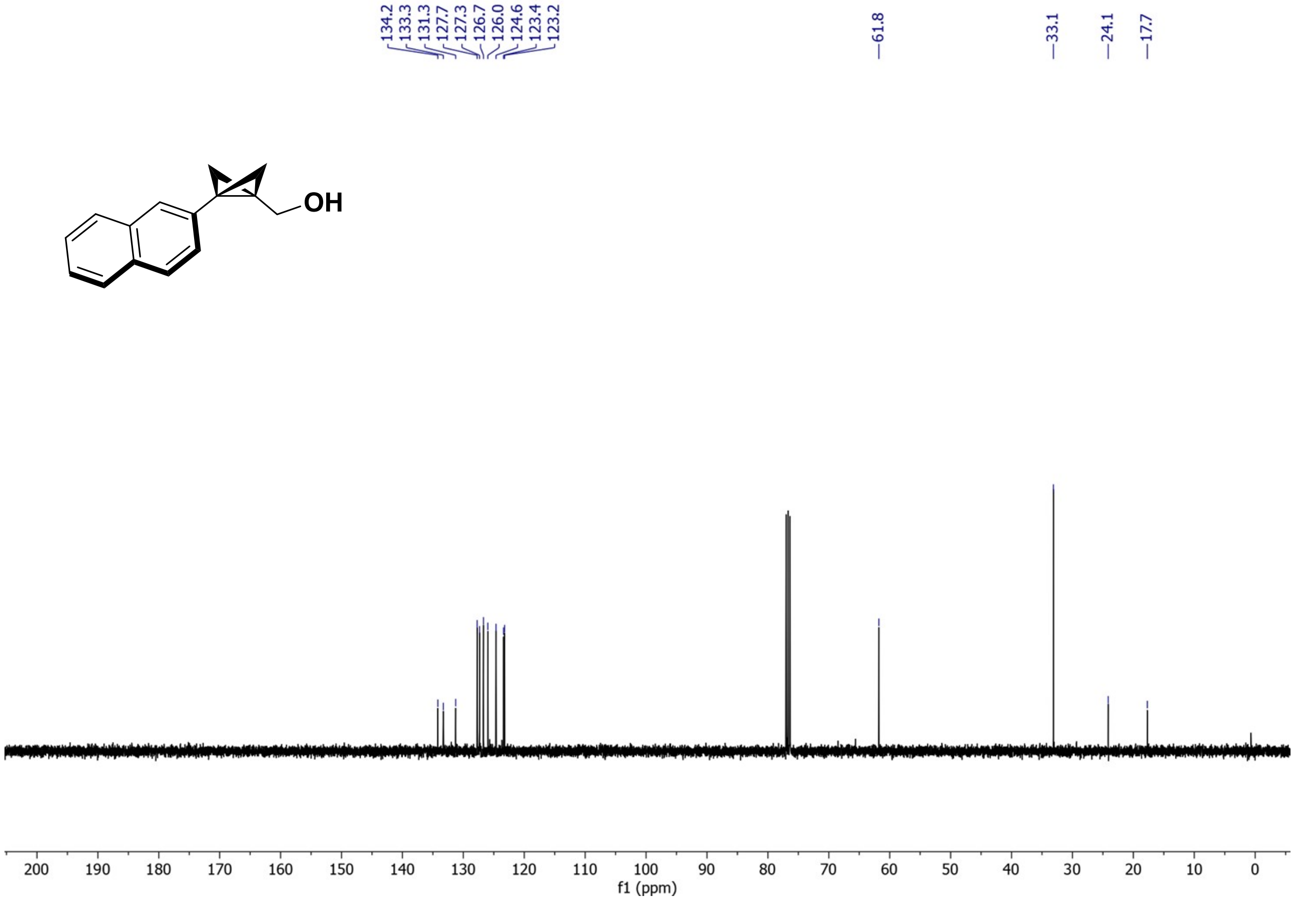

1c ^{1}H NMR (600 MHz, $CDCl_3$)

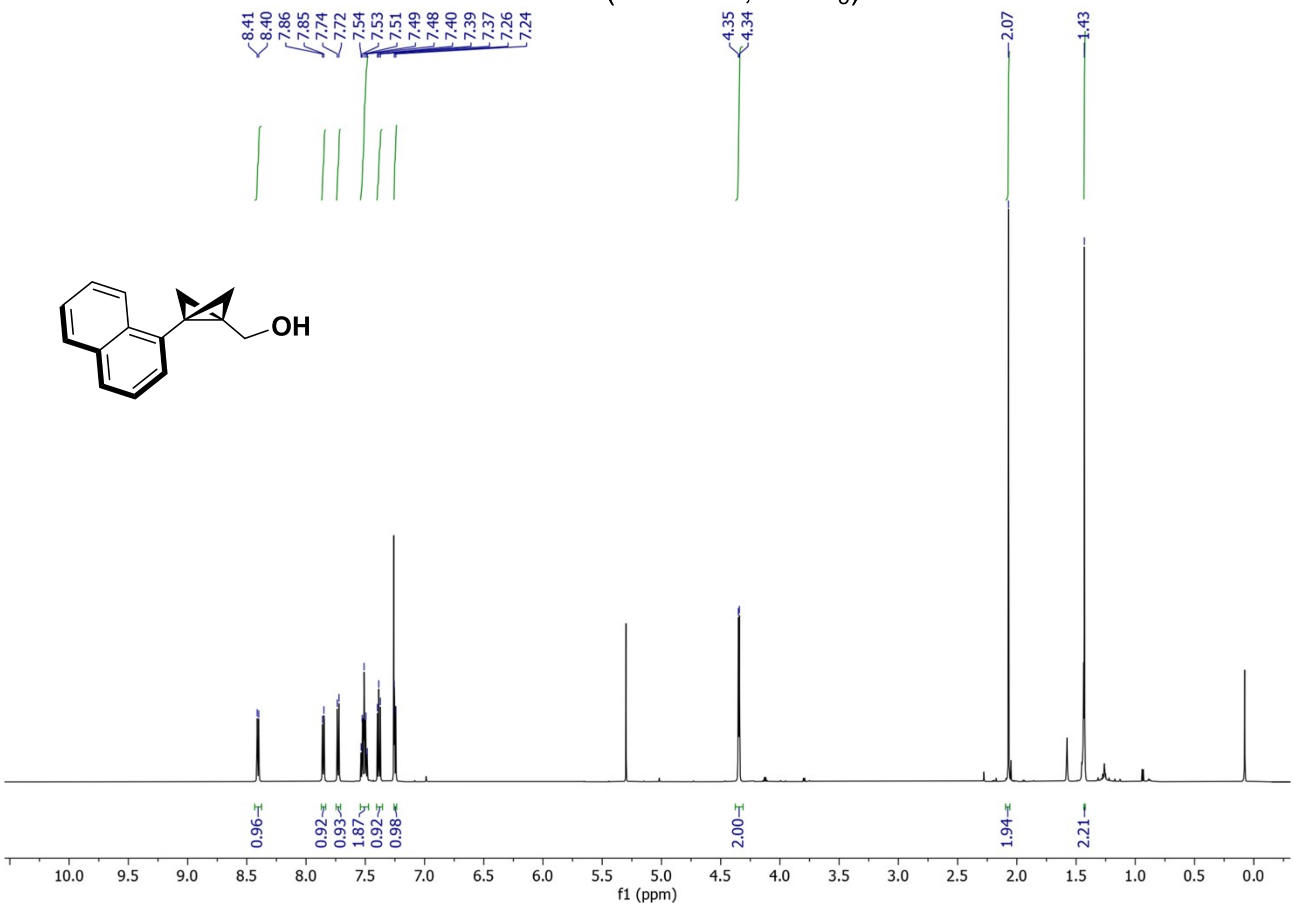

1c ^{13}C NMR (151 MHz, $CDCl_3$)

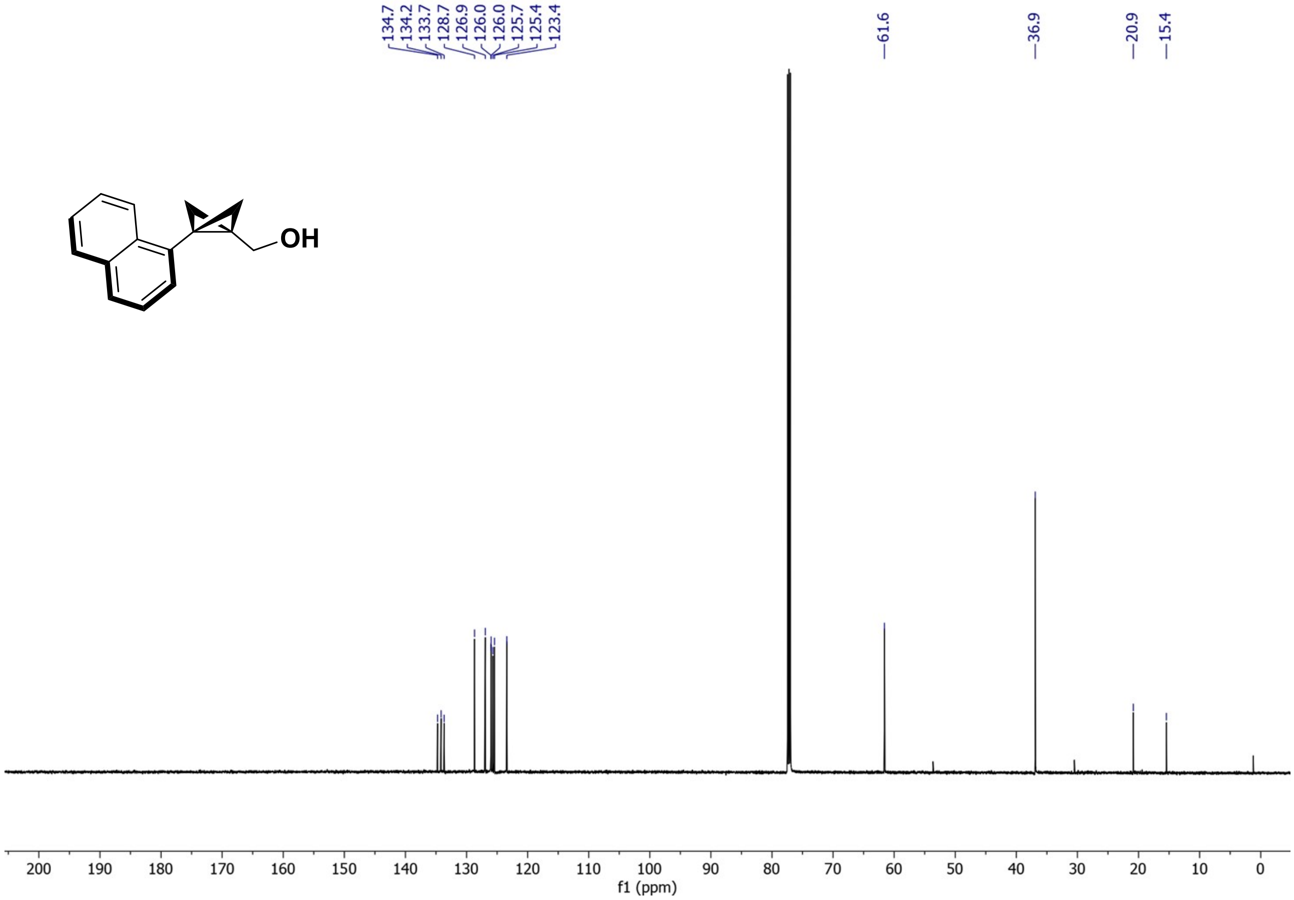

1d ^{1}H NMR (600 MHz, $CDCl_3$)

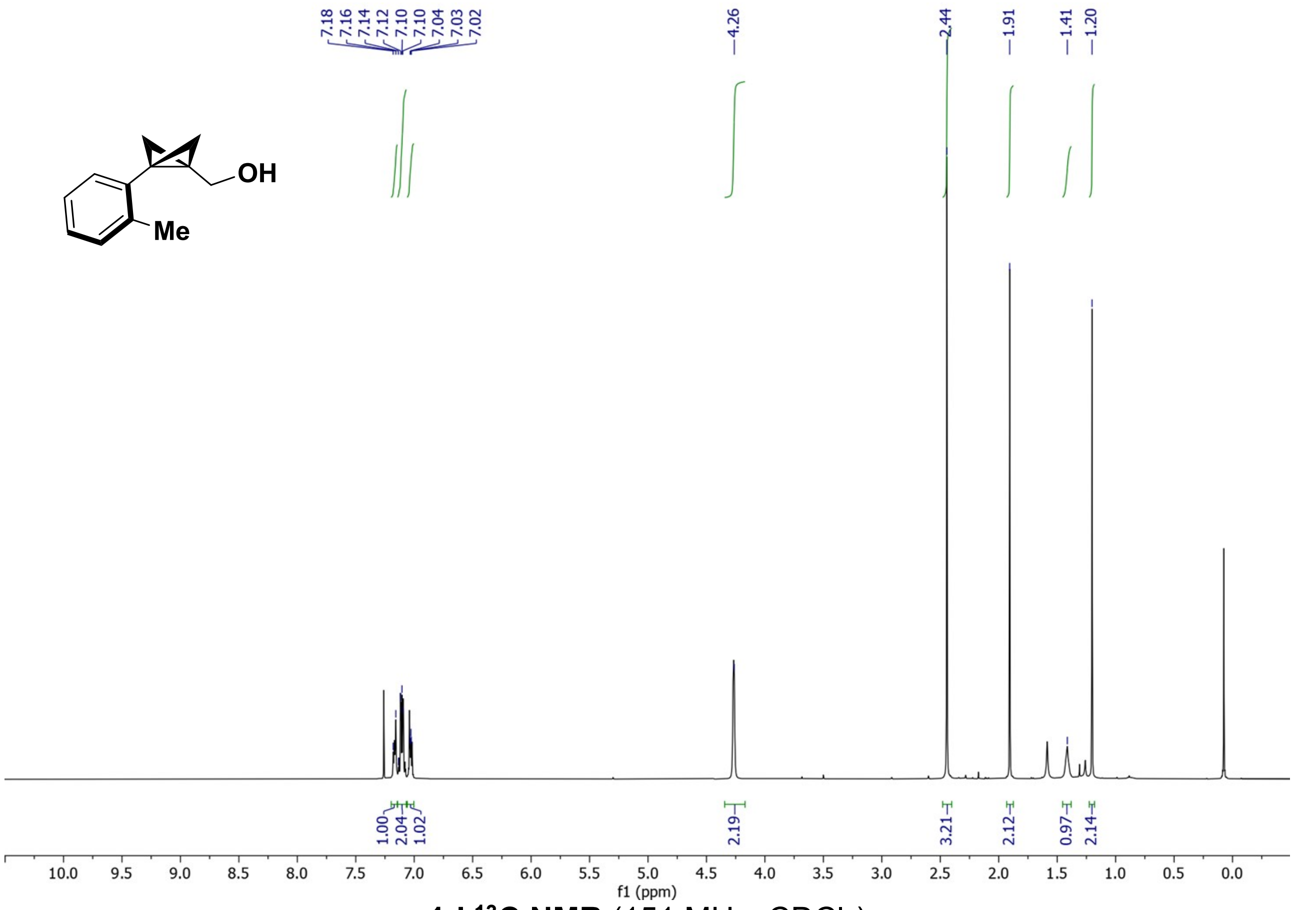

1d ^{13}C NMR (151 MHz, $CDCl_3$)

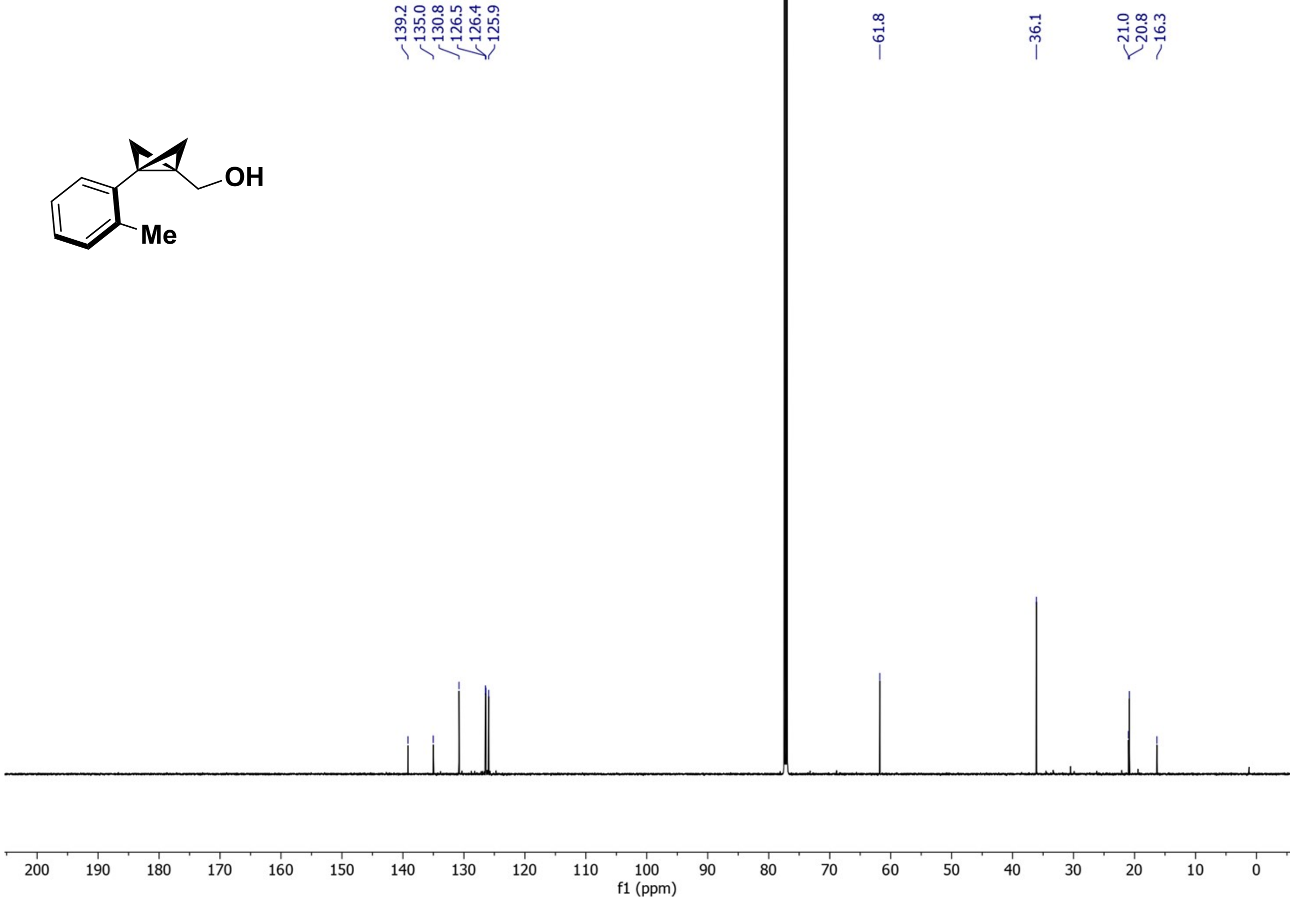

1e ^{1}H NMR (600 MHz, $CDCl_3$)

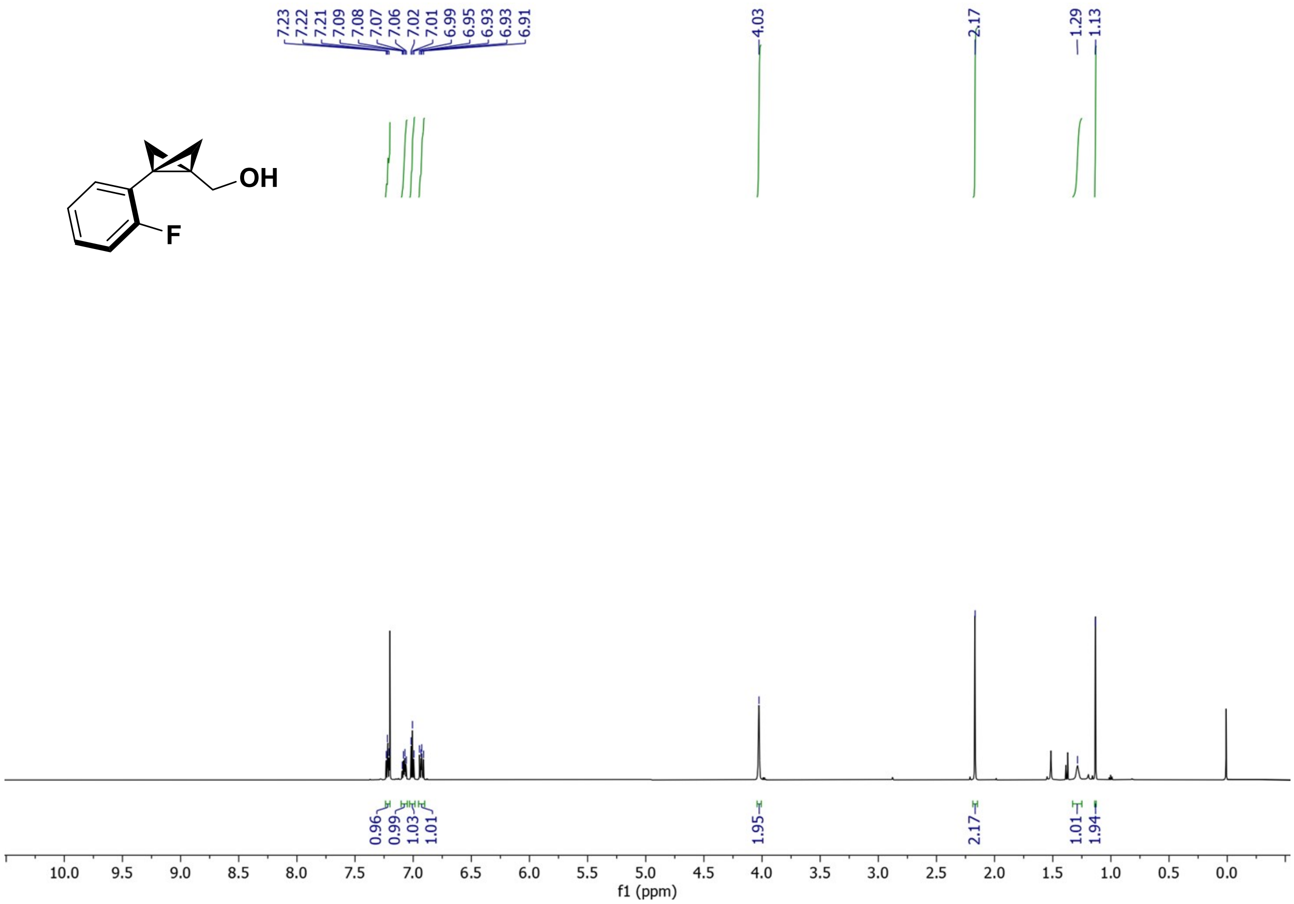

1e ^{13}C NMR (151 MHz, $CDCl_3$)

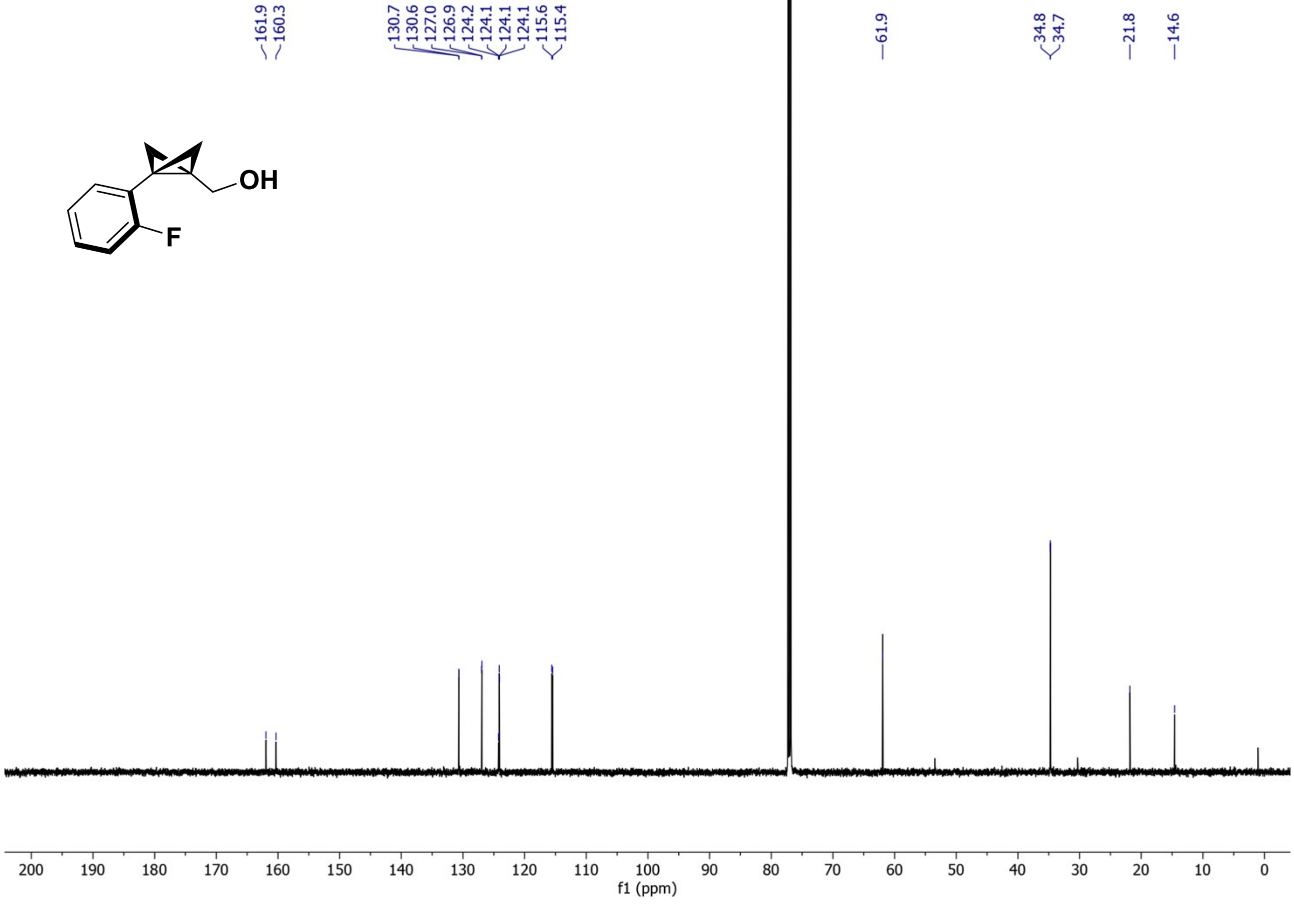

1e ¹⁹F NMR (565 MHz, $CDCl_3$)

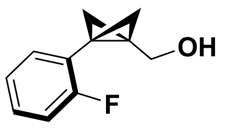

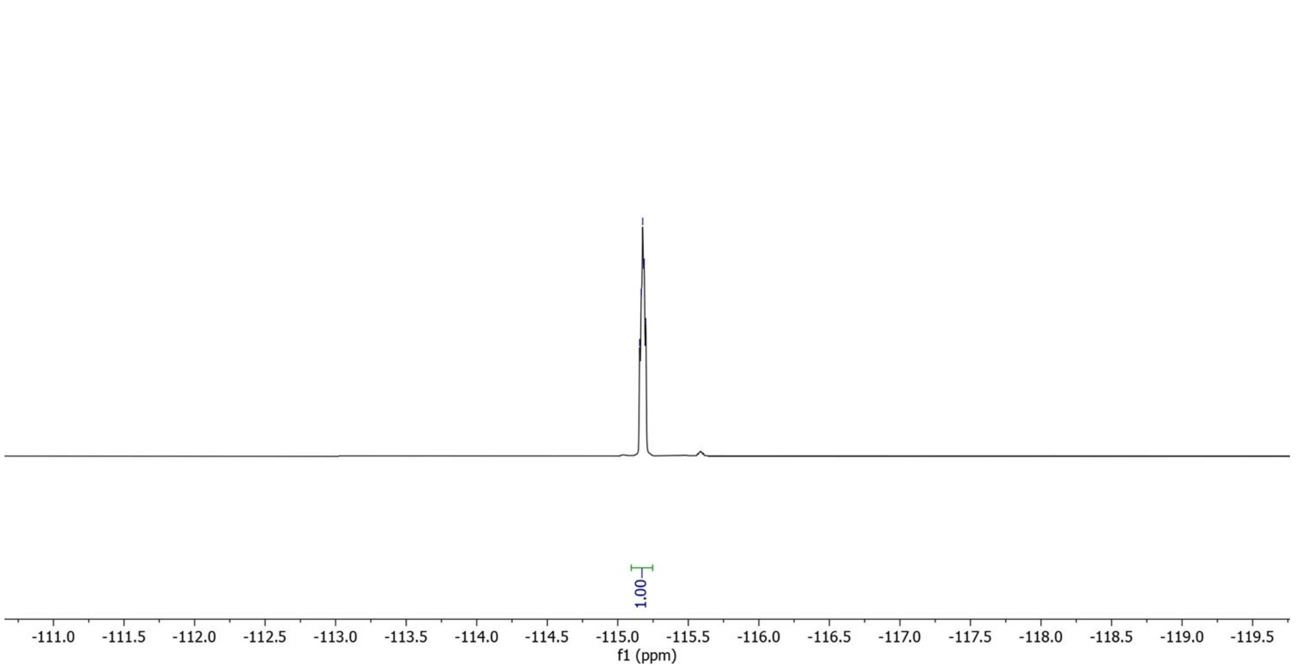

1f ^{1}H NMR (600 MHz, $CDCl_3$)

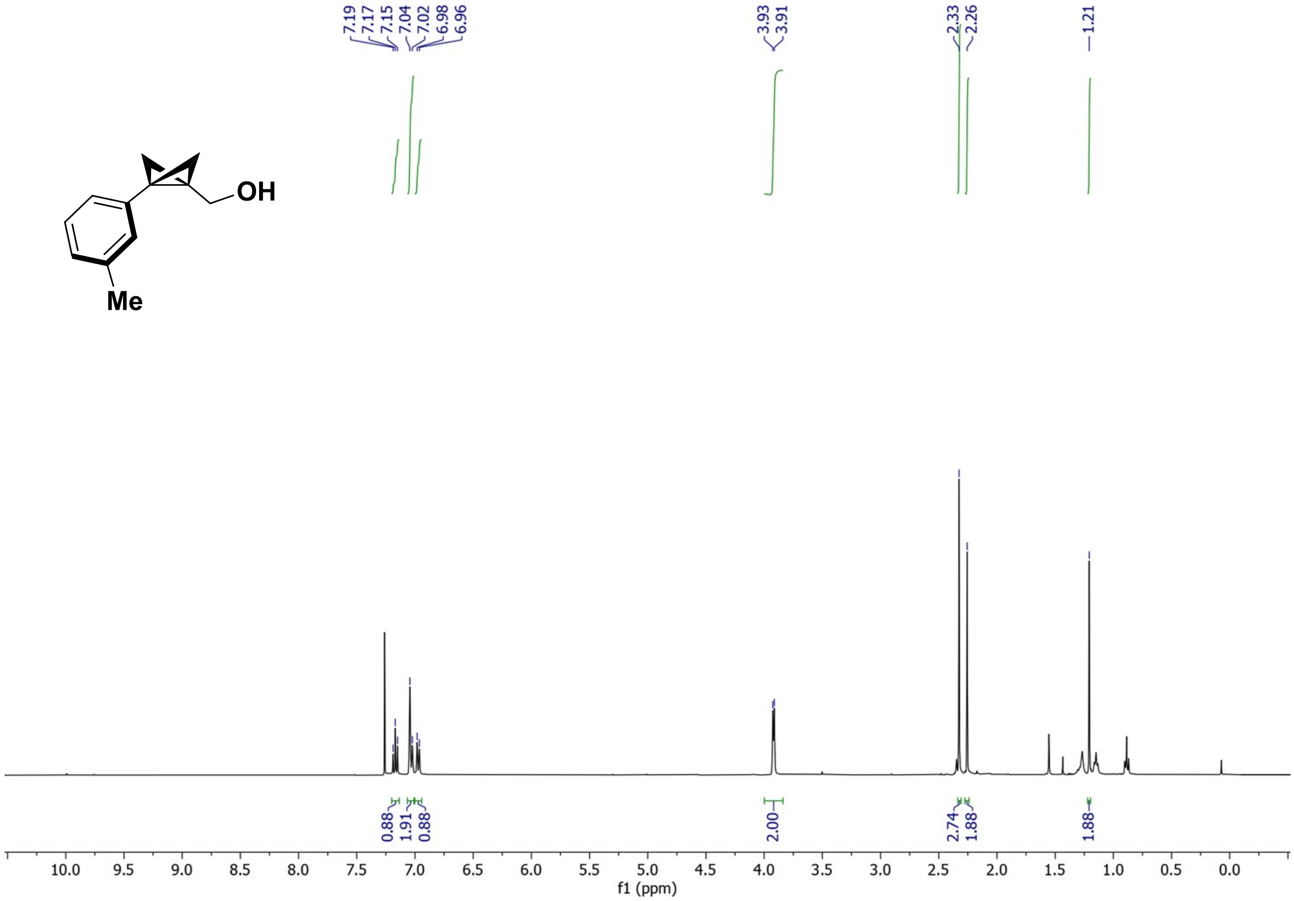

1f ^{13}C NMR (151 MHz, $CDCl_3$)

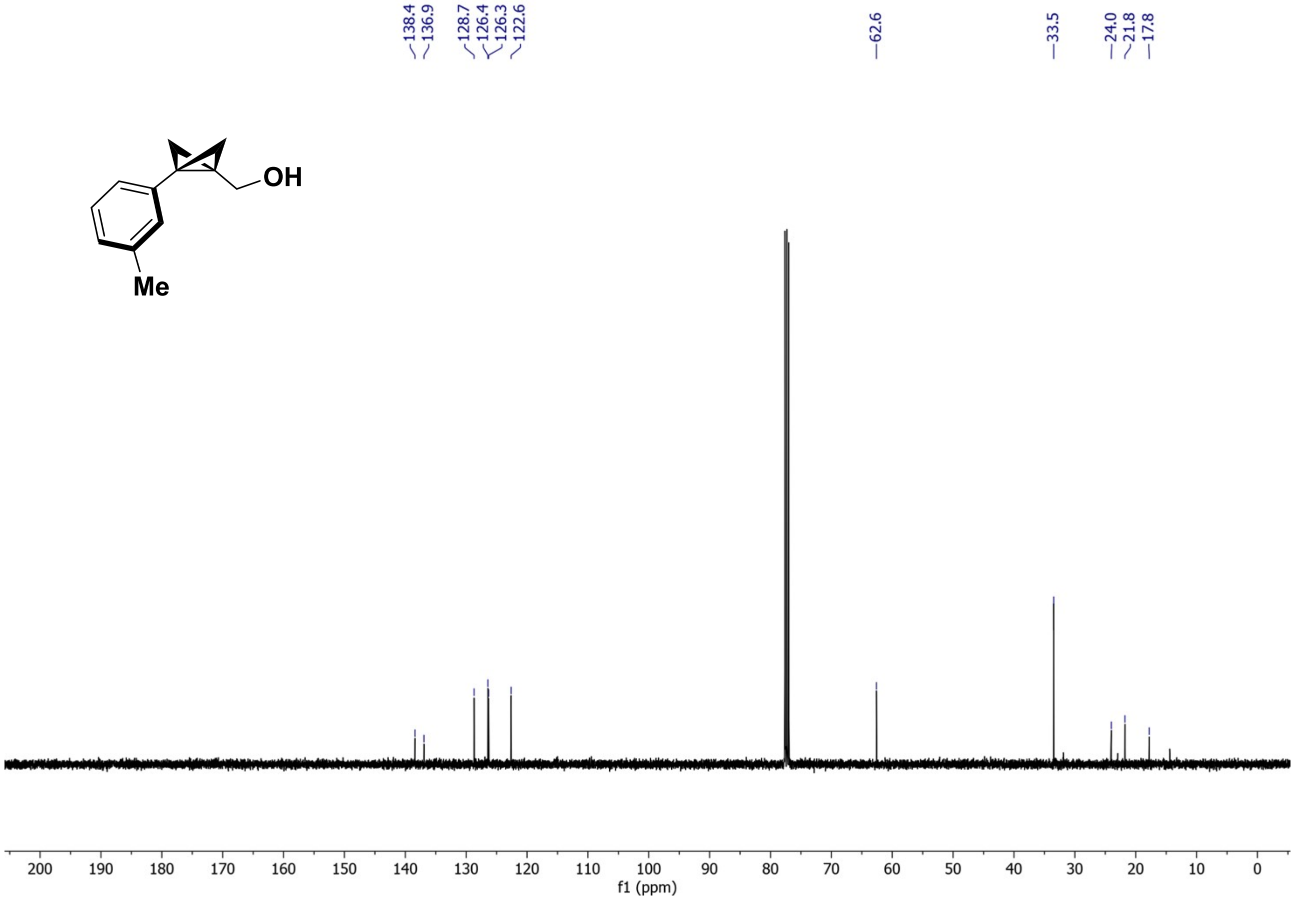

1g ^{1}H NMR (600 MHz, $CDCl_3$)

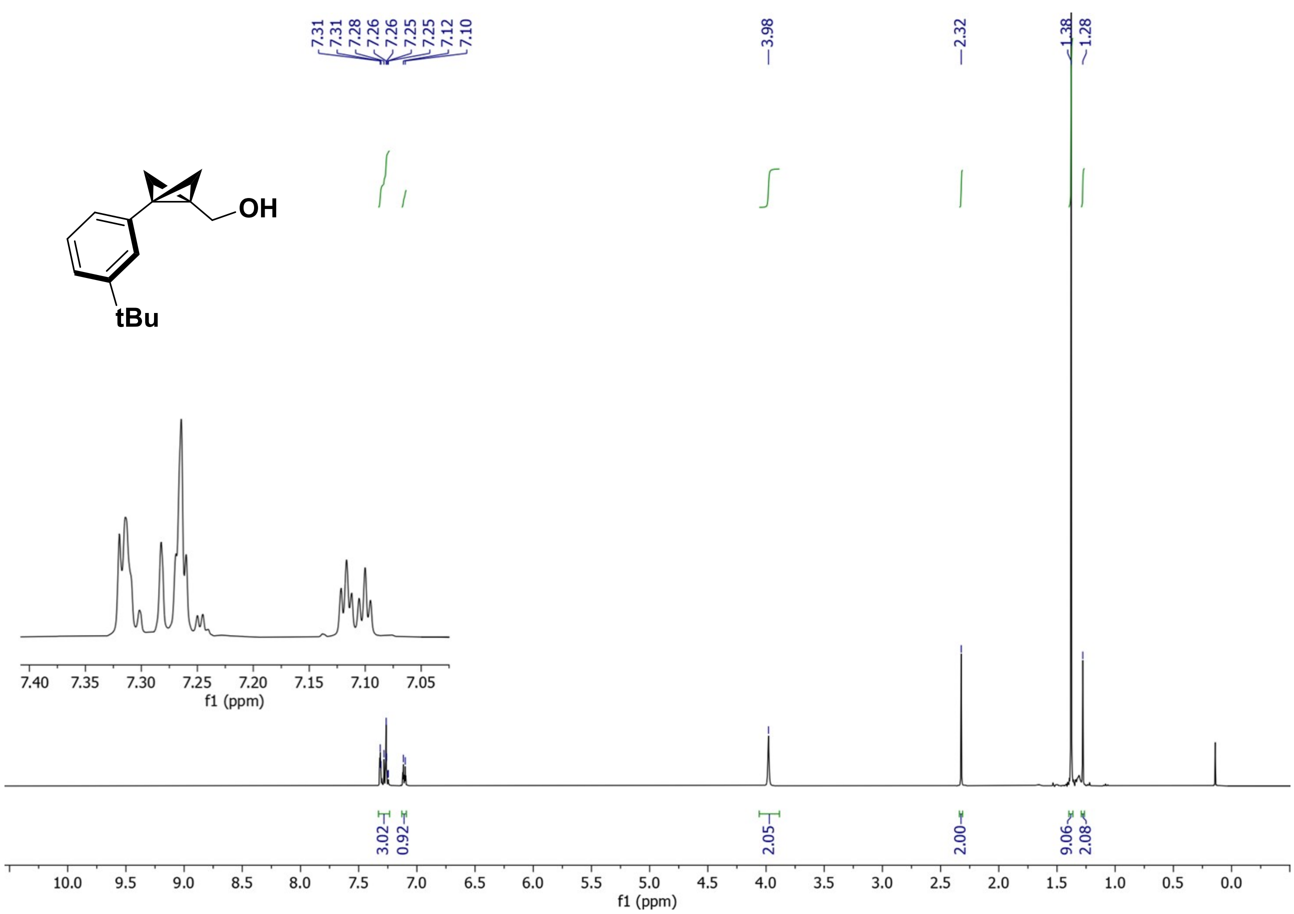

1g ^{13}C NMR (151 MHz, $CDCl_3$)

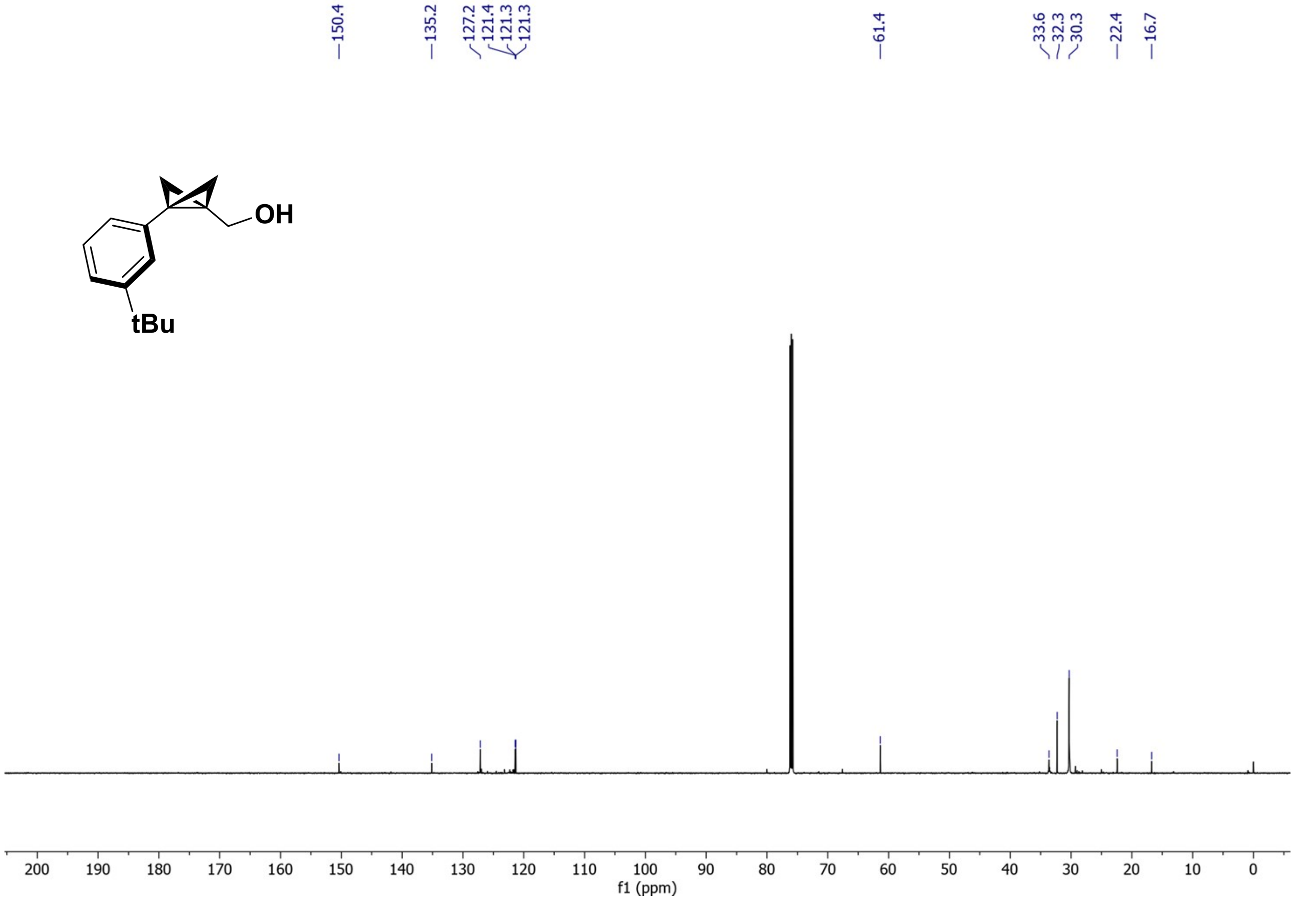

1h ^{1}H NMR (600 MHz, $CDCl_3$)

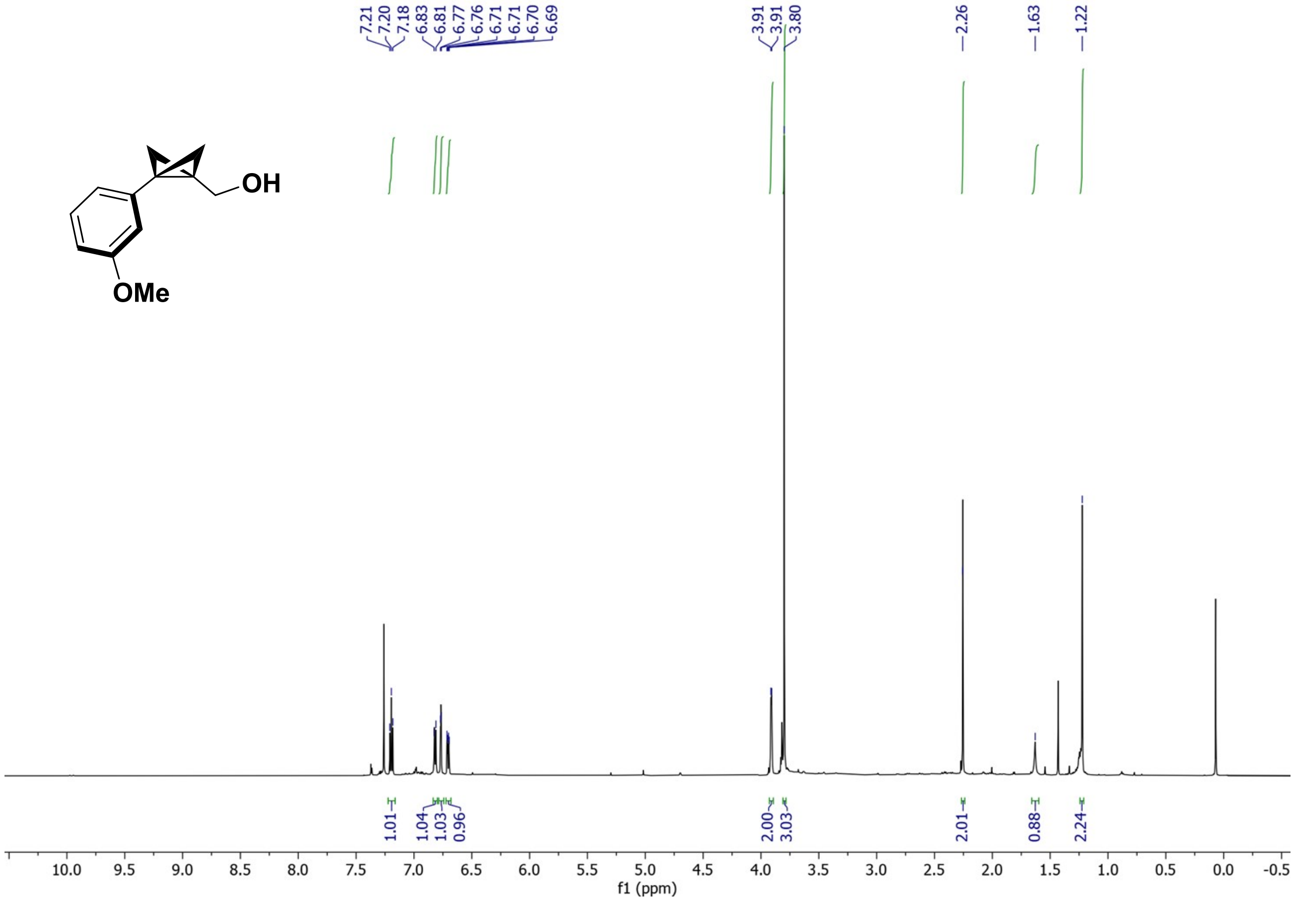

1h ^{13}C NMR (151 MHz, $CDCl_3$)

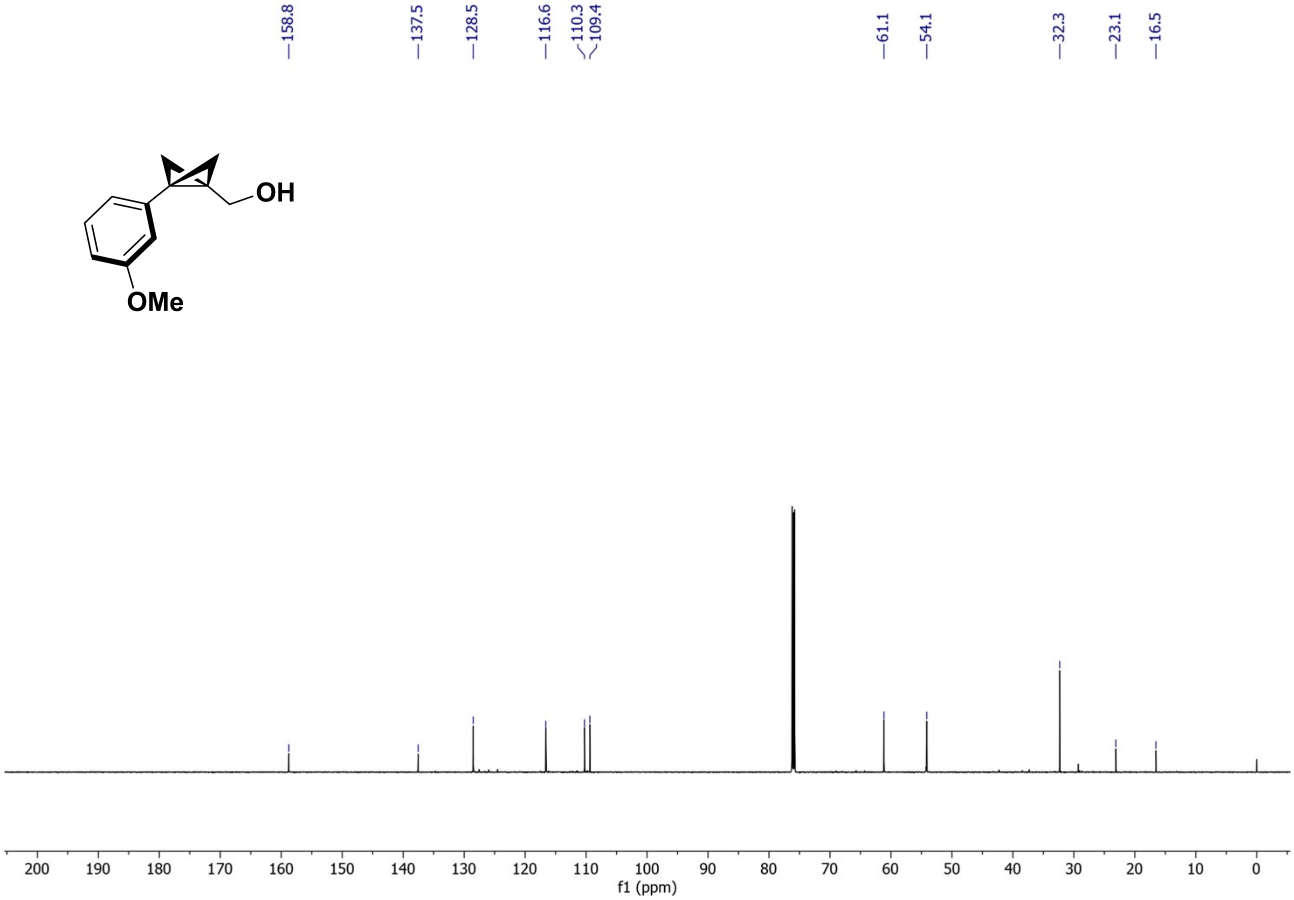

1i ^{1}H NMR (600 MHz, $CDCl_3$)

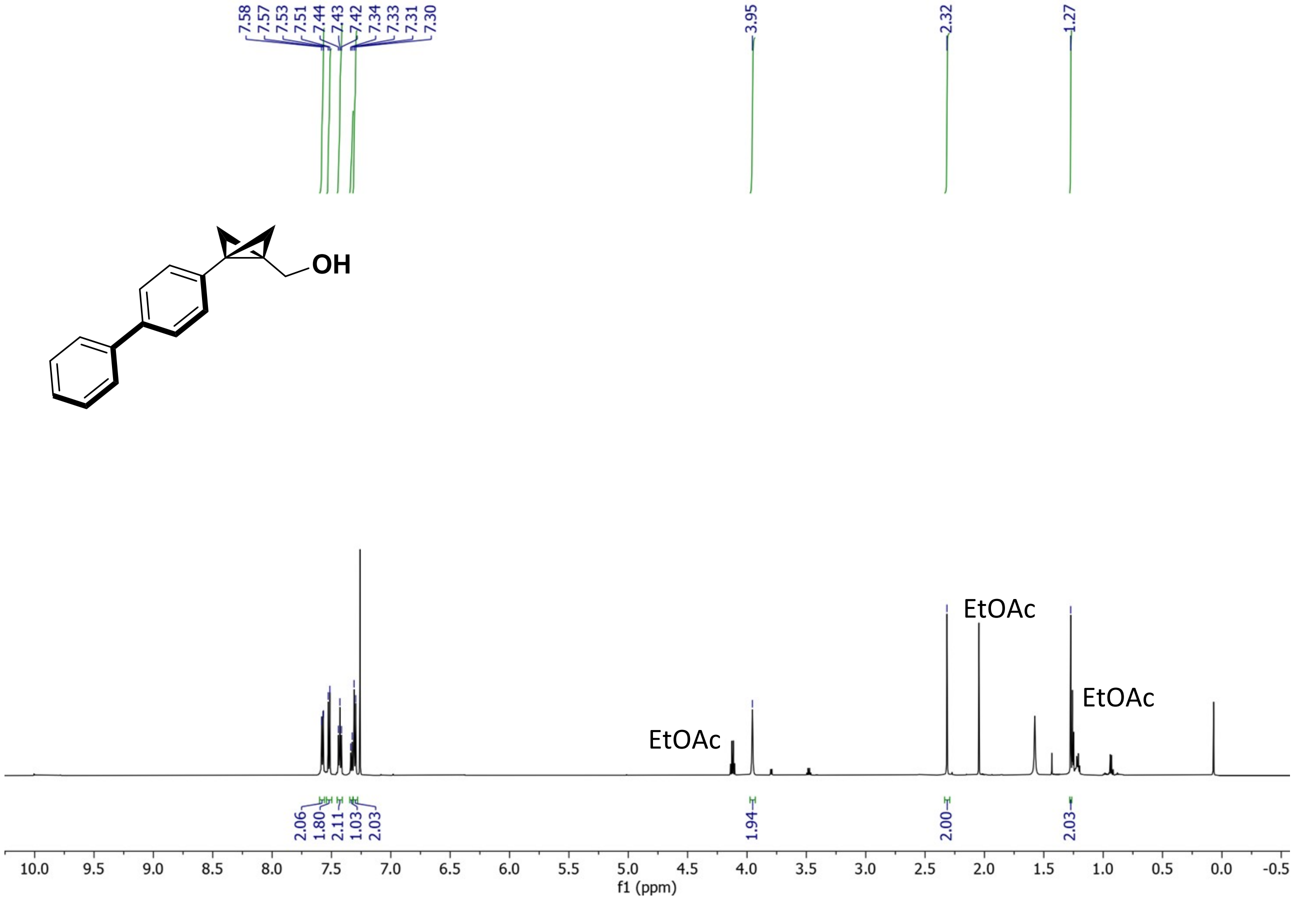

1i ^{13}C NMR (151 MHz, $CDCl_3$)

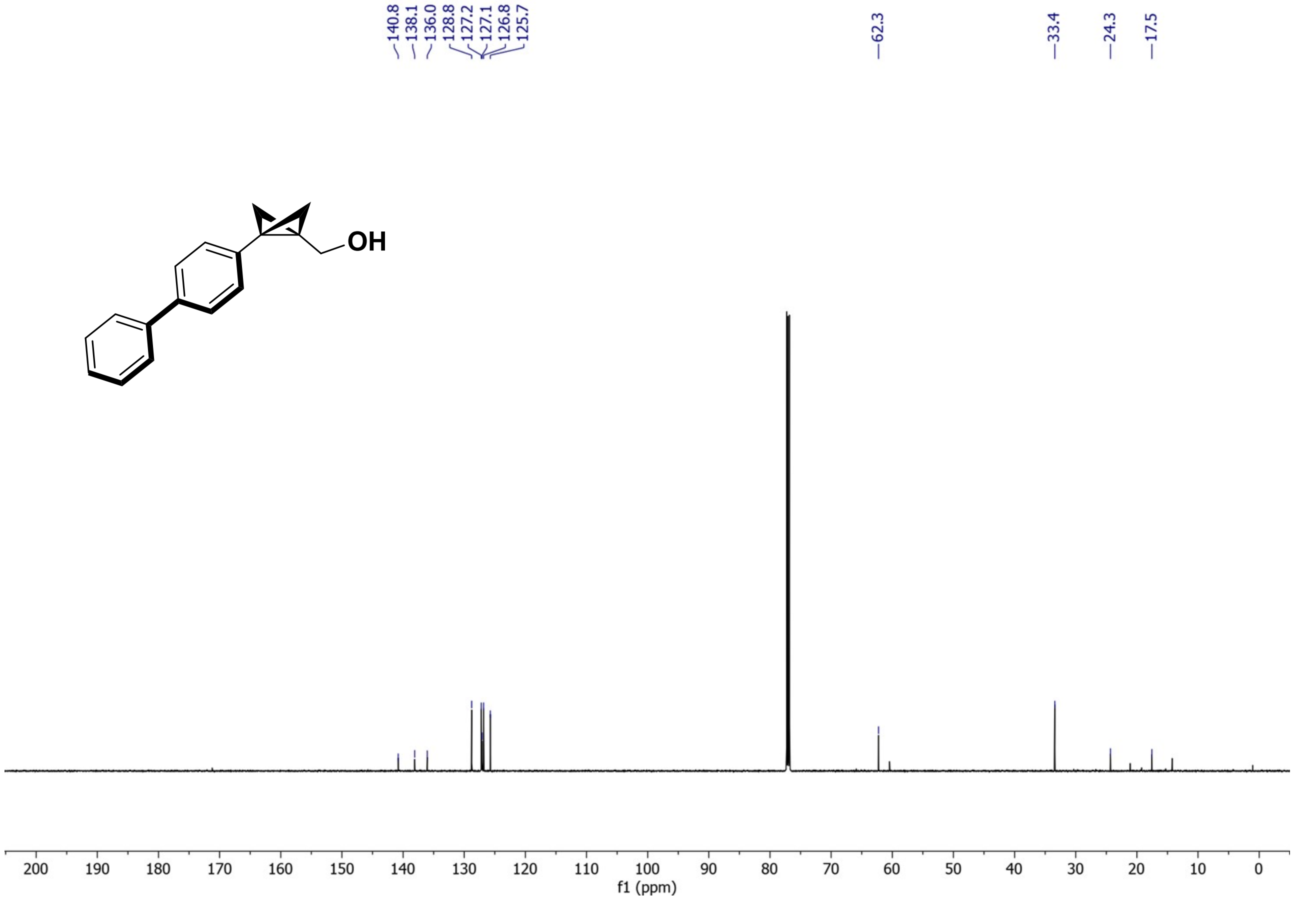

1j ^{1}H NMR (600 MHz, $CDCl_3$)

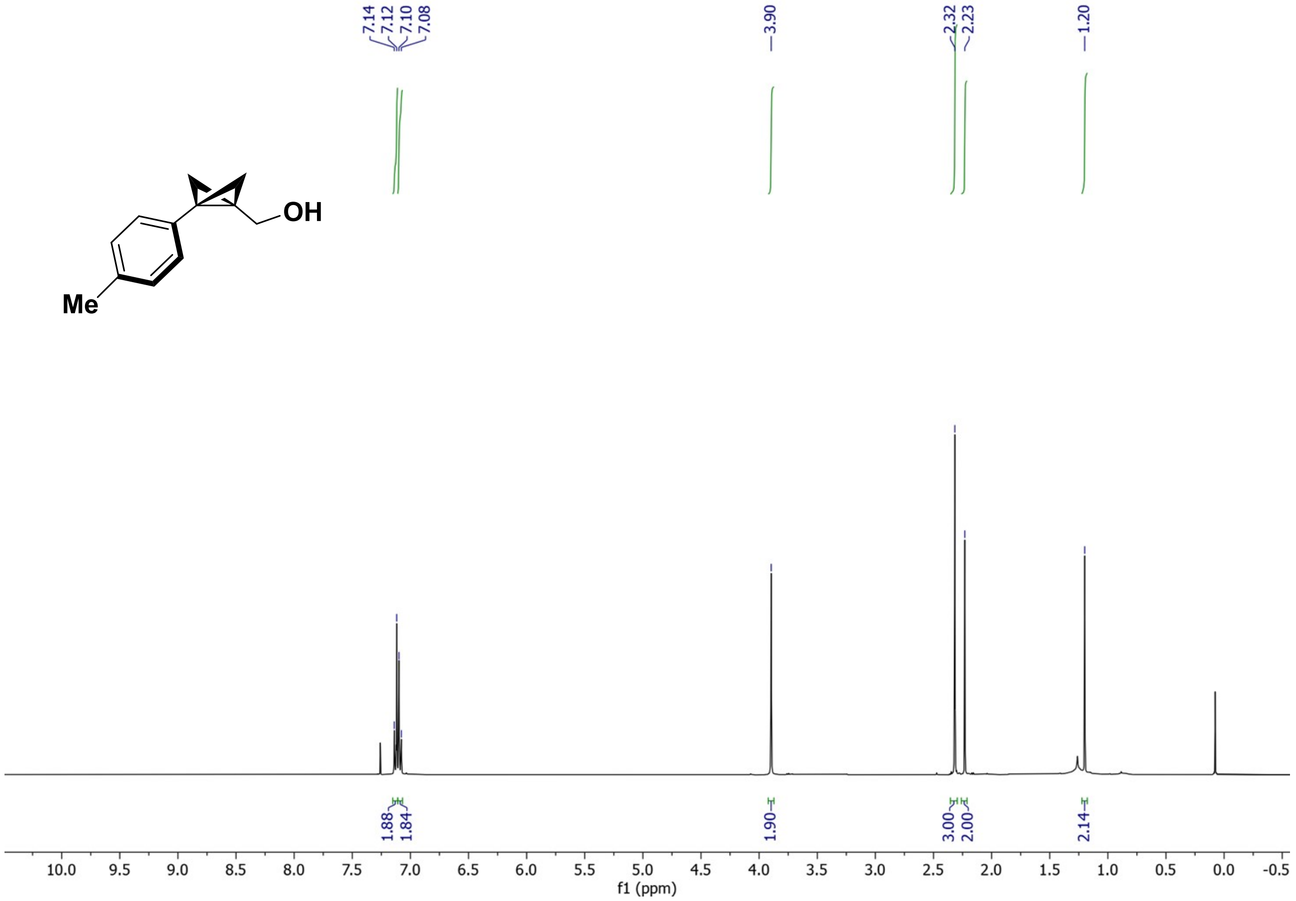

1j ^{13}C NMR (151 MHz, $CDCl_3$)

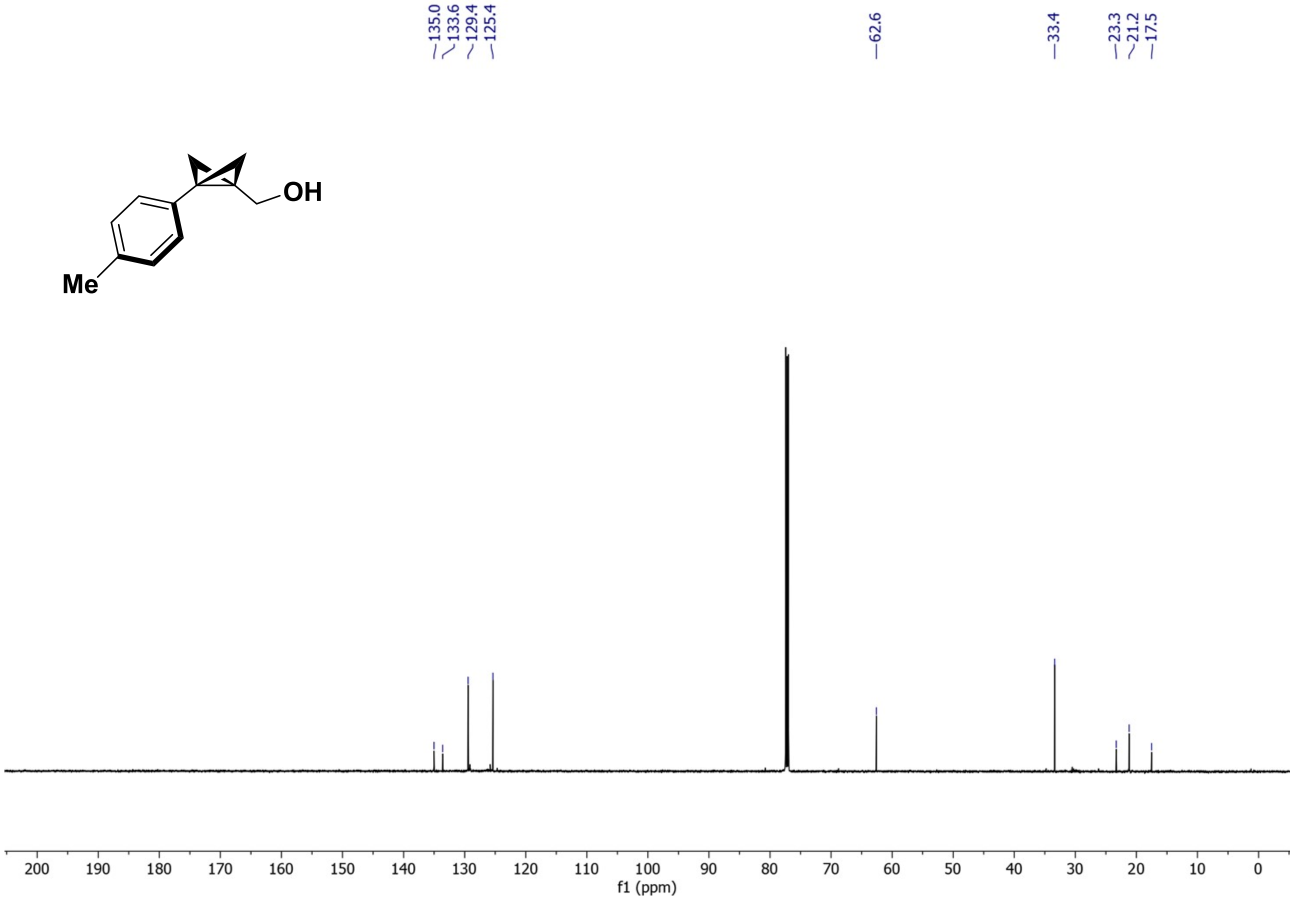

1k ^{1}H NMR (600 MHz, $CDCl_3$)

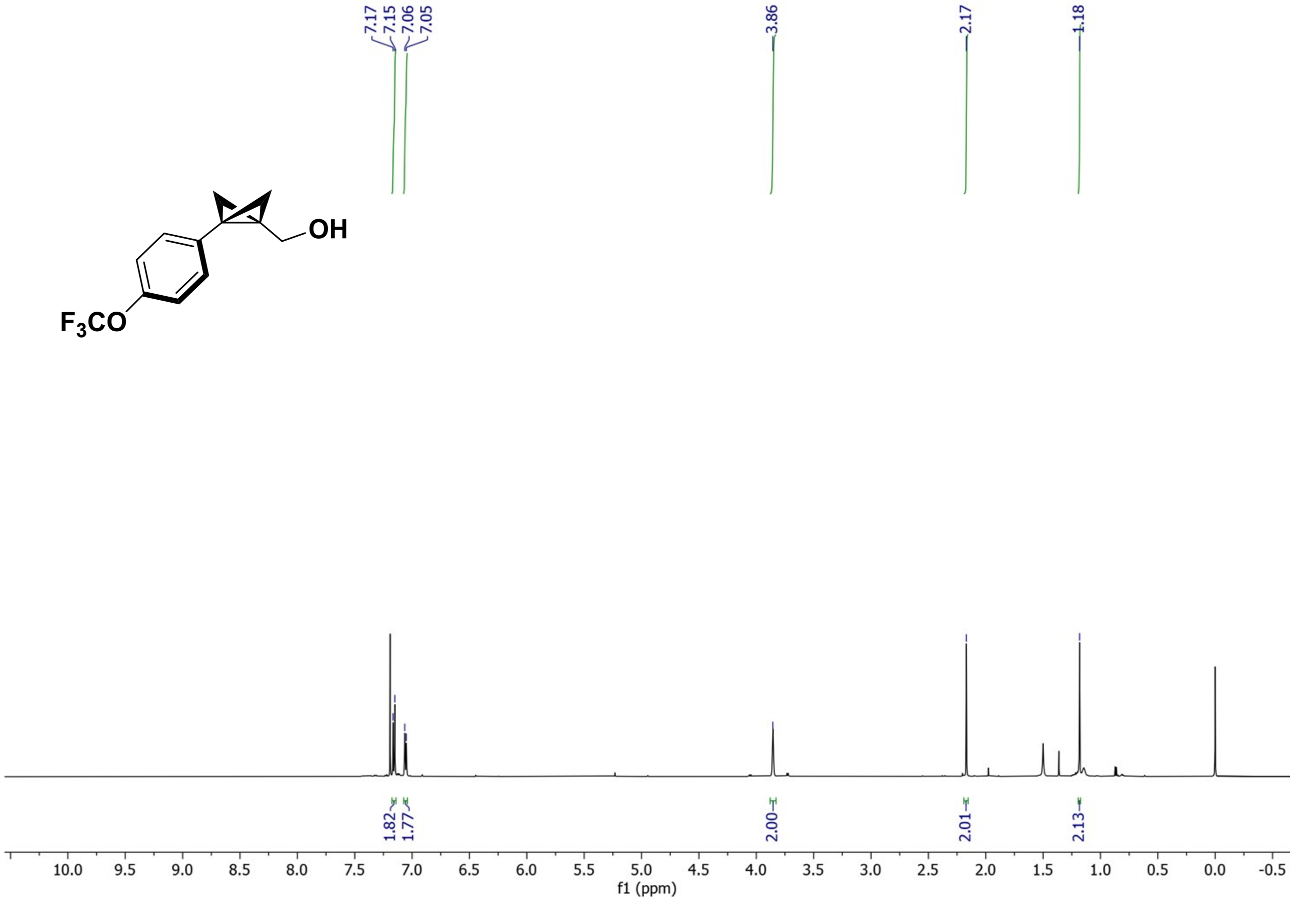

1k ^{13}C NMR (151 MHz, $CDCl_3$)

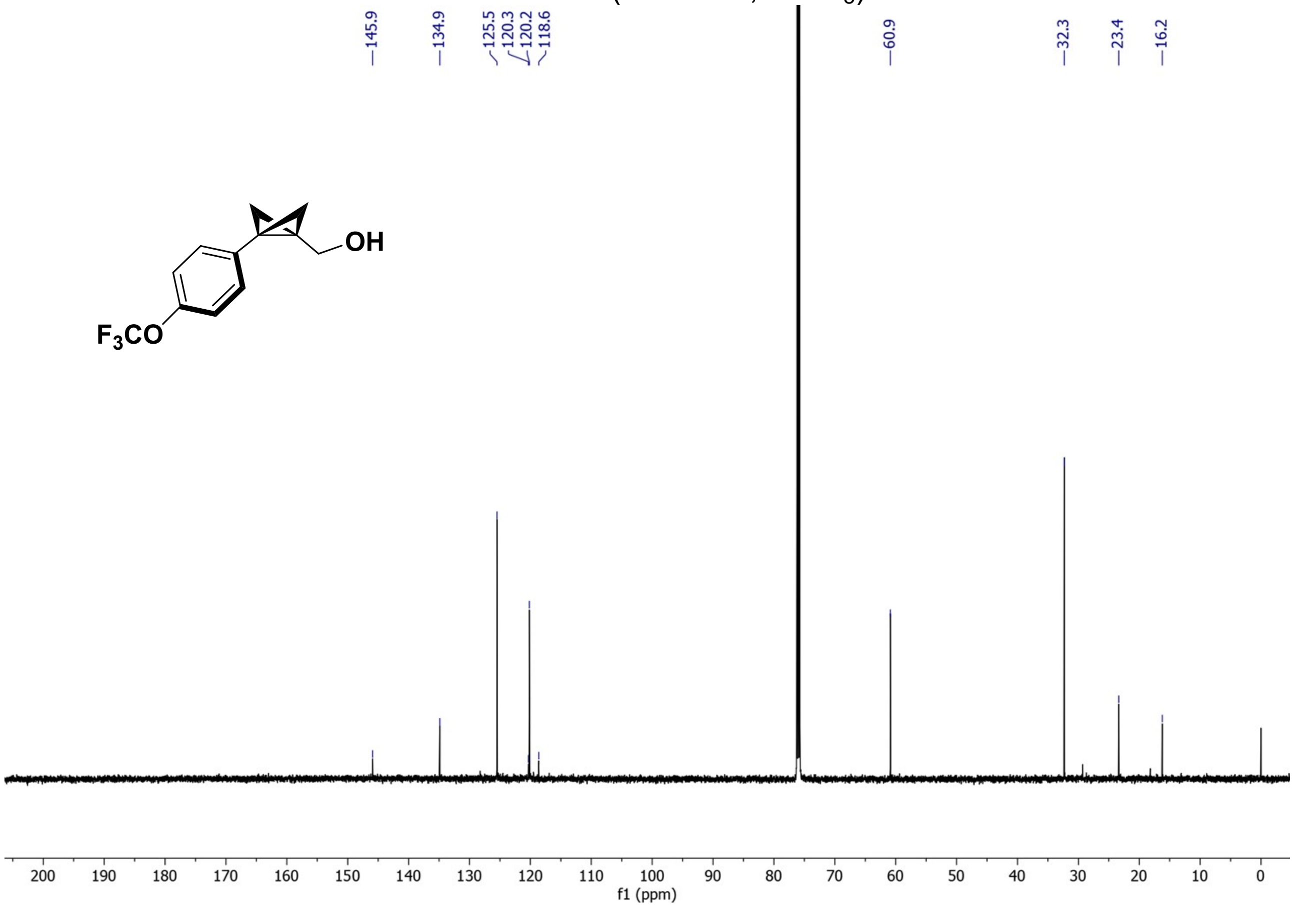

1k ^{19}F NMR (565 MHz, $CDCl_3$)

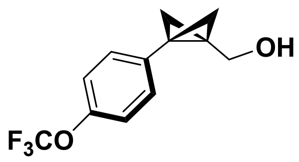

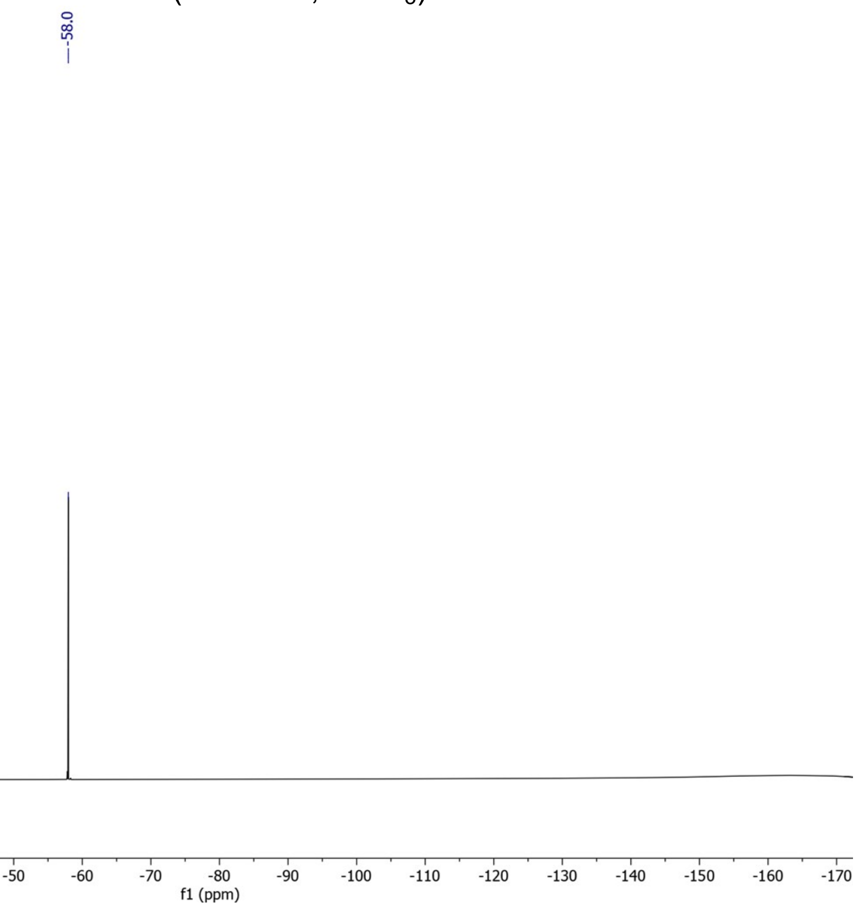

1l ^{1}H NMR (600 MHz, $CDCl_3$)

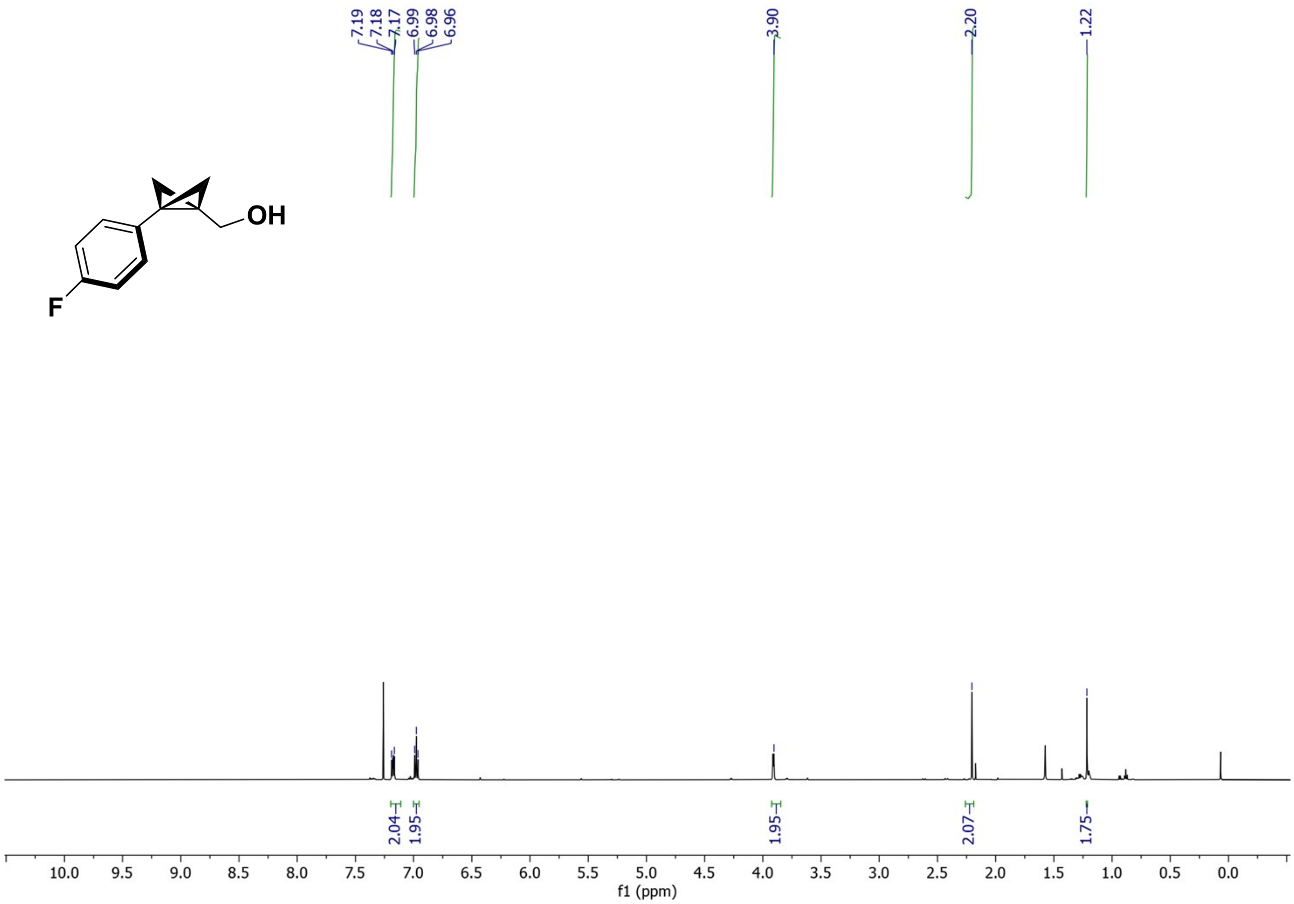

1l ^{13}C NMR (151 MHz, $CDCl_3$)

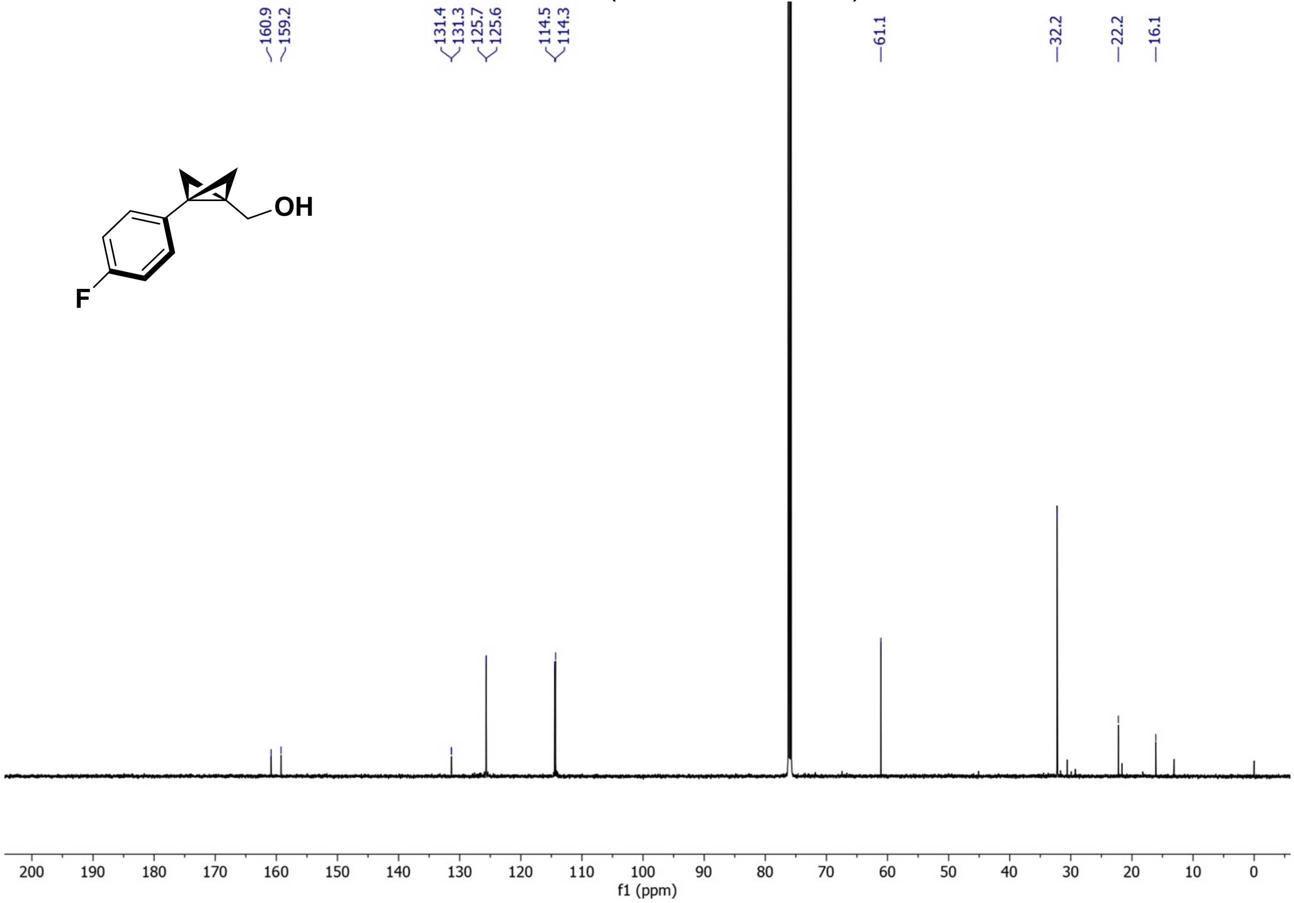

1l ^{19}F NMR (600 MHz, $CDCl_3$)

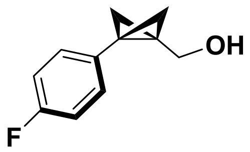

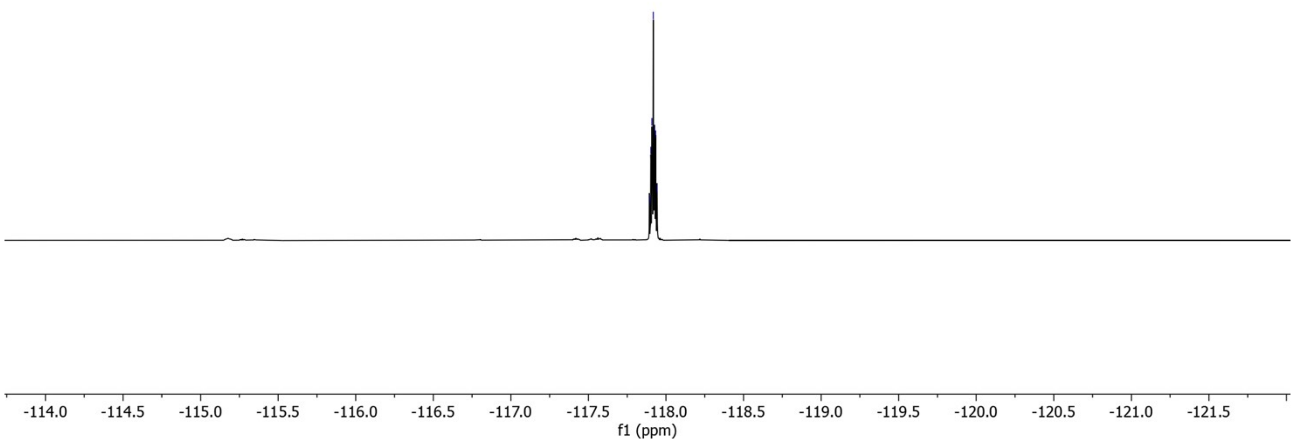

1m ^{1}H NMR (600 MHz, $CDCl_3$)

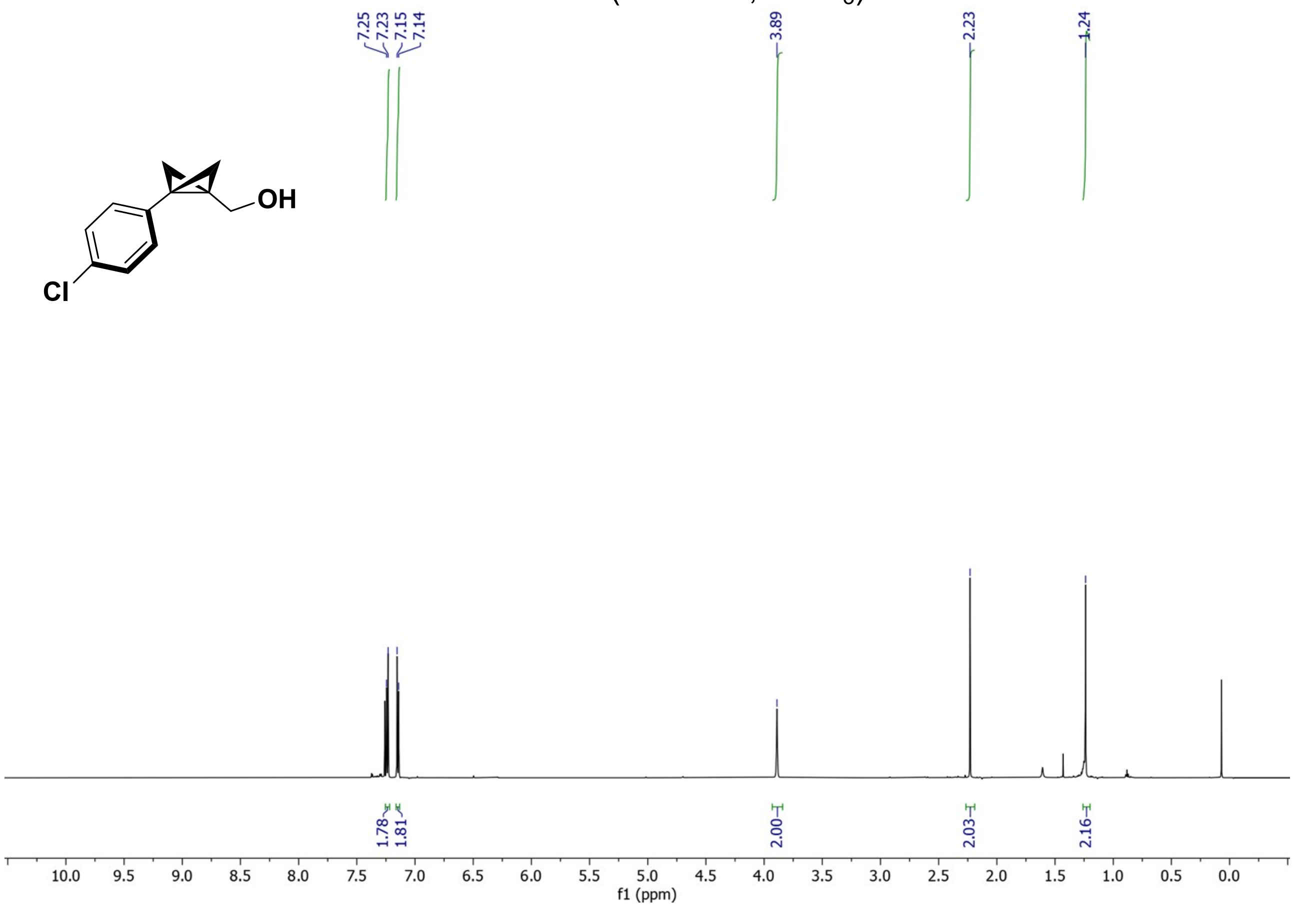

1m ^{13}C NMR (151 MHz, $CDCl_3$)

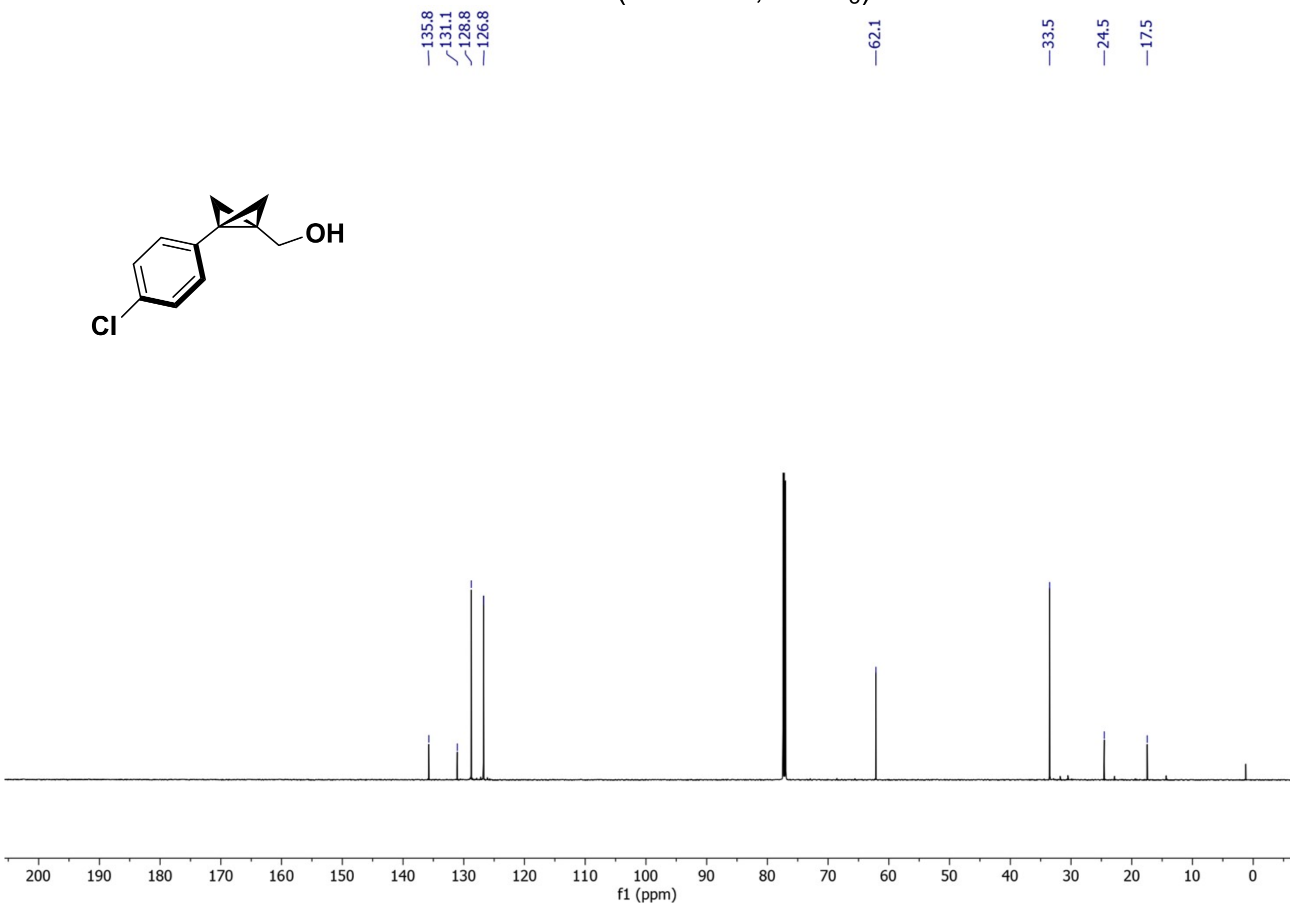

1n ^{1}H NMR (600 MHz, $CDCl_3$)

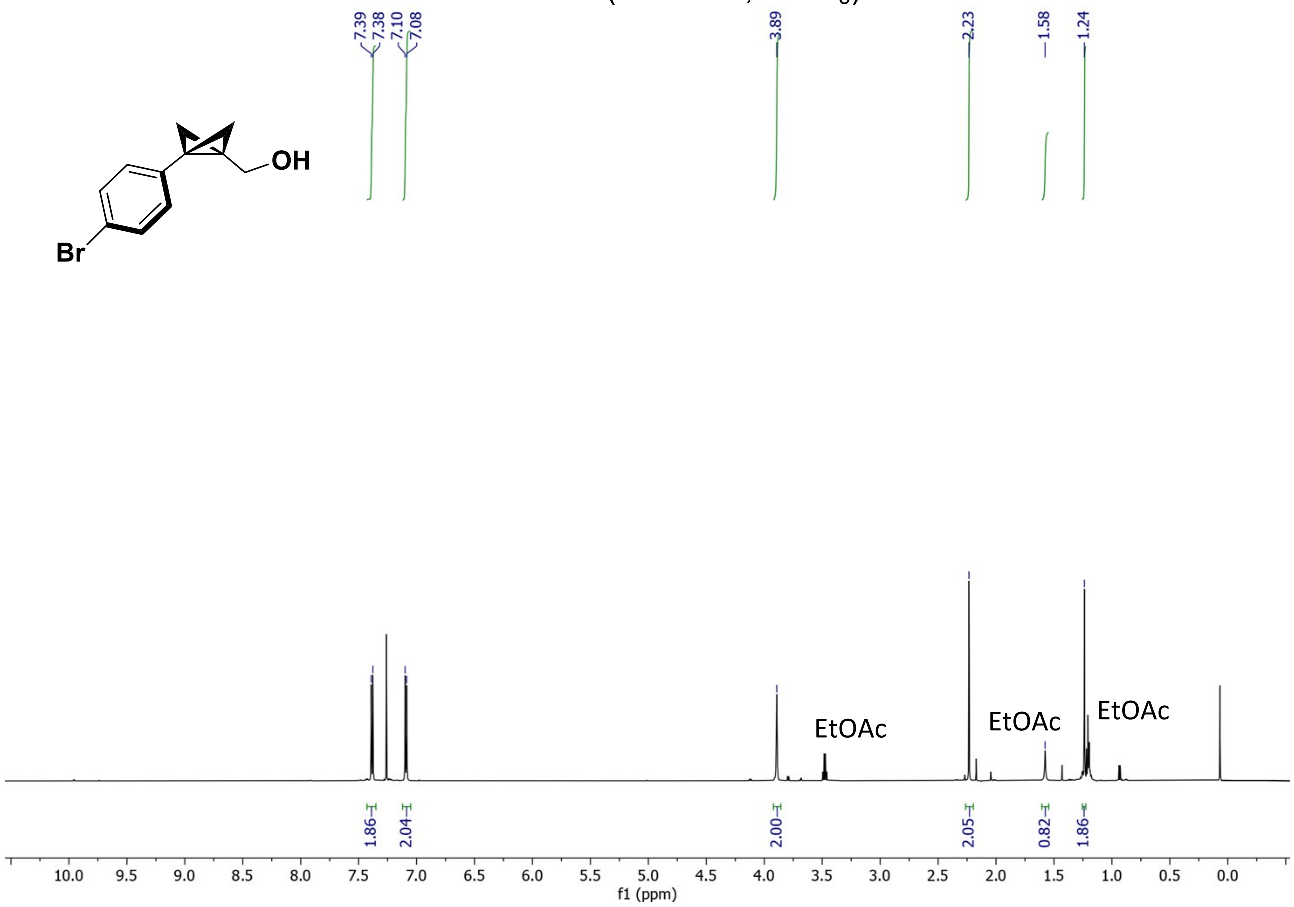

1n ^{13}C NMR (151 MHz, $CDCl_3$)

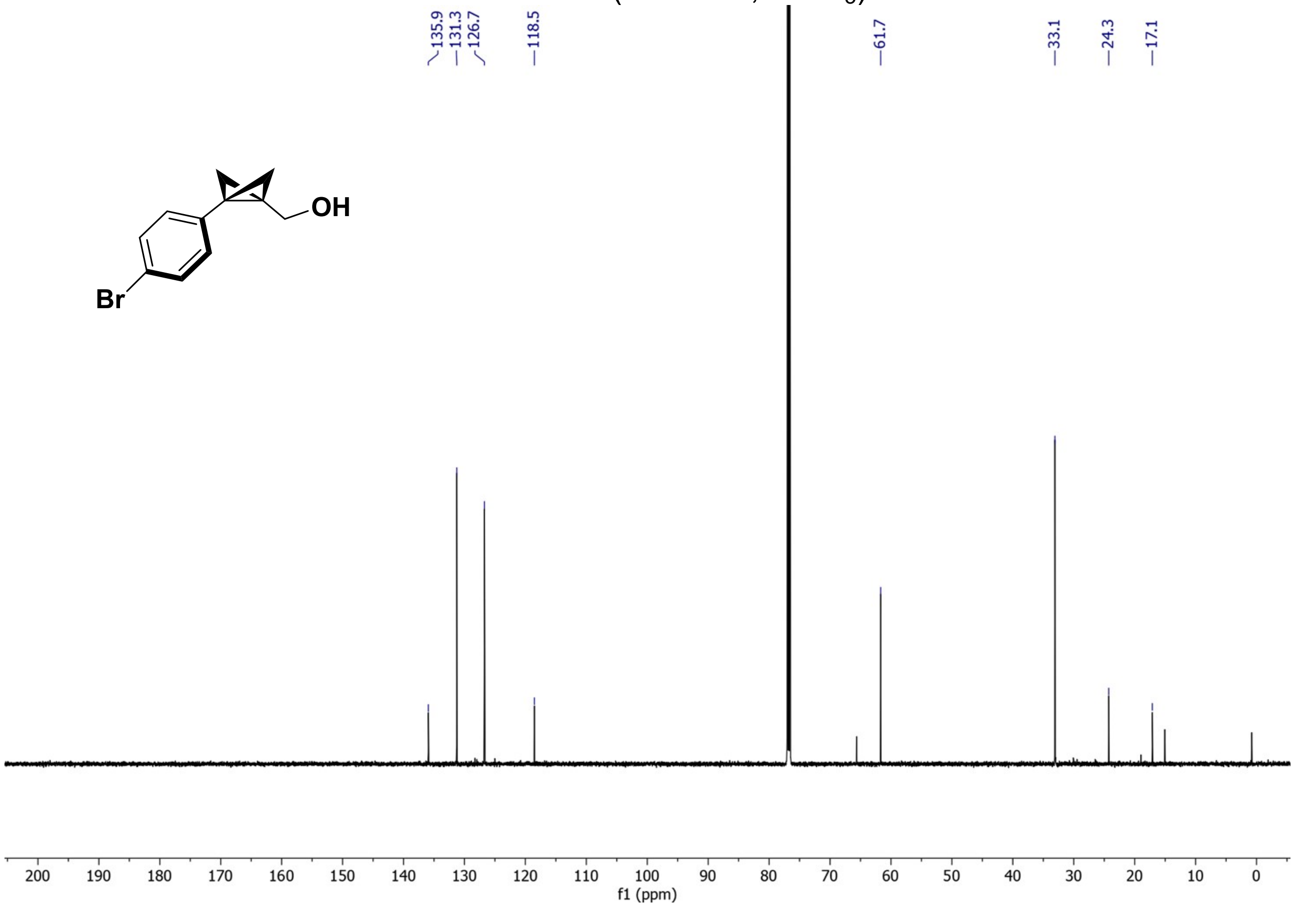

1o ^{1}H NMR (600 MHz, $CDCl_3$)

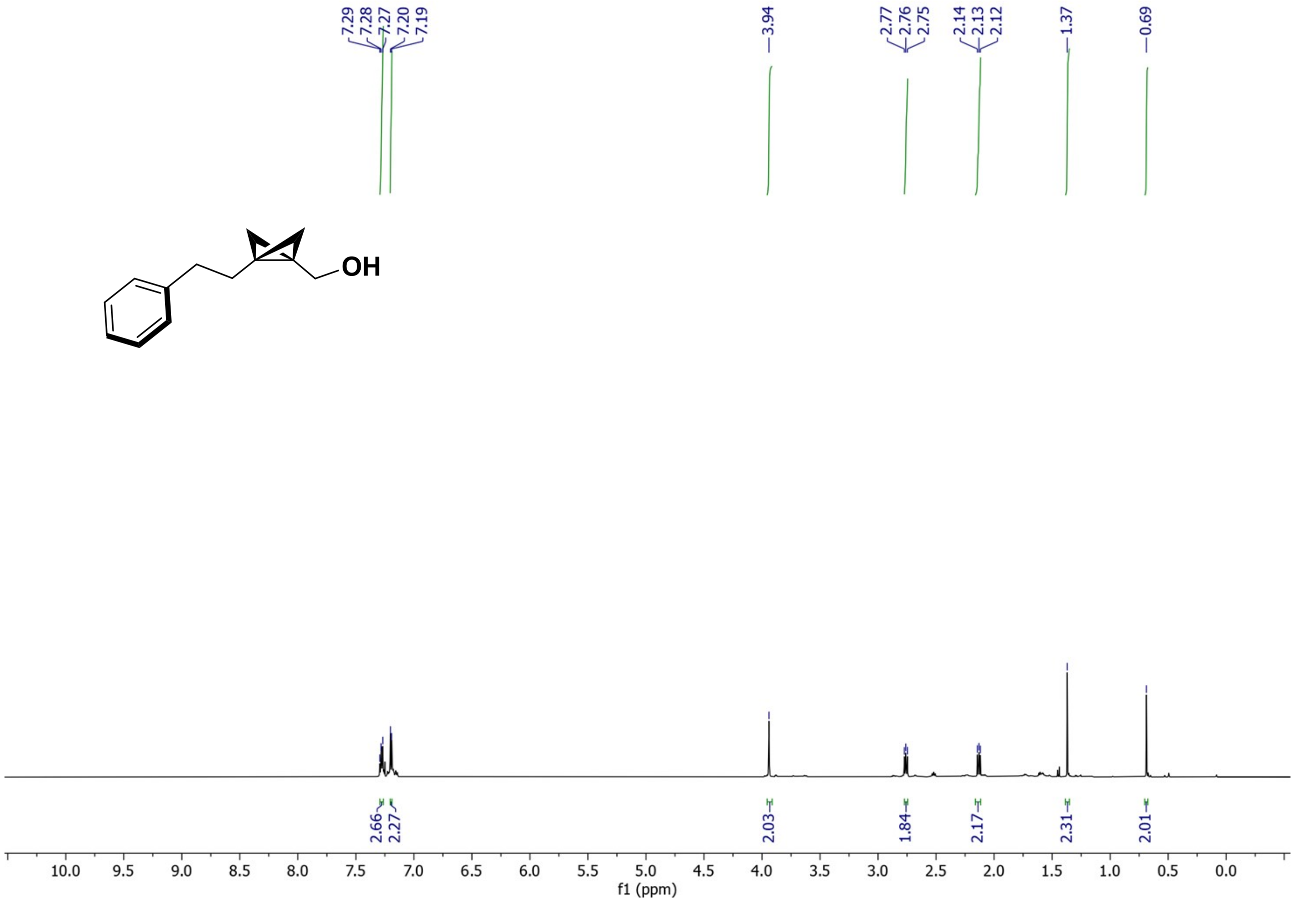

1o ^{13}C NMR (151 MHz, $CDCl_3$)

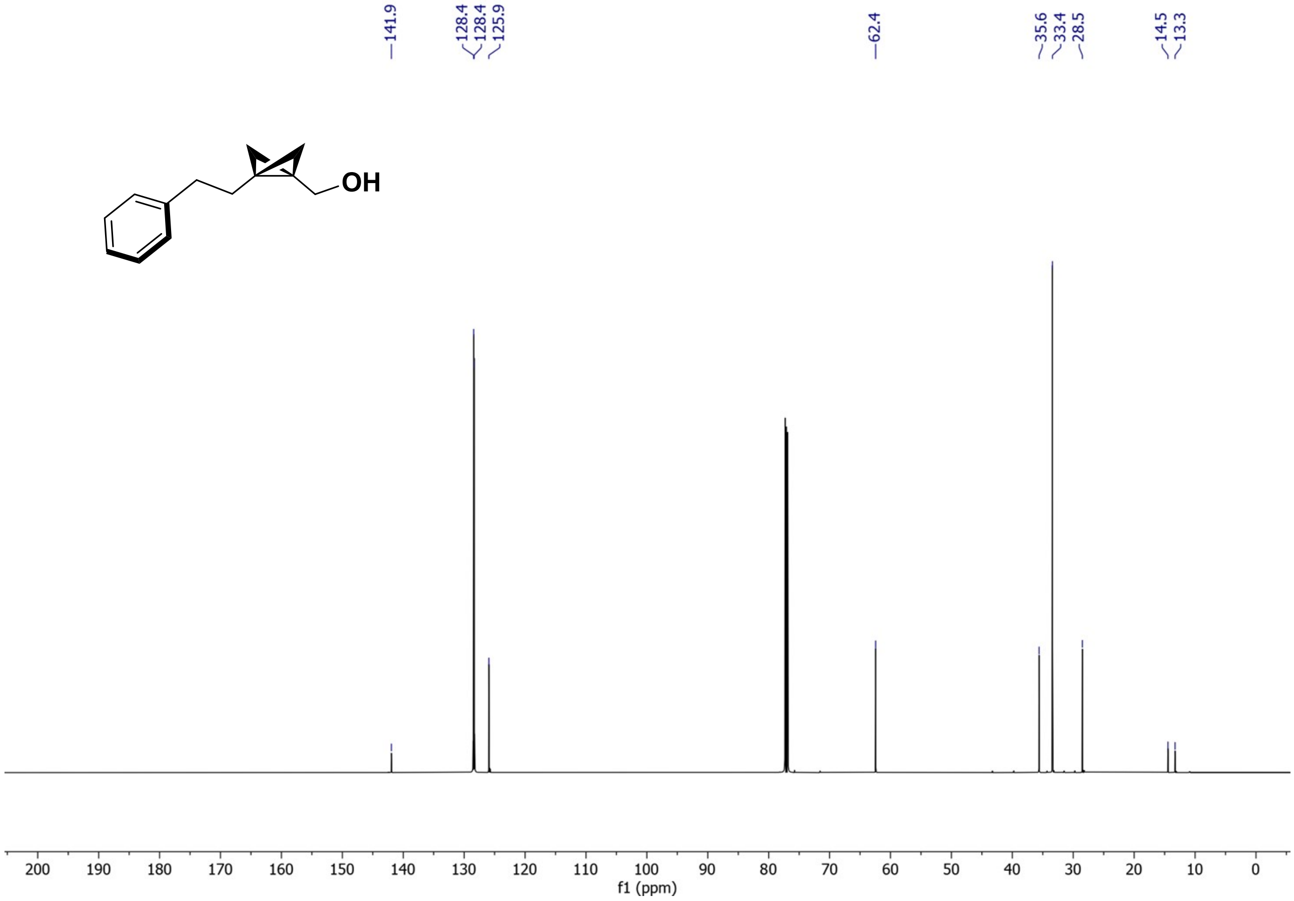

1p ^{1}H NMR (600 MHz, $CDCl_3$)

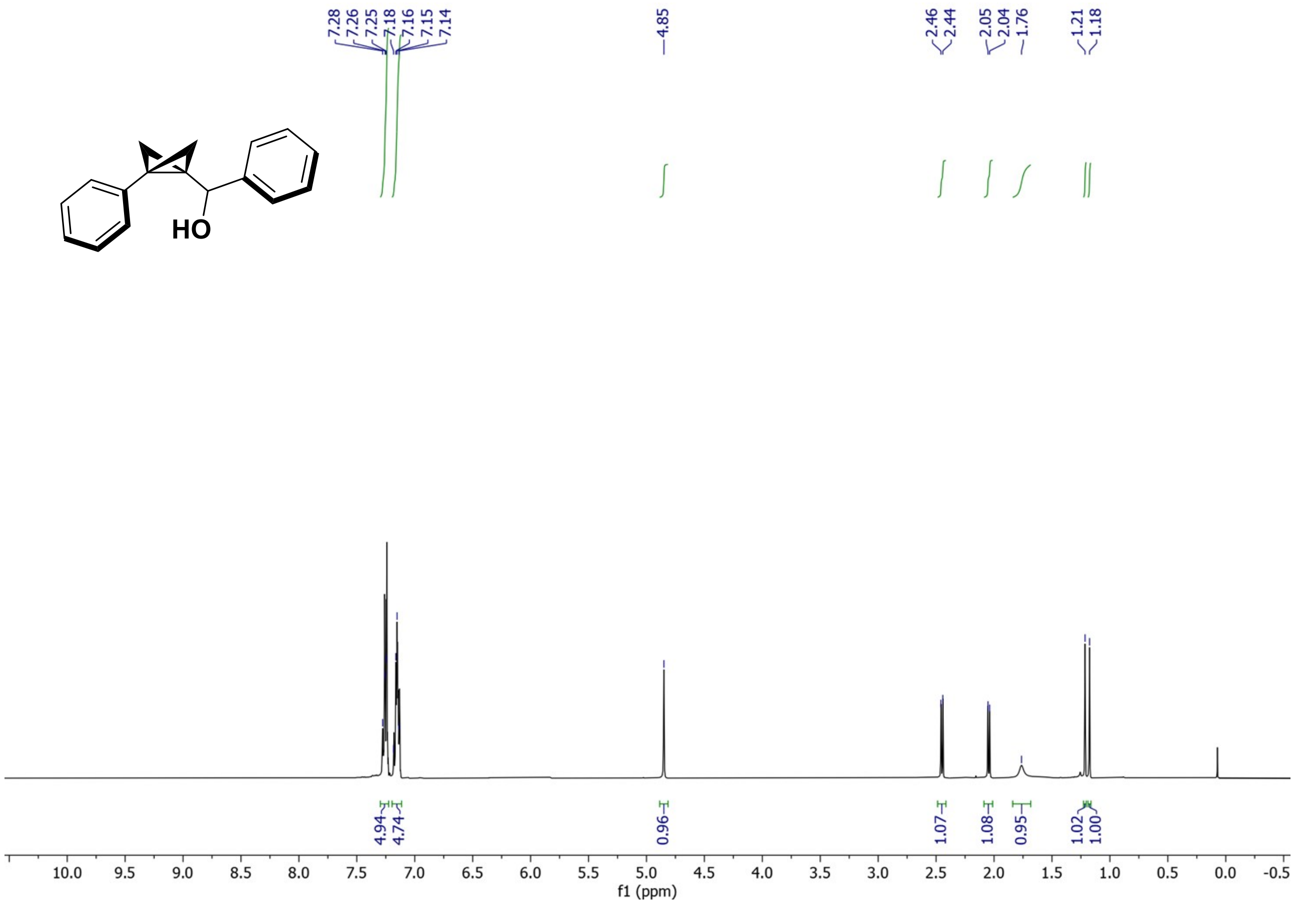

1p ^{13}C NMR (151 MHz, $CDCl_3$)

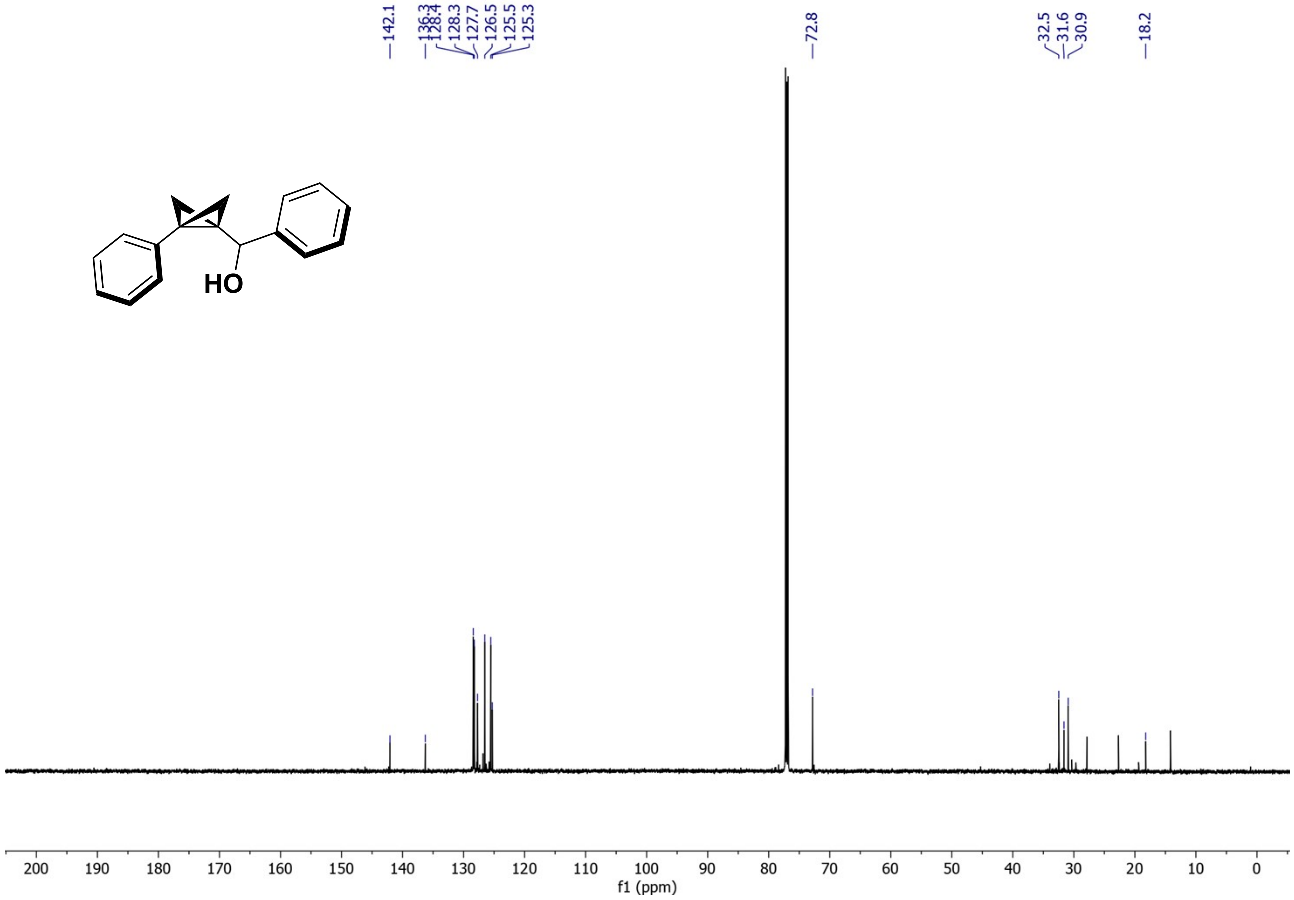

1q ^{1}H NMR (600 MHz, $CDCl_3$)

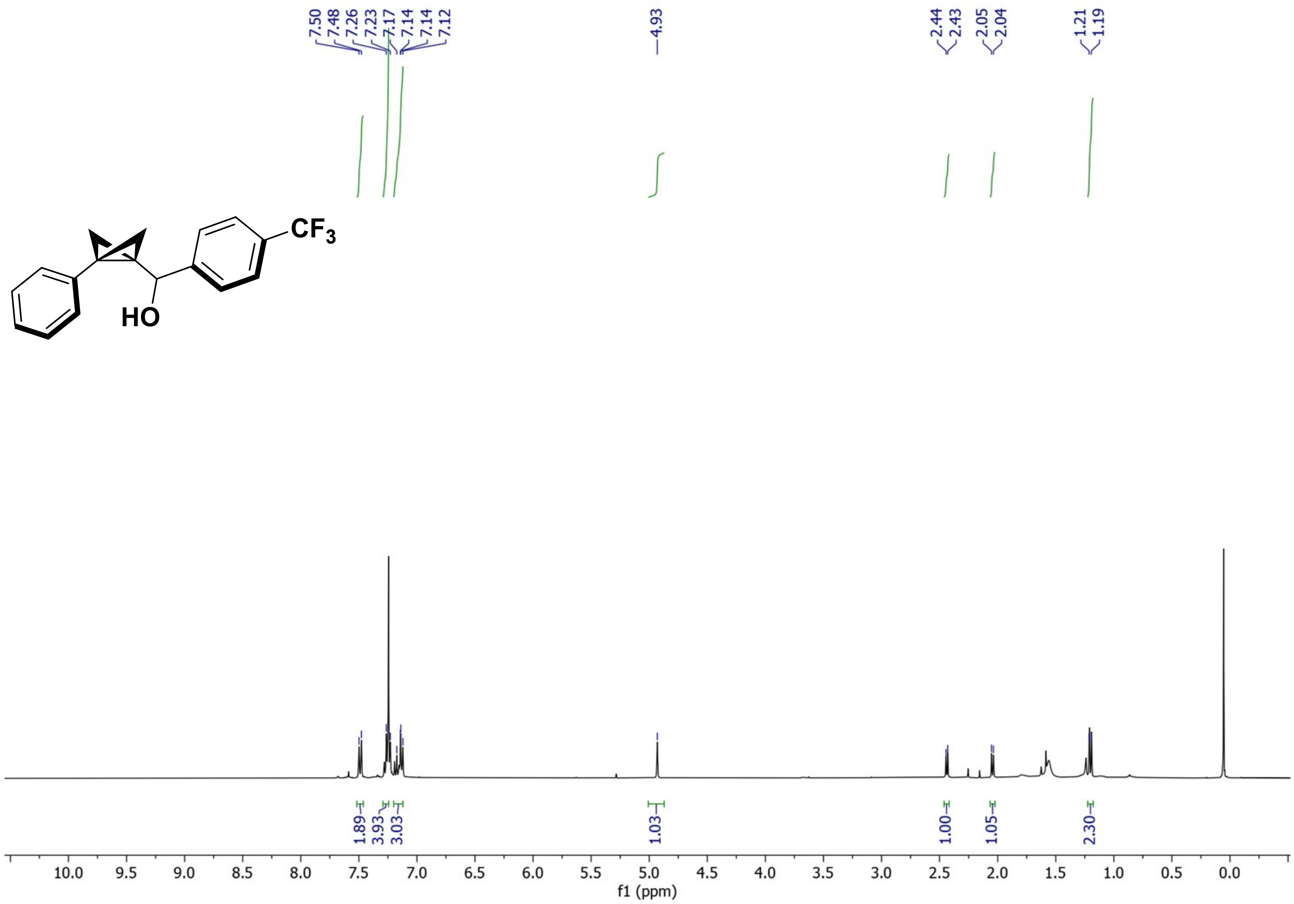

1q ^{13}C NMR (151 MHz, $CDCl_3$)

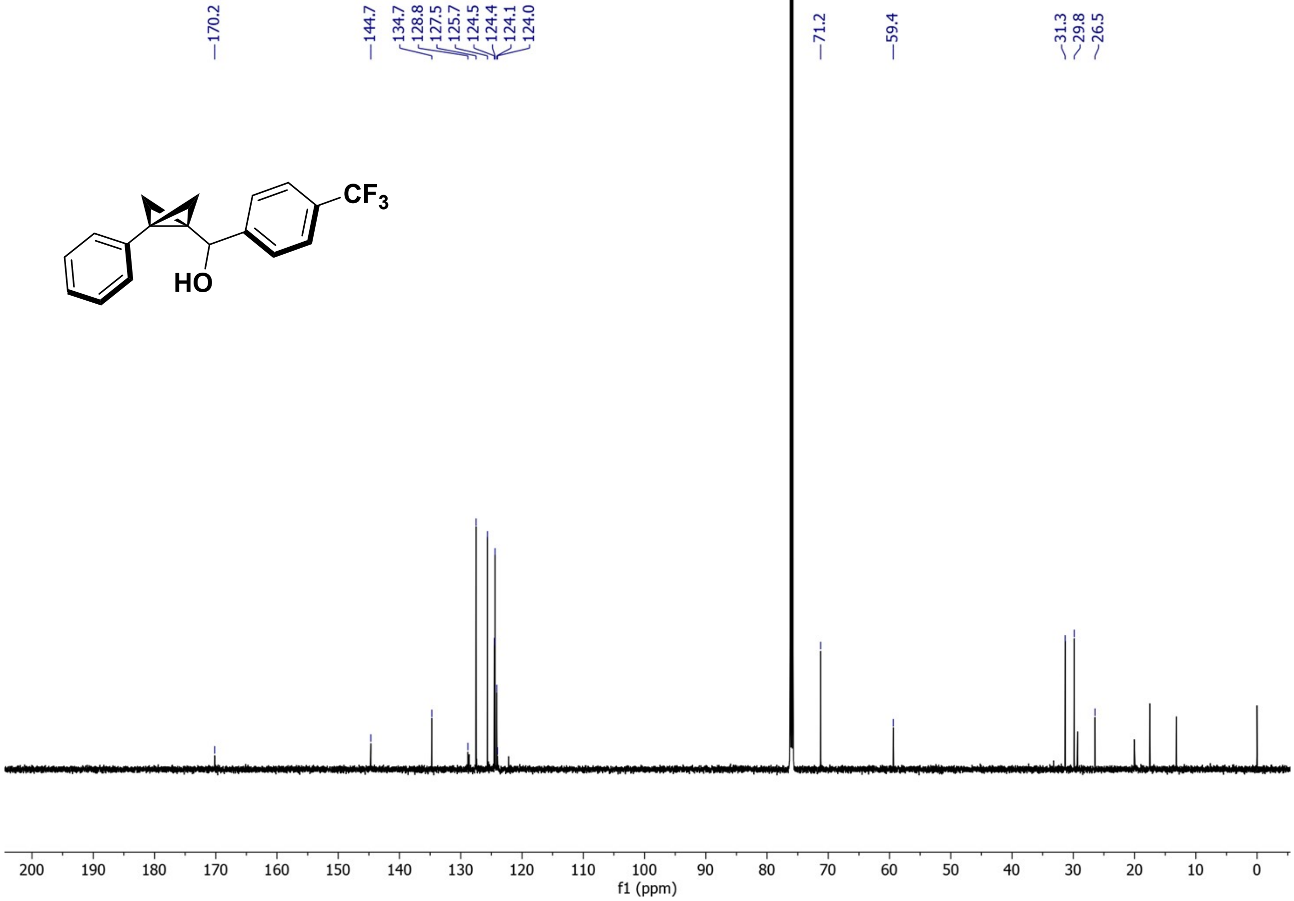

1q ^{19}F NMR (565 MHz, $CDCl_3$)

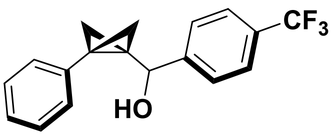

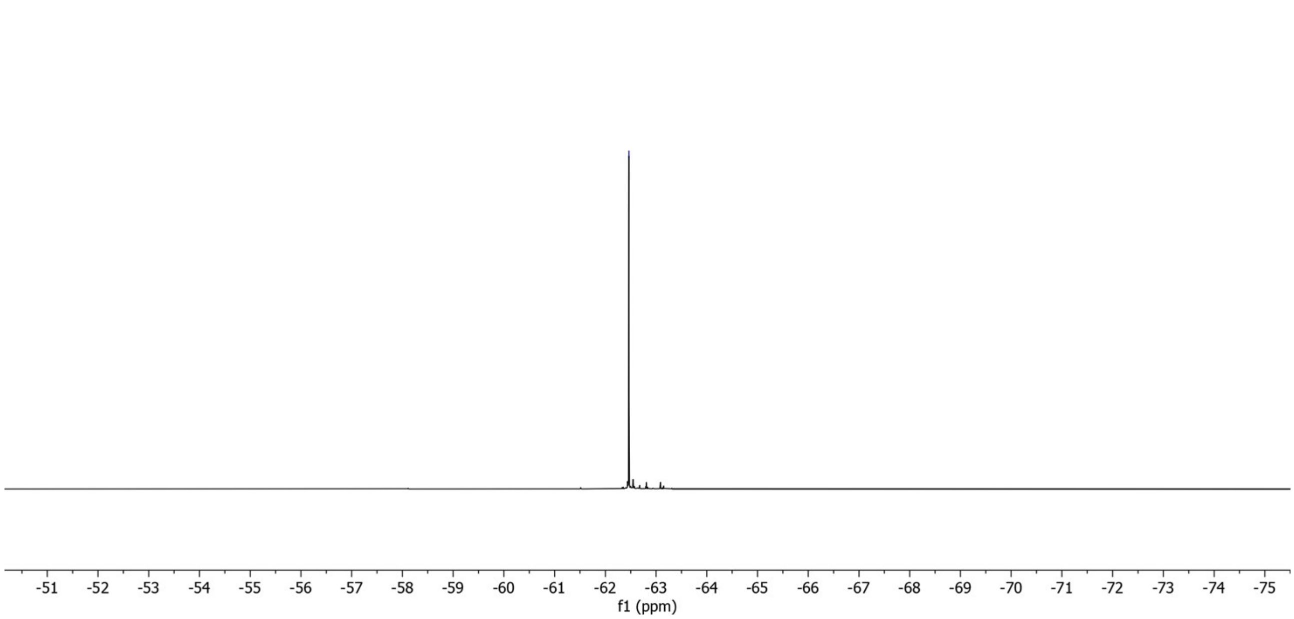

1r ^{1}**H NMR** (600 MHz, $CDCl_3$)

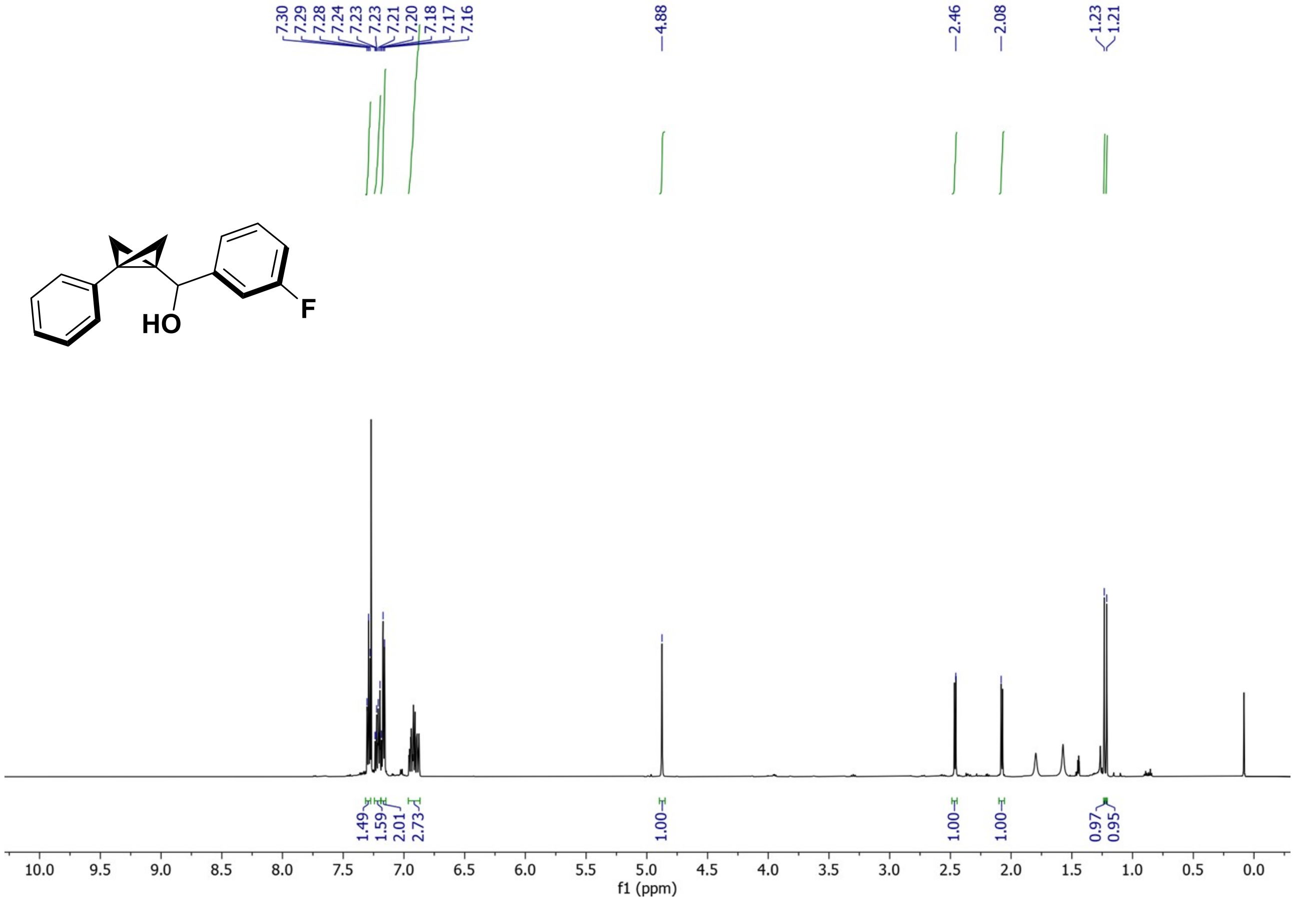

1r ^{13}**C NMR** (151 MHz, $CDCl_3$)

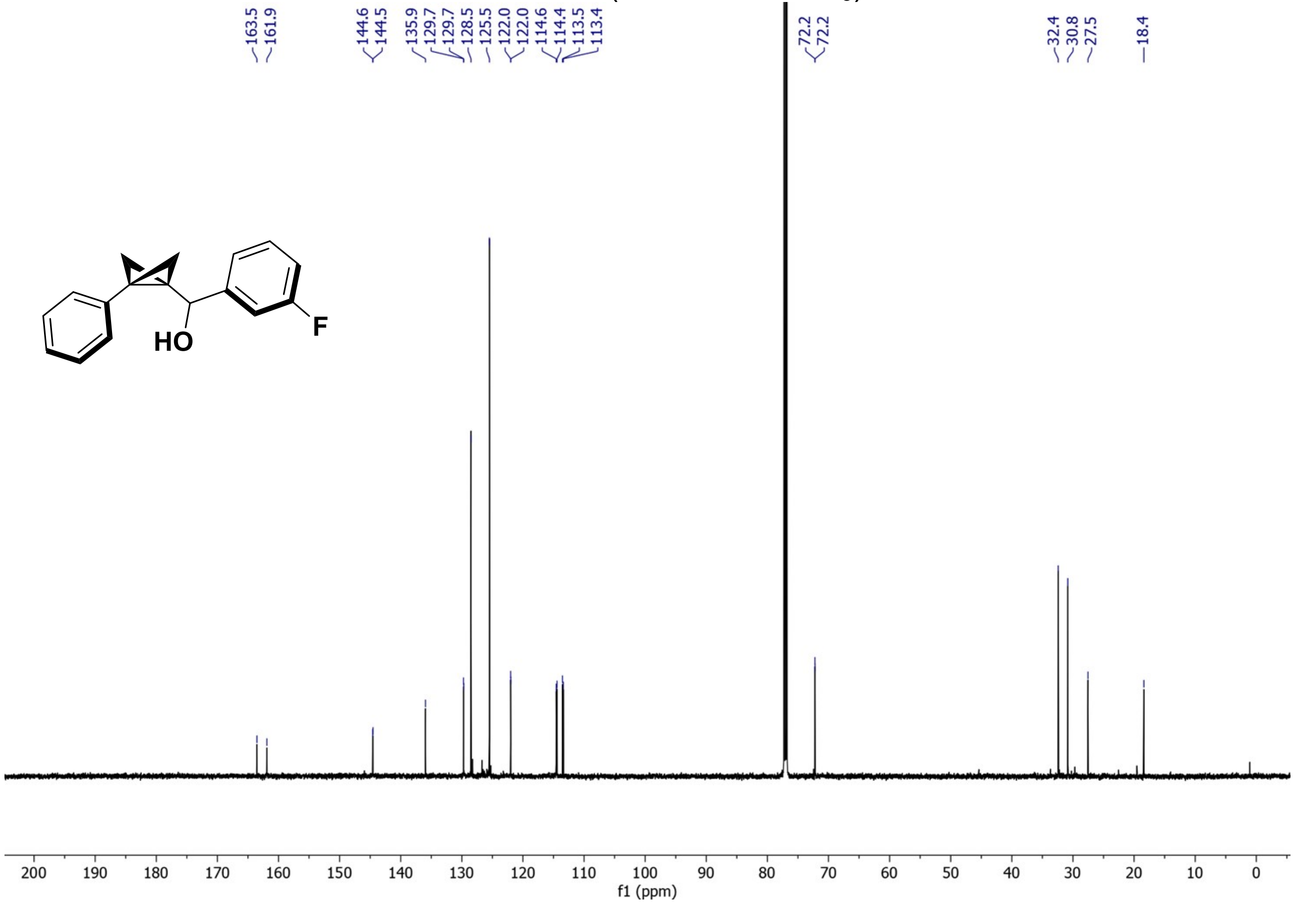

1r ^{19}F NMR (565 MHz, $CDCl_3$)

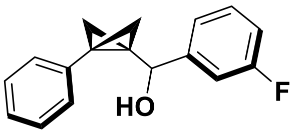

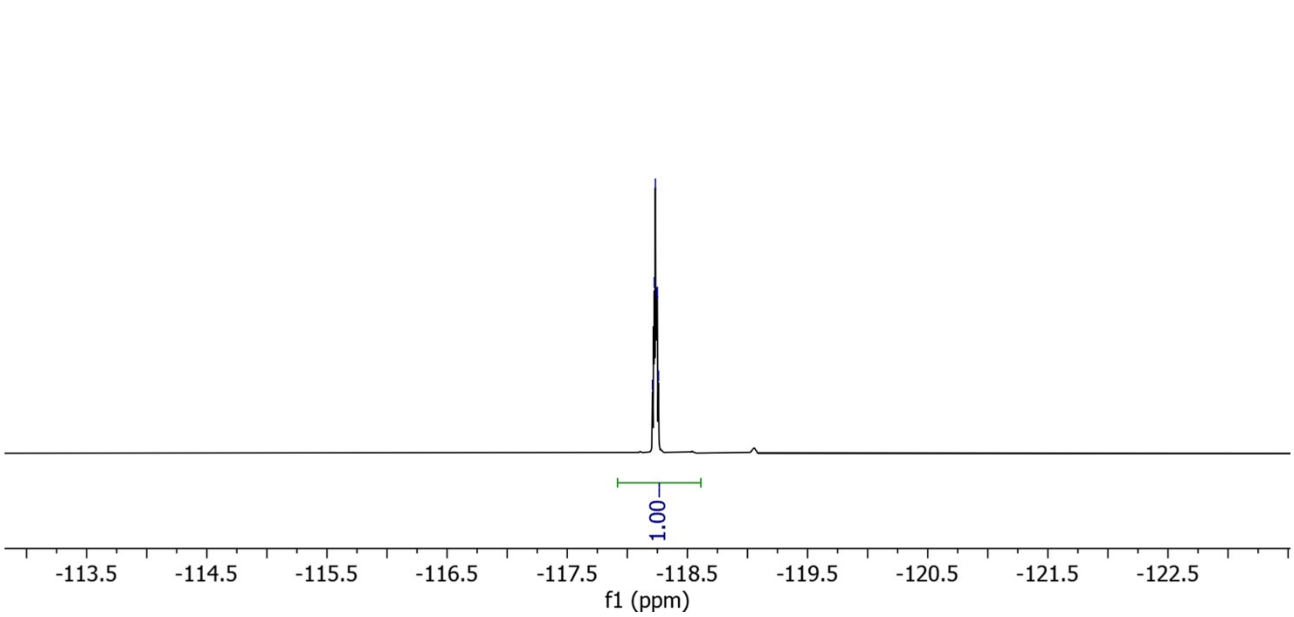

1s ^{1}H NMR (600 MHz, $CDCl_3$)

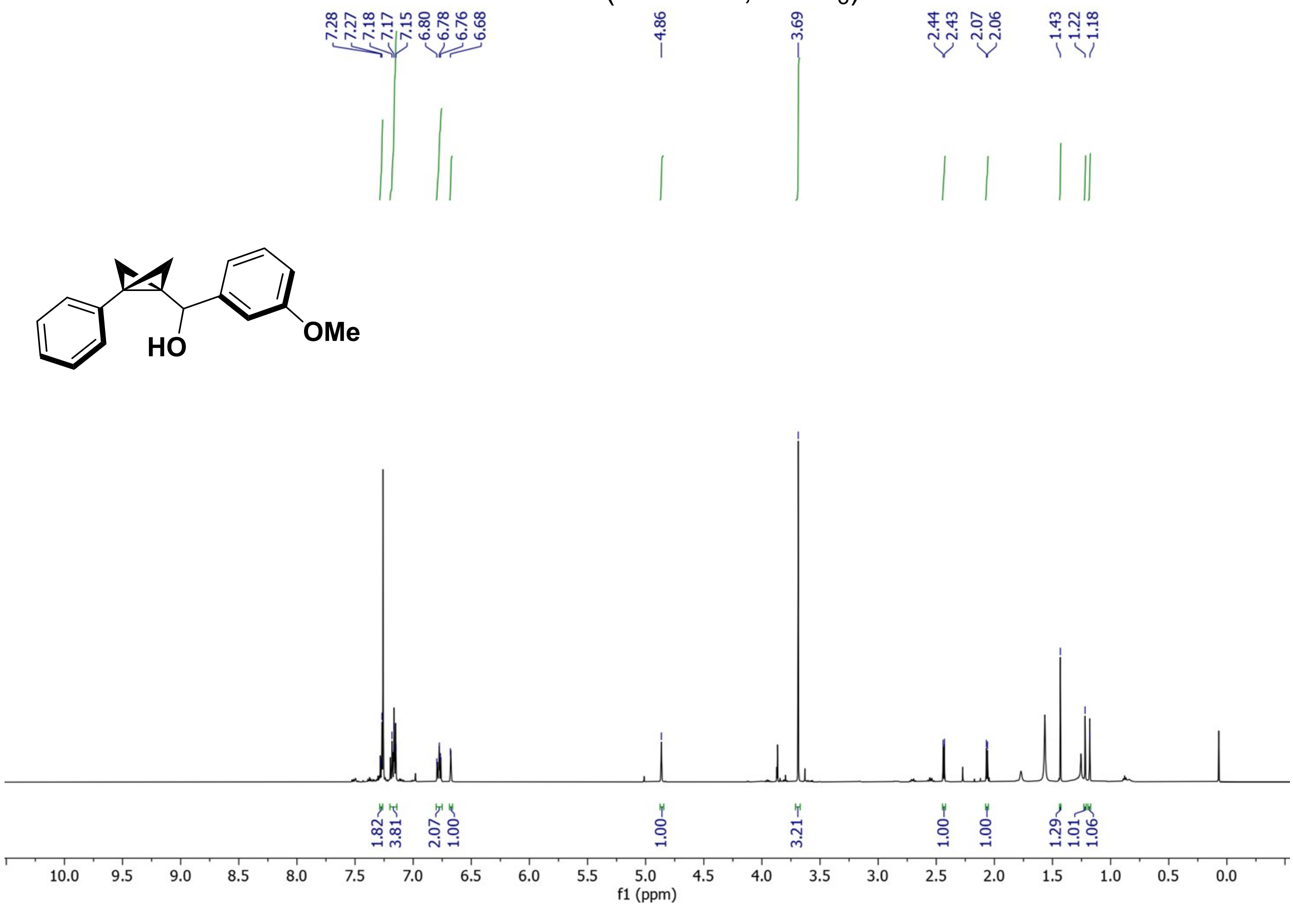

1s ^{13}C NMR (151 MHz, $CDCl_3$)

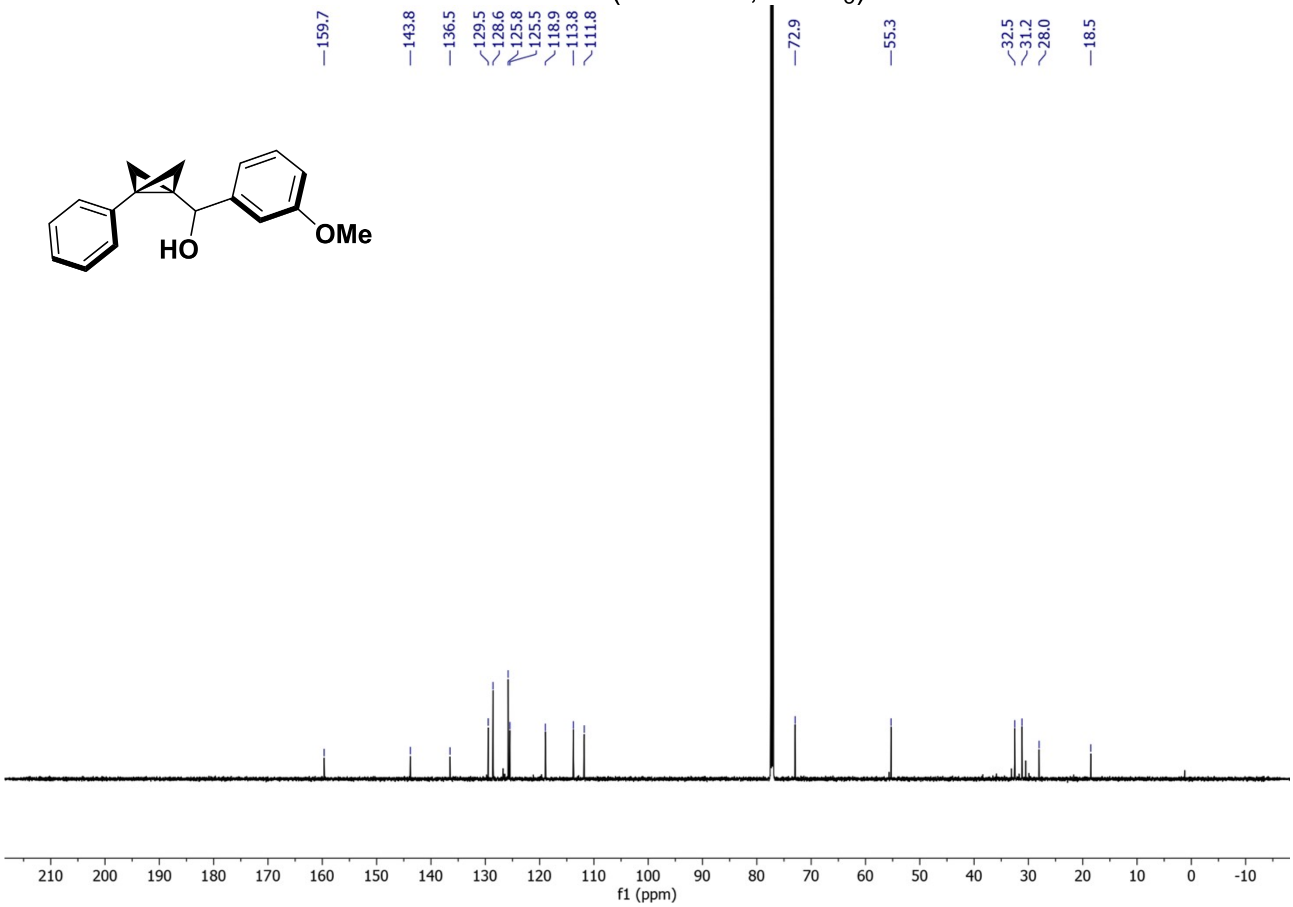

1u ^{1}H NMR (600 MHz, $CDCl_3$)

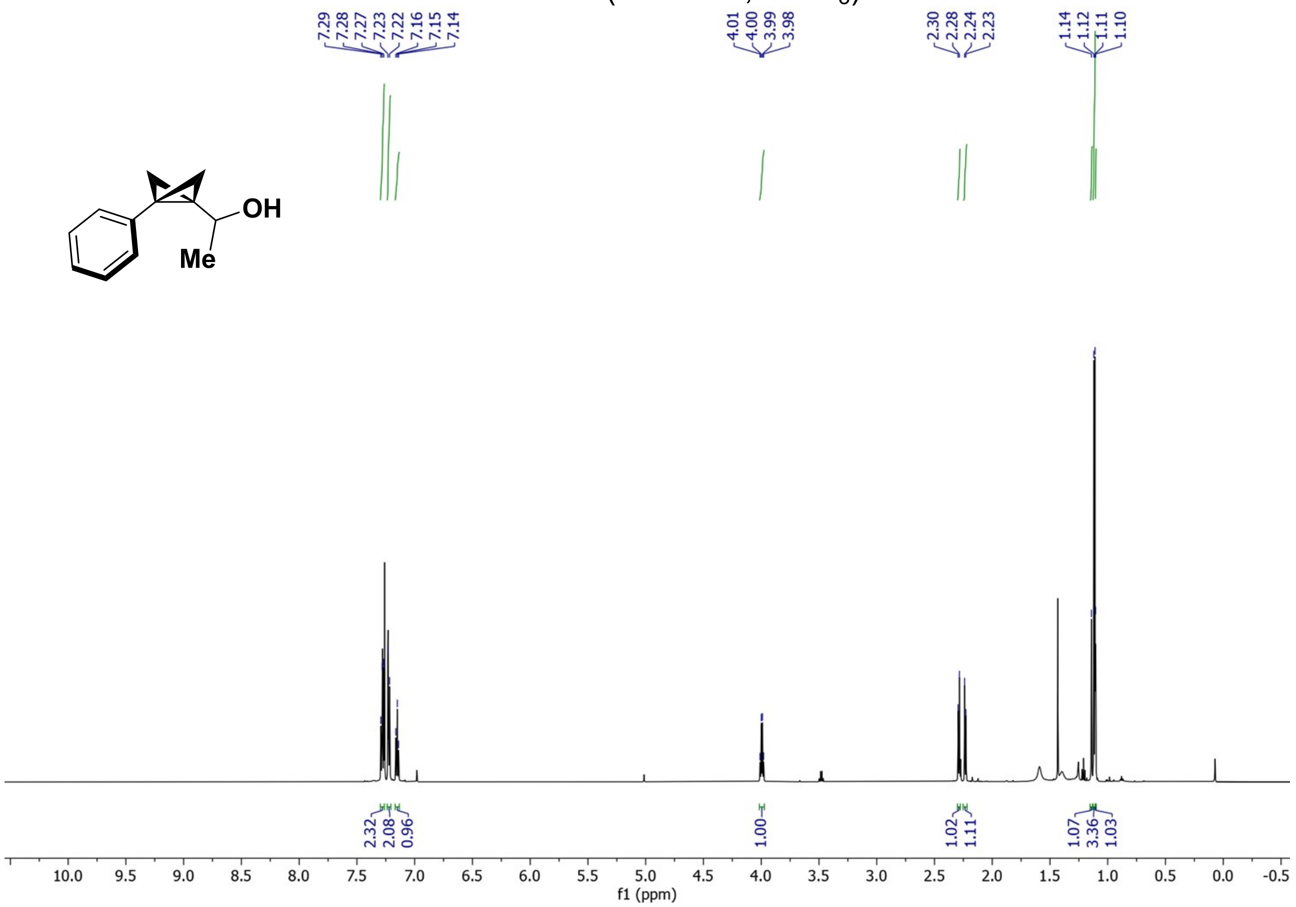

1u ^{13}C NMR (151 MHz, $CDCl_3$)

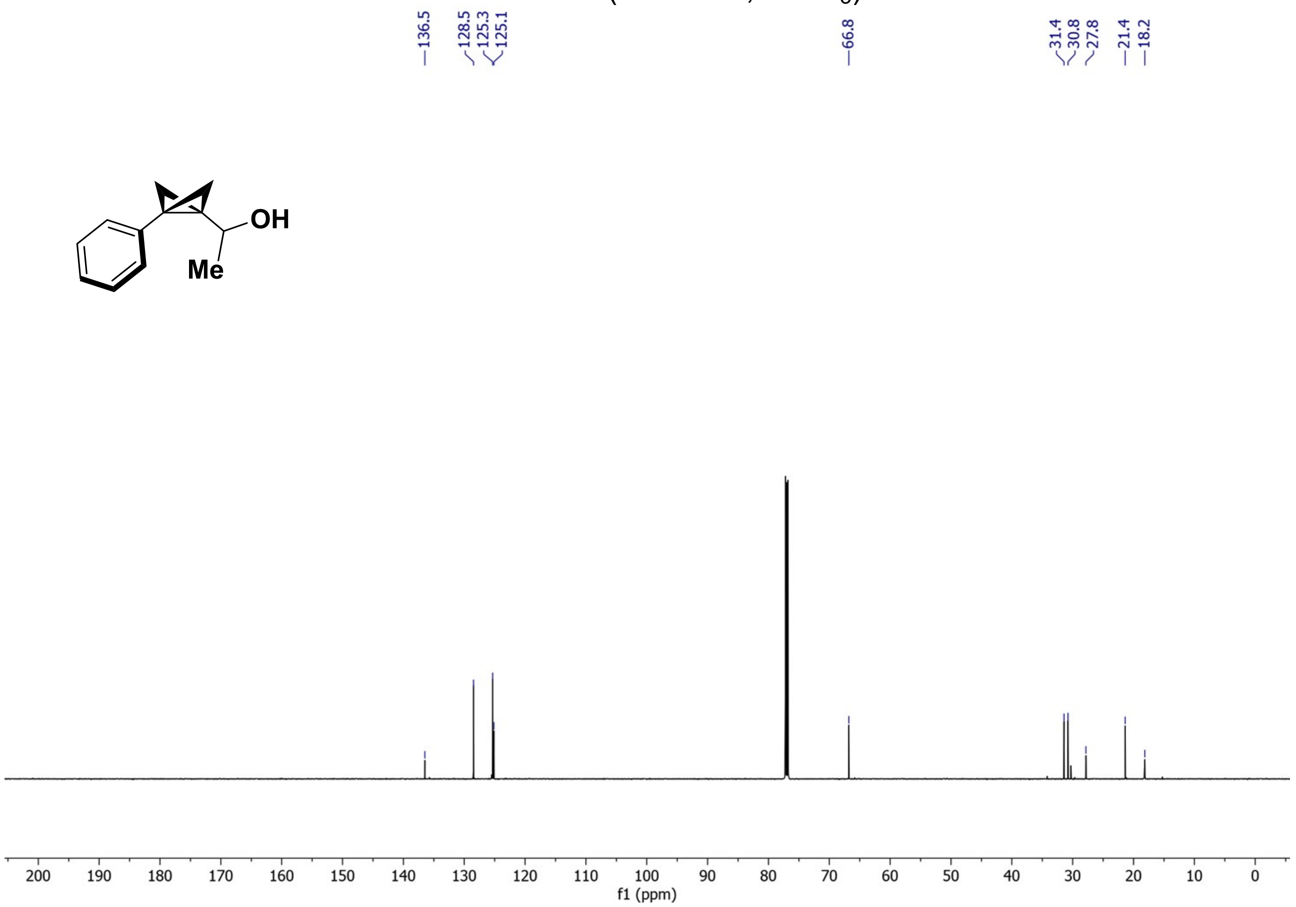

1v ^{1}H NMR (600 MHz, $CDCl_3$)

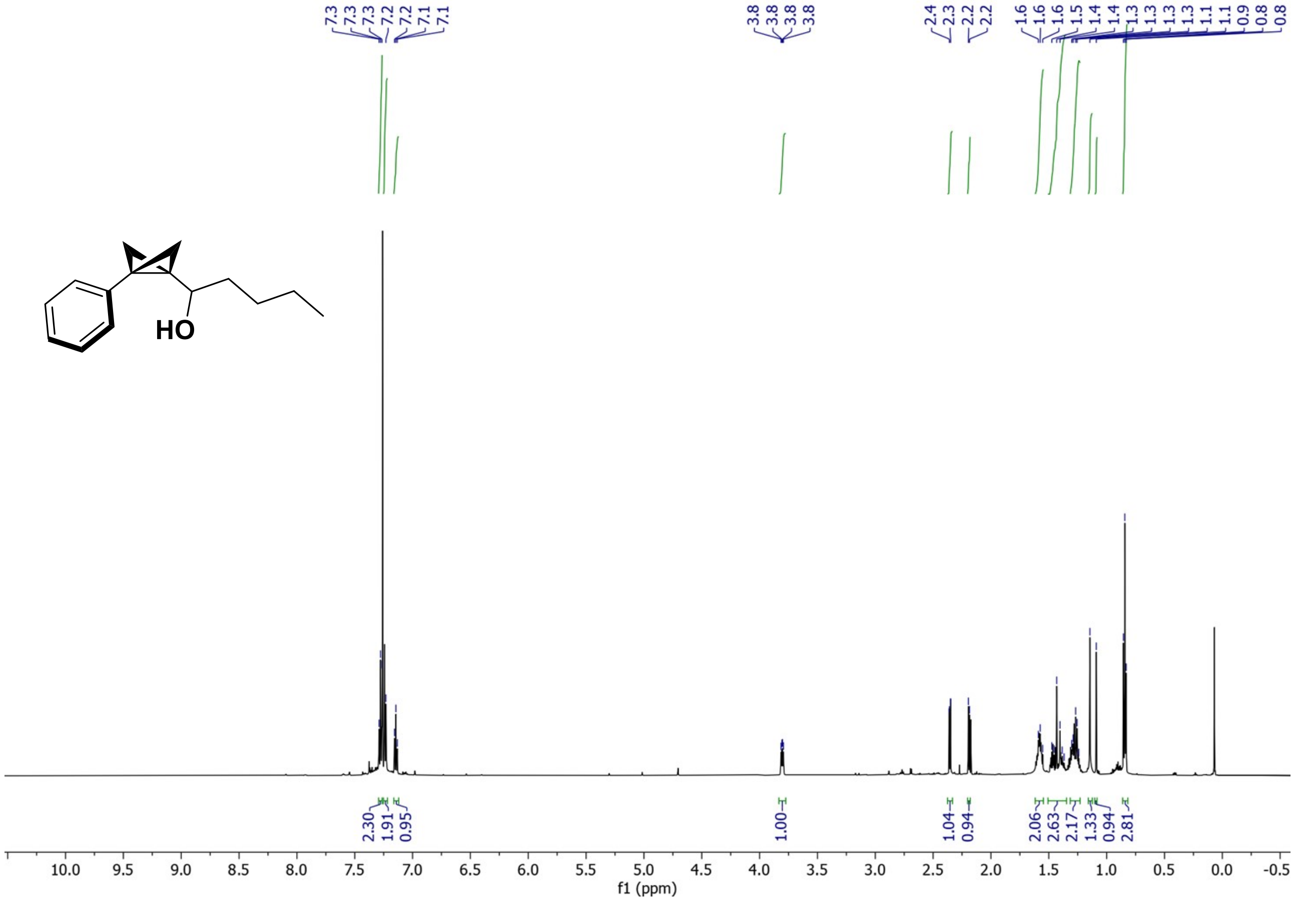

1v ^{13}C NMR (151 MHz, $CDCl_3$)

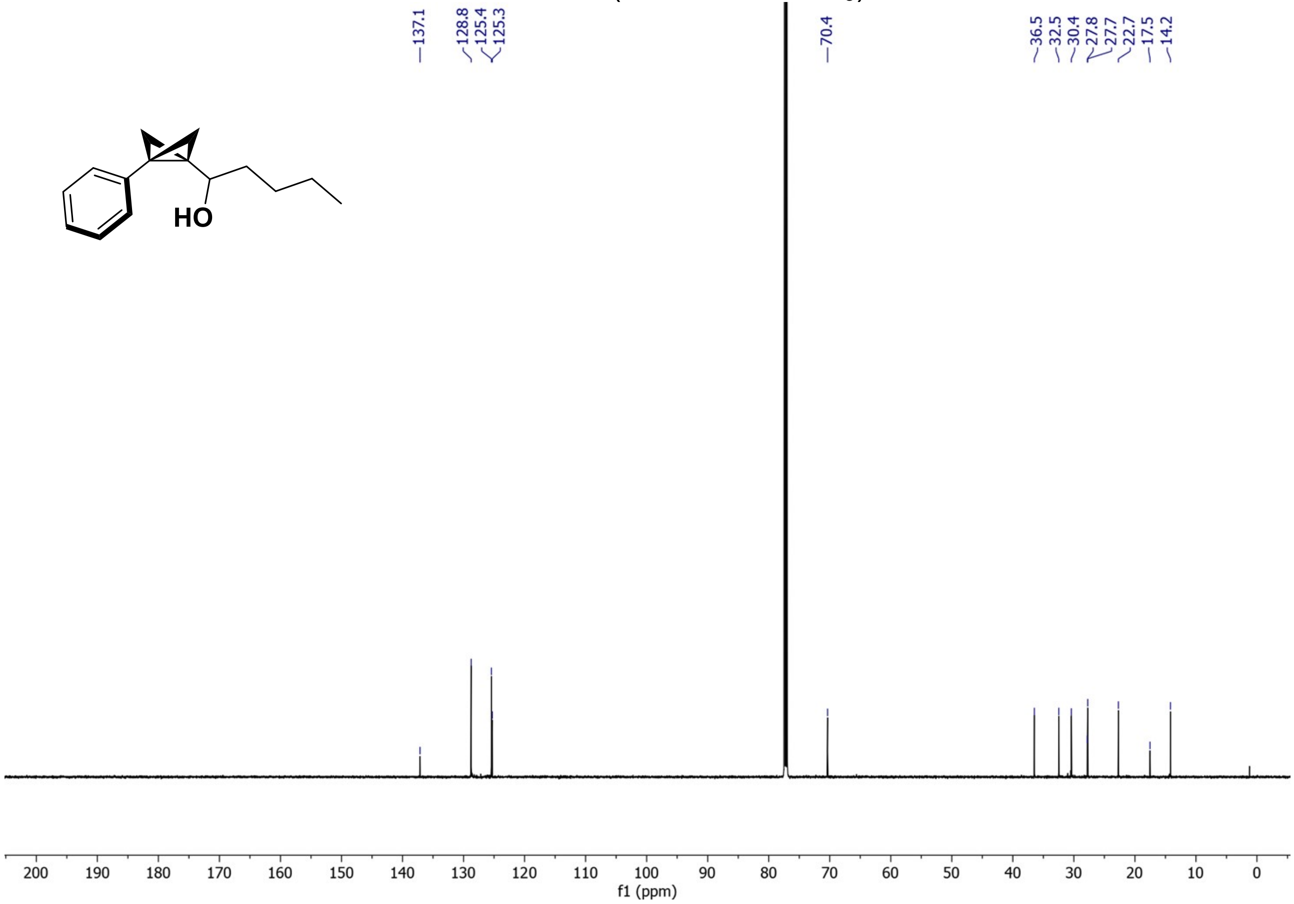

1w ^1H NMR (600 MHz, $CDCl_3$)

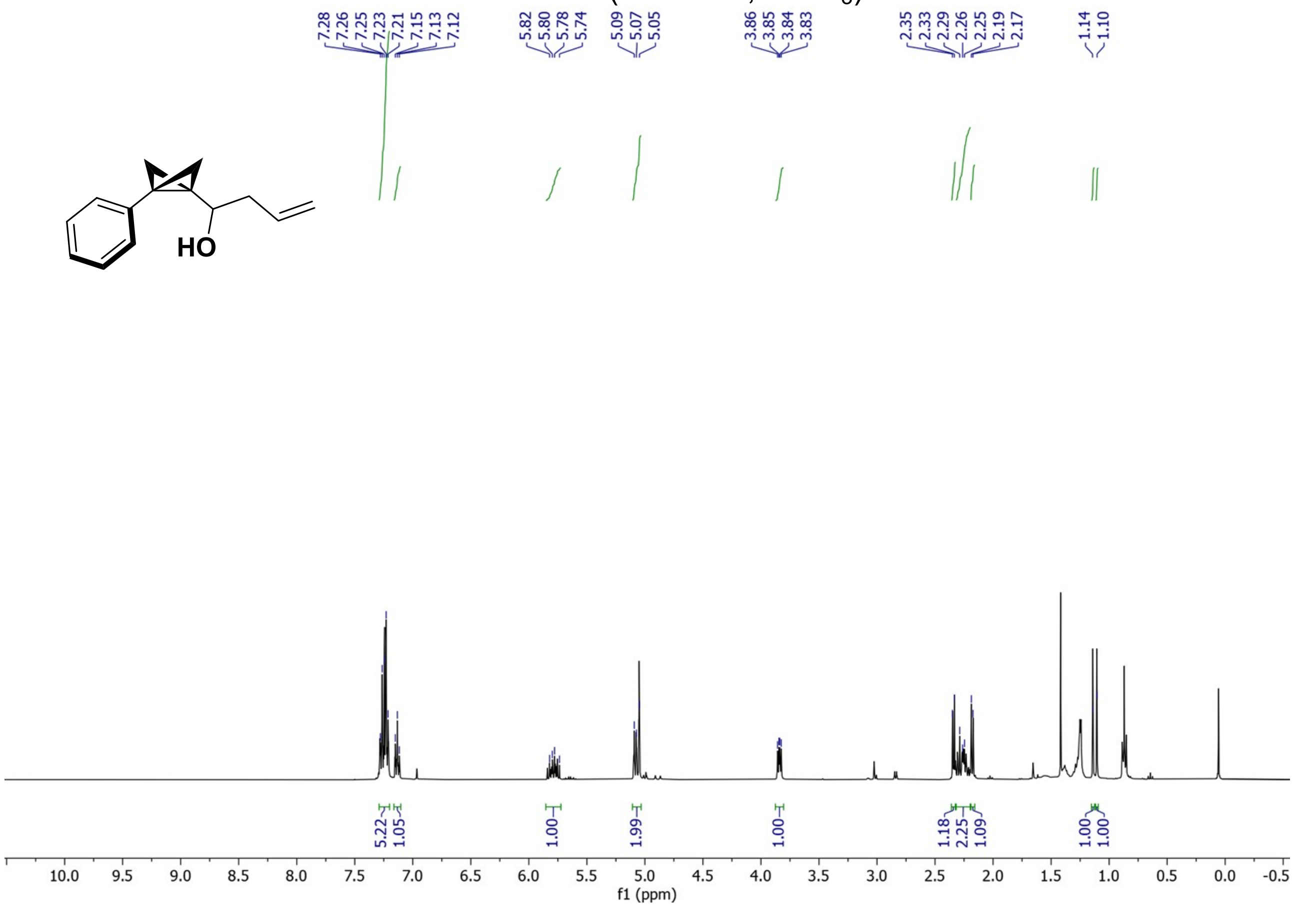

1w ^{13}C NMR (151 MHz, $CDCl_3$)

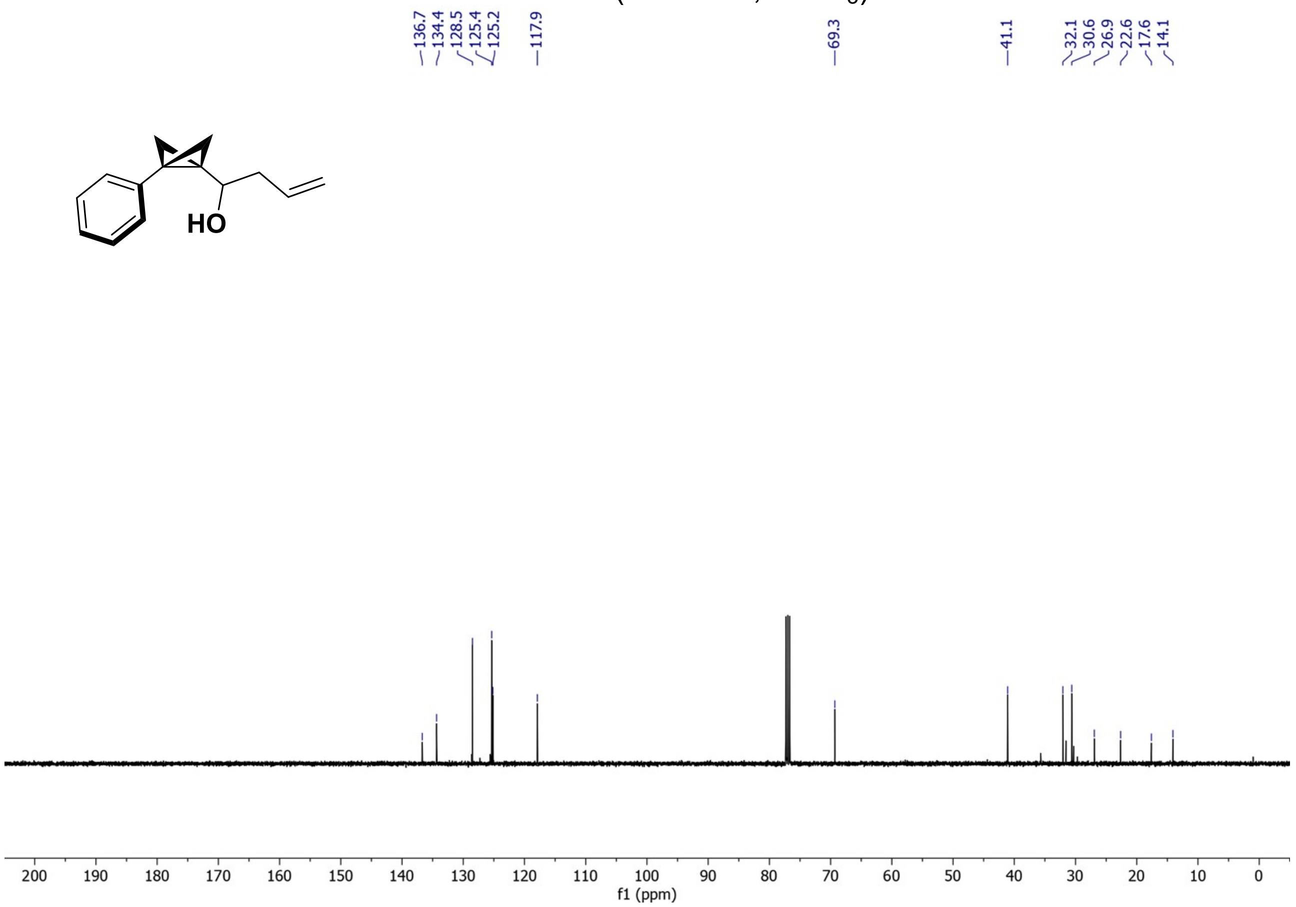

1x ^{1}H NMR (600 MHz, $CDCl_3$)

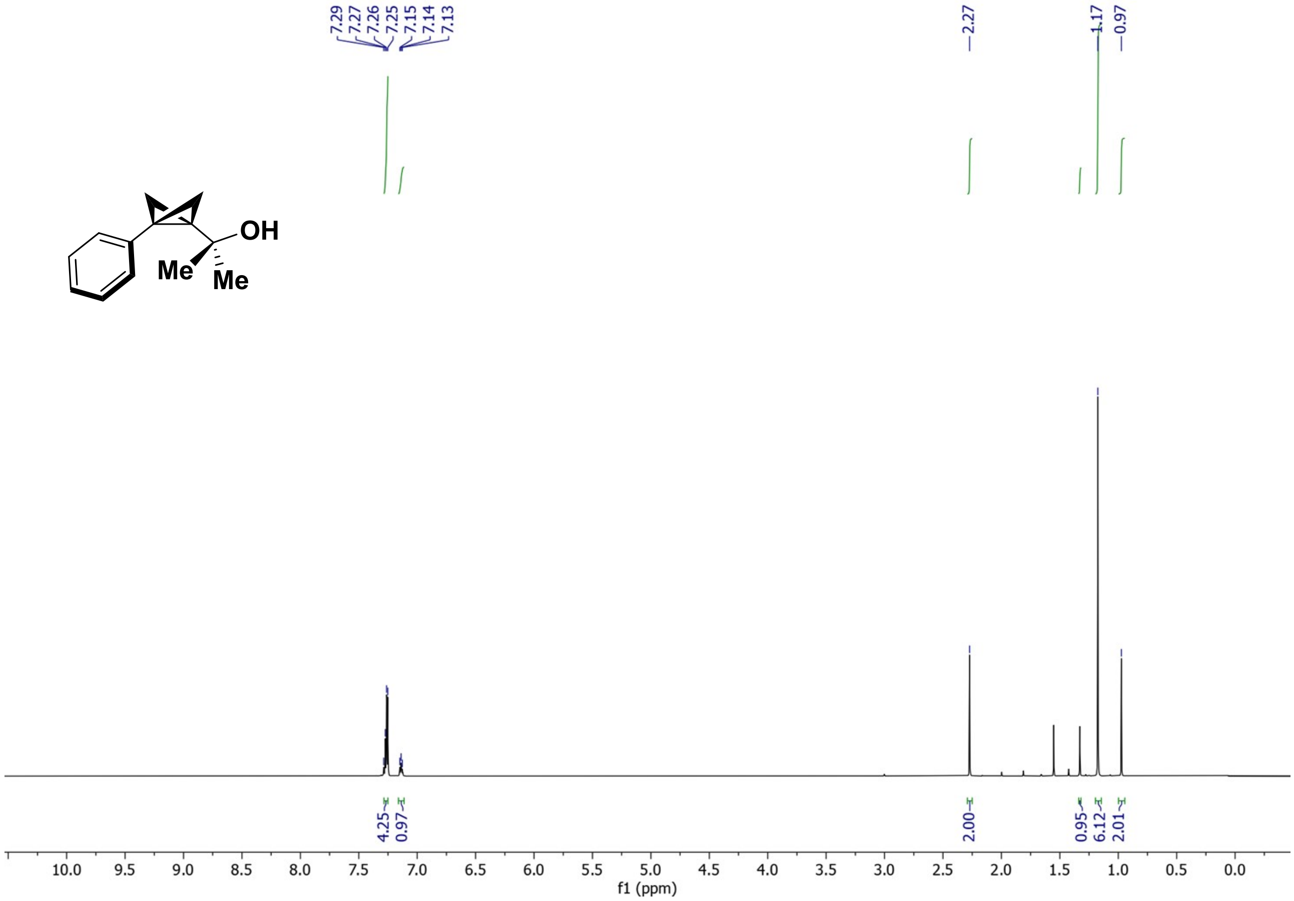

1x ^{13}C NMR (151 MHz, $CDCl_3$)

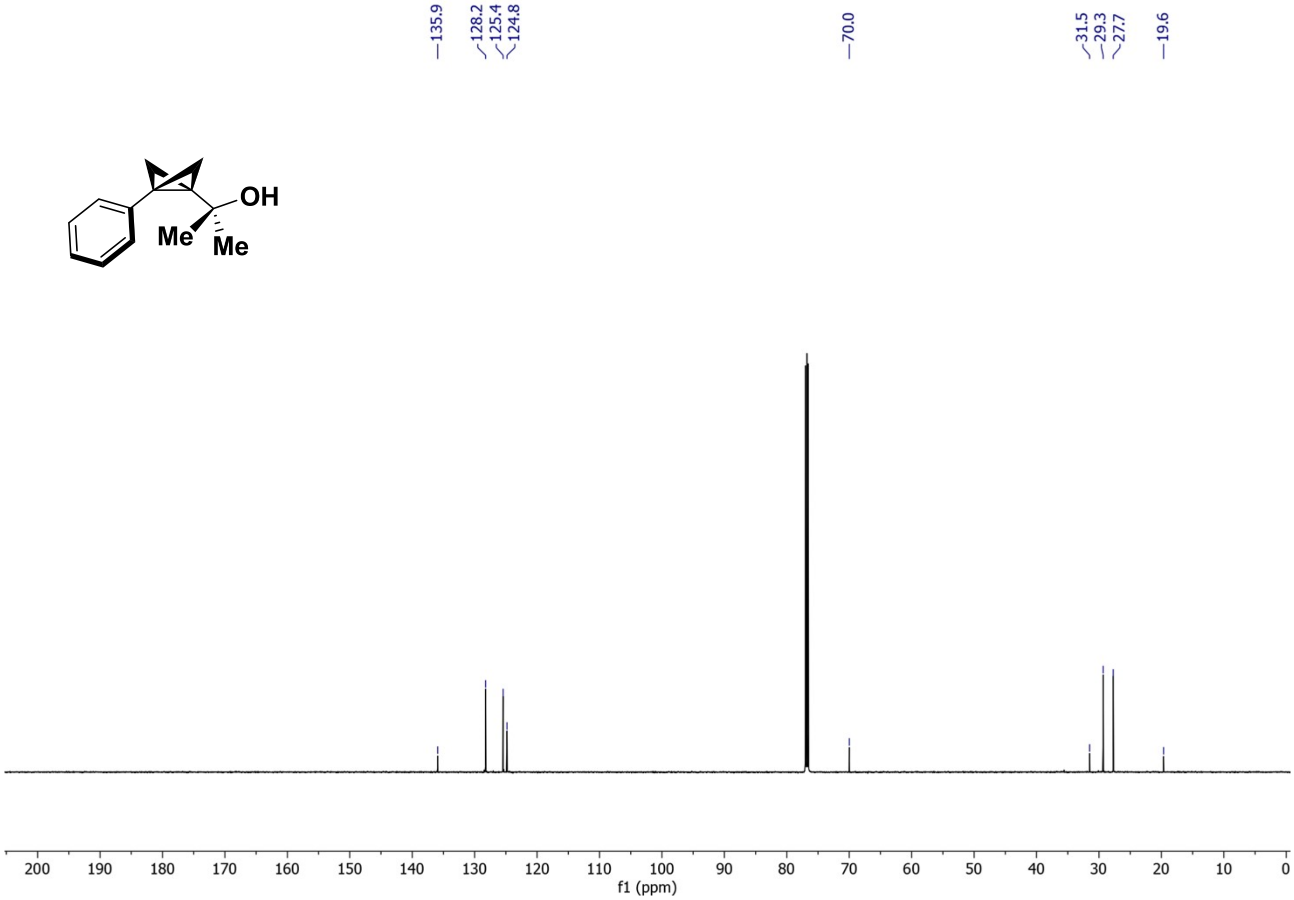

***d*$_4$-1a** **^{1}H NMR** (600 MHz, $CDCl_3$)

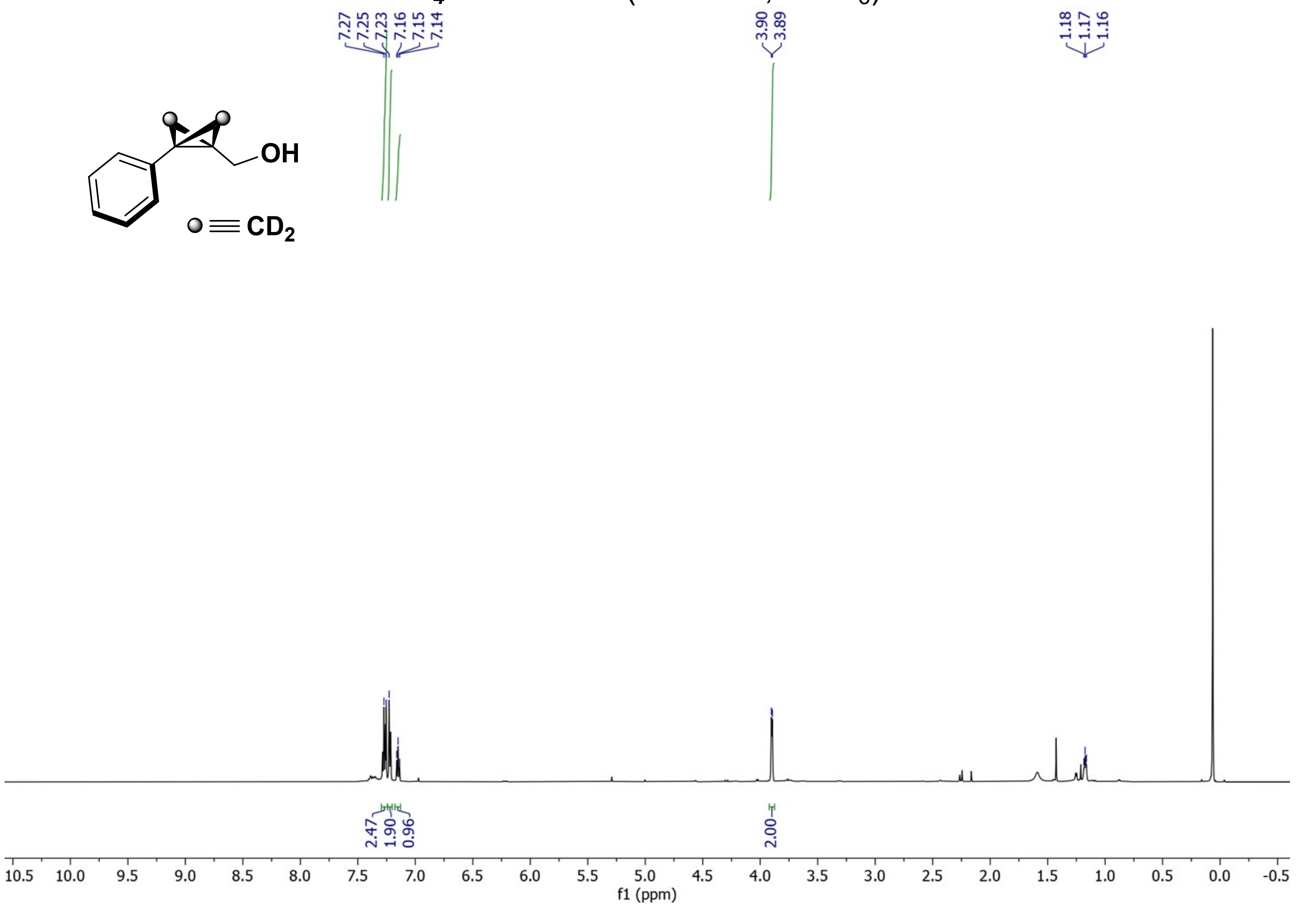

***d*$_4$-1a** **^{13}C NMR** (151 MHz, $CDCl_3$)

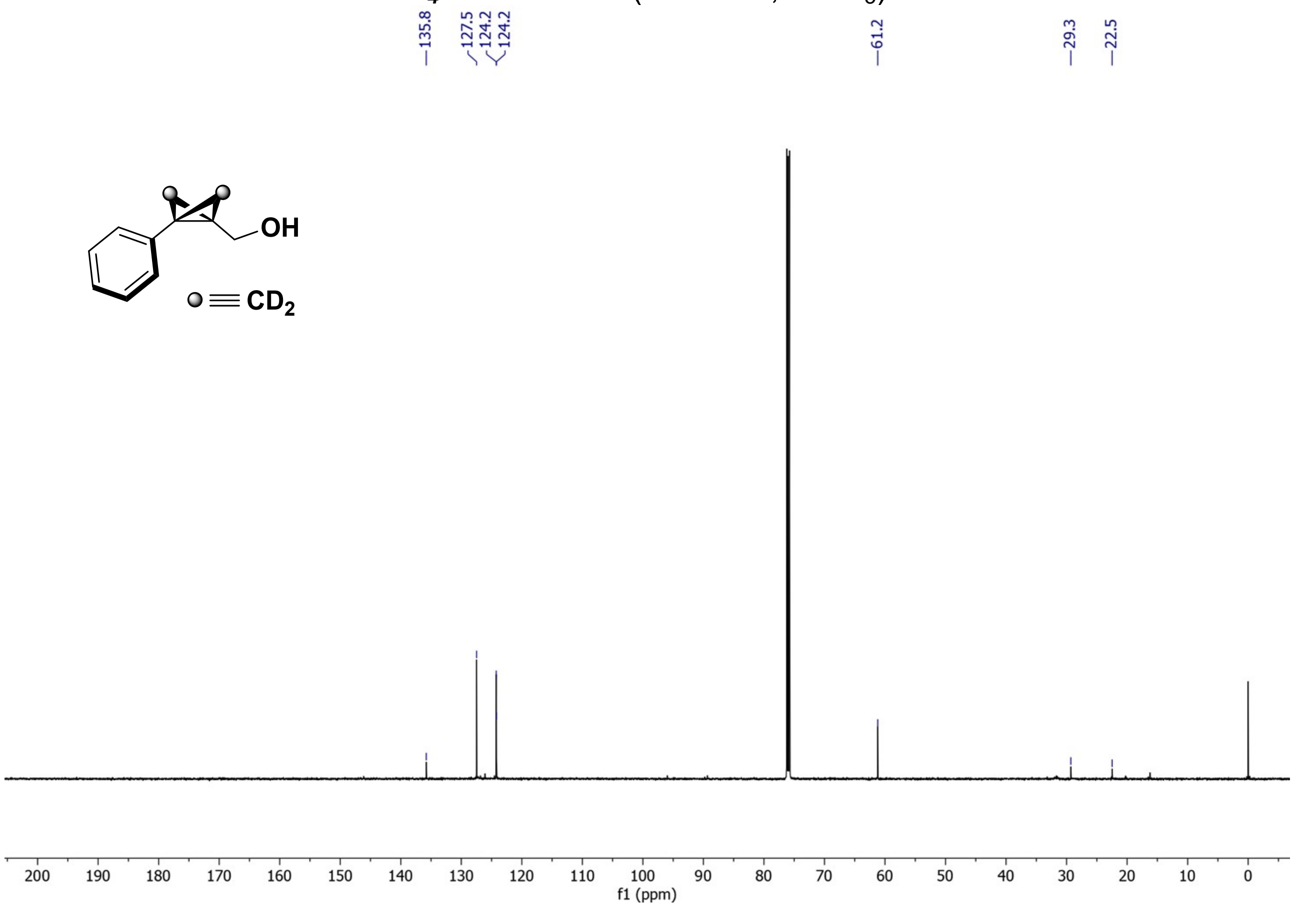

3aa ^{1}H NMR (600 MHz, $CDCl_3$)

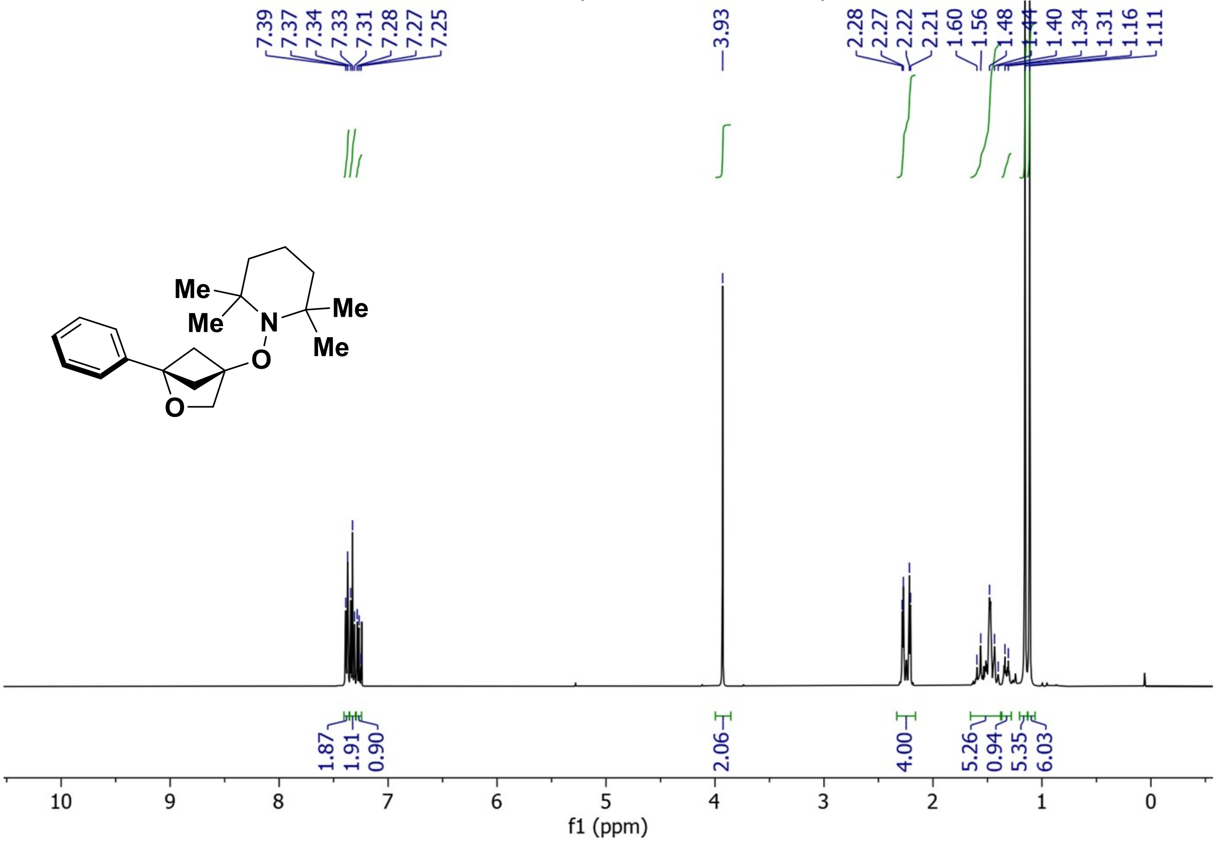

3aa ^{13}C NMR (151 MHz, $CDCl_3$)

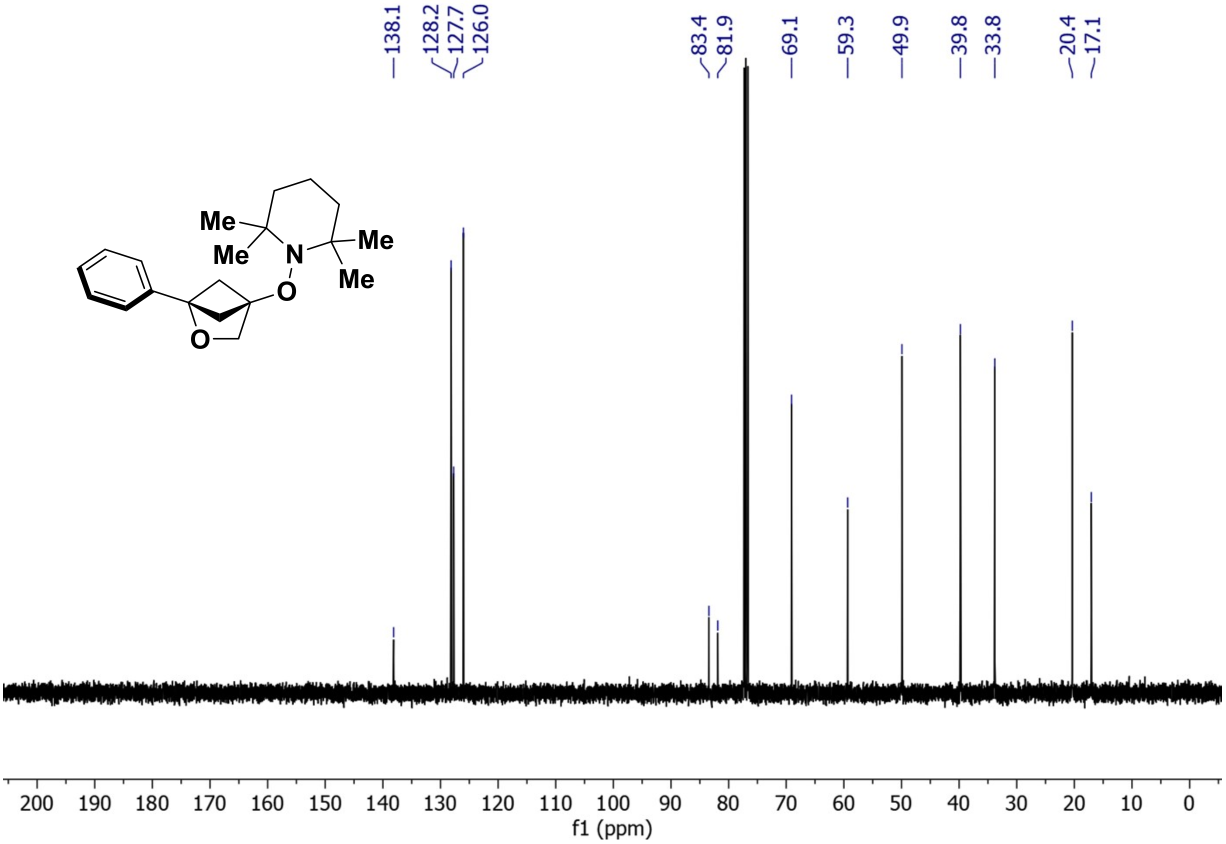

3ba ^{1}**H NMR** (600 MHz, $CDCl_3$)

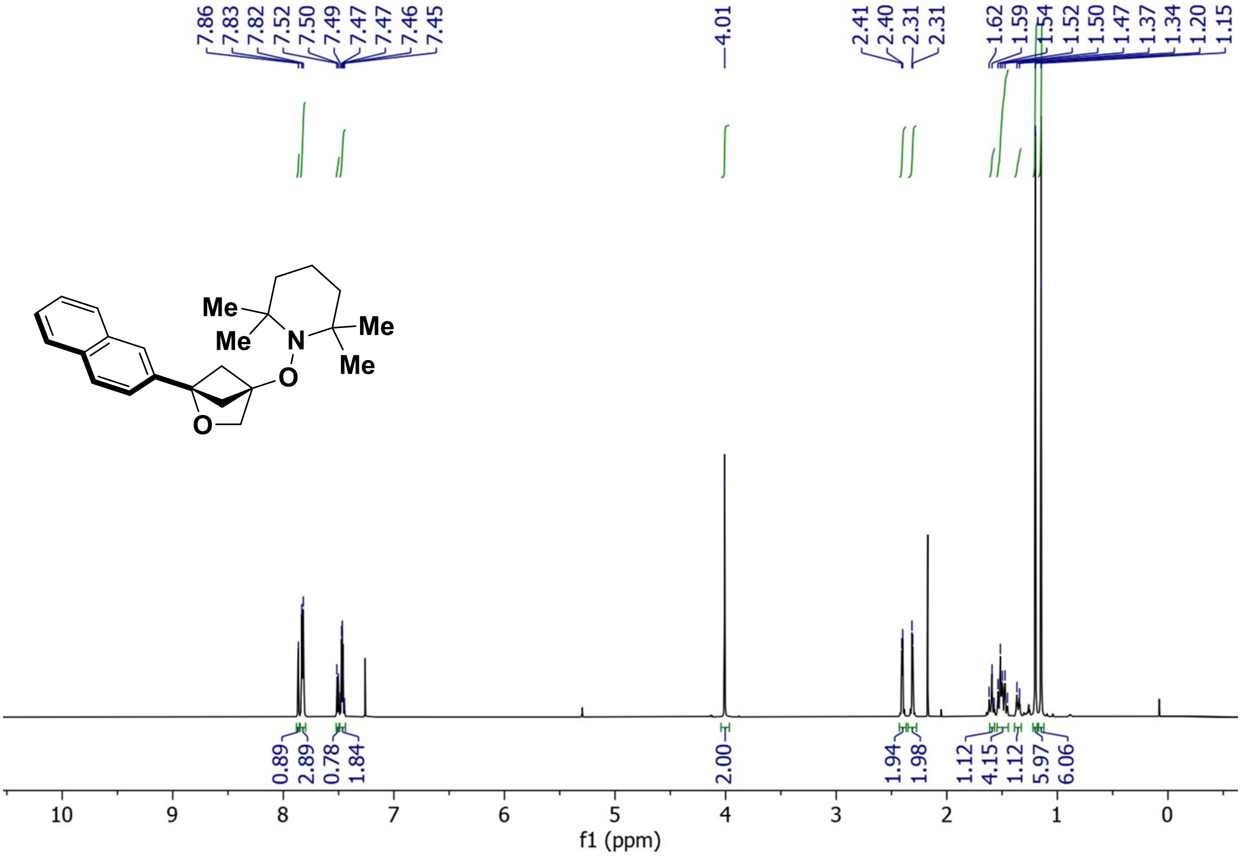

3ba ^{13}**C NMR** (151 MHz, $CDCl_3$)

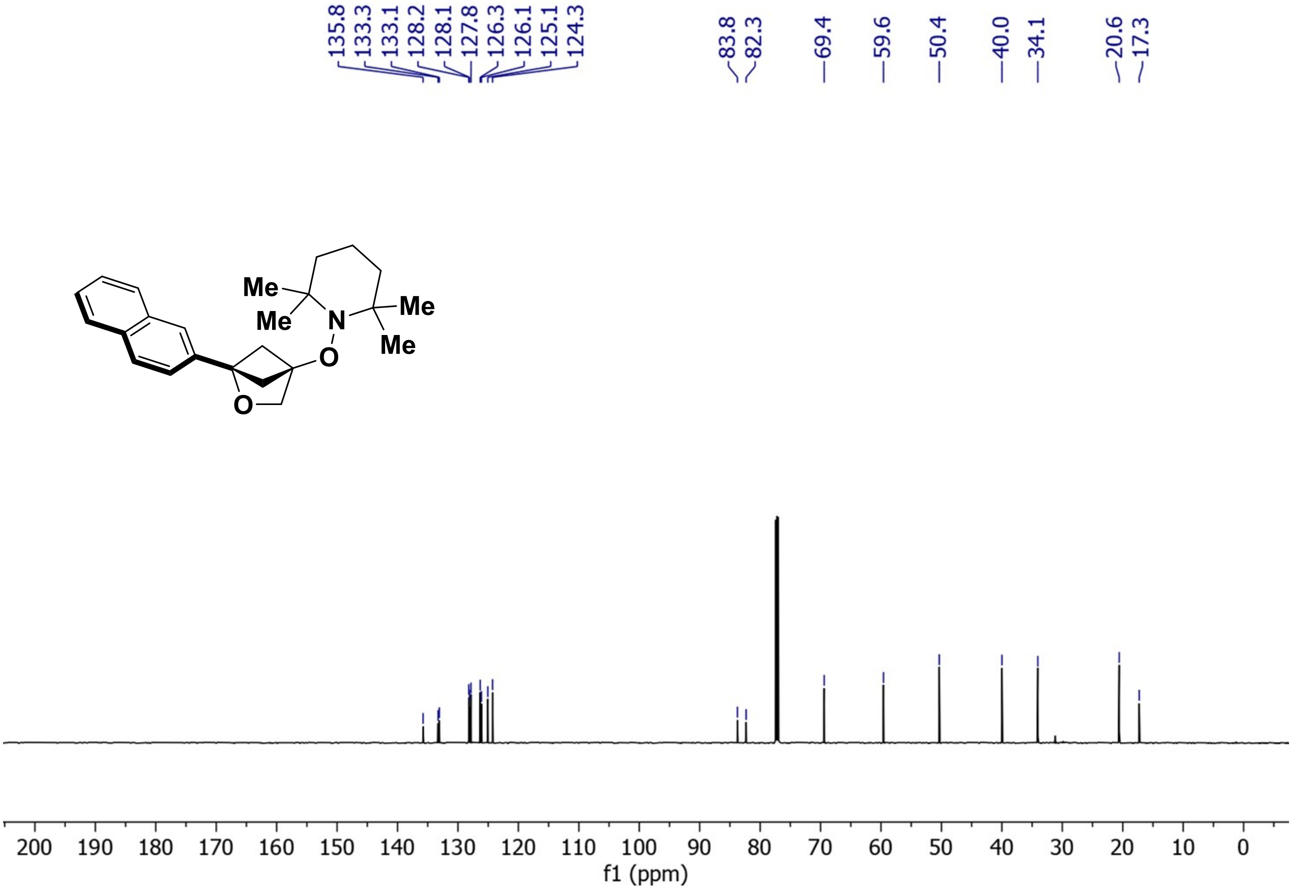

3ca ^{1}**H NMR** (600 MHz, $CDCl_3$)

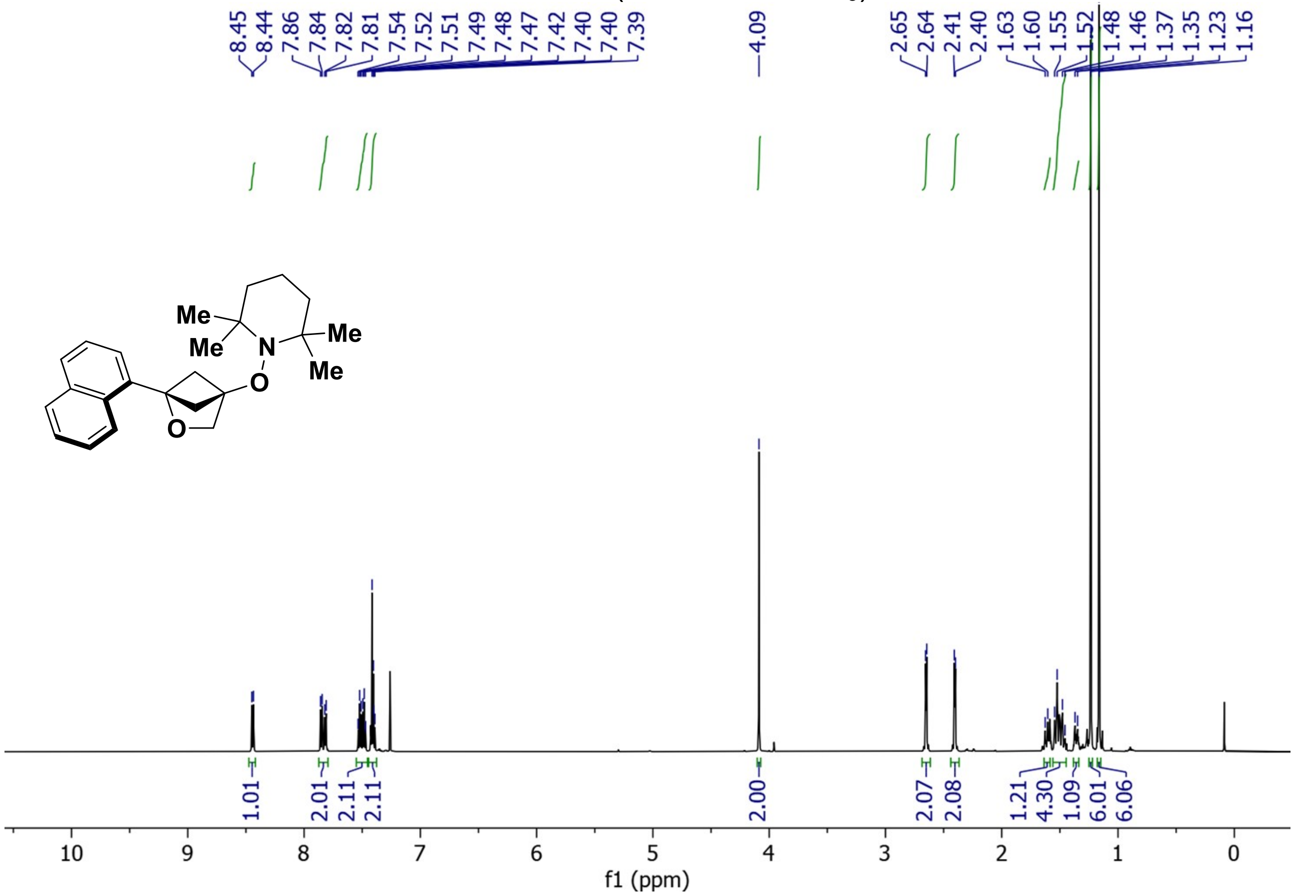

3ca ^{13}**C NMR** (151 MHz, $CDCl_3$)

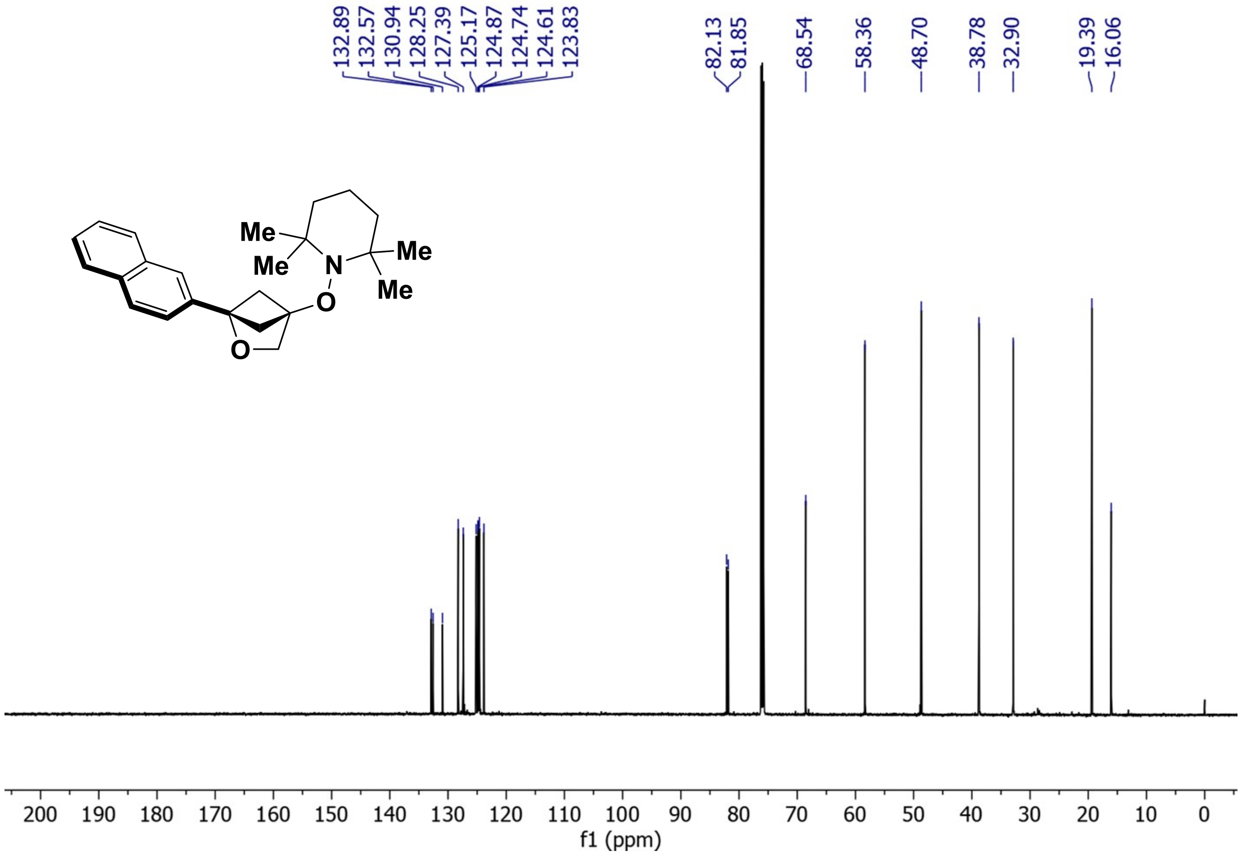

3da ^{1}H NMR (600 MHz, $CDCl_3$)

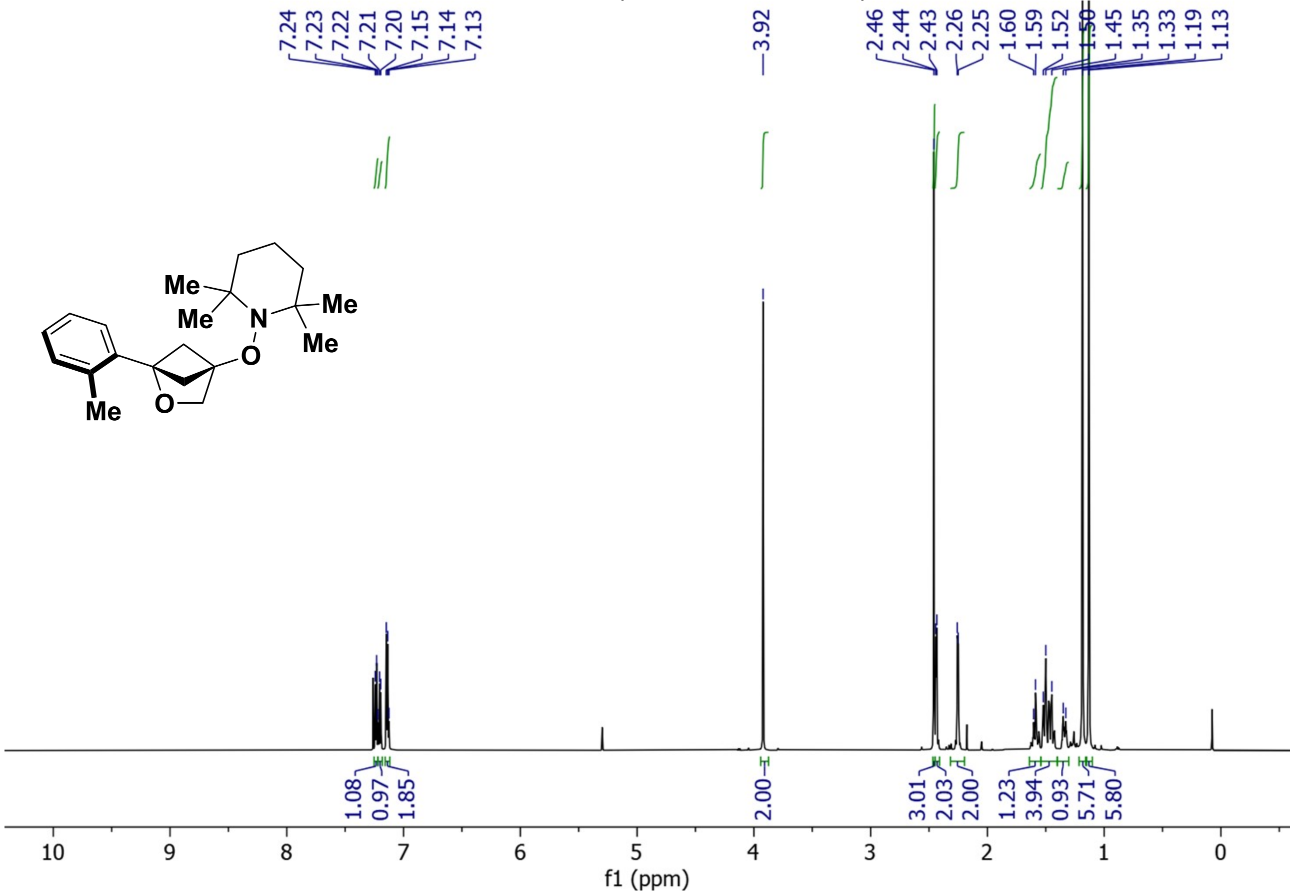

3da ^{13}C NMR (151 MHz, $CDCl_3$)

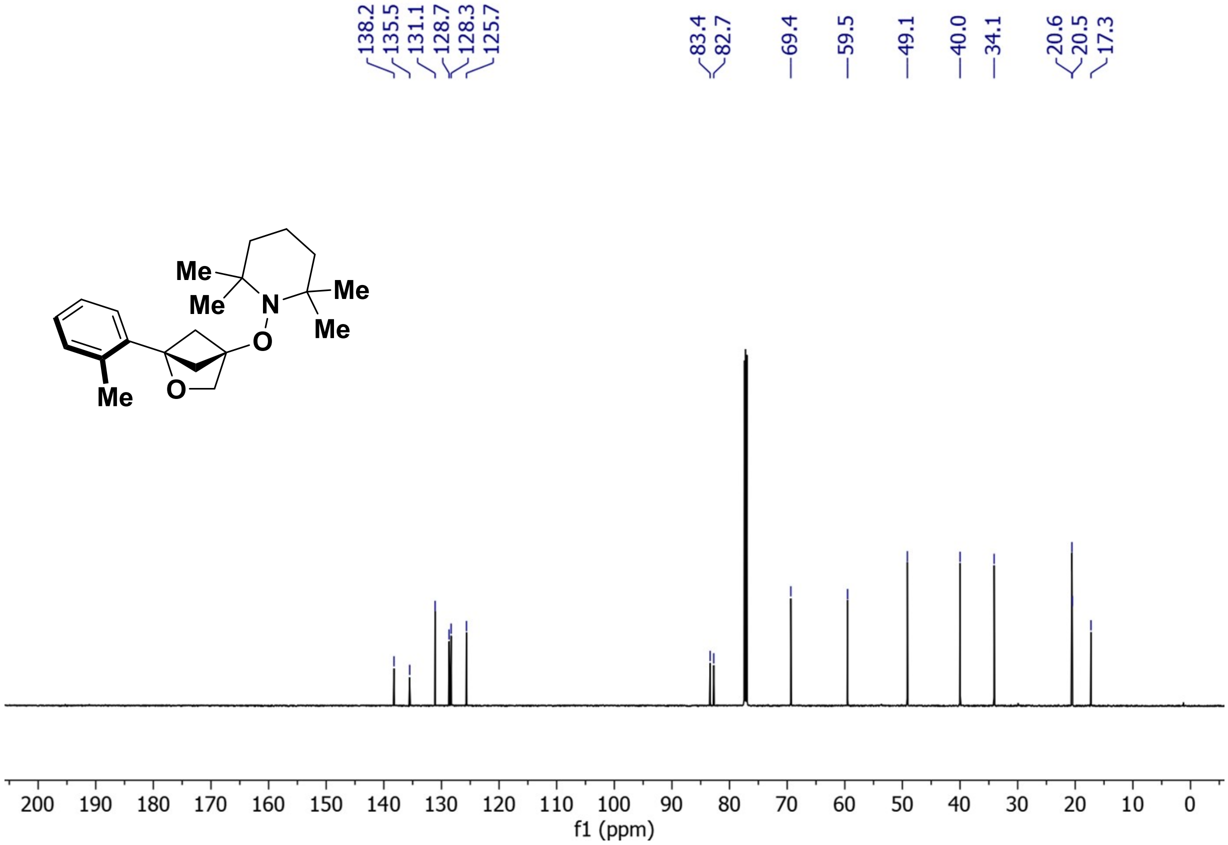

3ea ^{1}**H NMR** (600 MHz, $CDCl_3$)

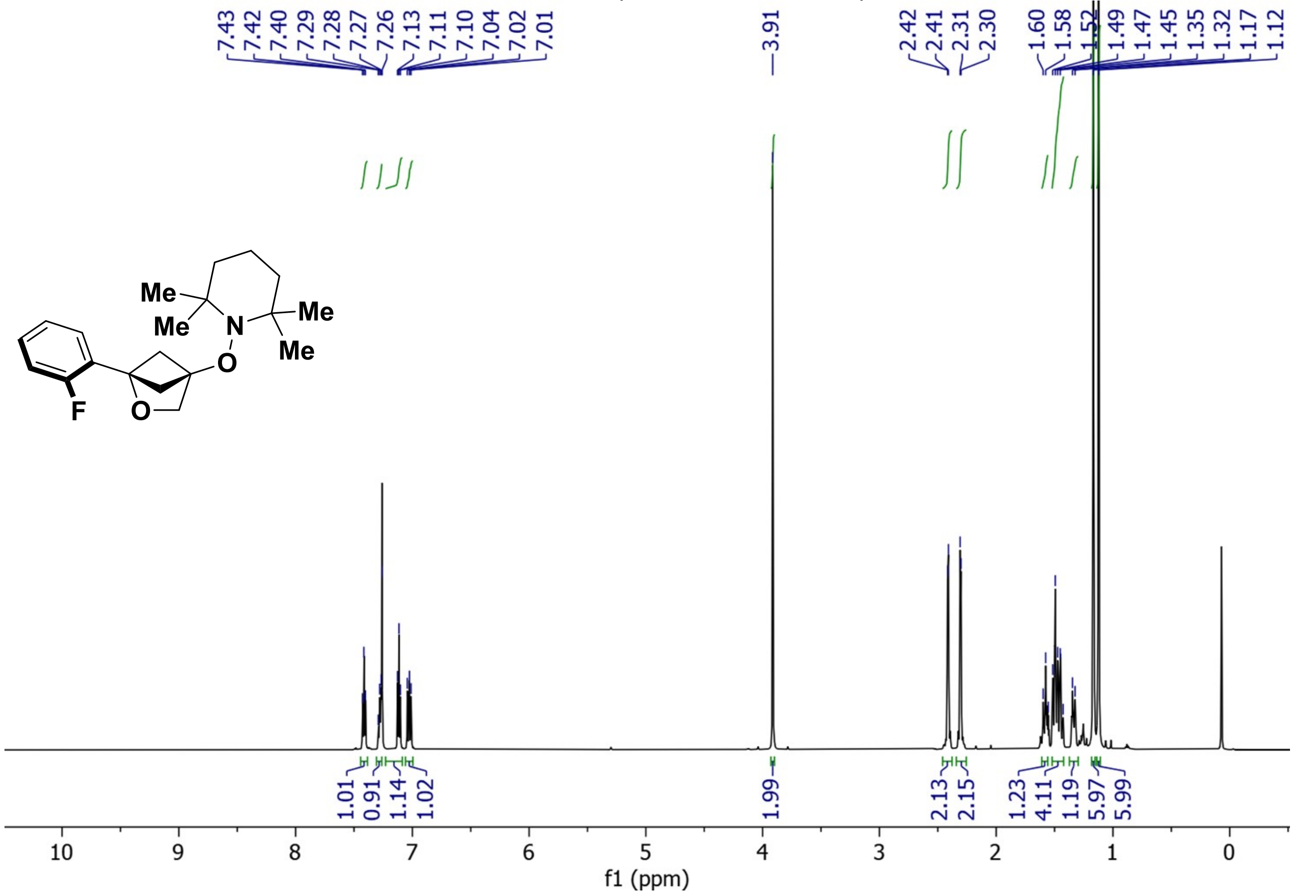

3ea ^{13}**C NMR** (151 MHz, $CDCl_3$)

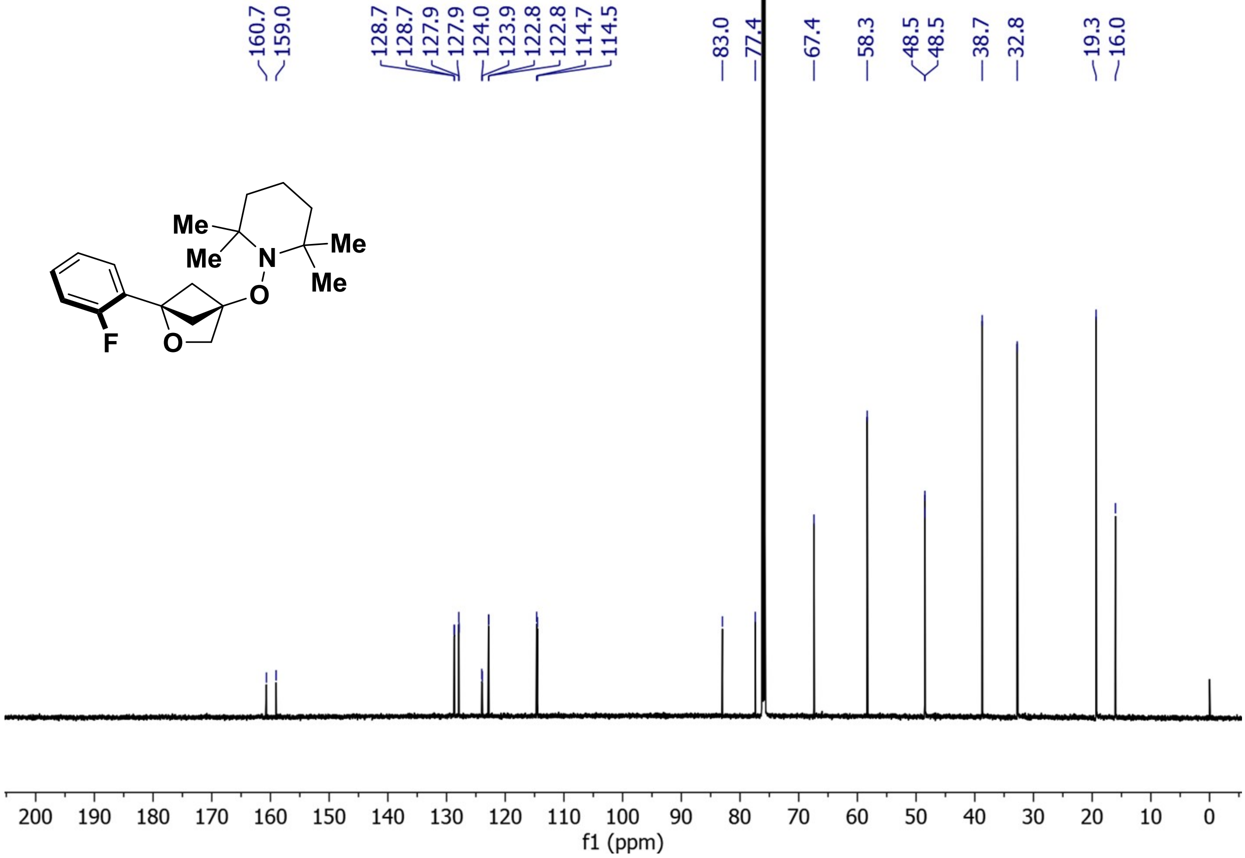

3ea ^{19}F NMR (565 MHz, $CDCl_3$)

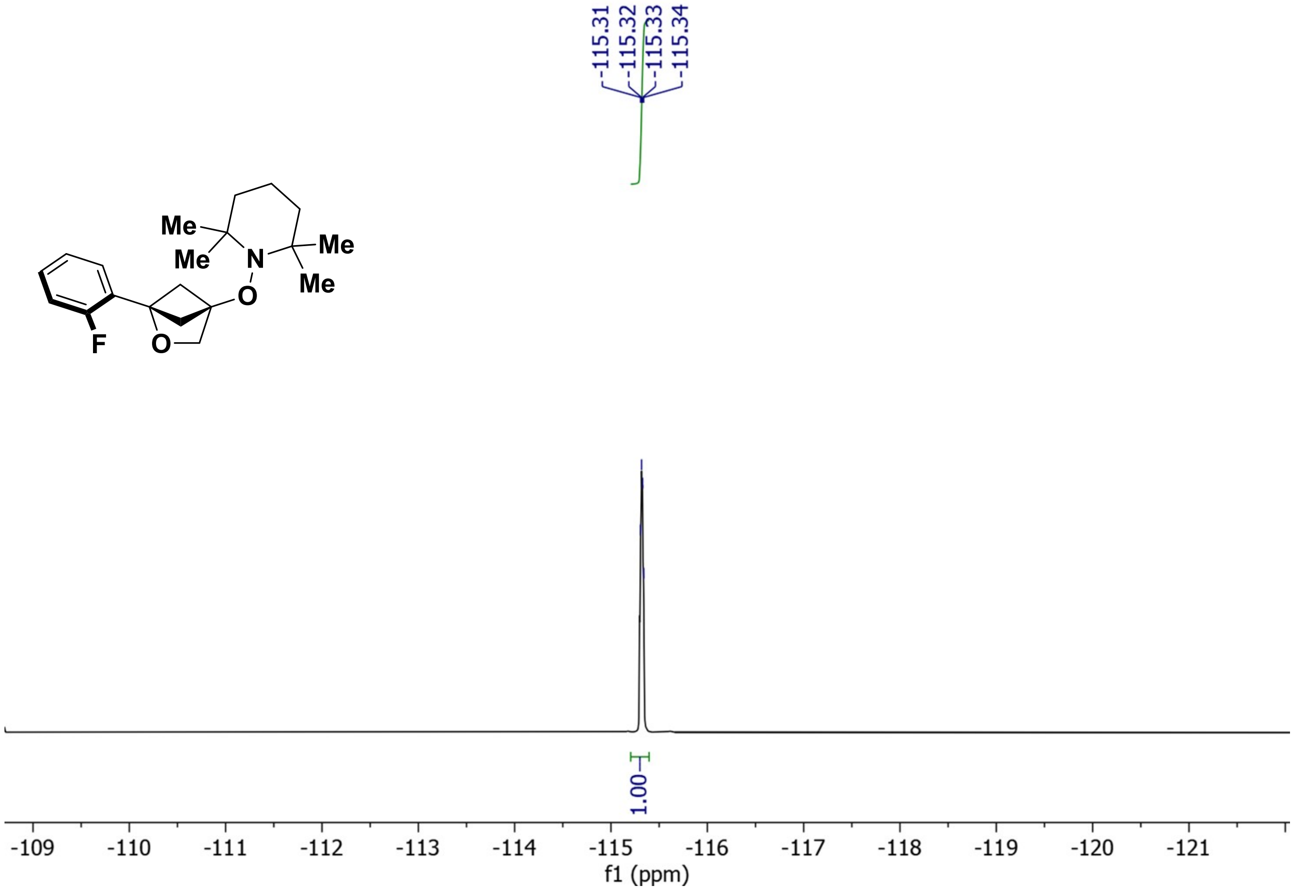

3fa ^{1}H NMR (600 MHz, $CDCl_3$)

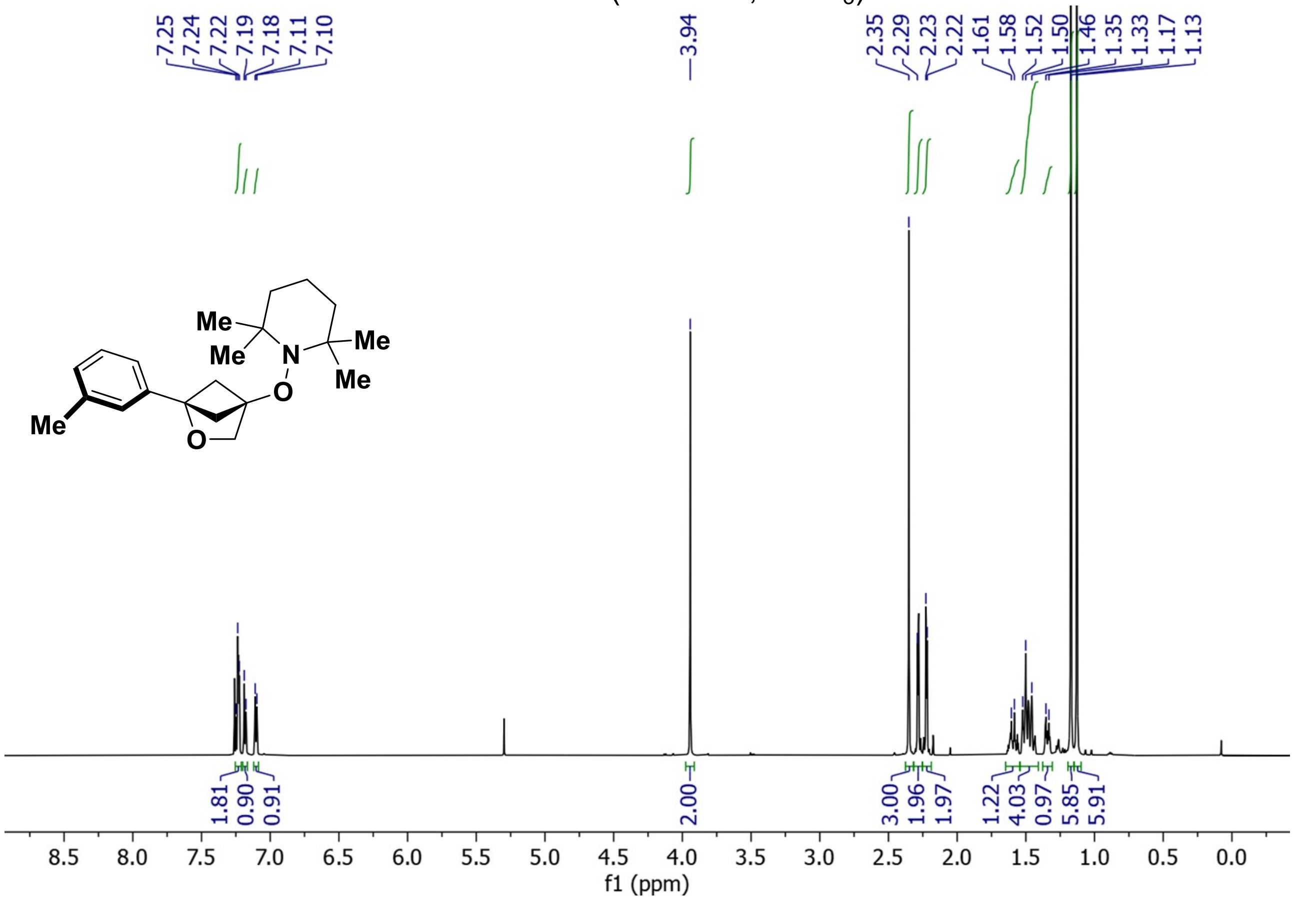

3fa ^{13}C NMR (151 MHz, $CDCl_3$)

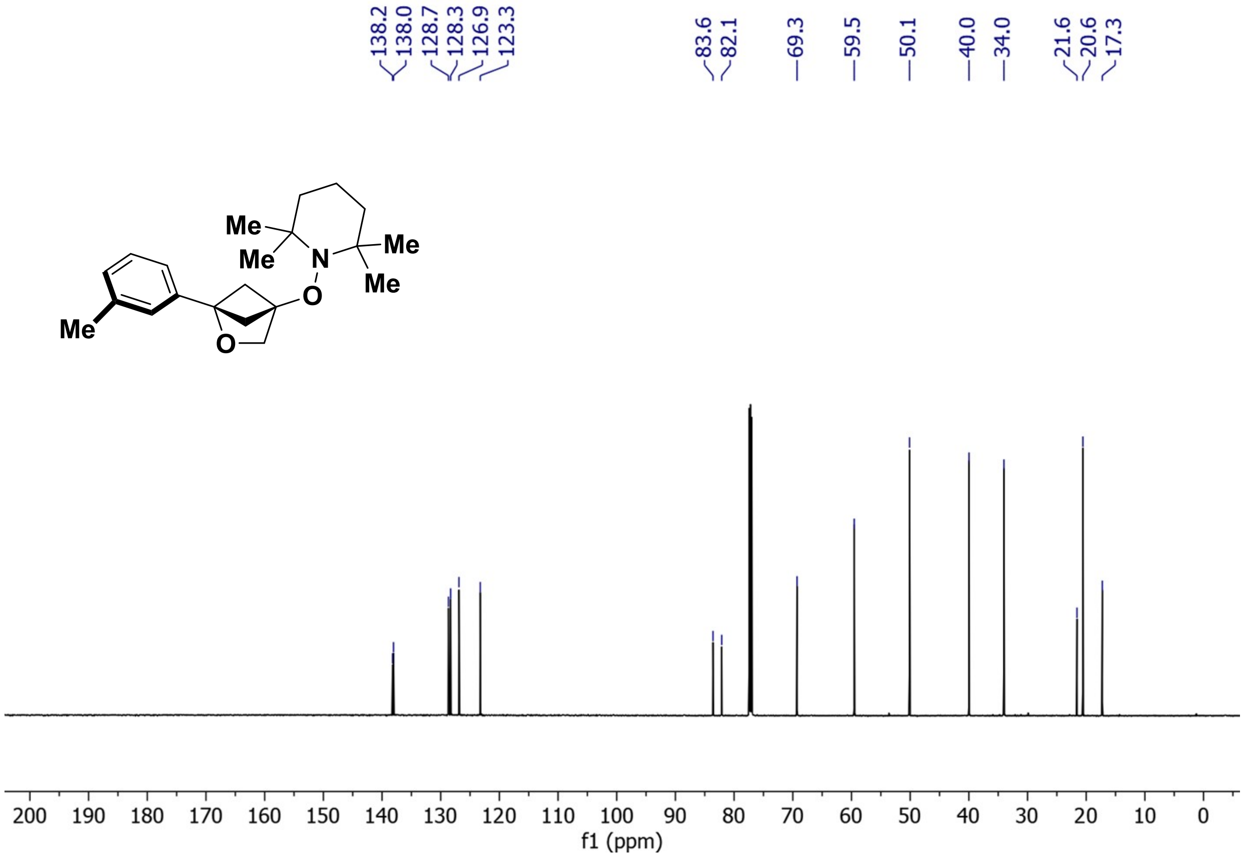

3ga ^{1}H NMR (600 MHz, $CDCl_3$)

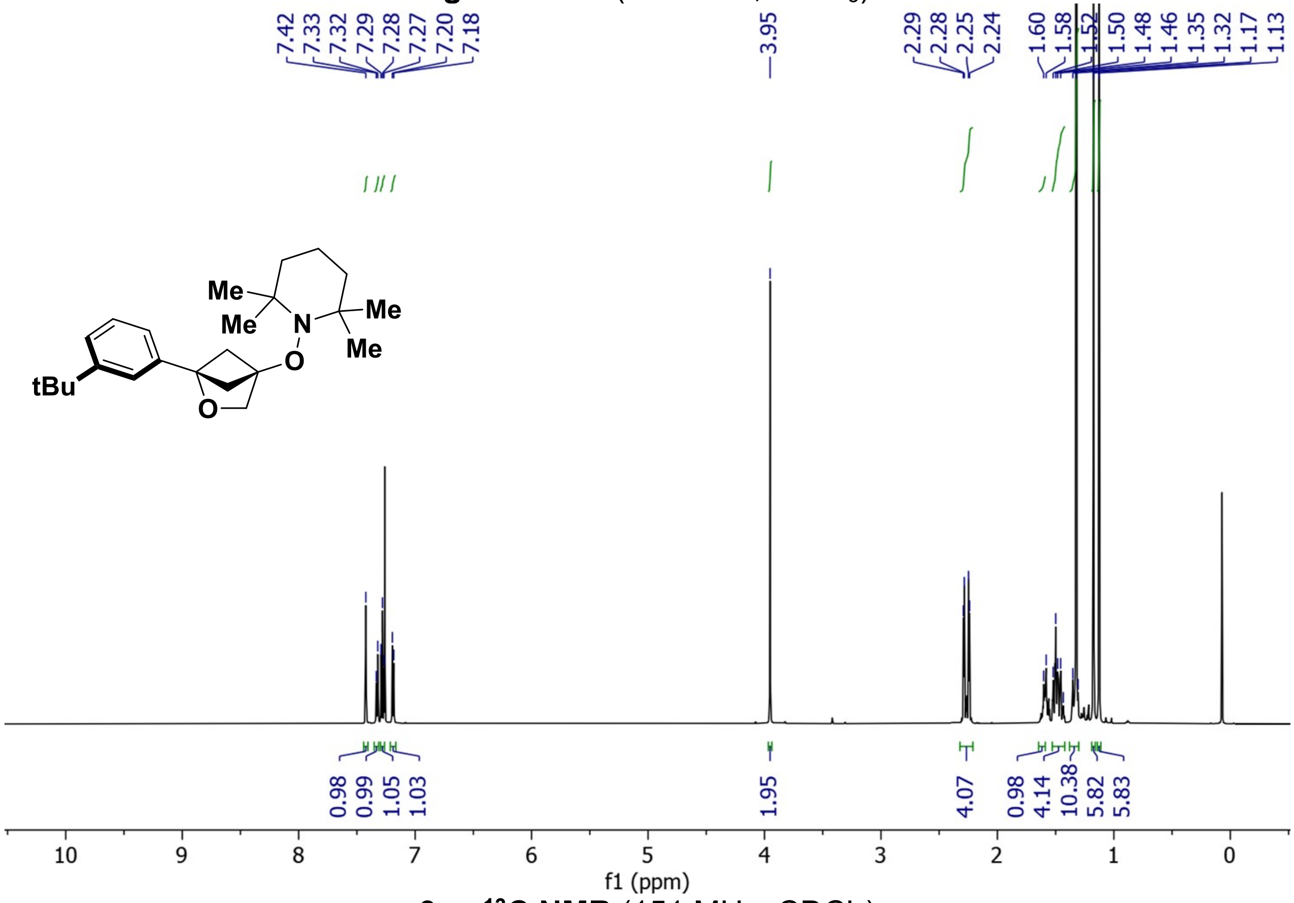

3ga ^{13}C NMR (151 MHz, $CDCl_3$)

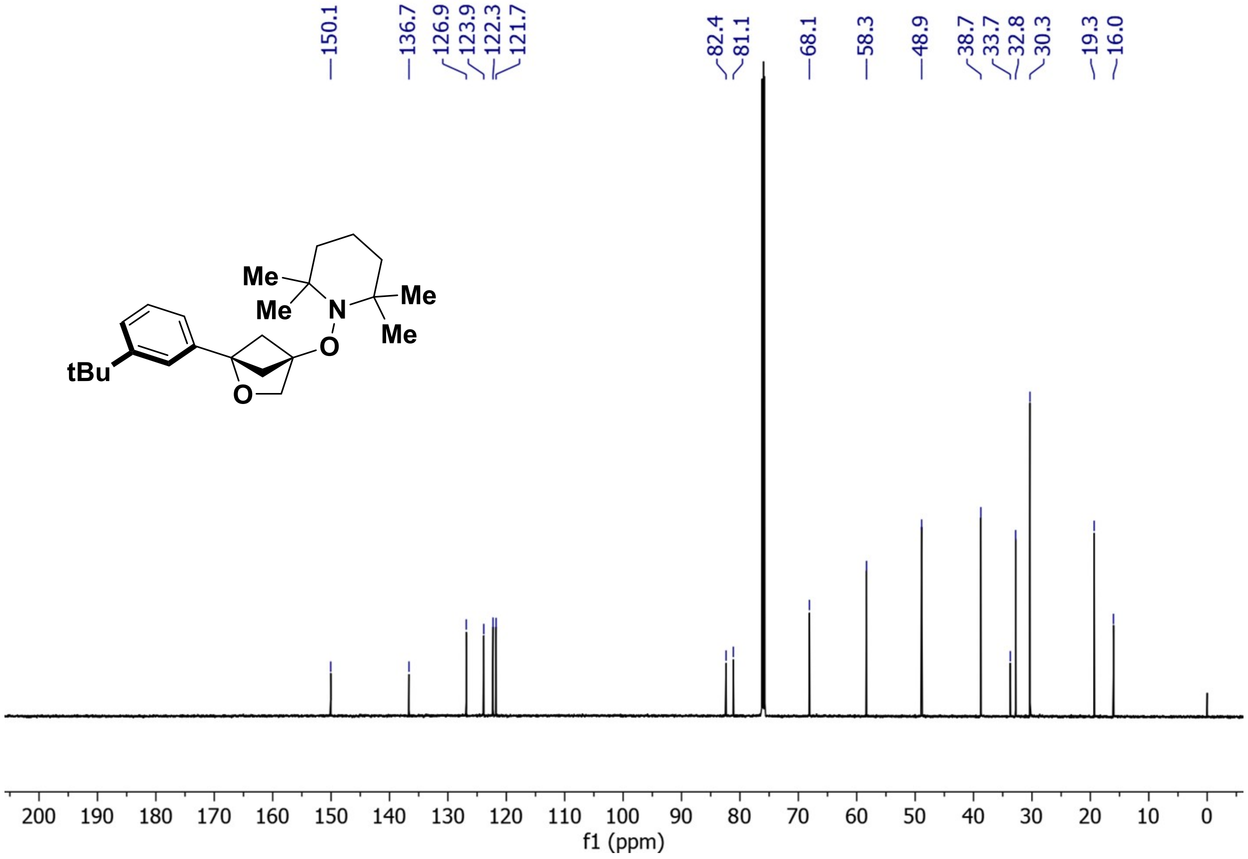

3ha ^{1}**H NMR** (600 MHz, $CDCl_3$)

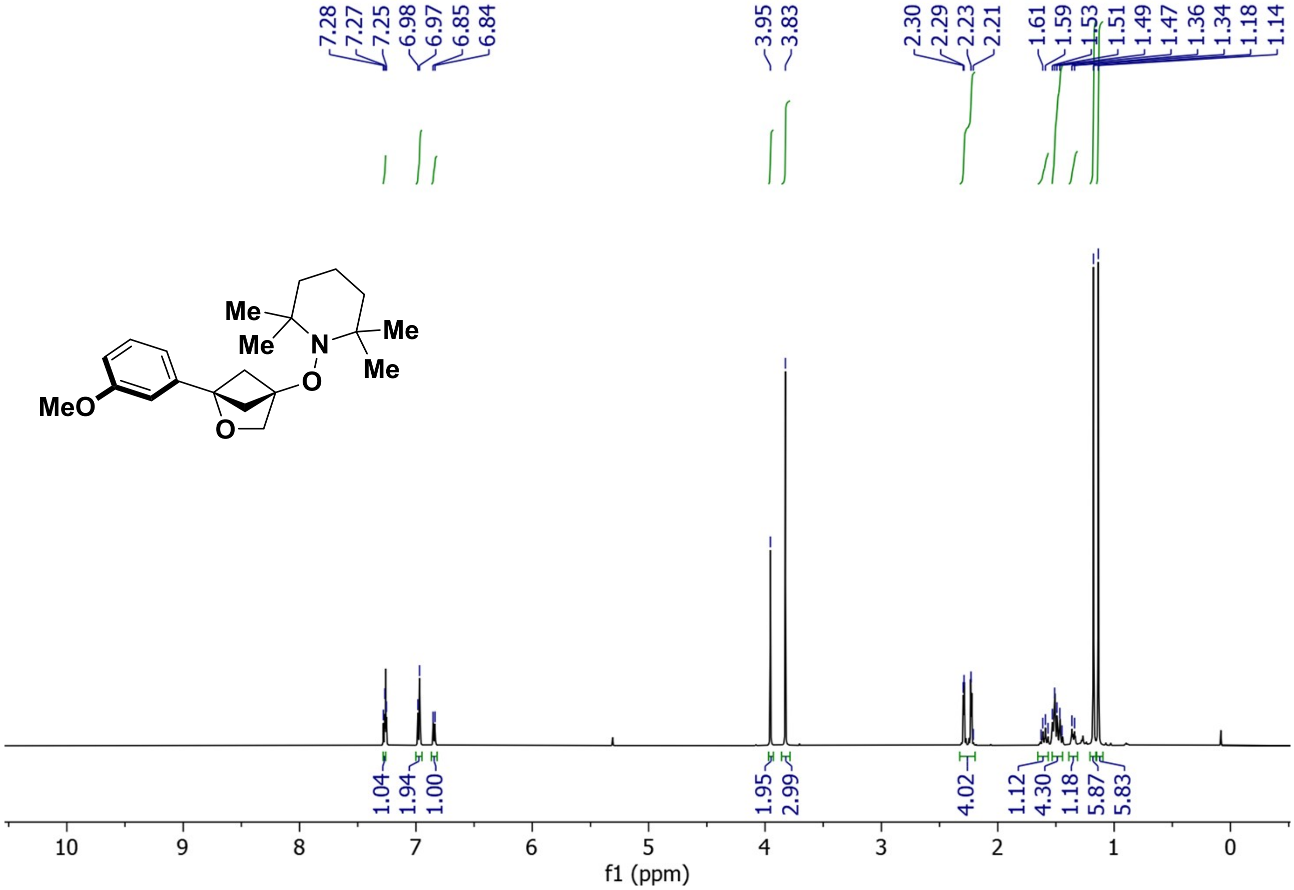

3ha ^{13}**C NMR** (151 MHz, $CDCl_3$)

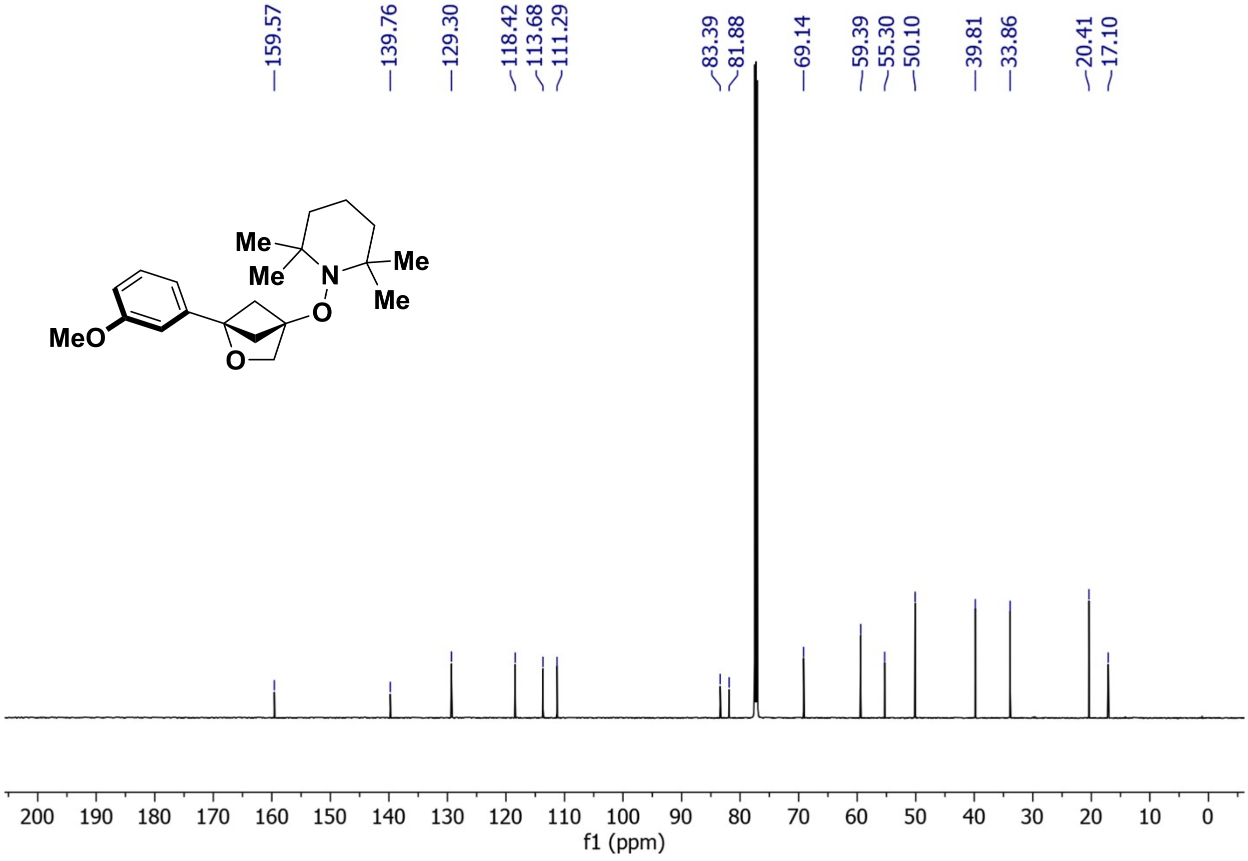

3ia ^{1}H NMR (600 MHz, $CDCl_3$)

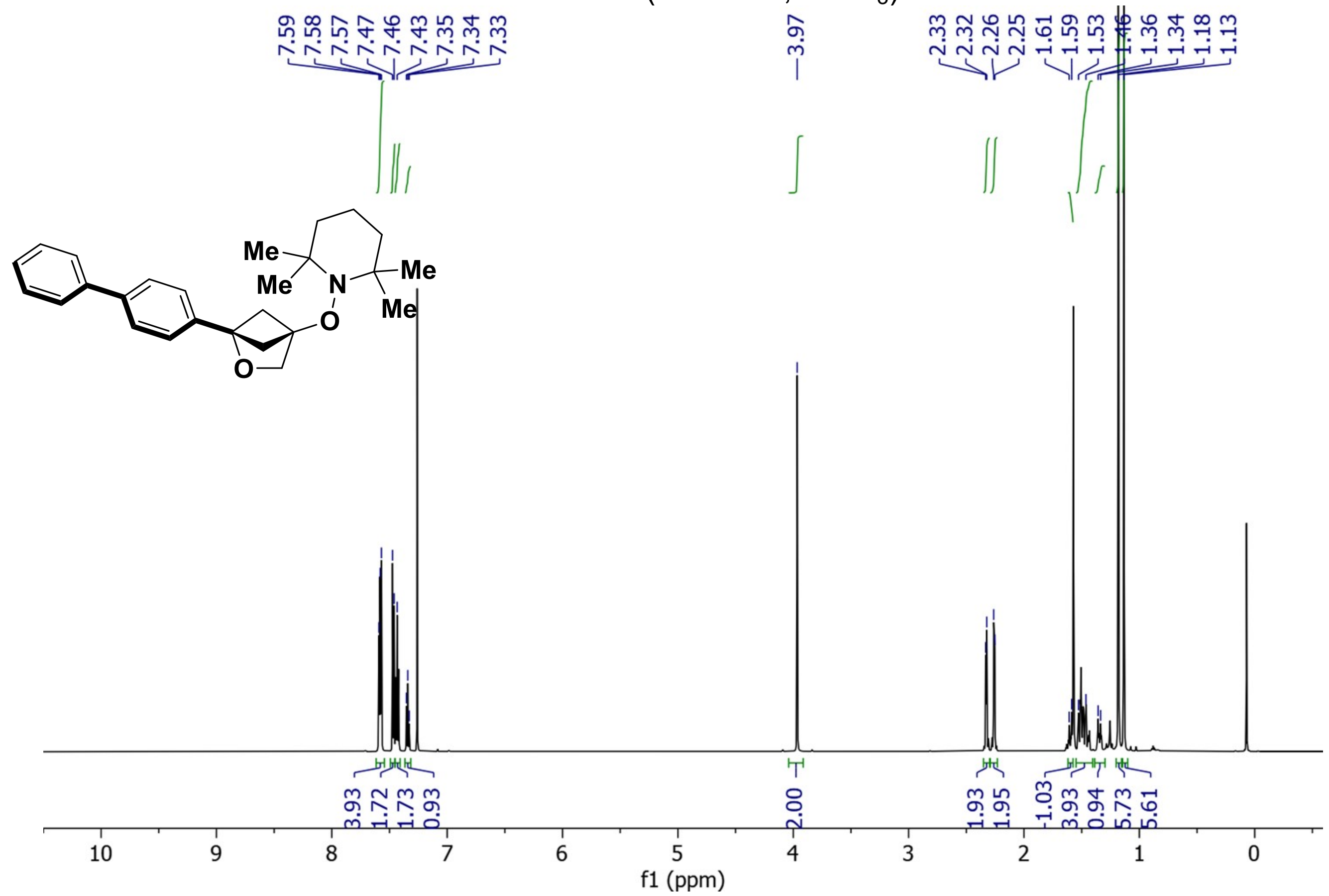

3ia ^{13}C NMR (151 MHz, $CDCl_3$)

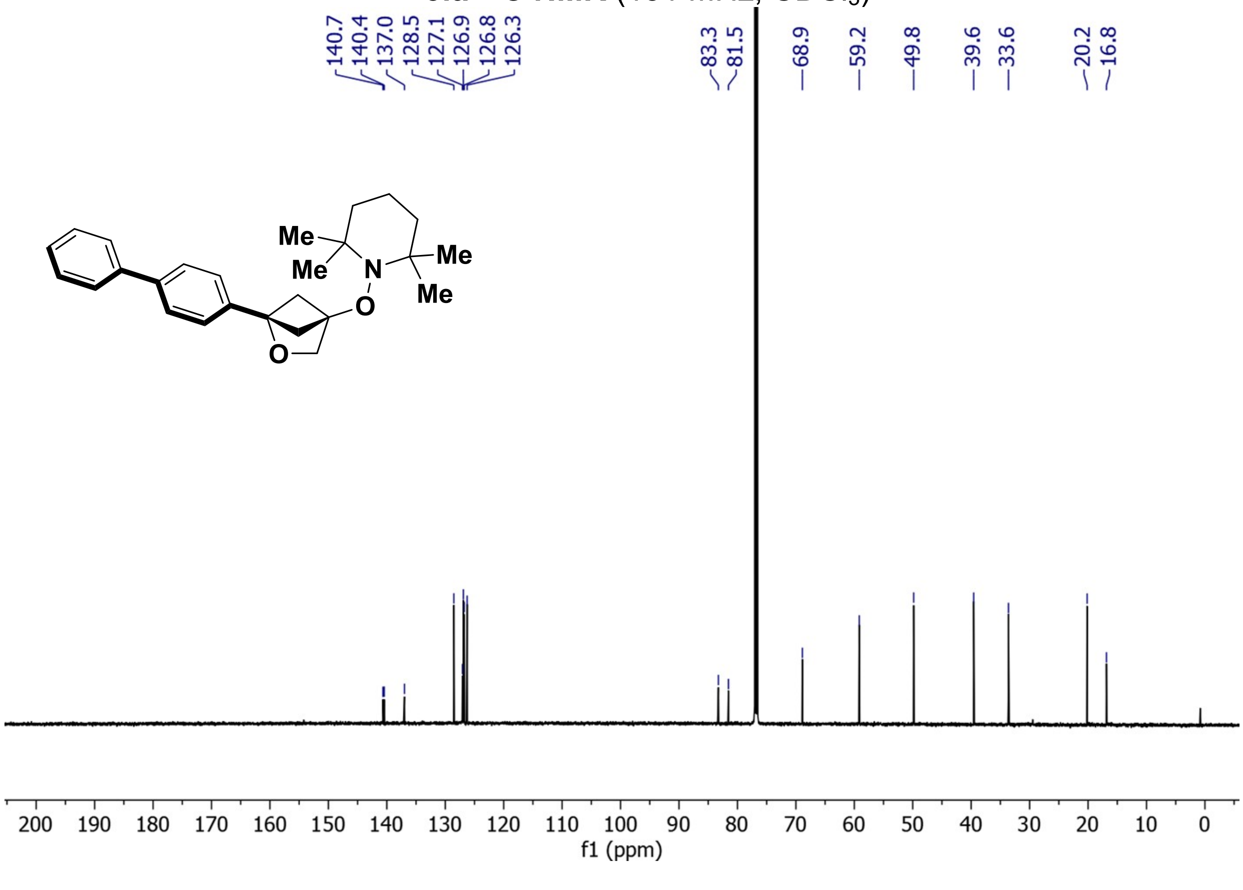

3ja ^{1}H NMR (600 MHz, $CDCl_3$)

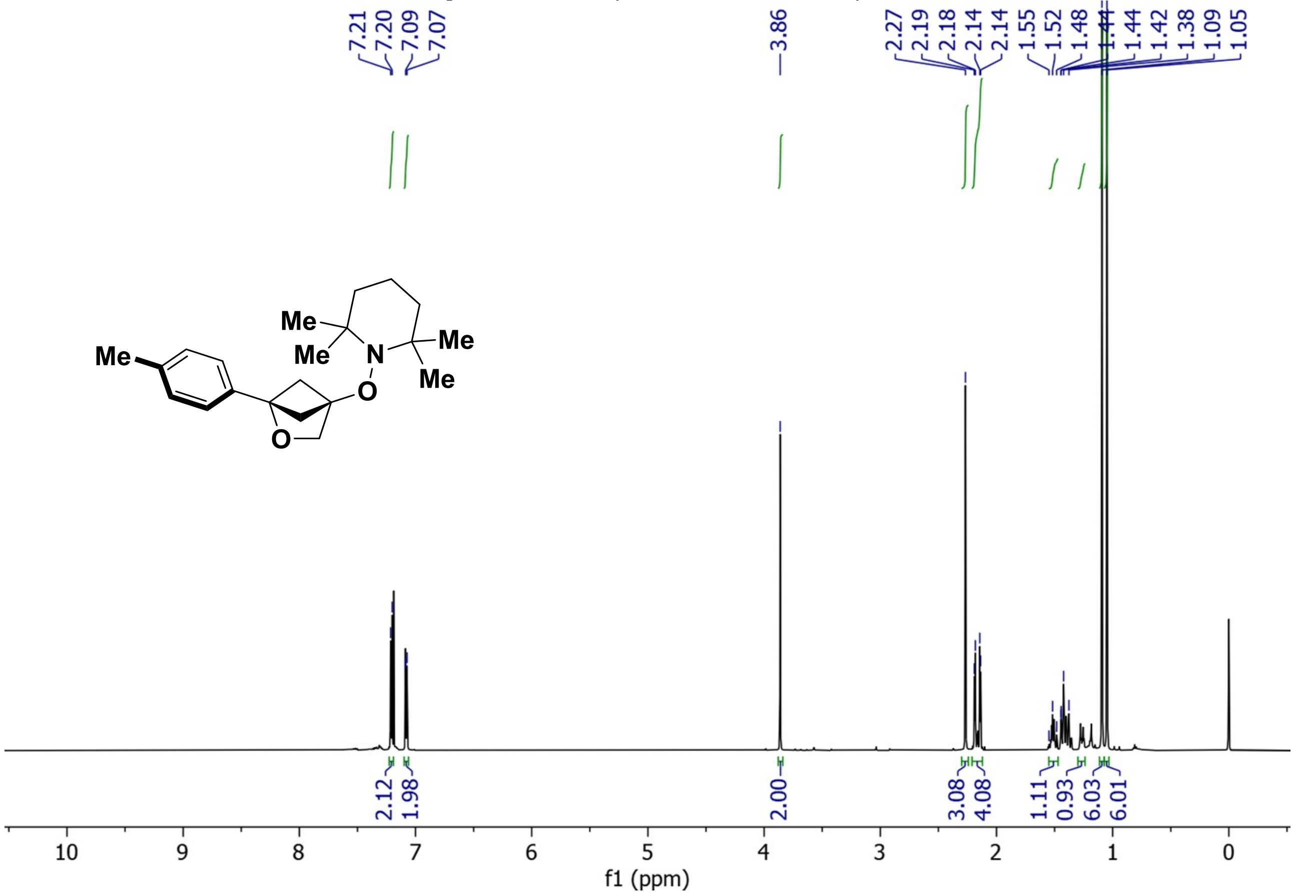

3ja ^{13}C NMR (151 MHz, $CDCl_3$)

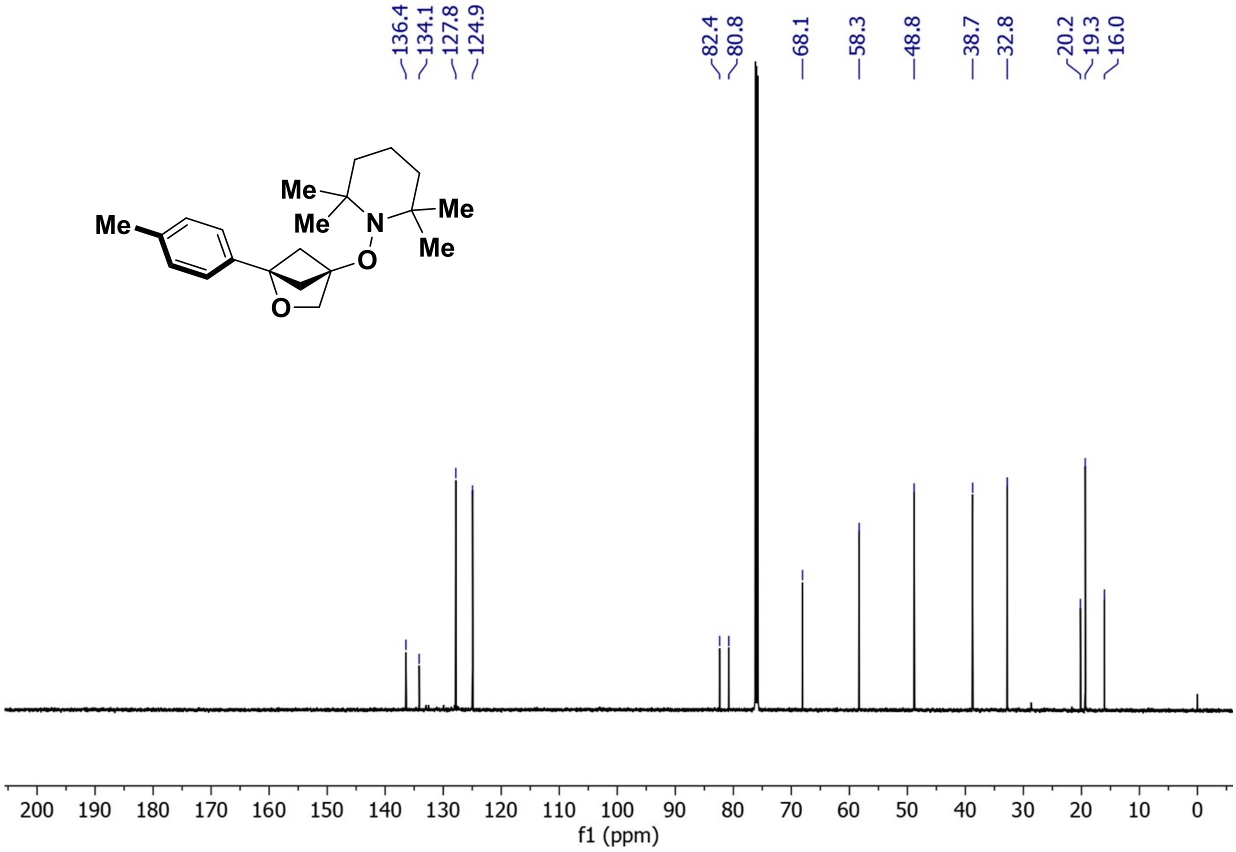

3ka ^{1}H NMR (600 MHz, $CDCl_3$)

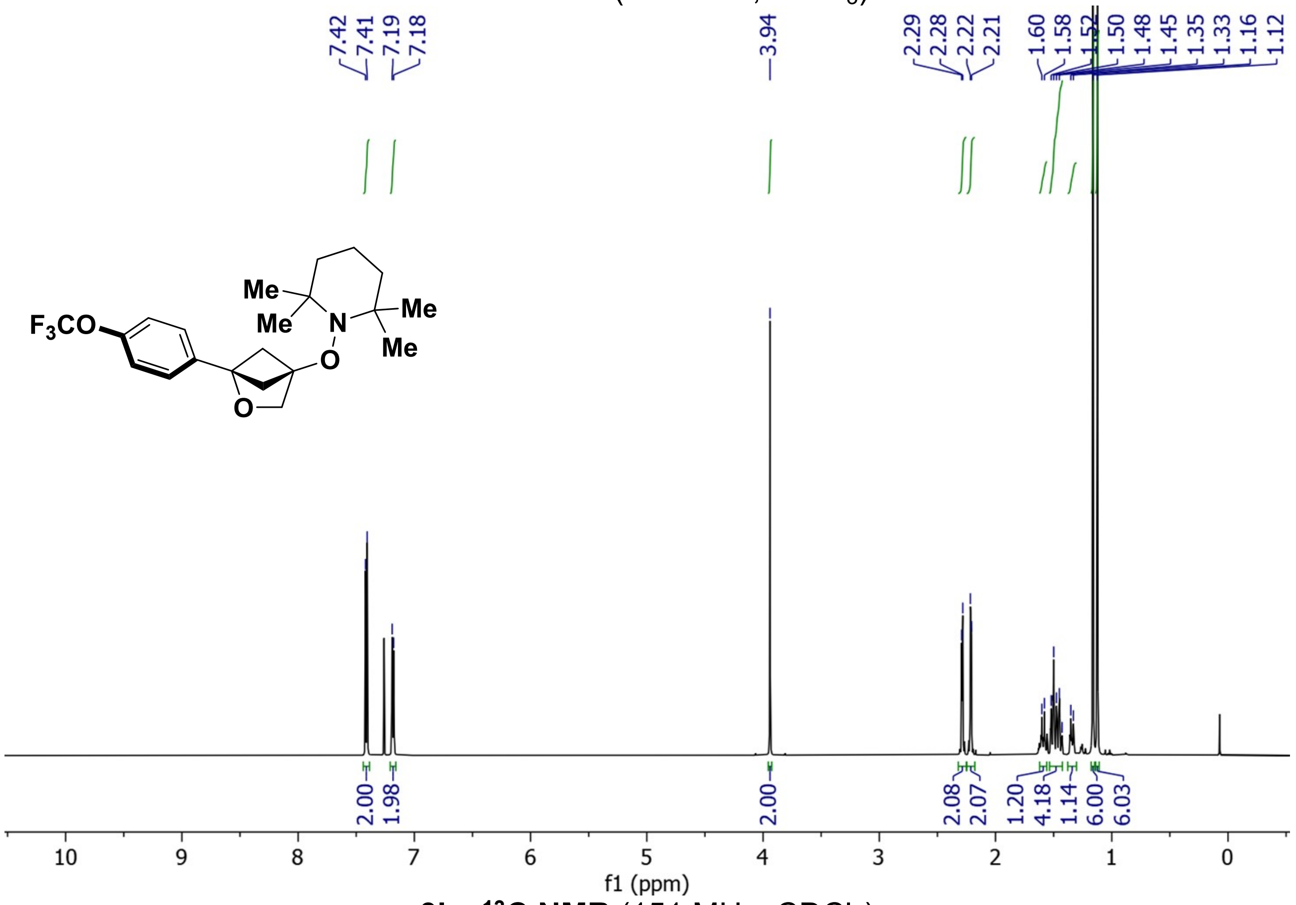

3ka ^{13}C NMR (151 MHz, $CDCl_3$)

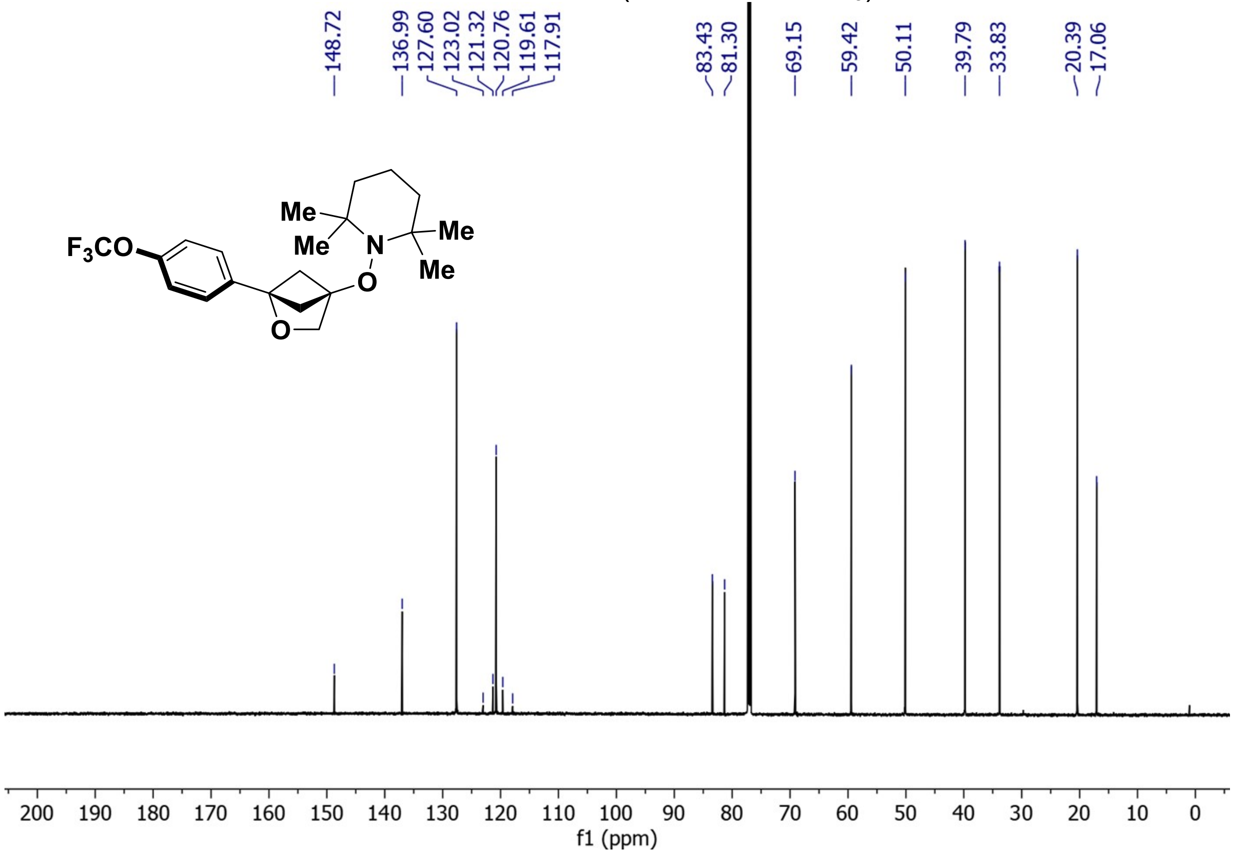

3ka ^{19}F NMR (565 MHz, $CDCl_3$)

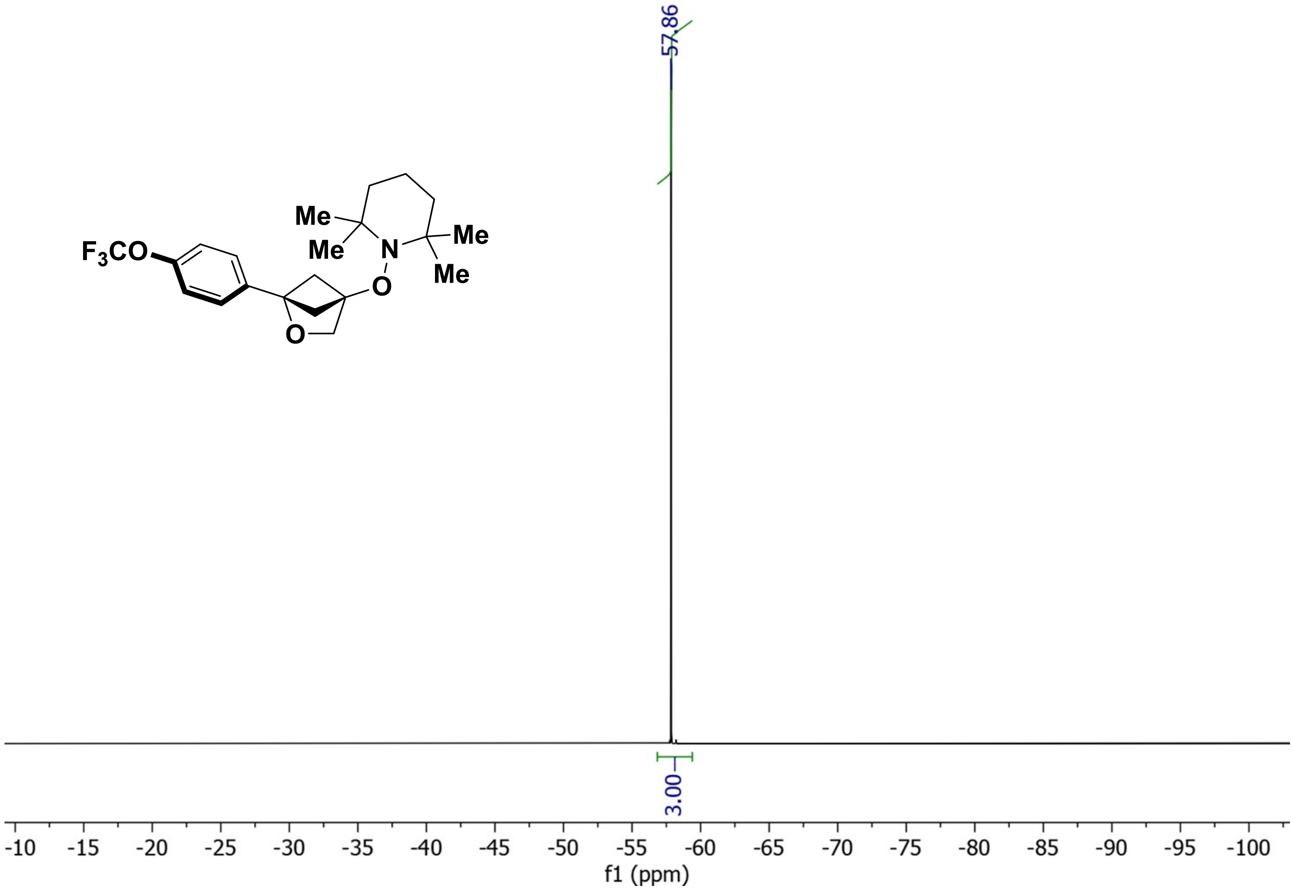

3la ^{1}H NMR (600 MHz, $CDCl_3$)

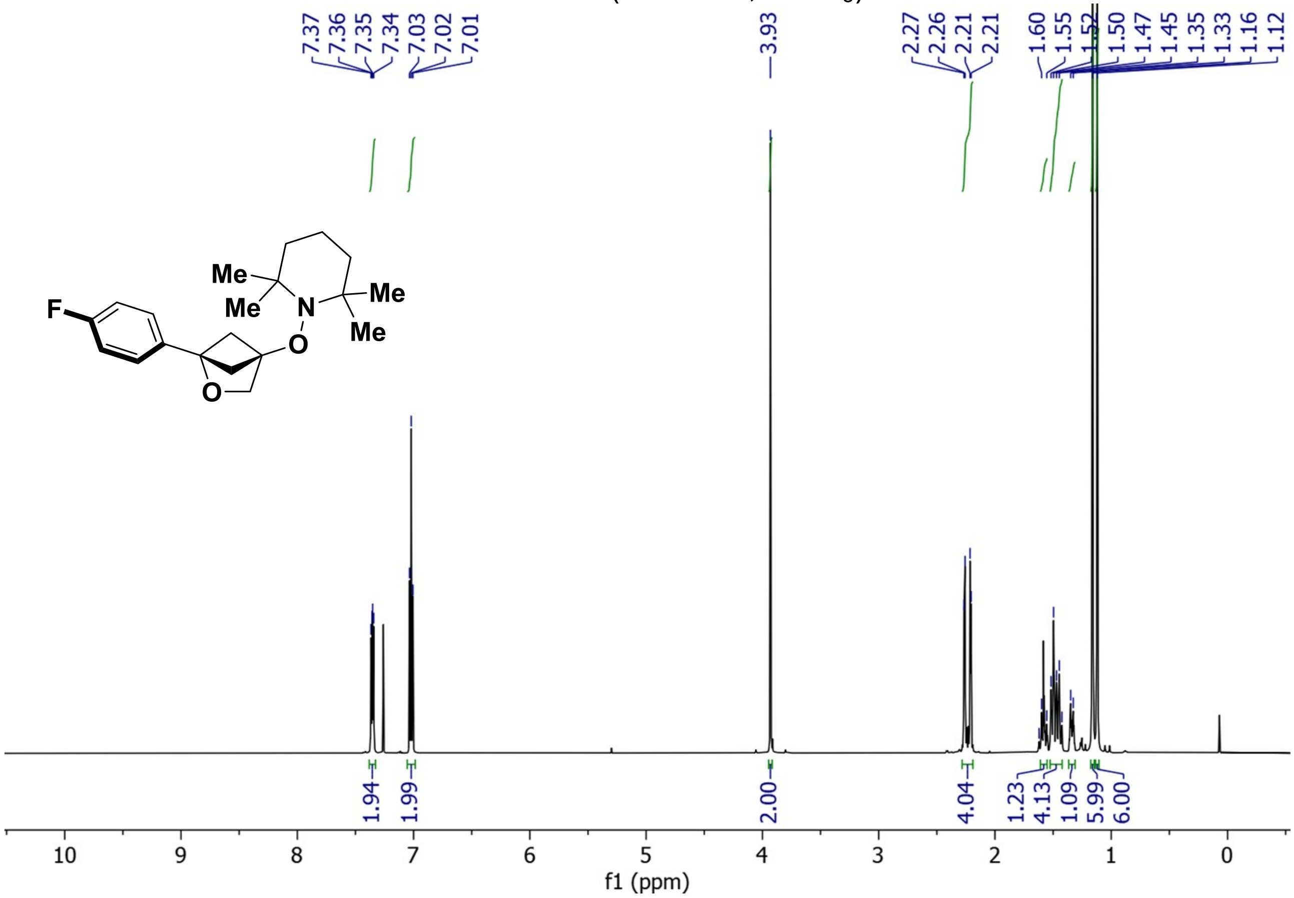

3la ^{13}C NMR (151 MHz, $CDCl_3$)

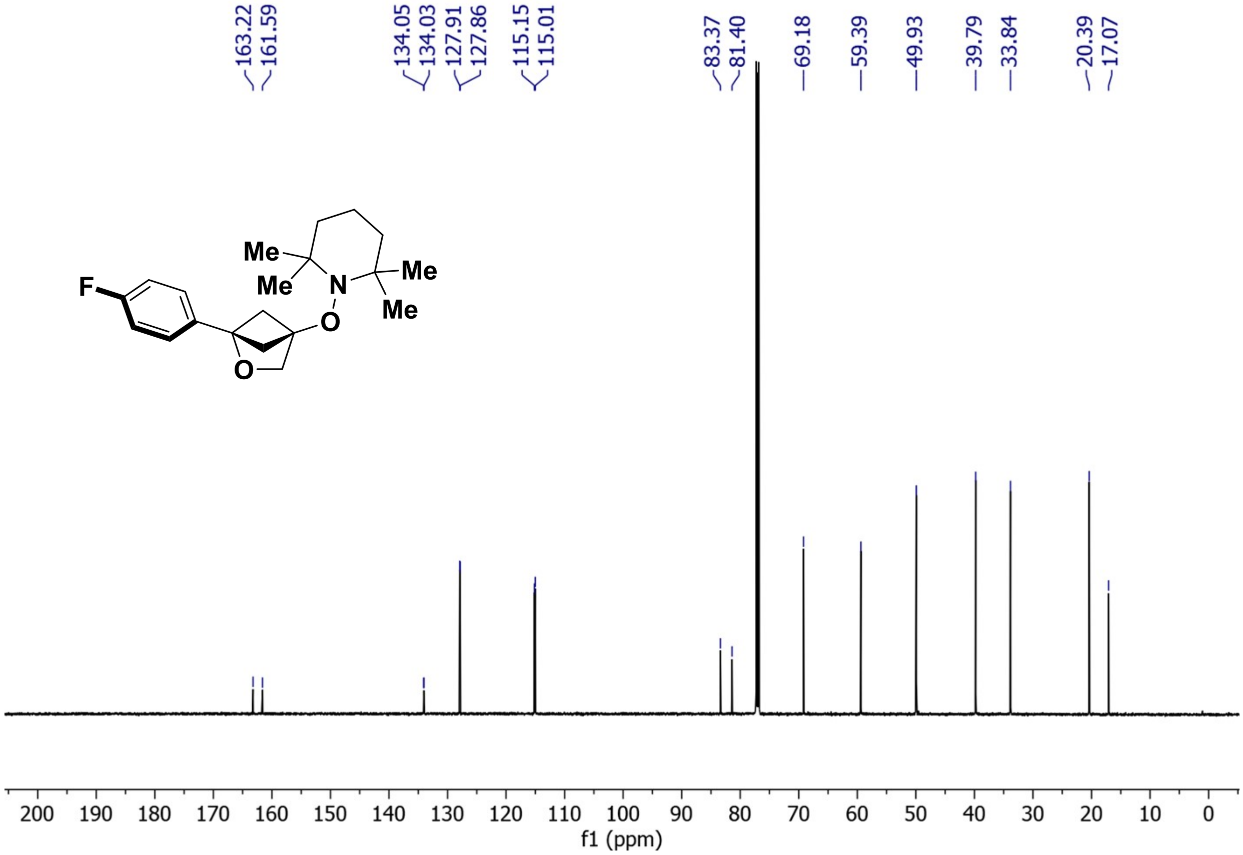

3la ^{19}F NMR (565 MHz, $CDCl_3$)

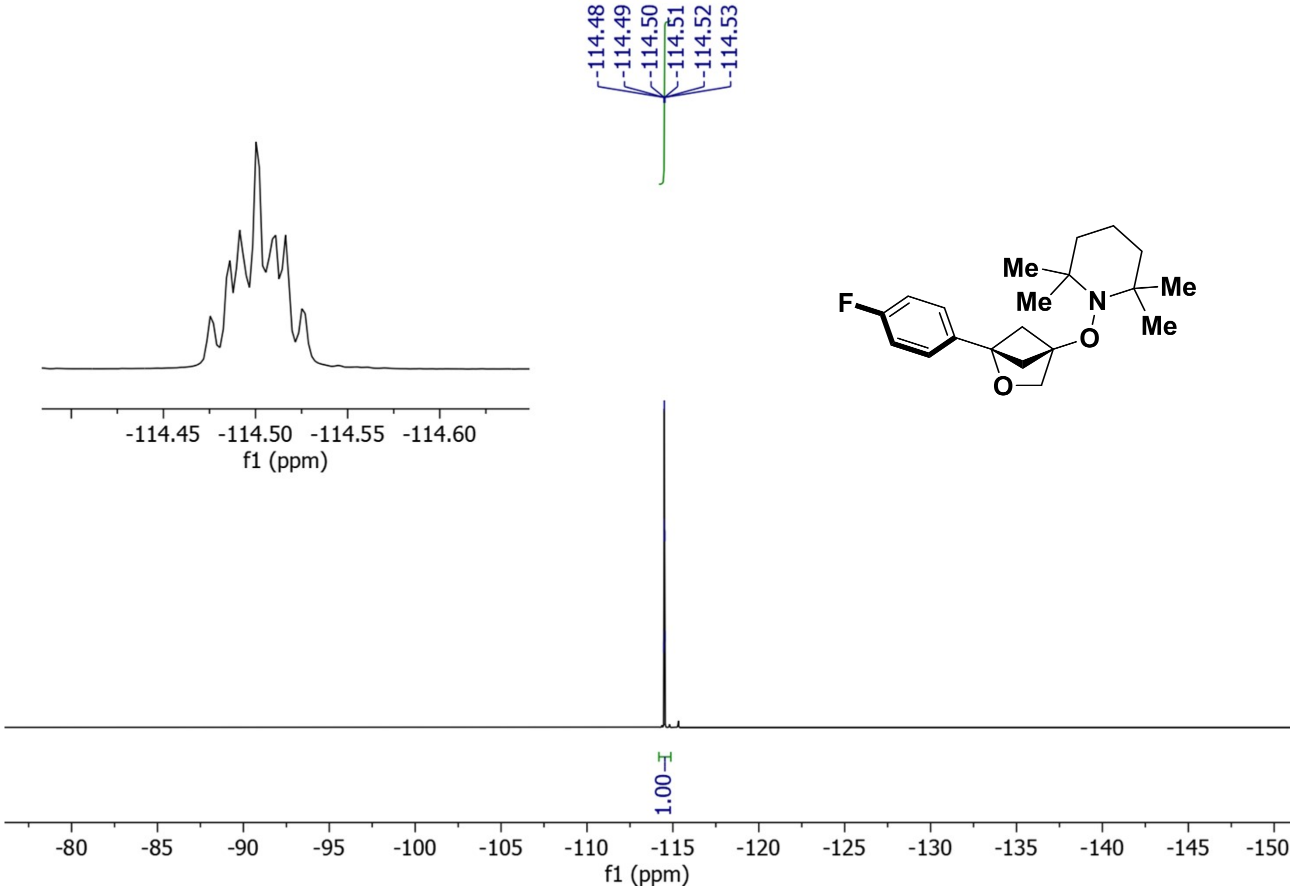

3ma ^{1}H NMR (600 MHz, $CDCl_3$)

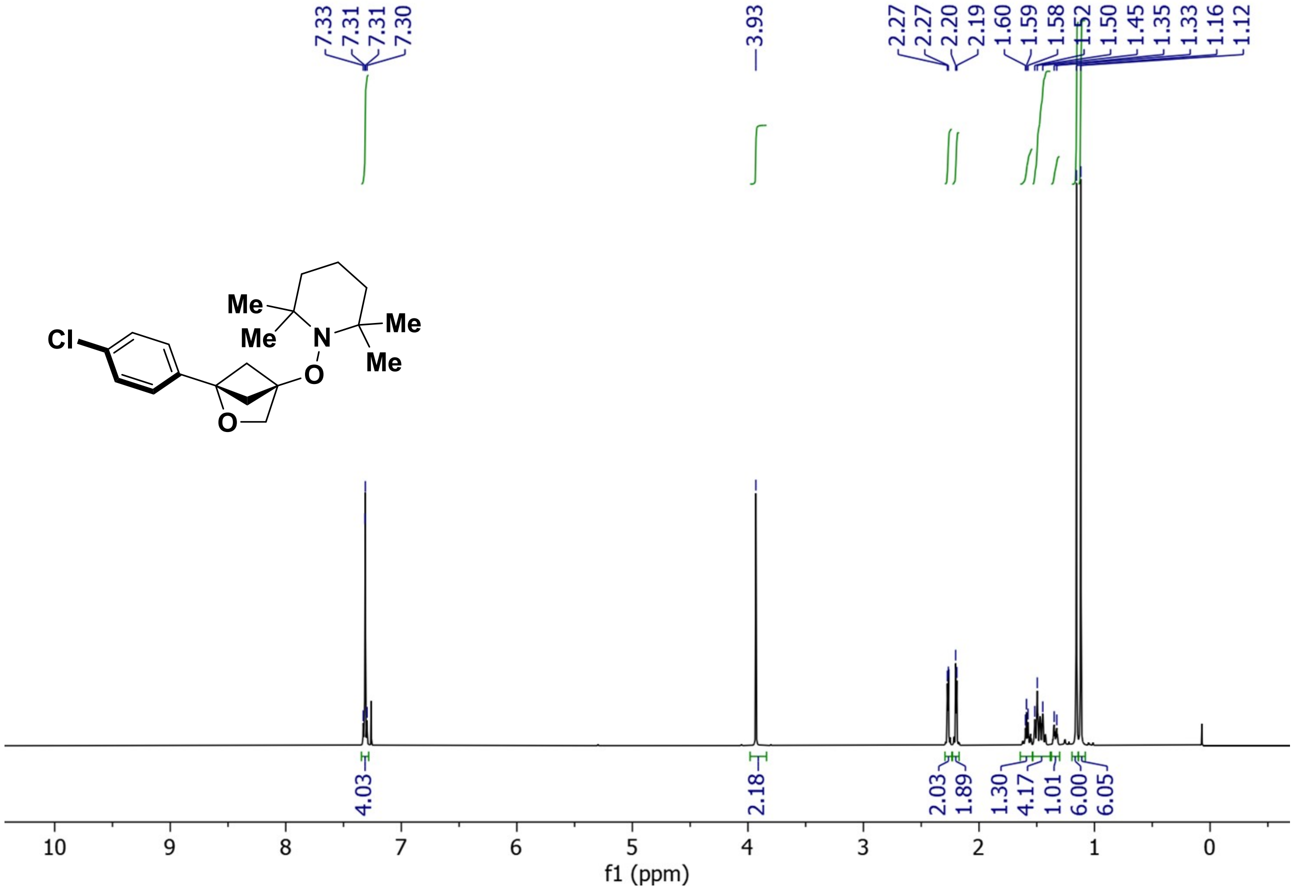

3ma ^{13}C NMR (151 MHz, $CDCl_3$)

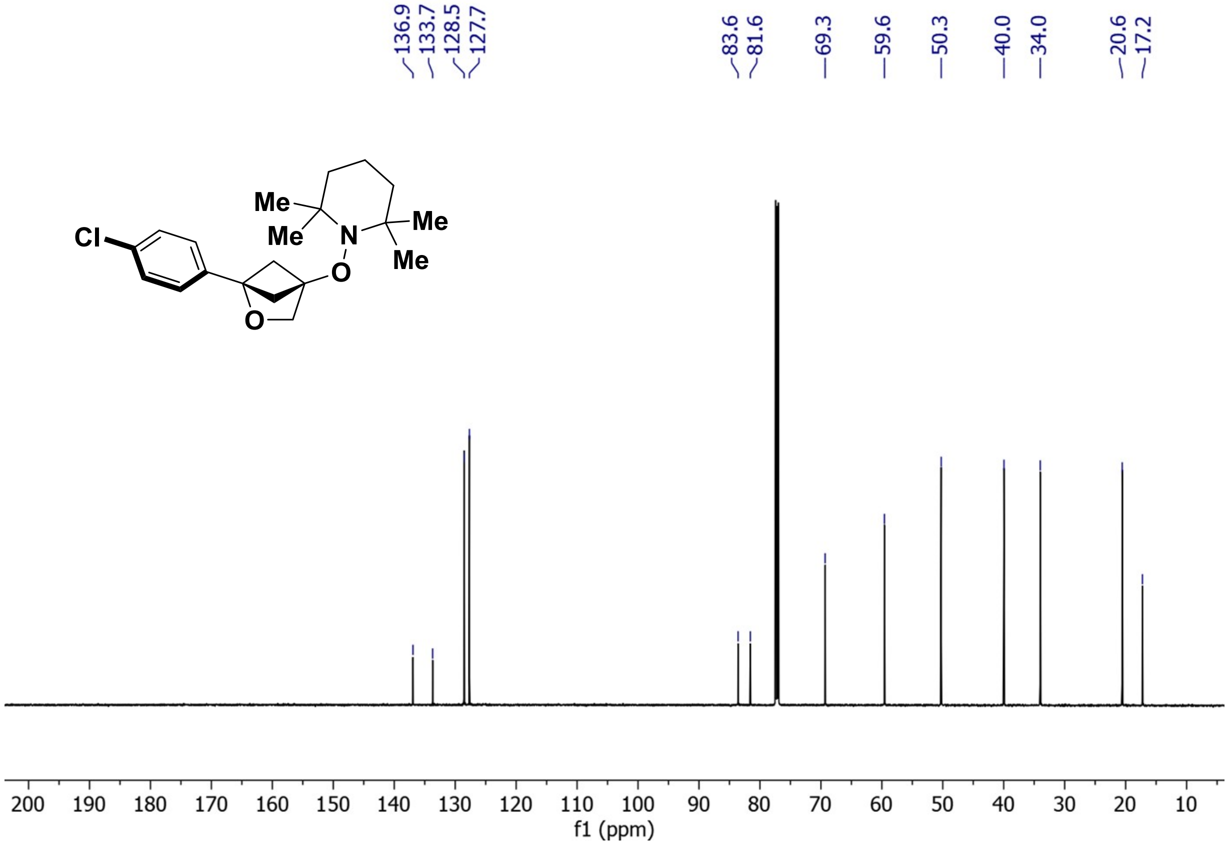

3na ^{1}H NMR (600 MHz, $CDCl_3$)

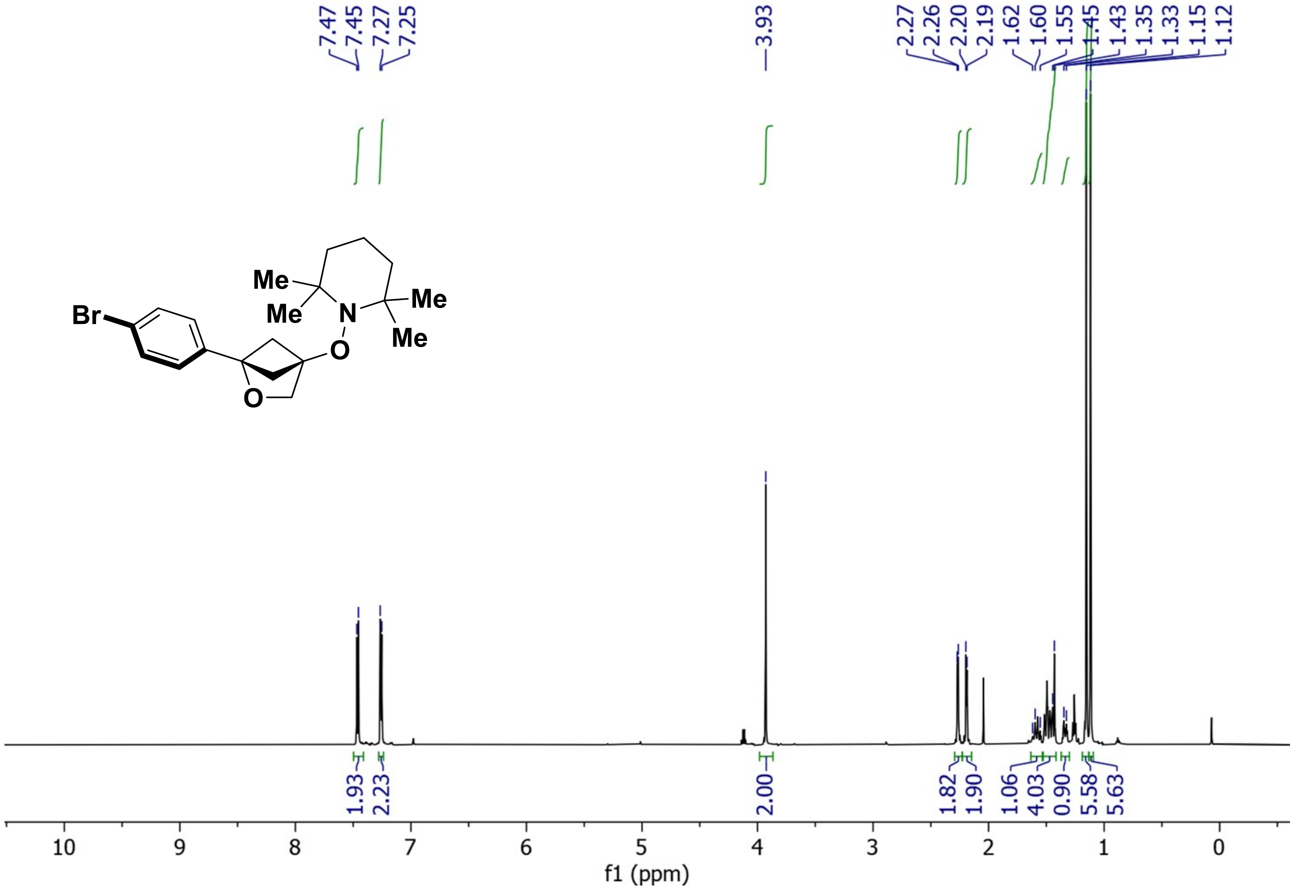

3na ^{13}C NMR (151 MHz, $CDCl_3$)

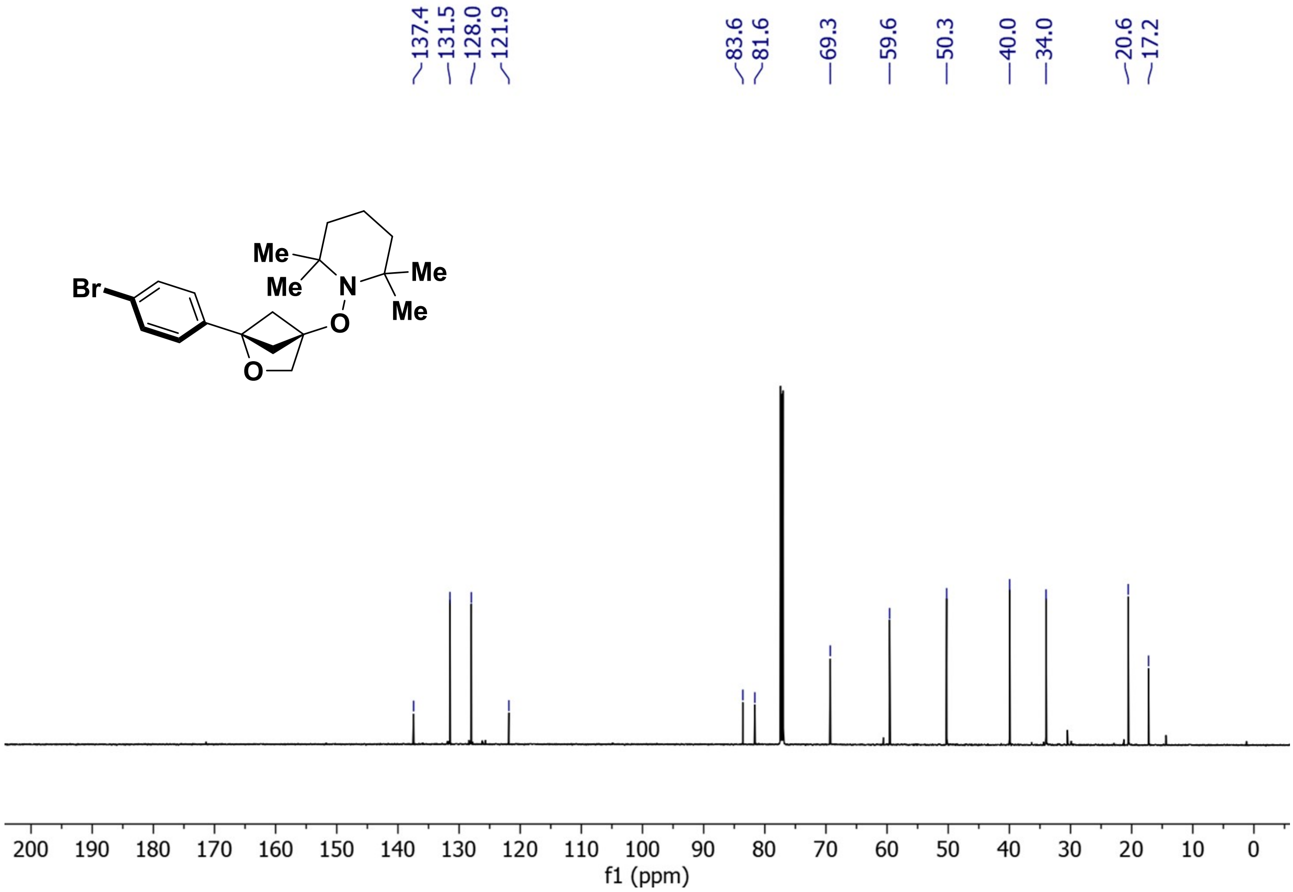

3oa ^{1}H NMR (600 MHz, $CDCl_3$)

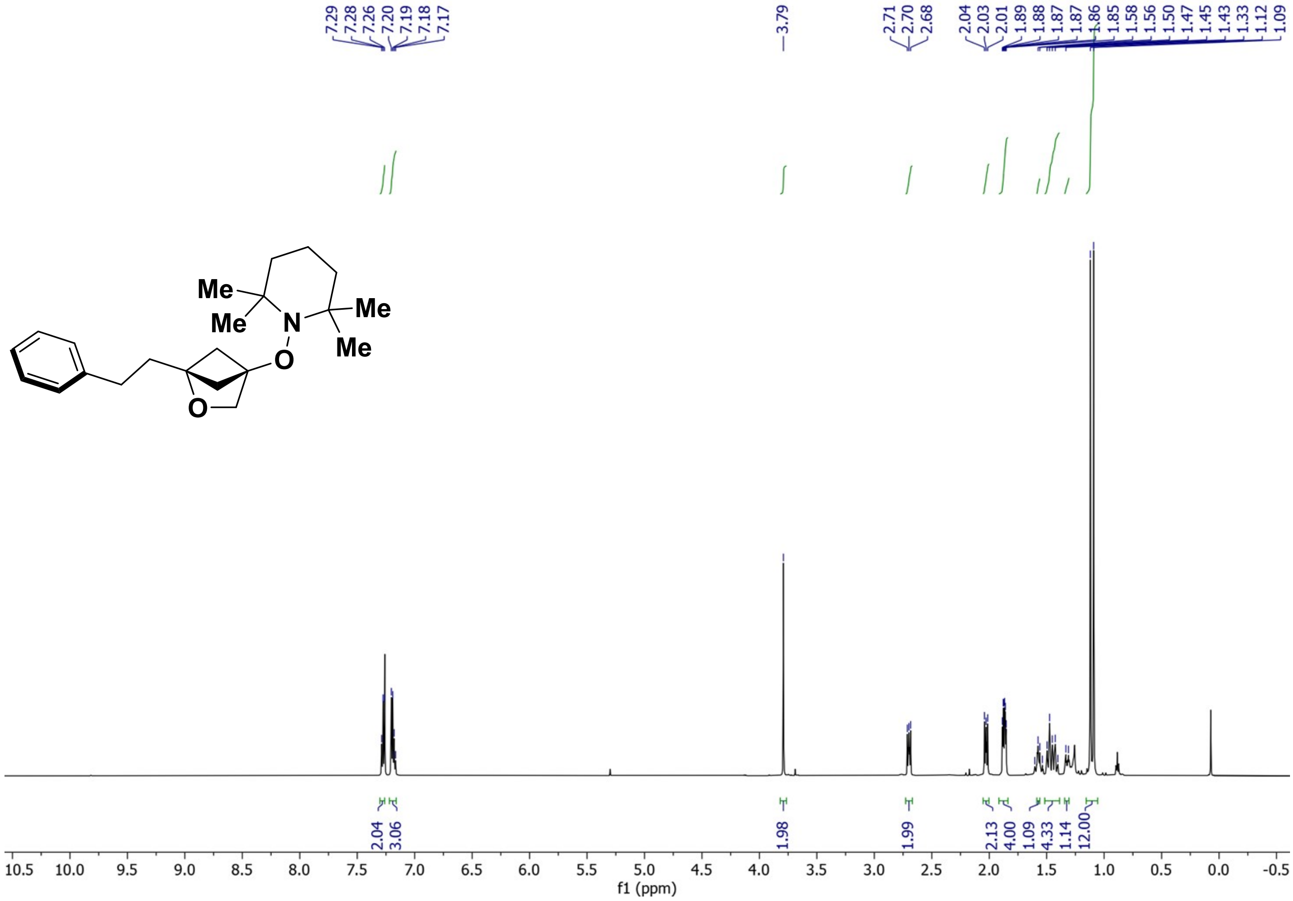

3oa ^{13}C NMR (151 MHz, $CDCl_3$)

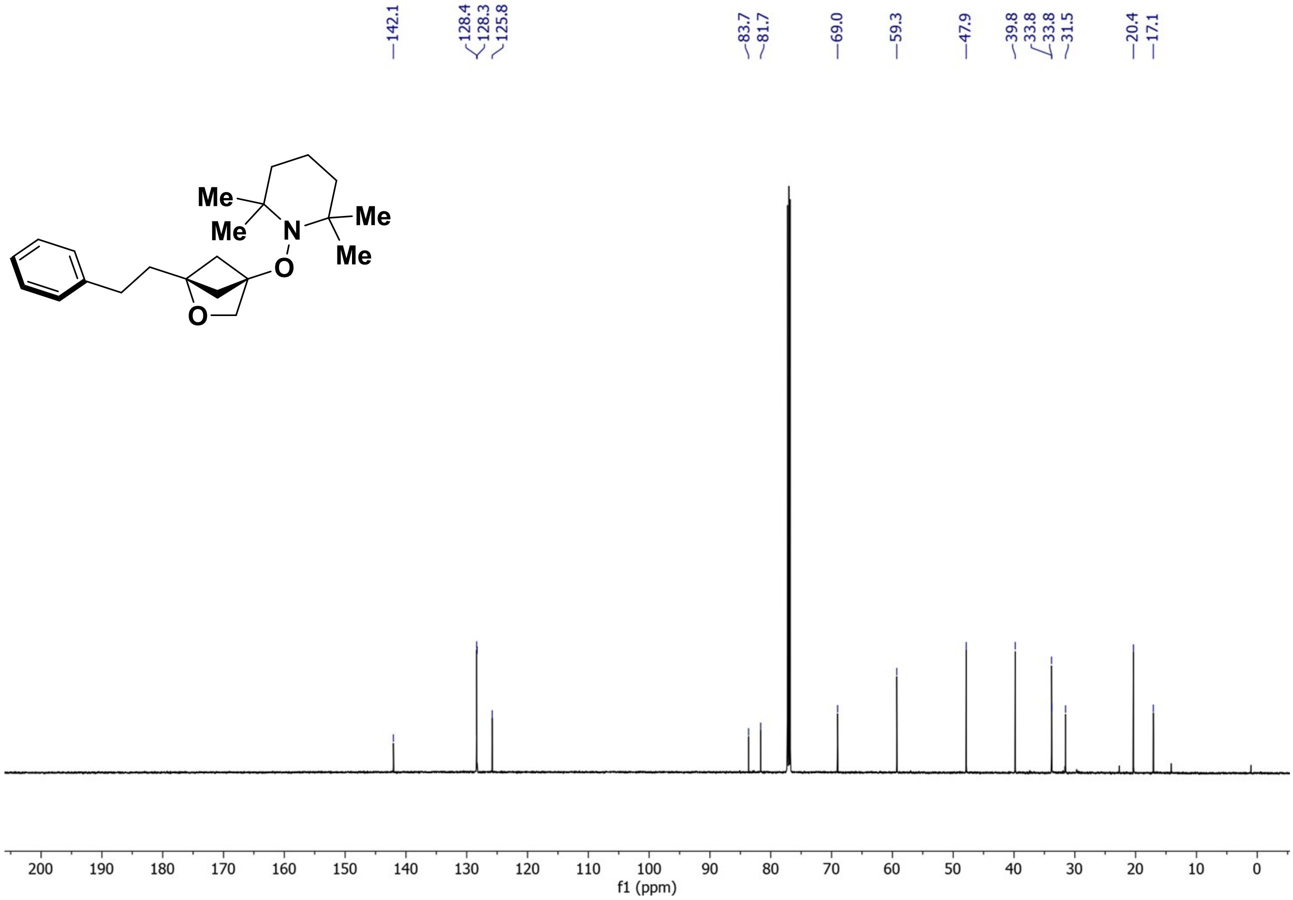

3pa ^{1}H NMR (600 MHz, $CDCl_3$)

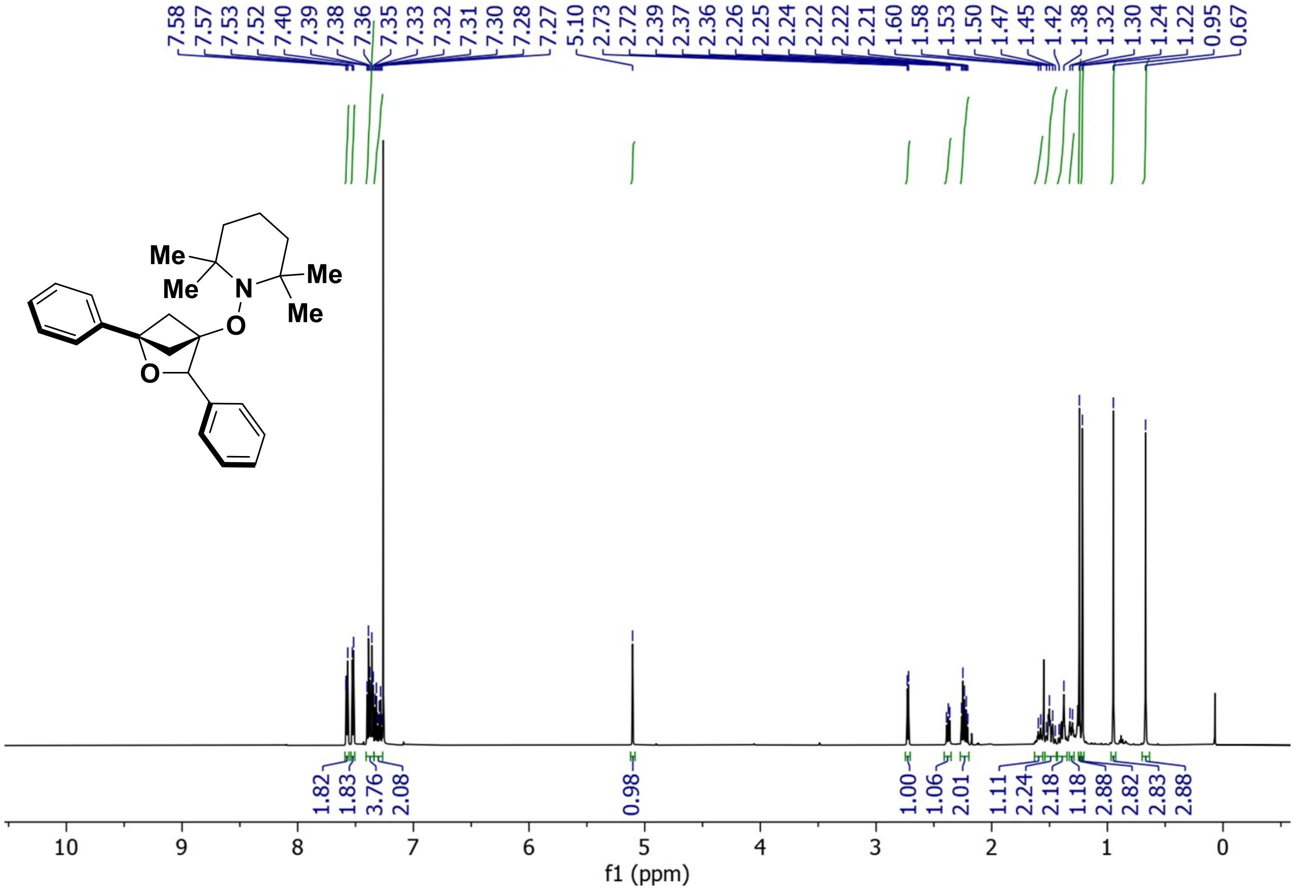

3pa ^{13}C NMR (151 MHz, $CDCl_3$)

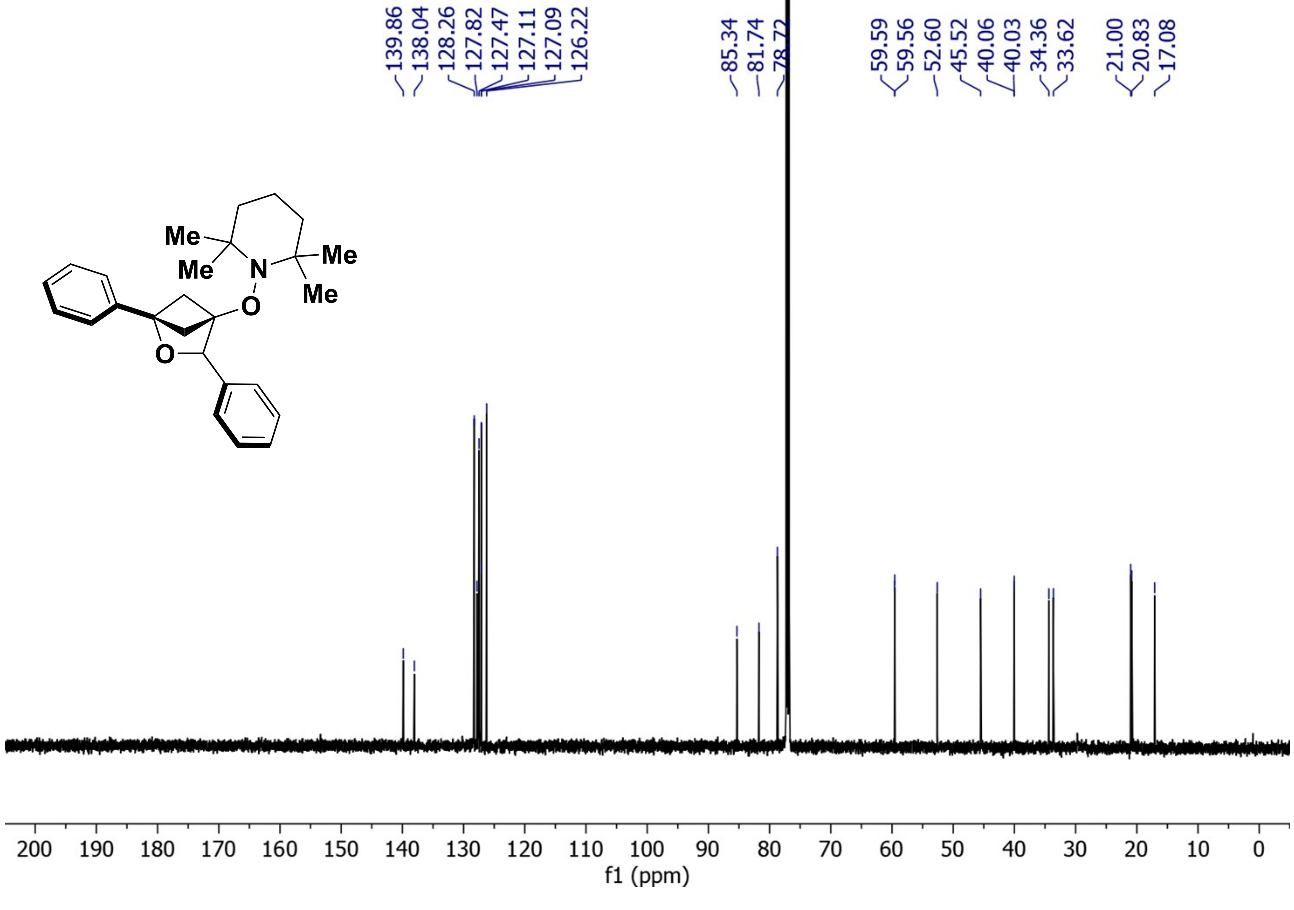

3qa ^{1}**H NMR** (600 MHz, $CDCl_3$)

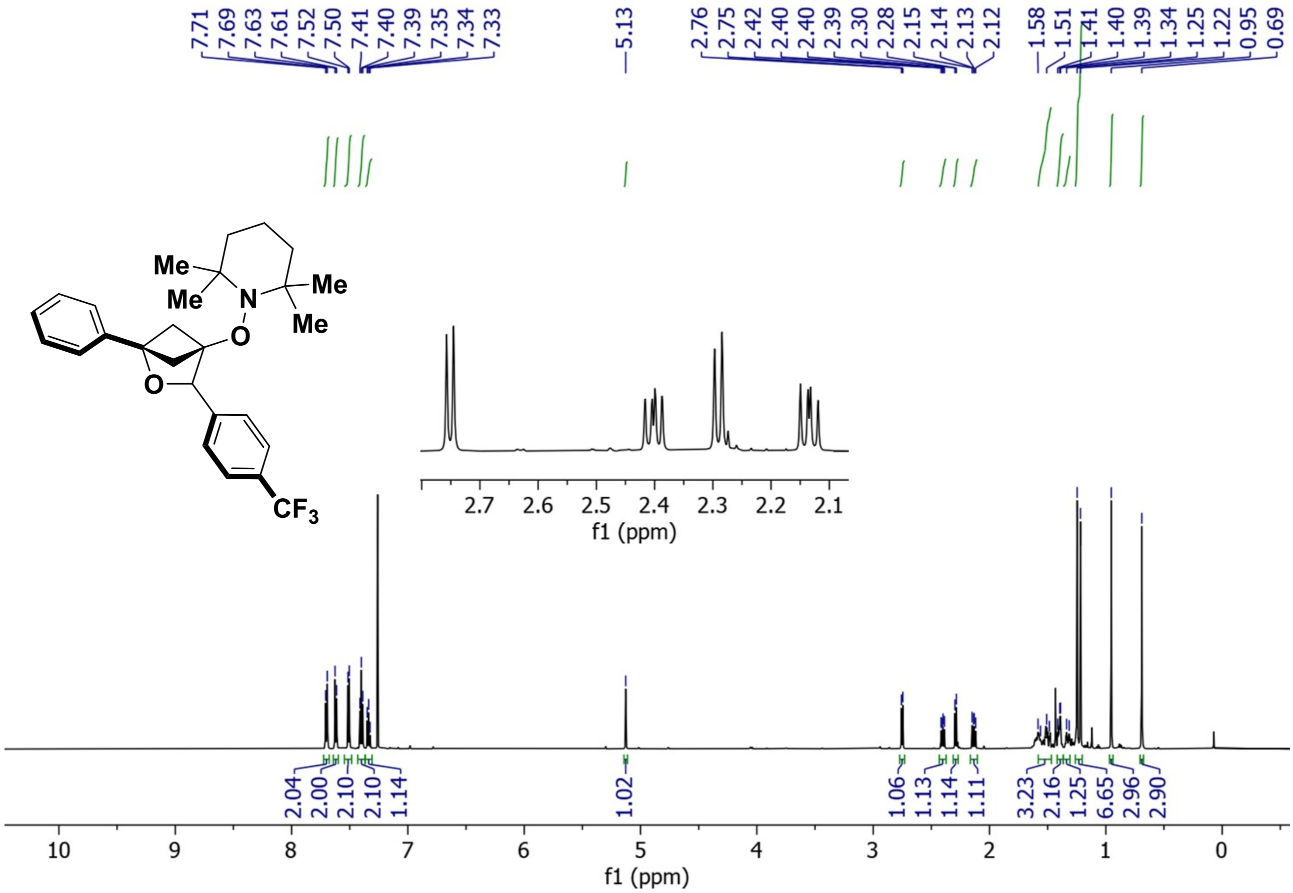

3qa ^{13}**C NMR** (151 MHz, $CDCl_3$)

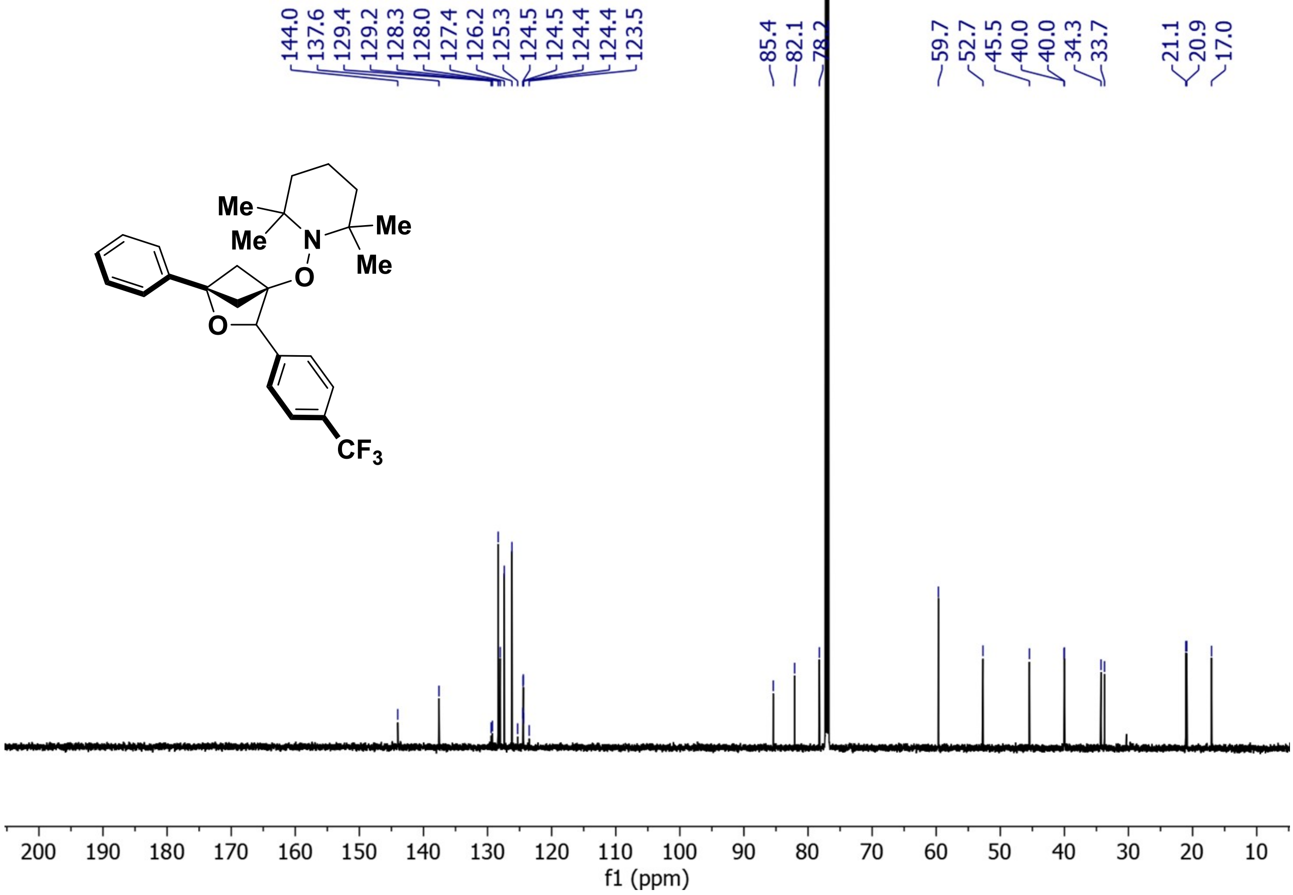

3qa ^{19}F NMR (565 MHz, $CDCl_3$)

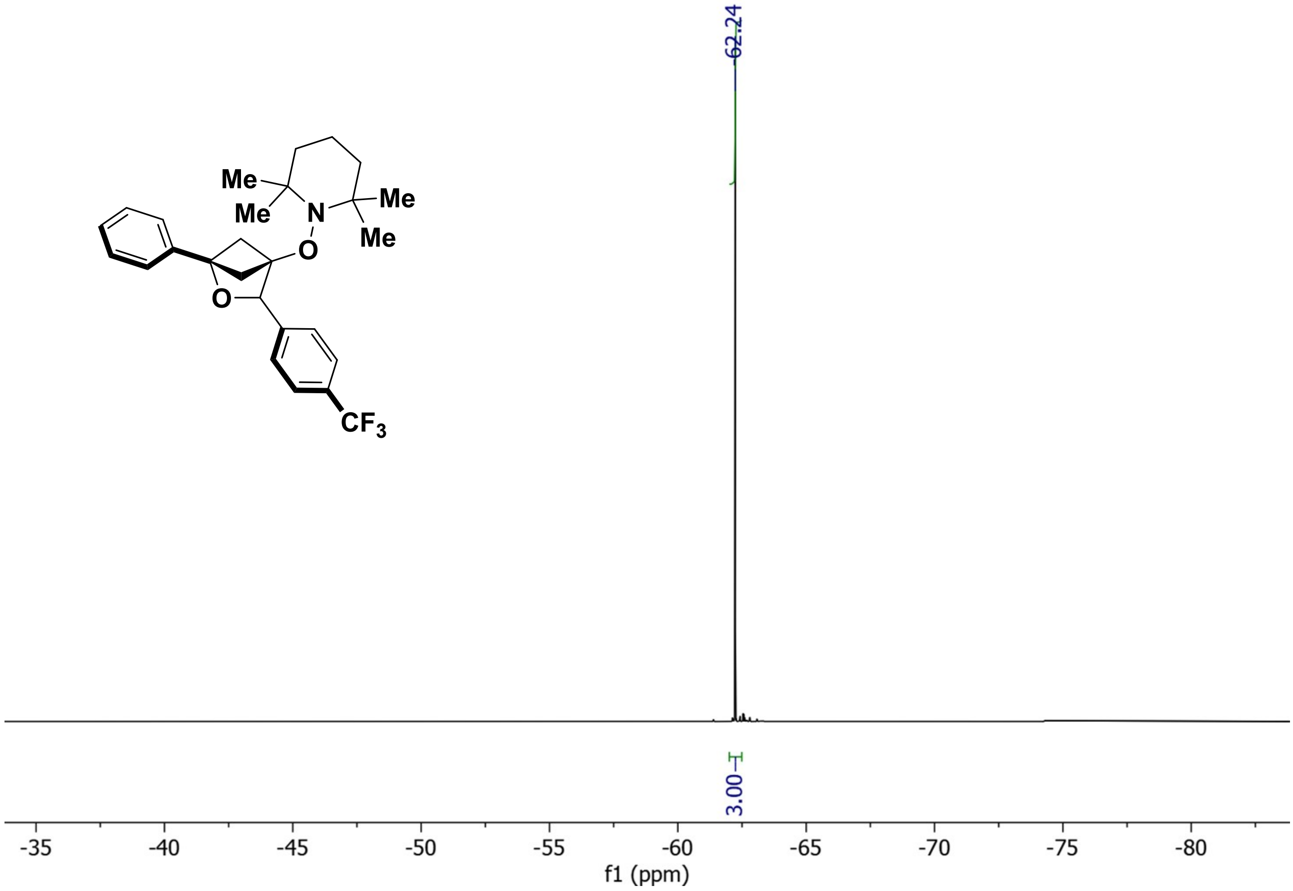

3ra ^{1}H NMR (600 MHz, $CDCl_3$)

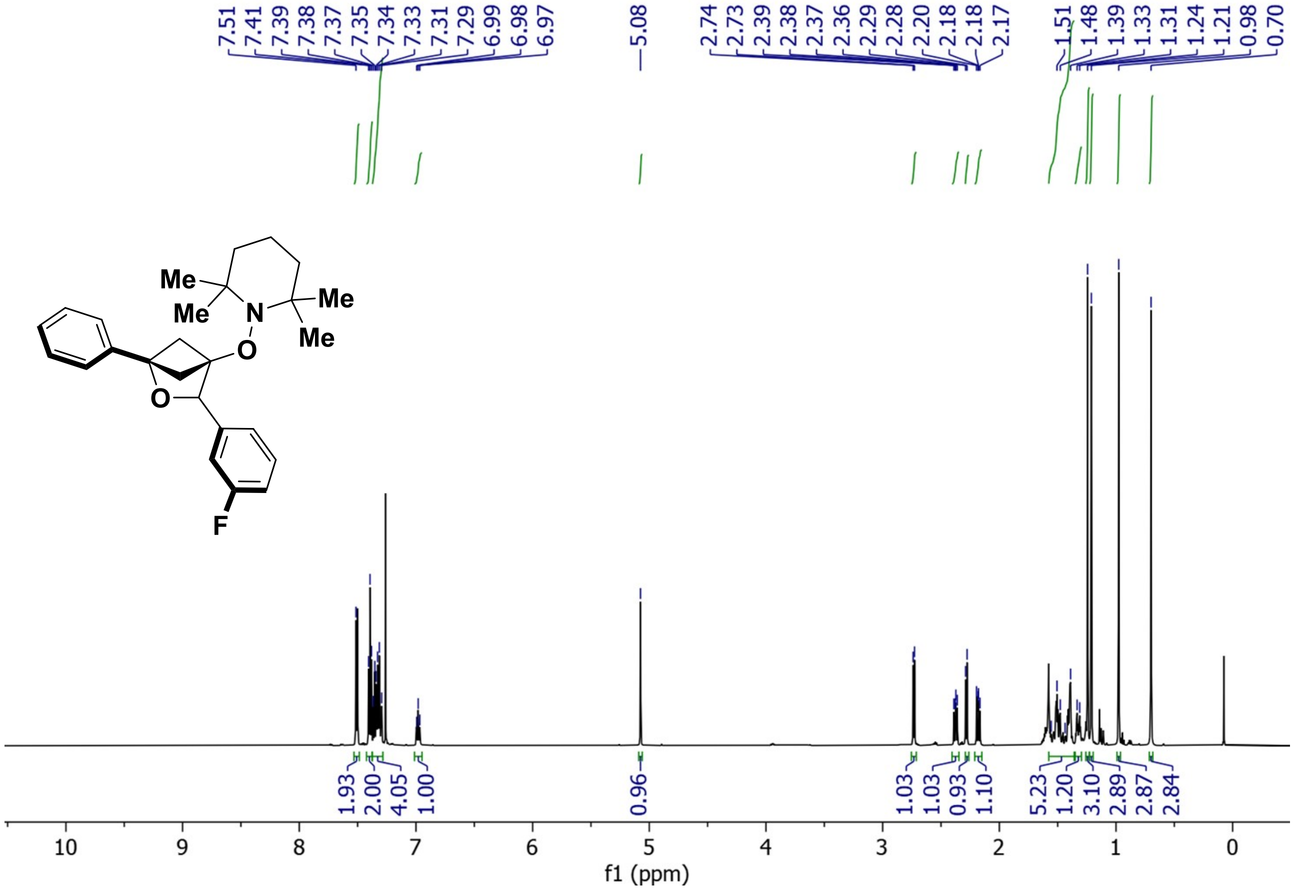

3ra ^{13}C NMR (151 MHz, $CDCl_3$)

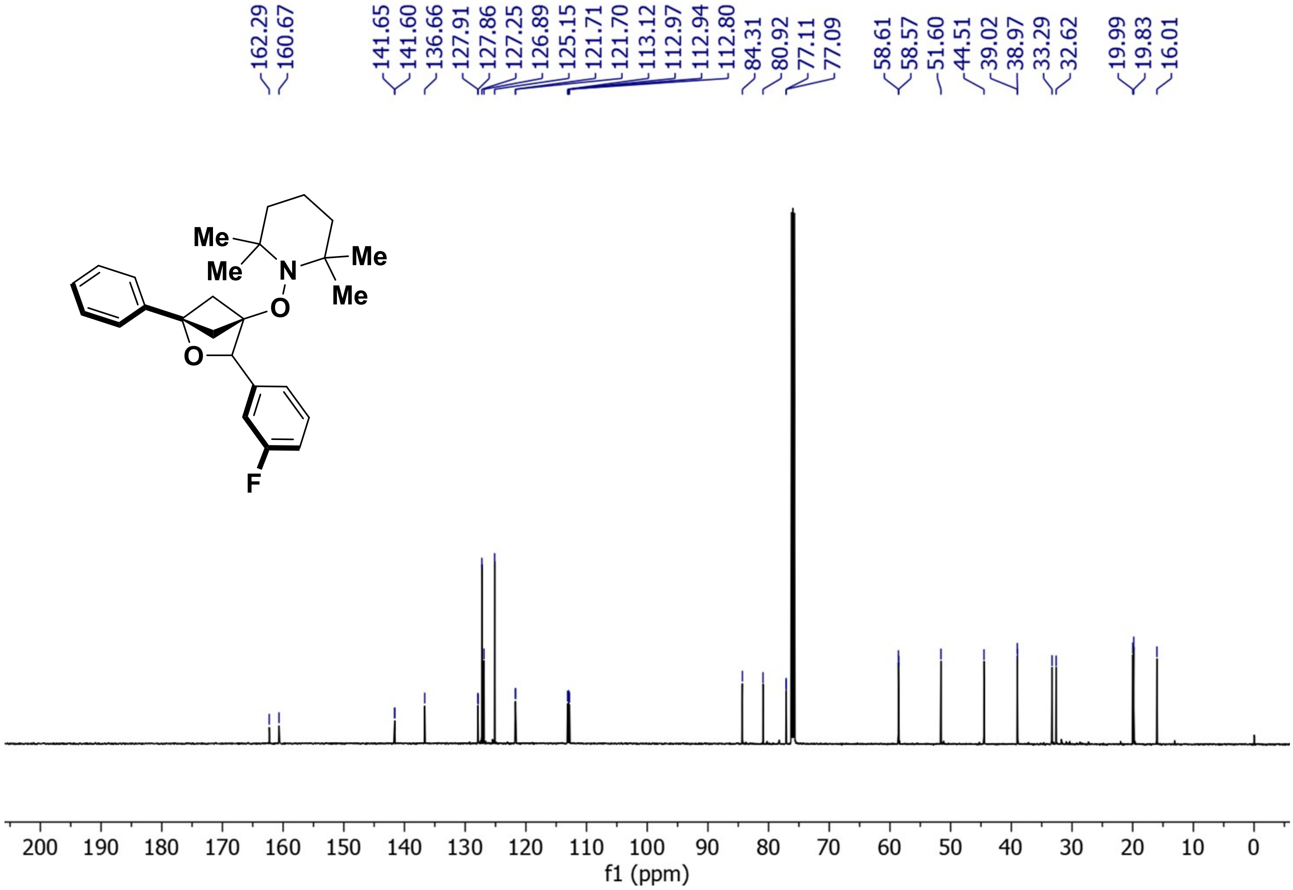

3ra ^{1}H NMR (565 MHz, $CDCl_3$)

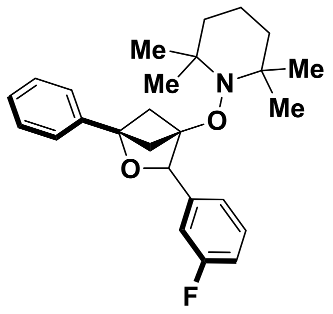

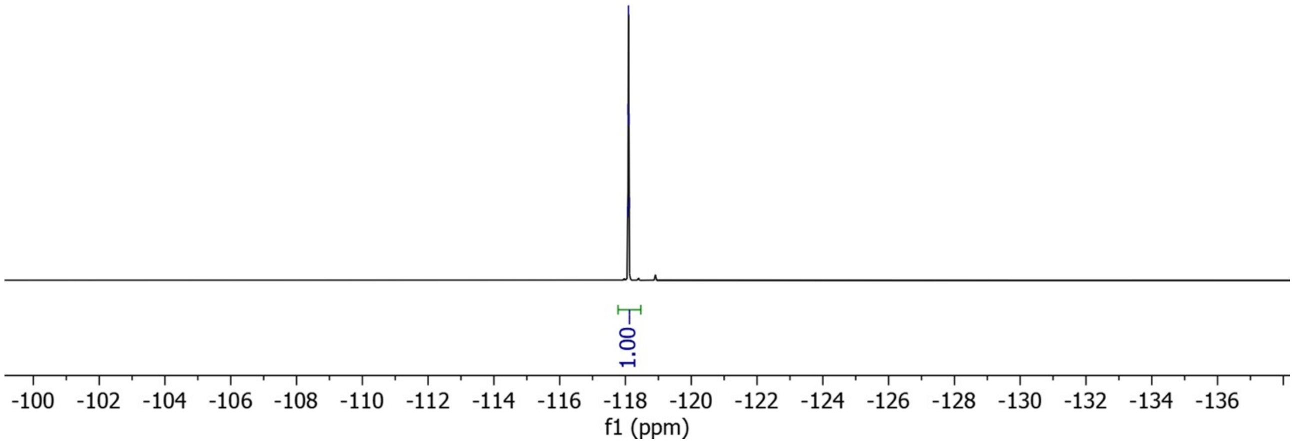

3sa ^{1}H NMR (600 MHz, $CDCl_3$)

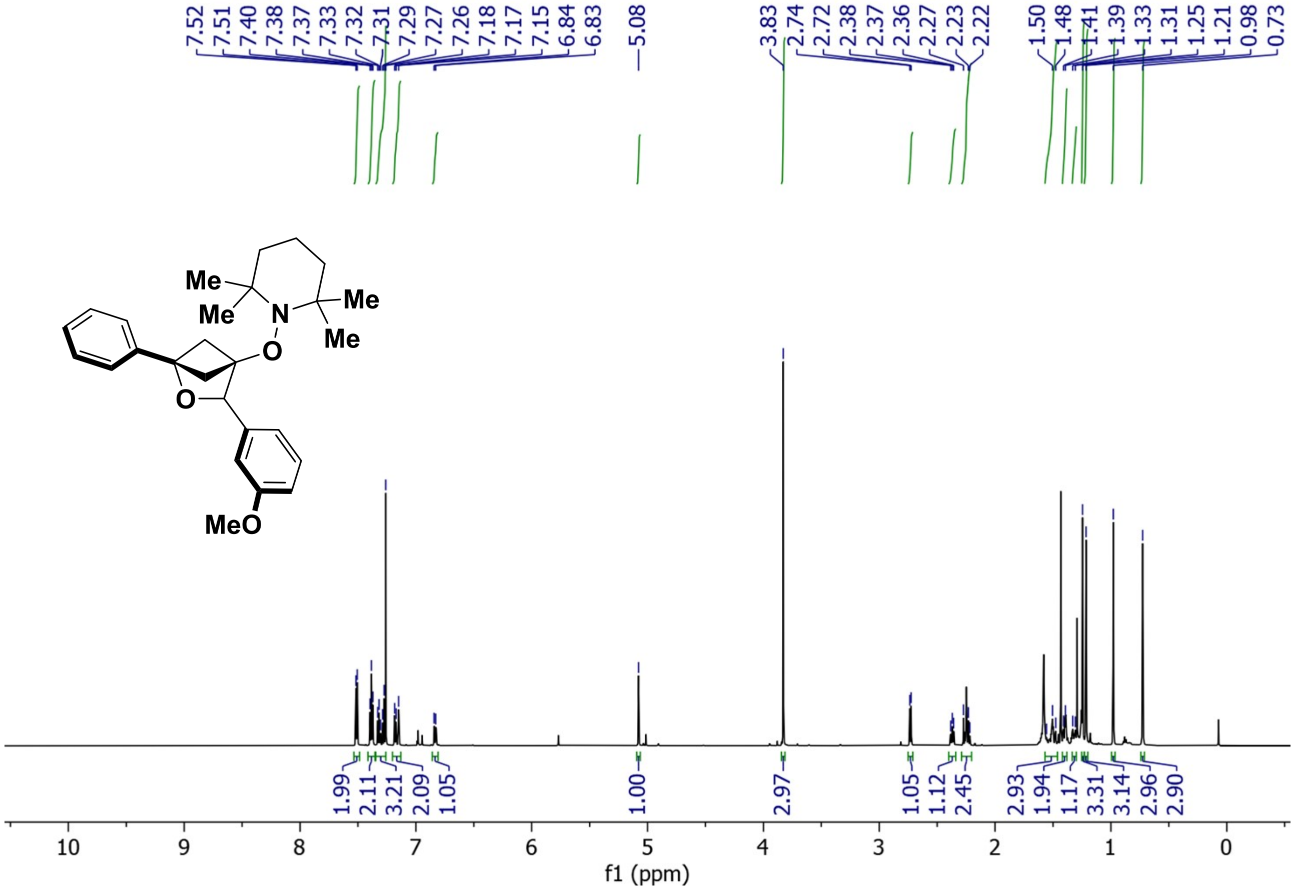

3sa ^{13}C NMR (151 MHz, $CDCl_3$)

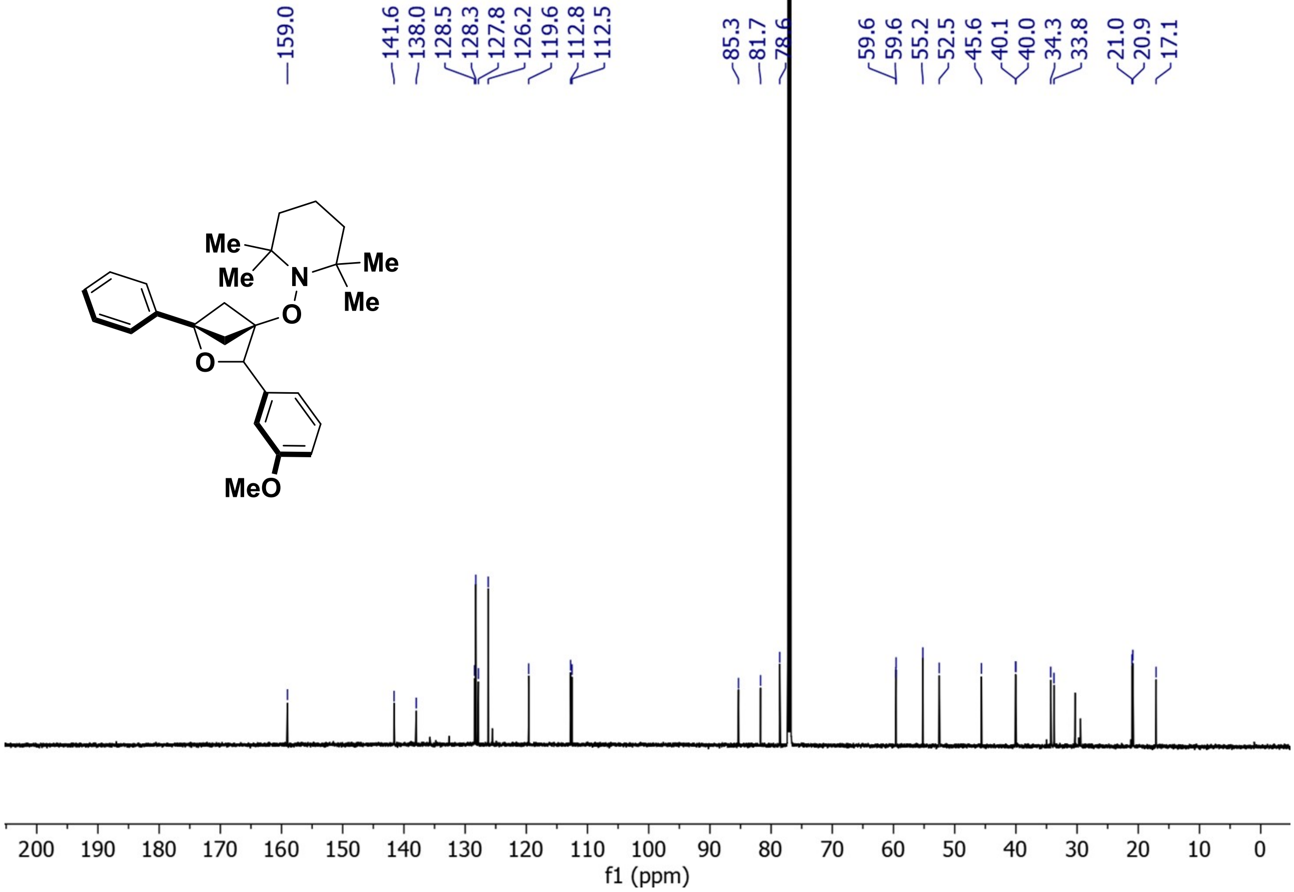

3ta ^{1}H NMR (600 MHz, $CDCl_3$)

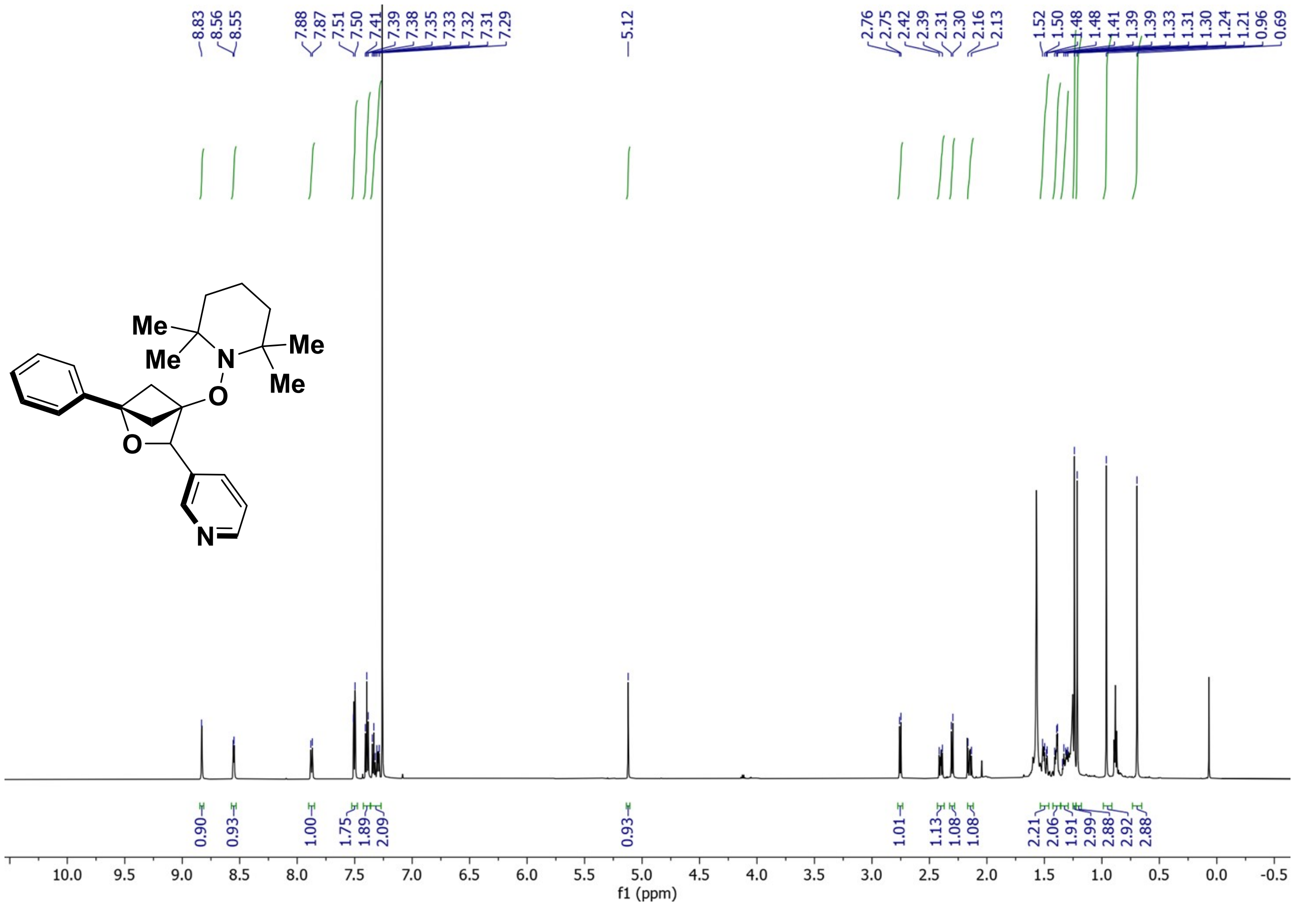

3ta ^{13}C NMR (151 MHz, $CDCl_3$)

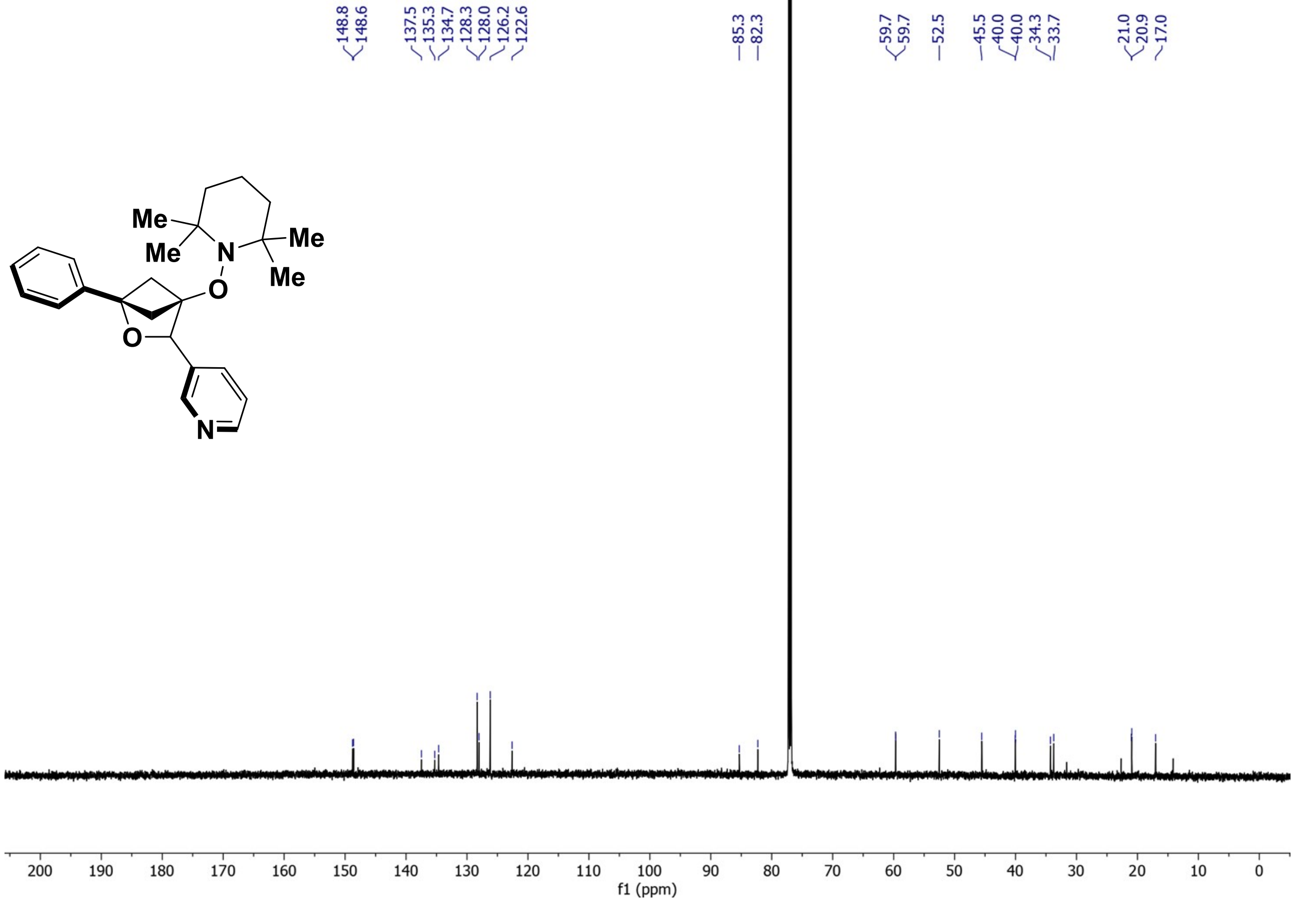

3ua ^{1}H NMR (600 MHz, $CDCl_3$)

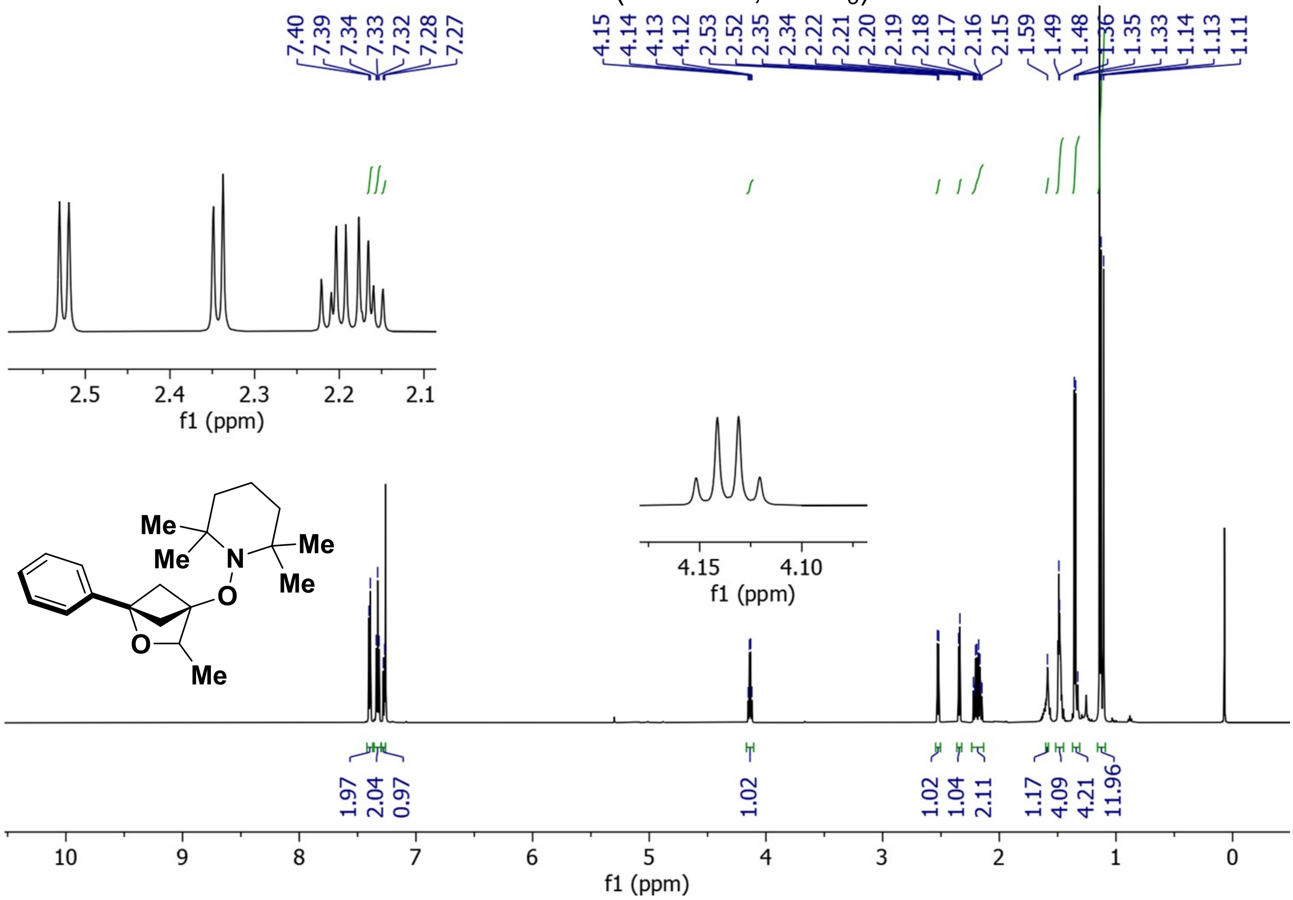

3ua ^{13}C NMR (151 MHz, $CDCl_3$)

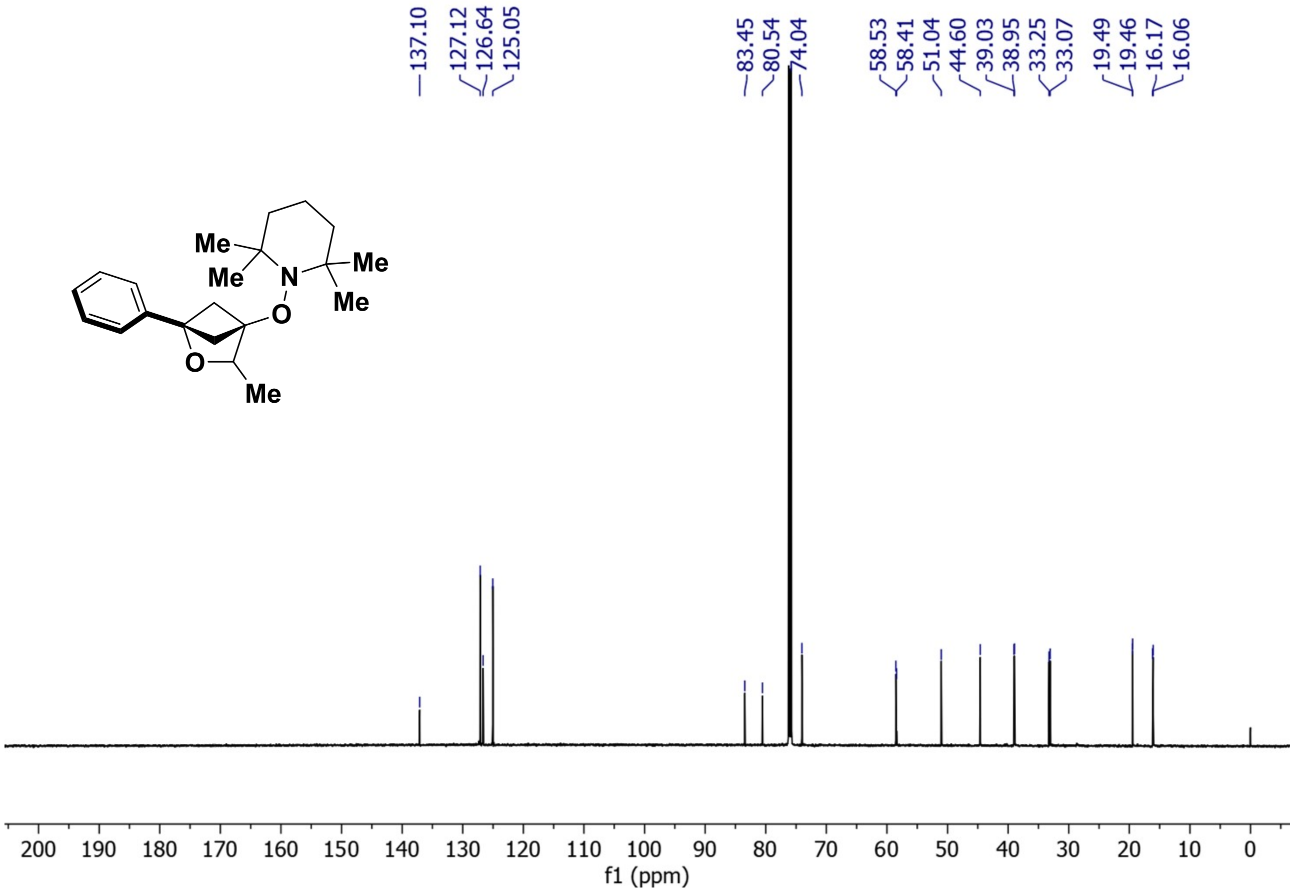

3va ^{1}H NMR (600 MHz, $CDCl_3$)

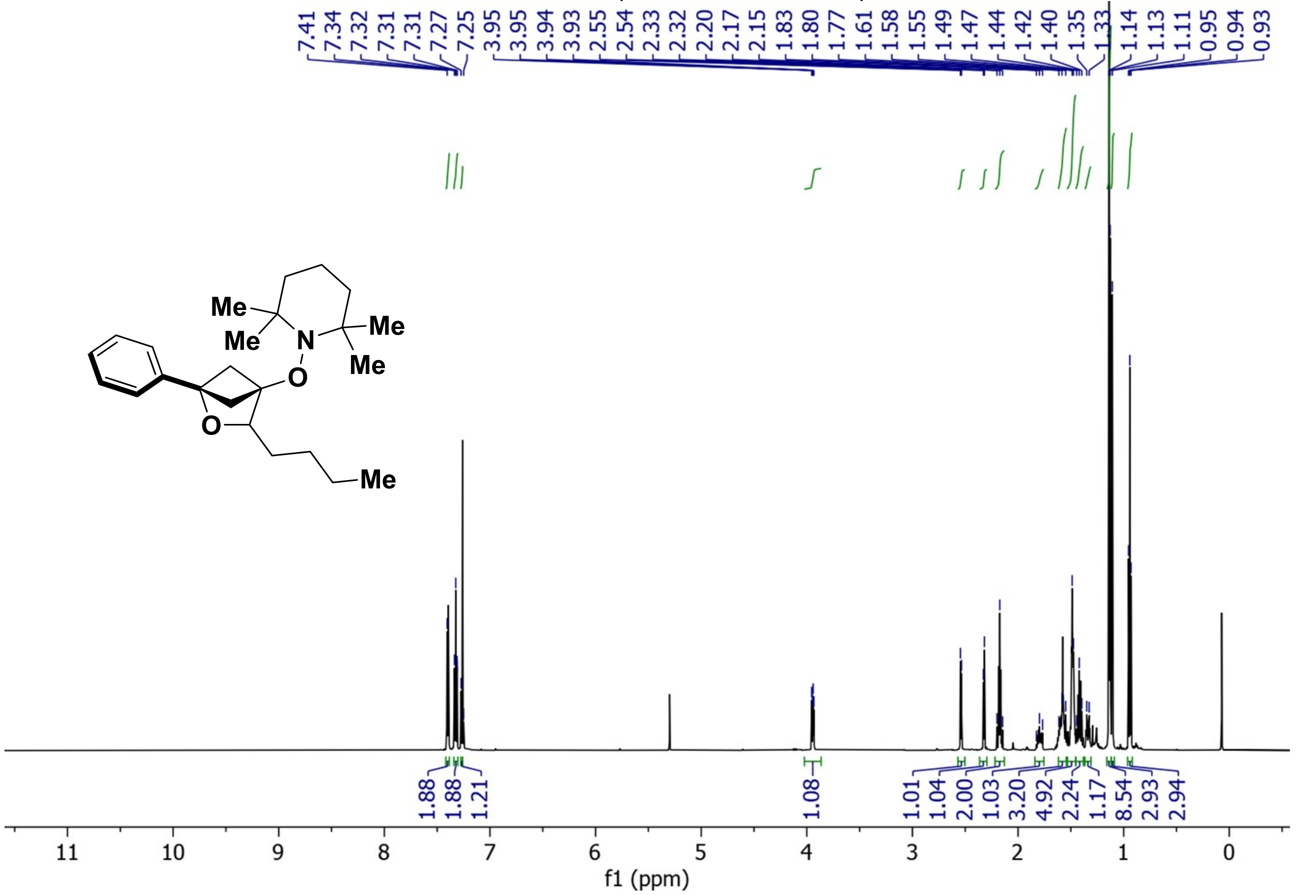

3va ^{13}C NMR (151 MHz, $CDCl_3$)

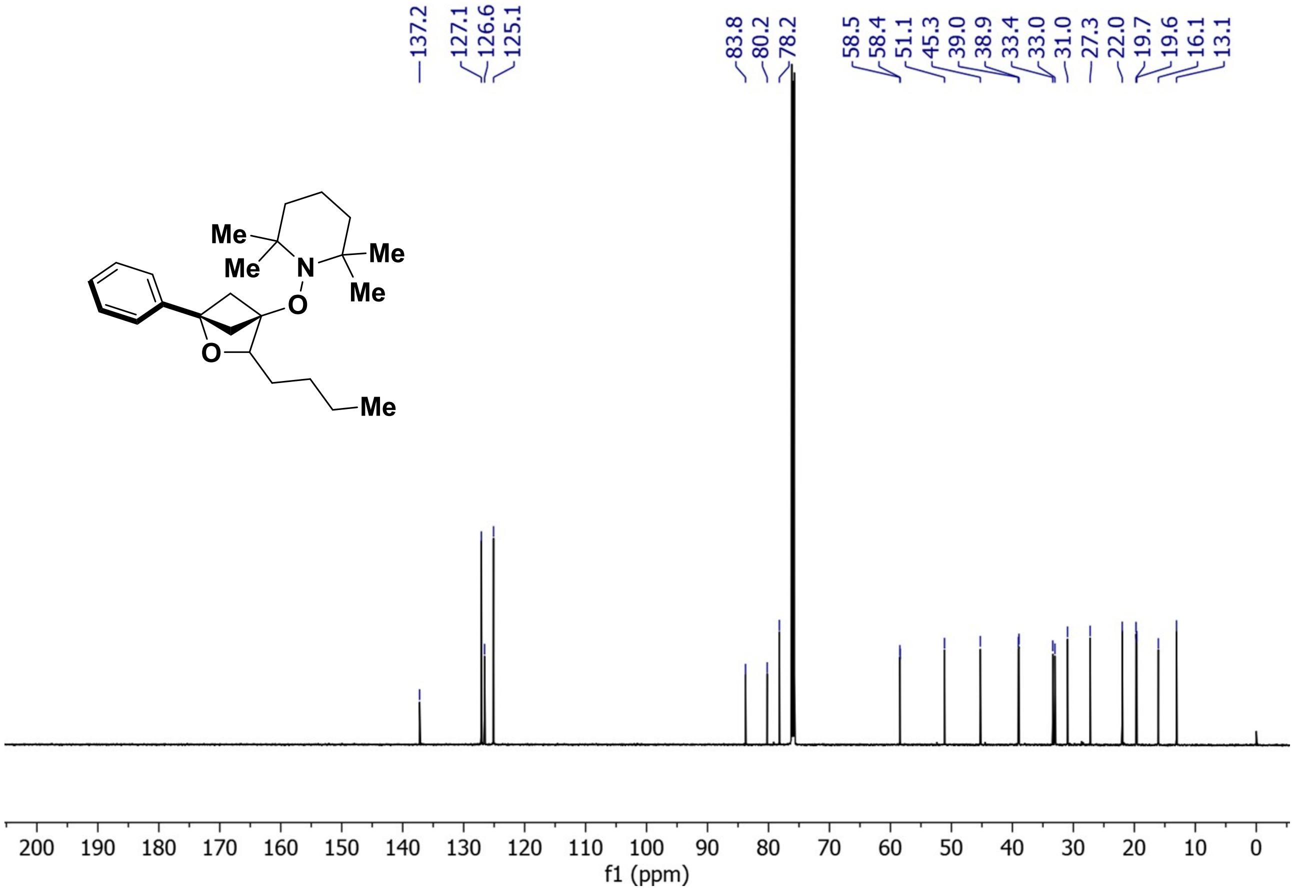

3wa ^{1}**H NMR** (600 MHz, $CDCl_3$)

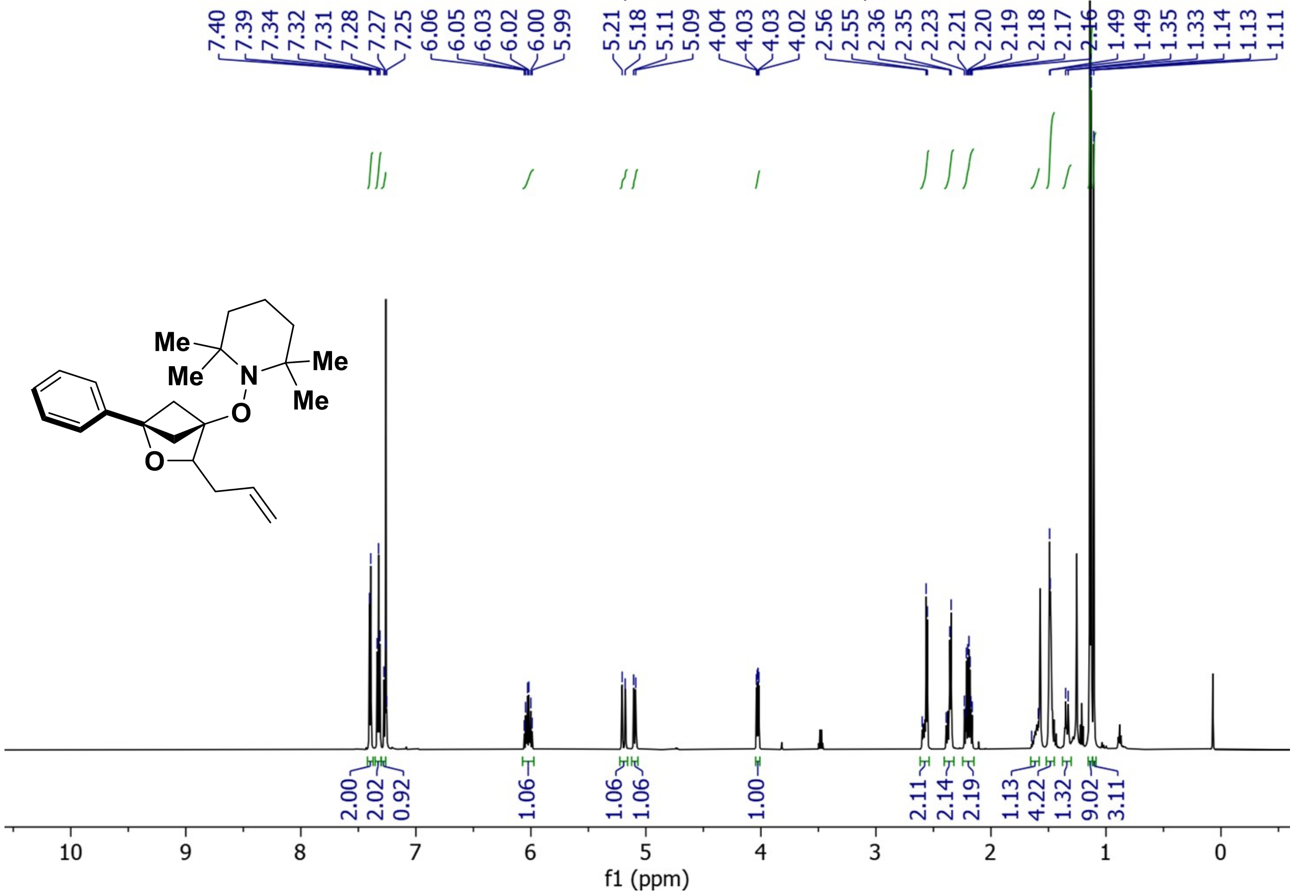

3wa ^{13}**C NMR** (151 MHz, $CDCl_3$)

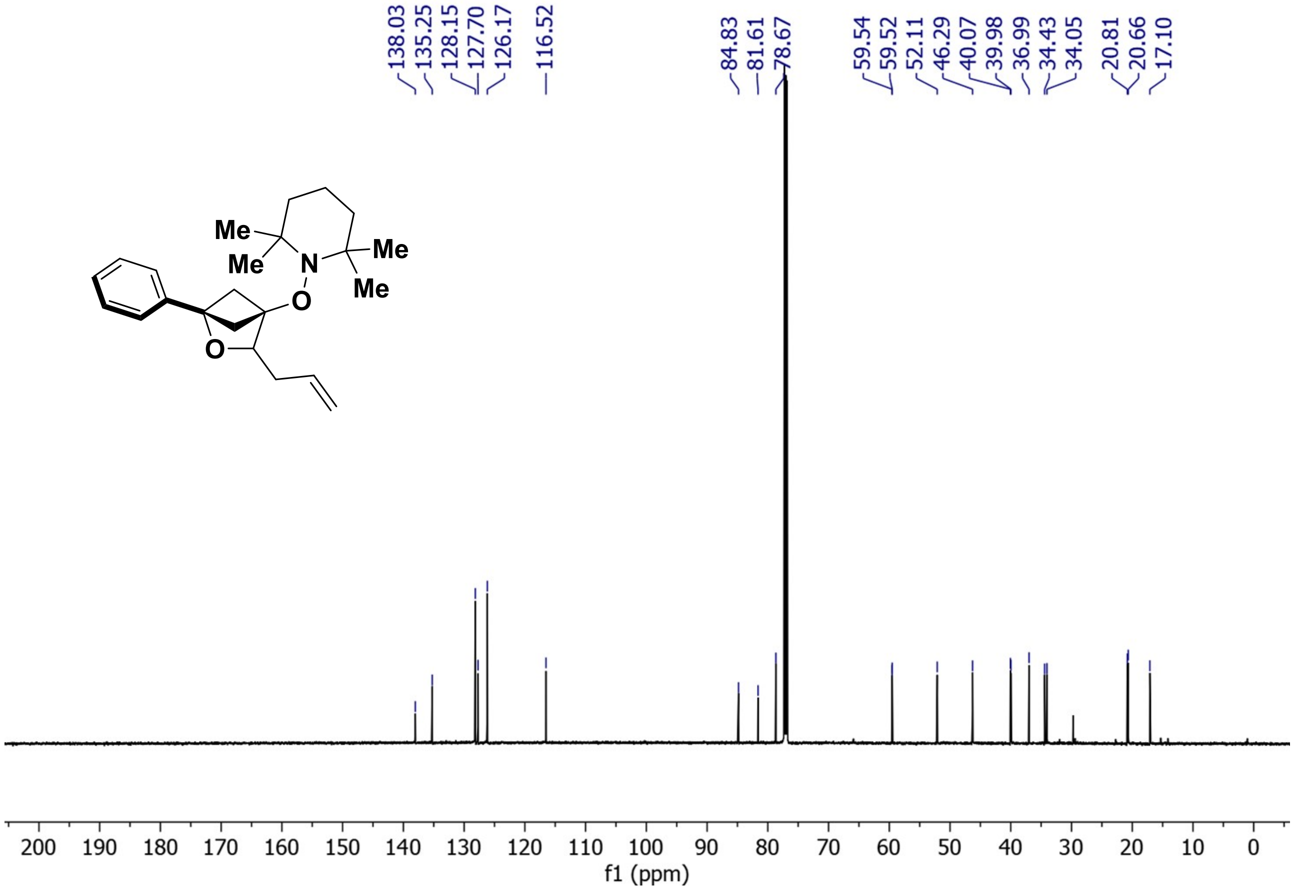

3ab ^{1}H NMR (600 MHz, $CDCl_3$)

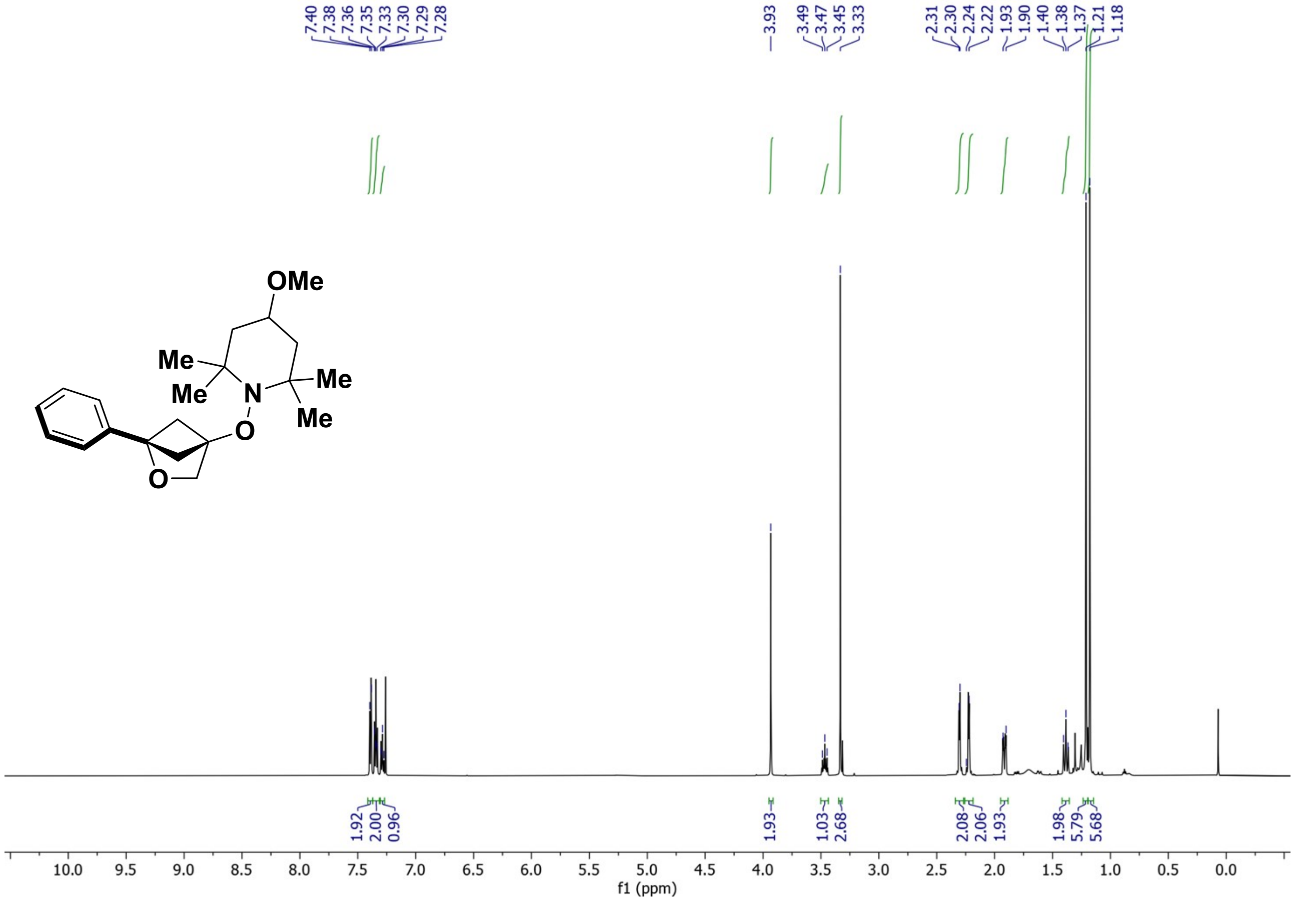

3ab ^{13}C NMR (151 MHz, $CDCl_3$)

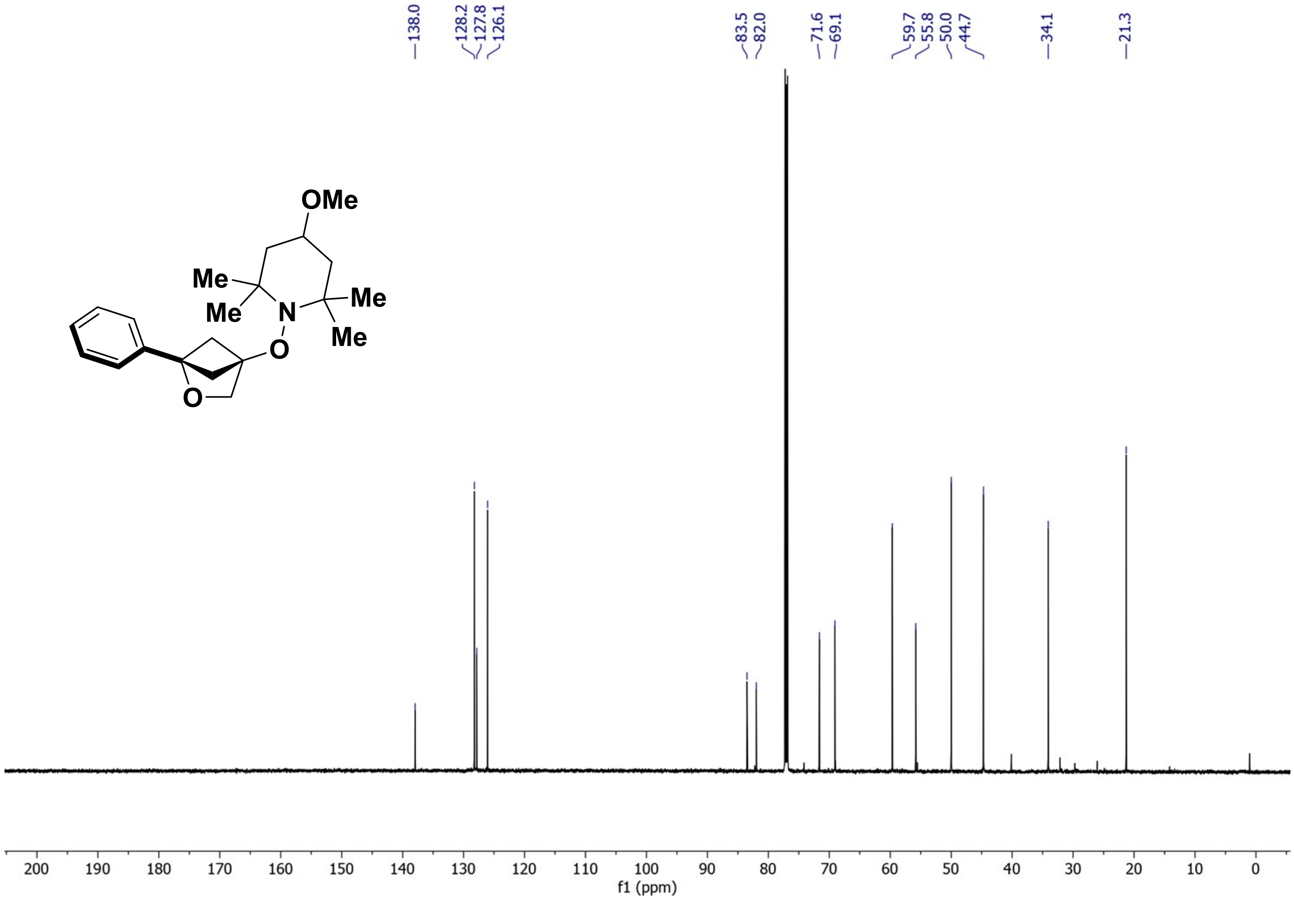

3ac ¹H NMR (600 MHz, $CDCl_3$)

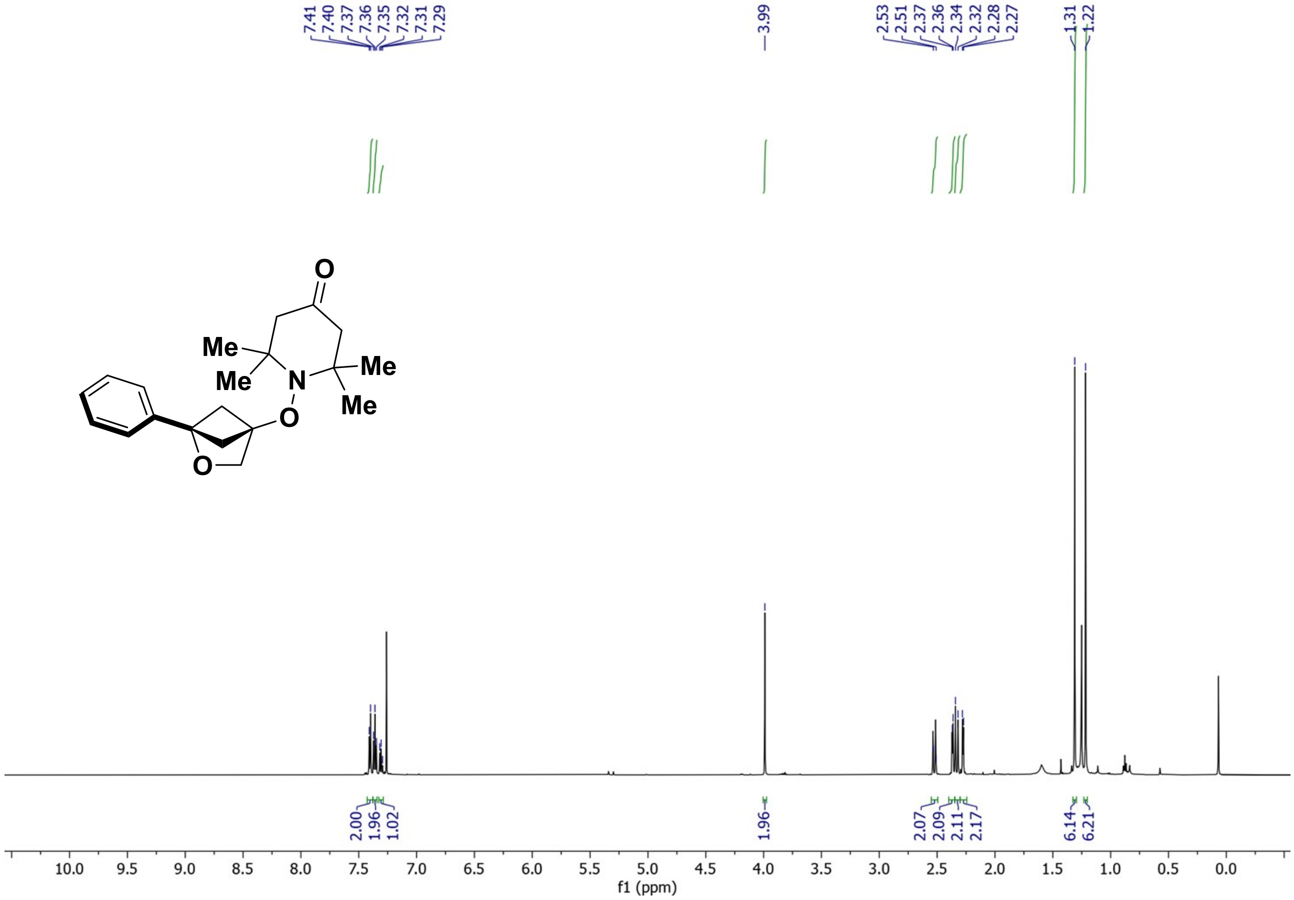

3ac ¹³C NMR (151 MHz, $CDCl_3$)

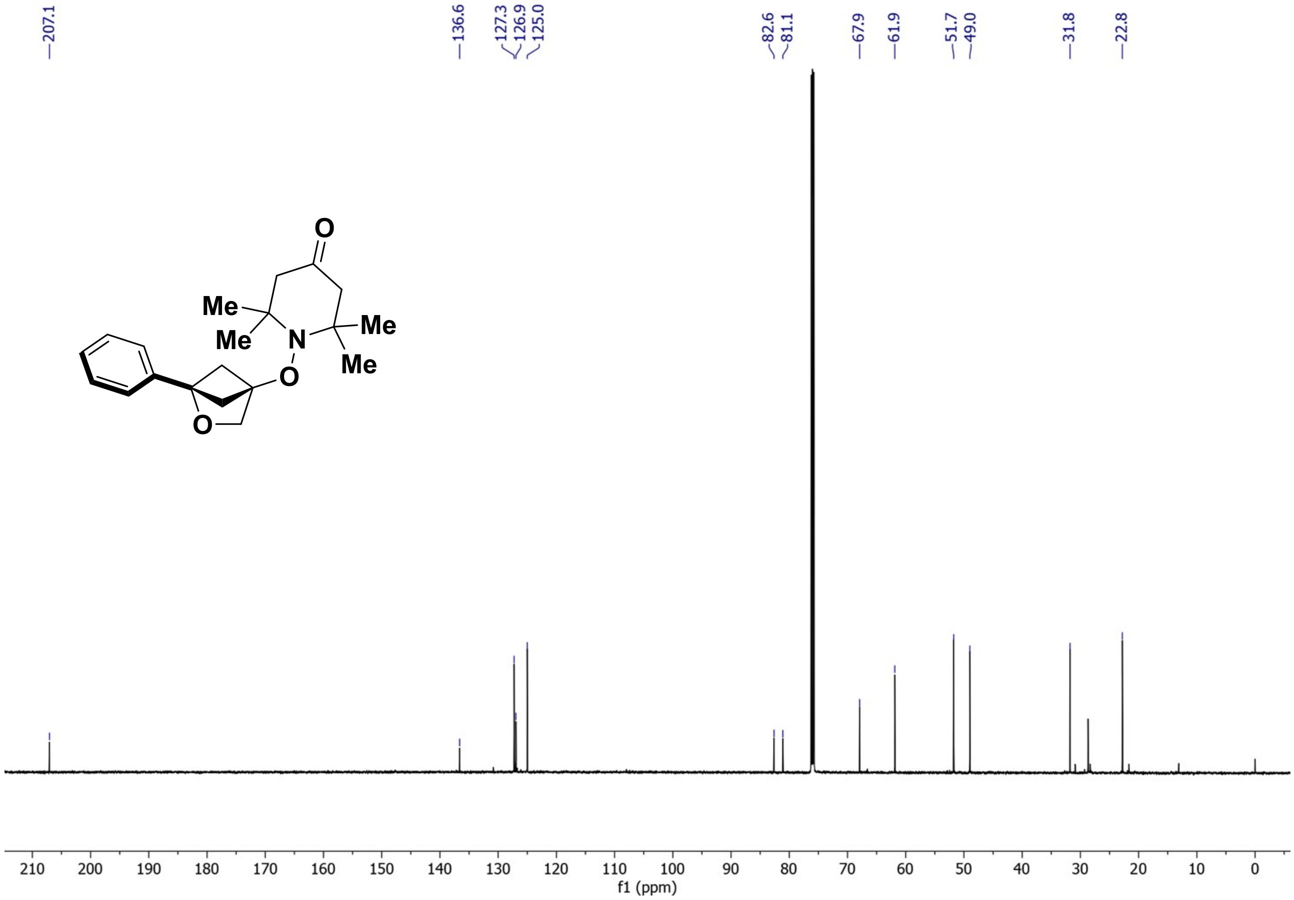

3ad ^{1}H NMR (600 MHz, $CDCl_3$)

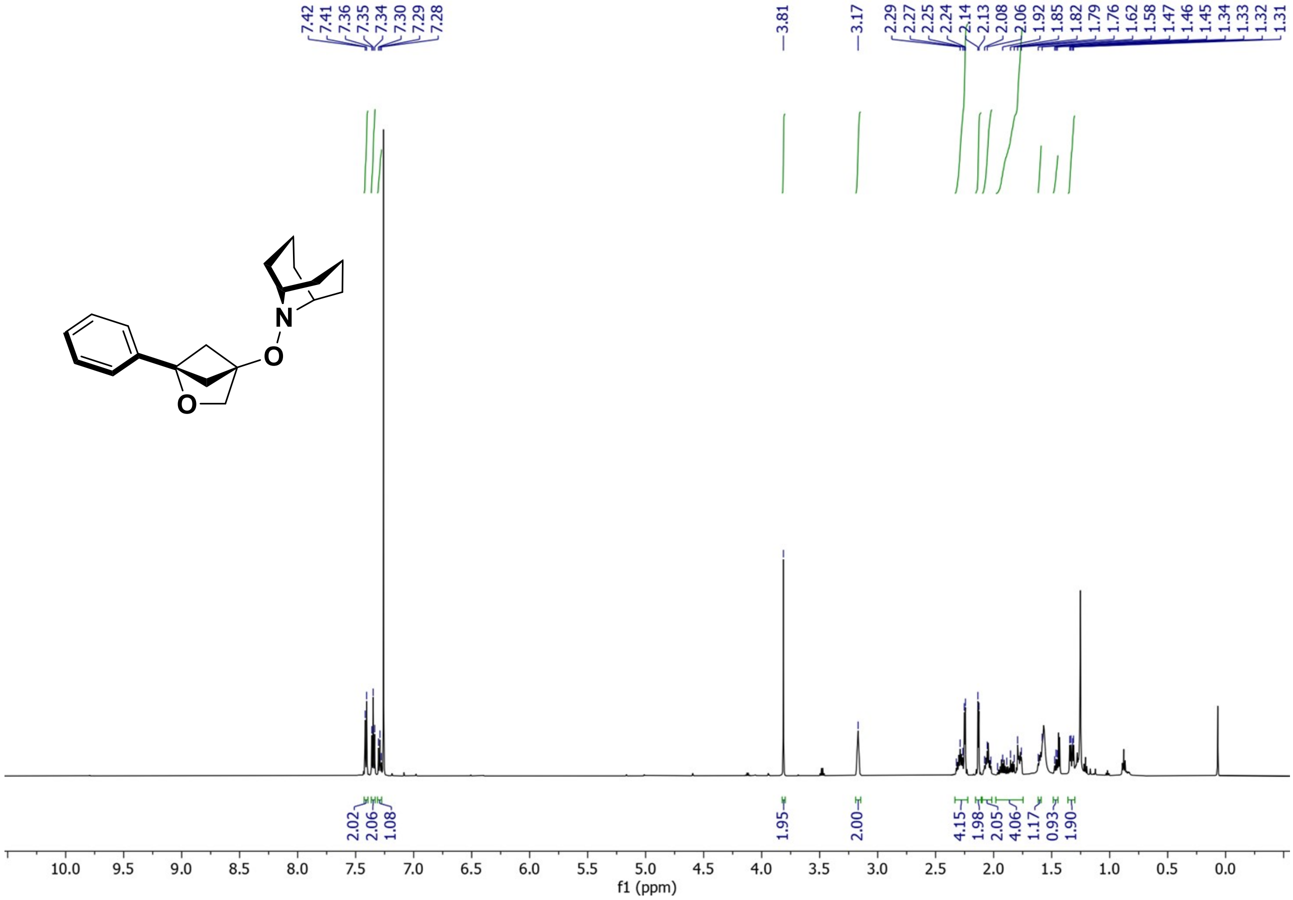

3ad ^{13}C NMR (151 MHz, $CDCl_3$)

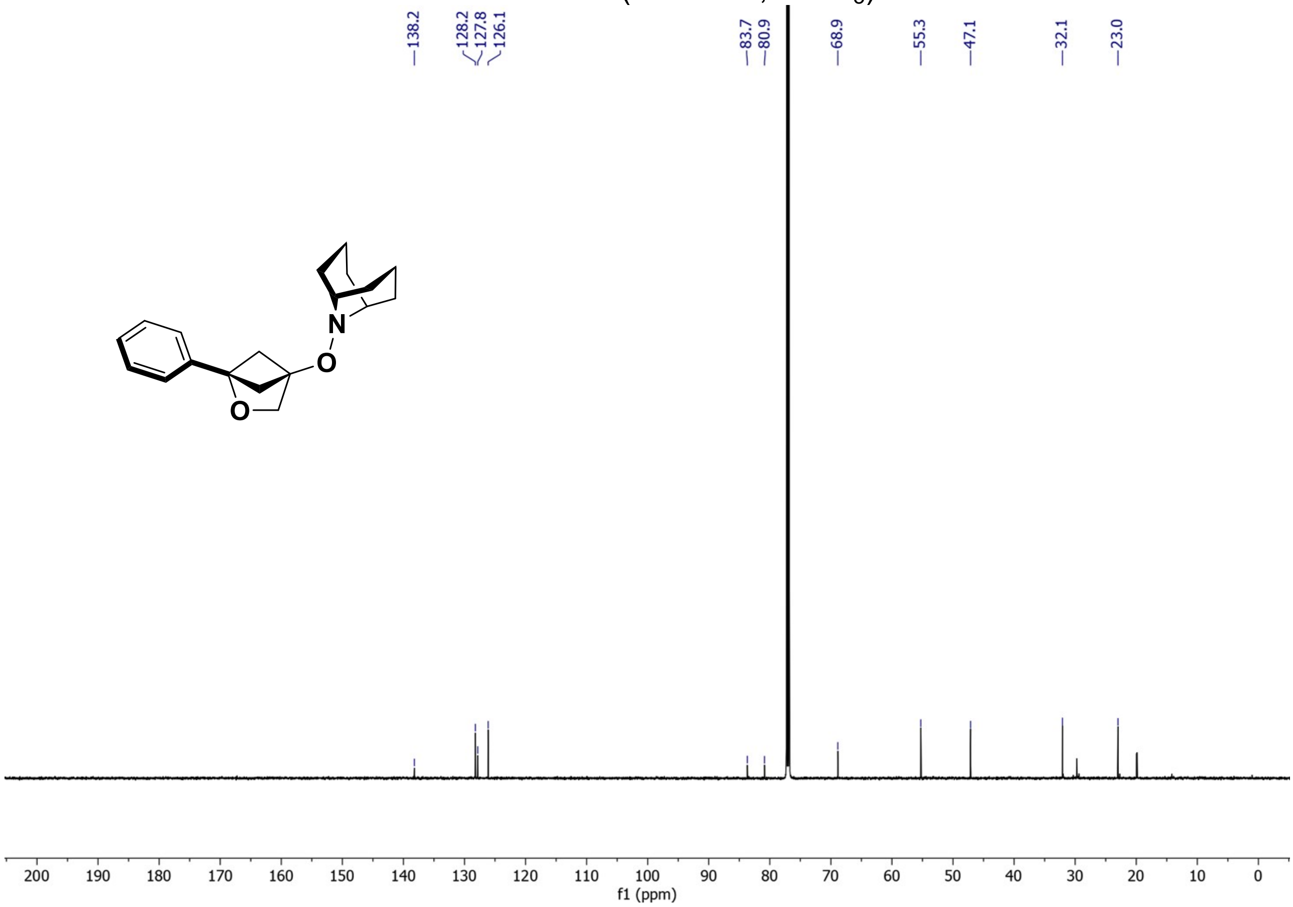

3ae ^{1}H NMR (600 MHz, $CDCl_3$)

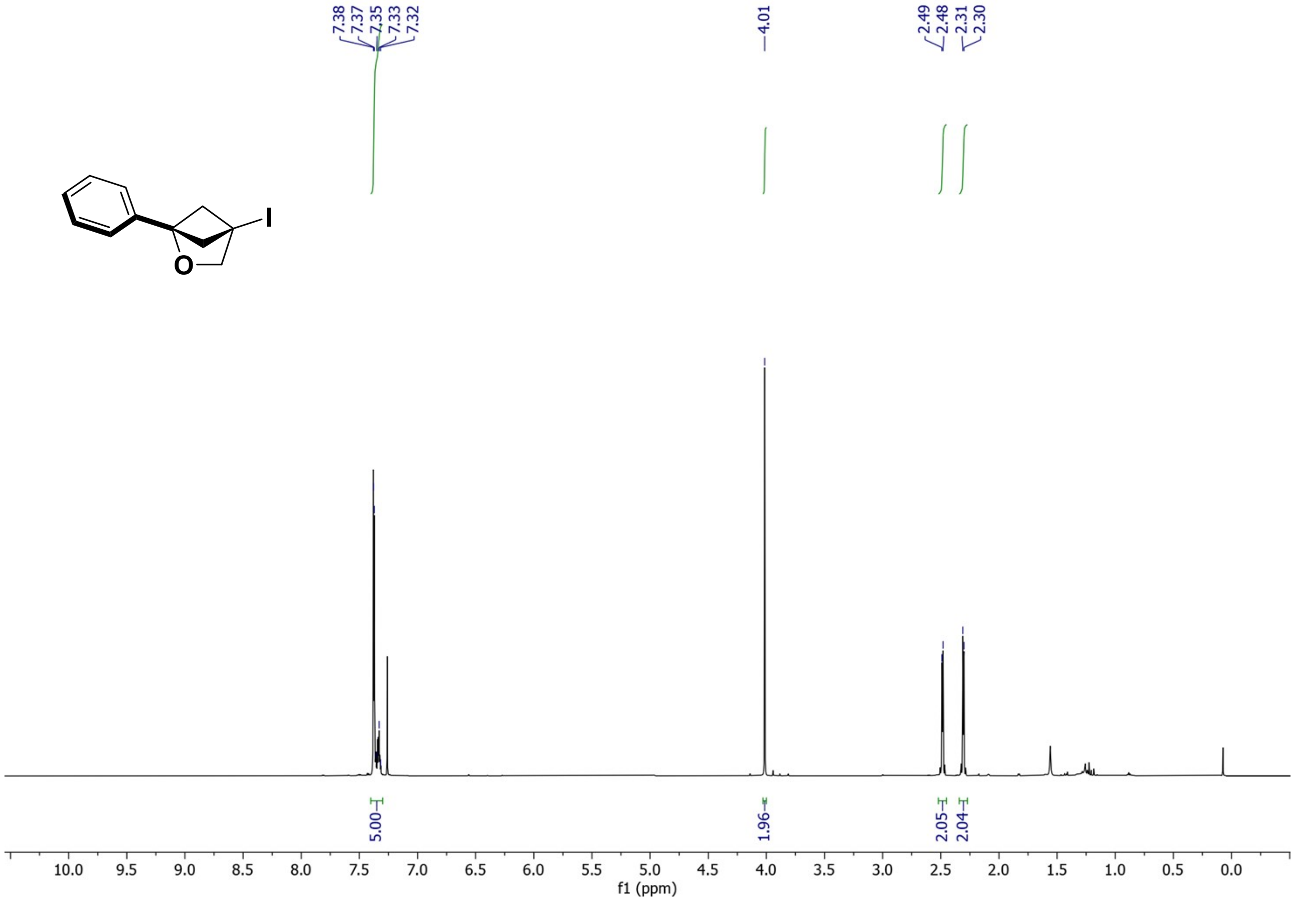

3ae ^{13}C NMR (151 MHz, $CDCl_3$)

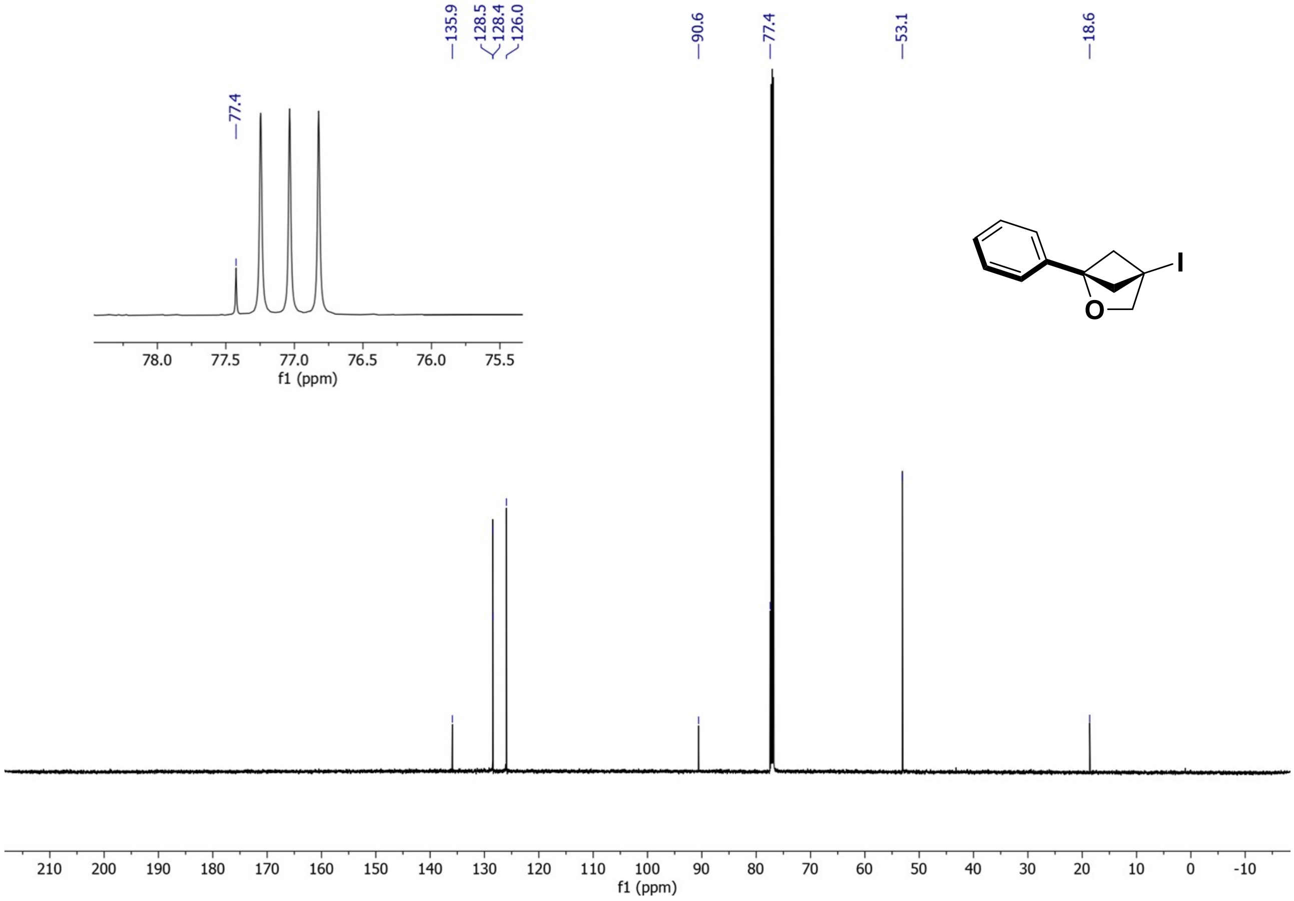

3ie ^{1}H NMR (600 MHz, $CDCl_3$)

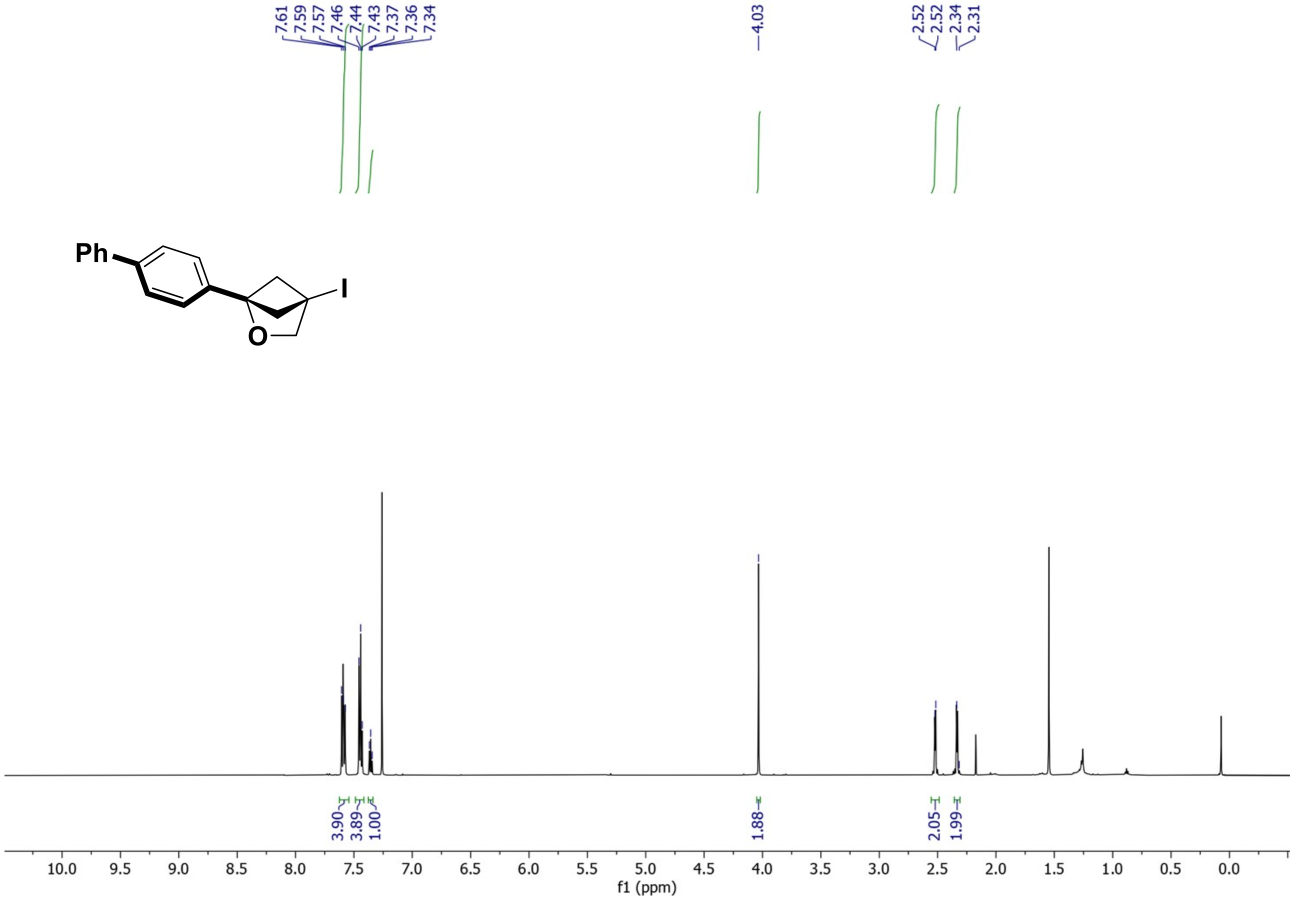

3ie ^{13}C NMR (151 MHz, $CDCl_3$)

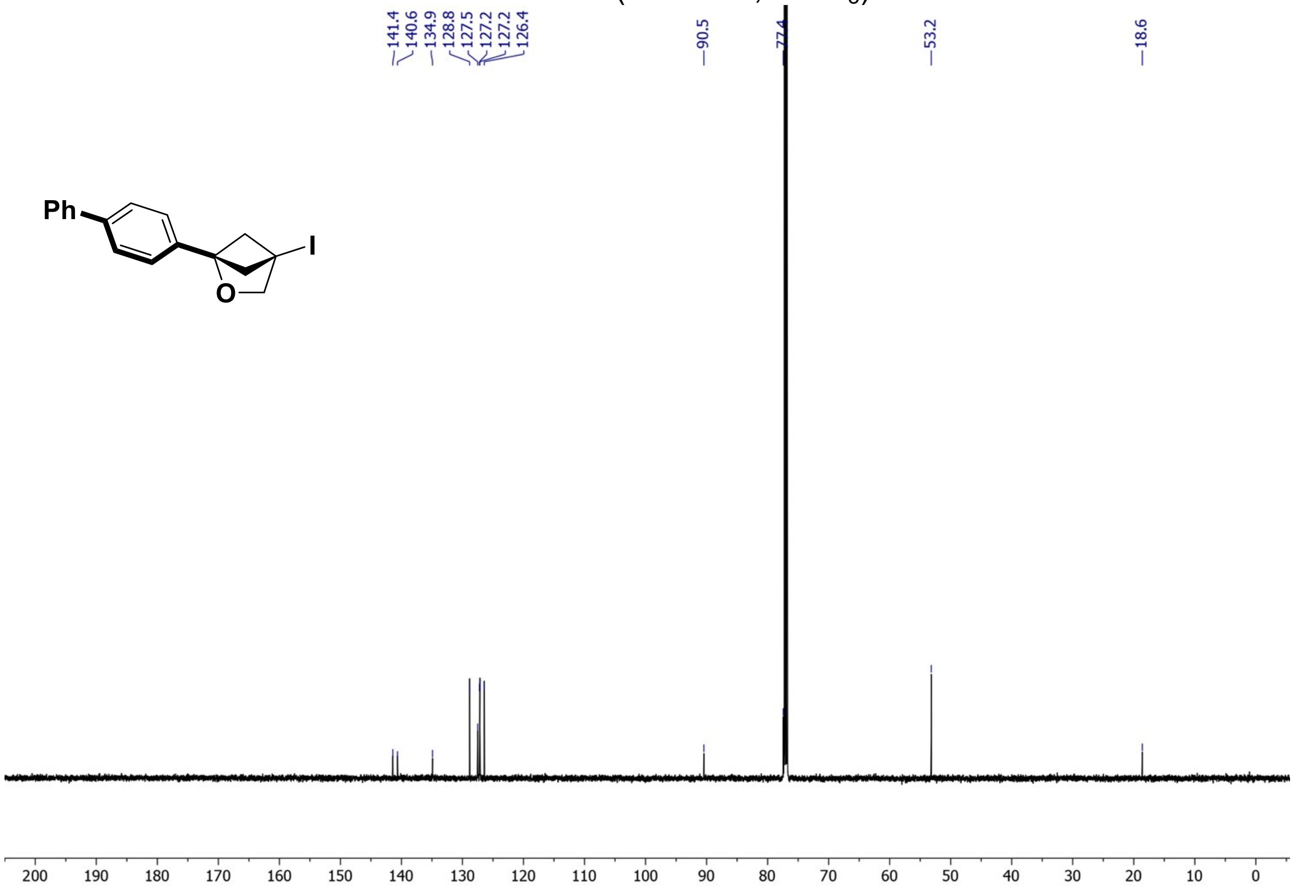

3ke ^{1}**H NMR** (600 MHz, $CDCl_3$)

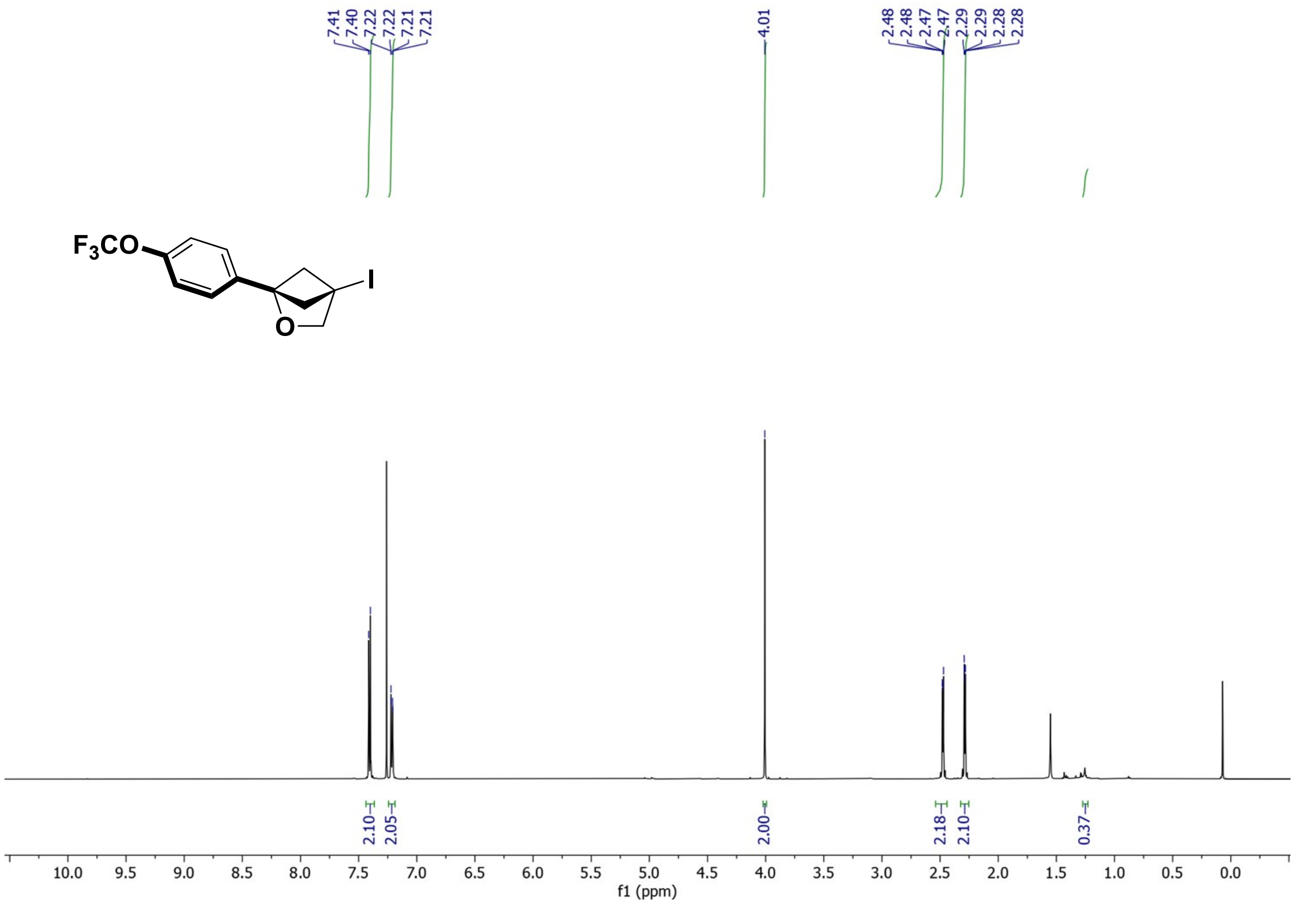

3ke ^{13}**C NMR** (151 MHz, $CDCl_3$)

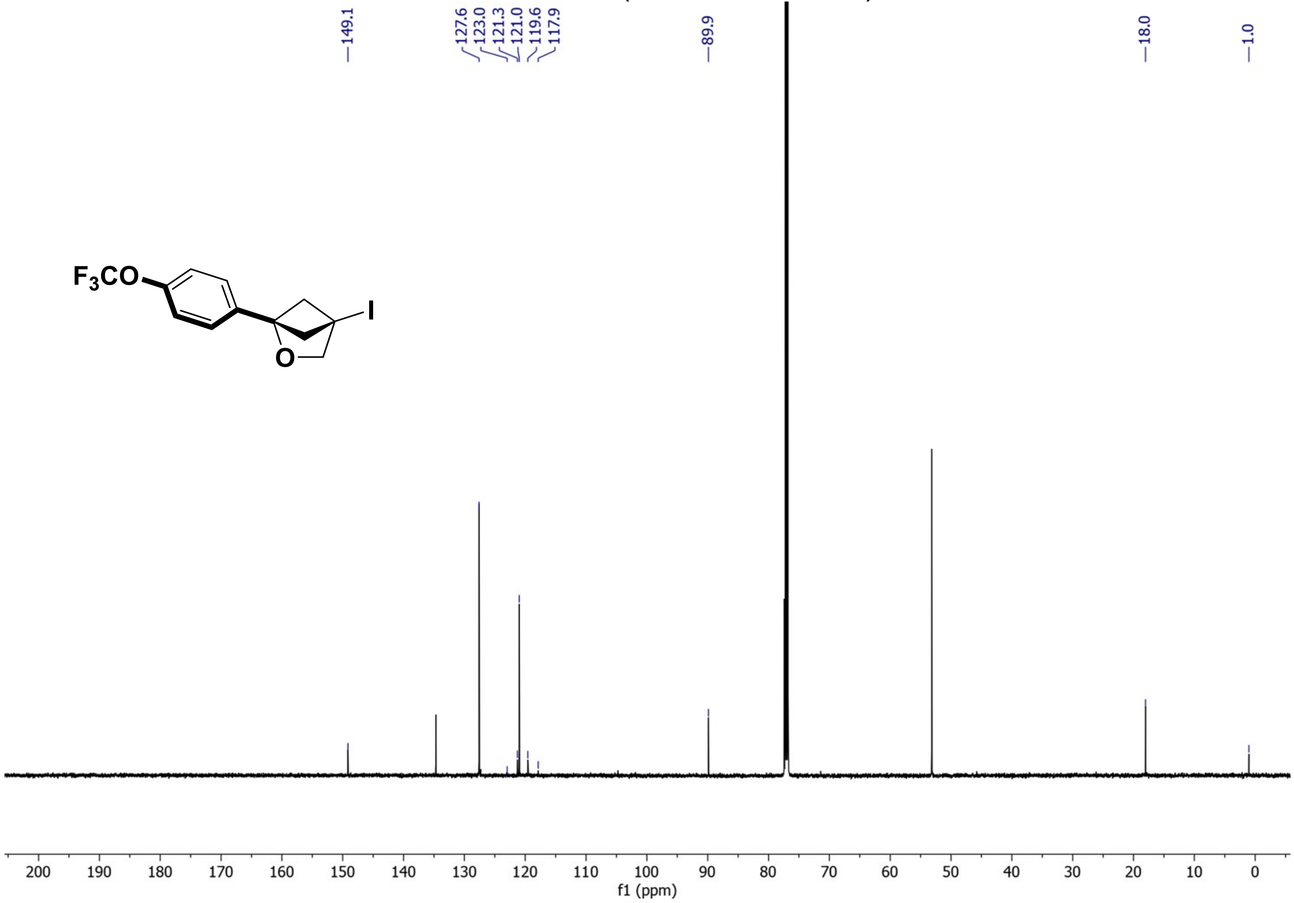

3ke ^{19}F NMR (565 MHz, $CDCl_3$)

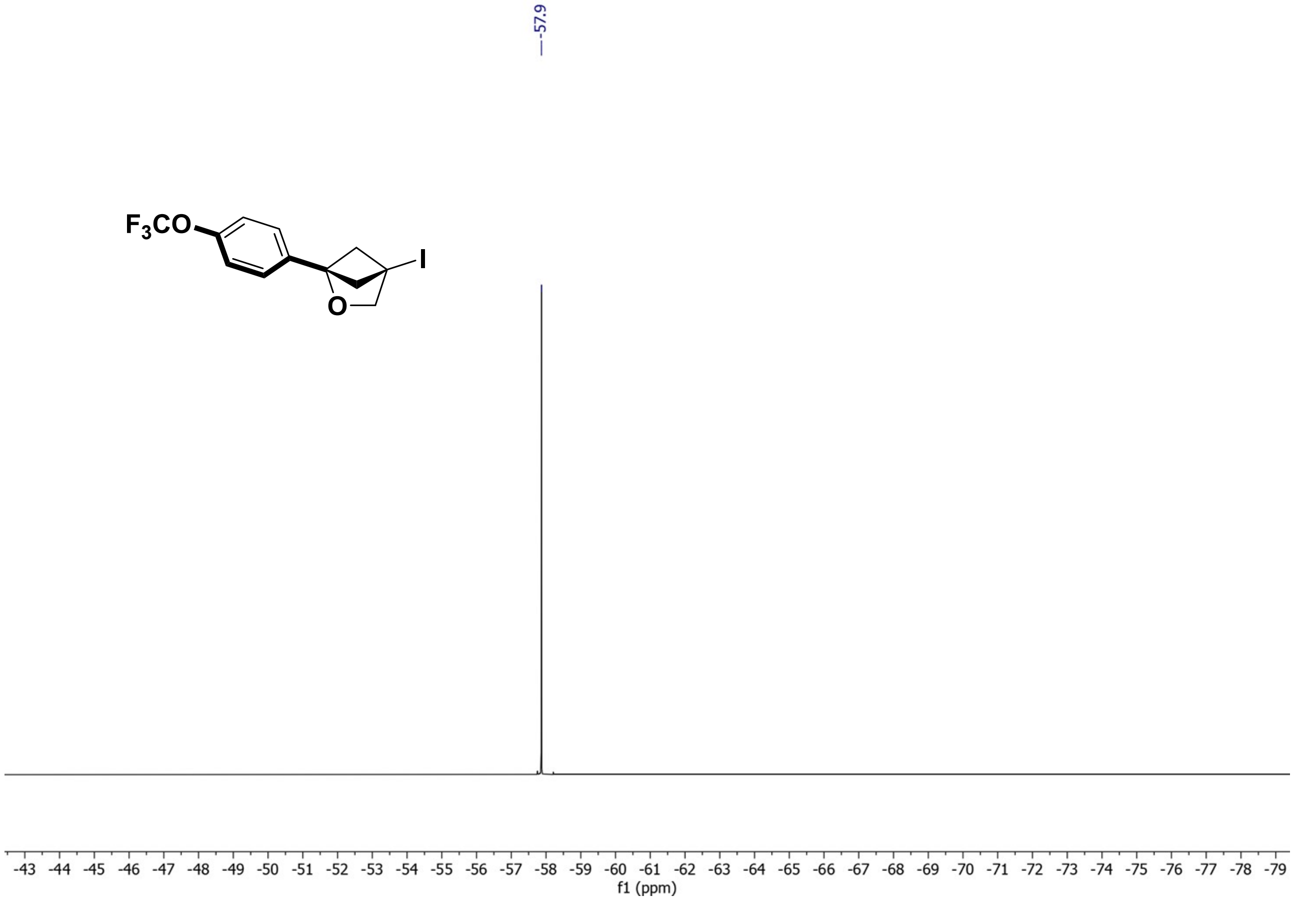

3ne ^{1}H NMR (600 MHz, $CDCl_3$)

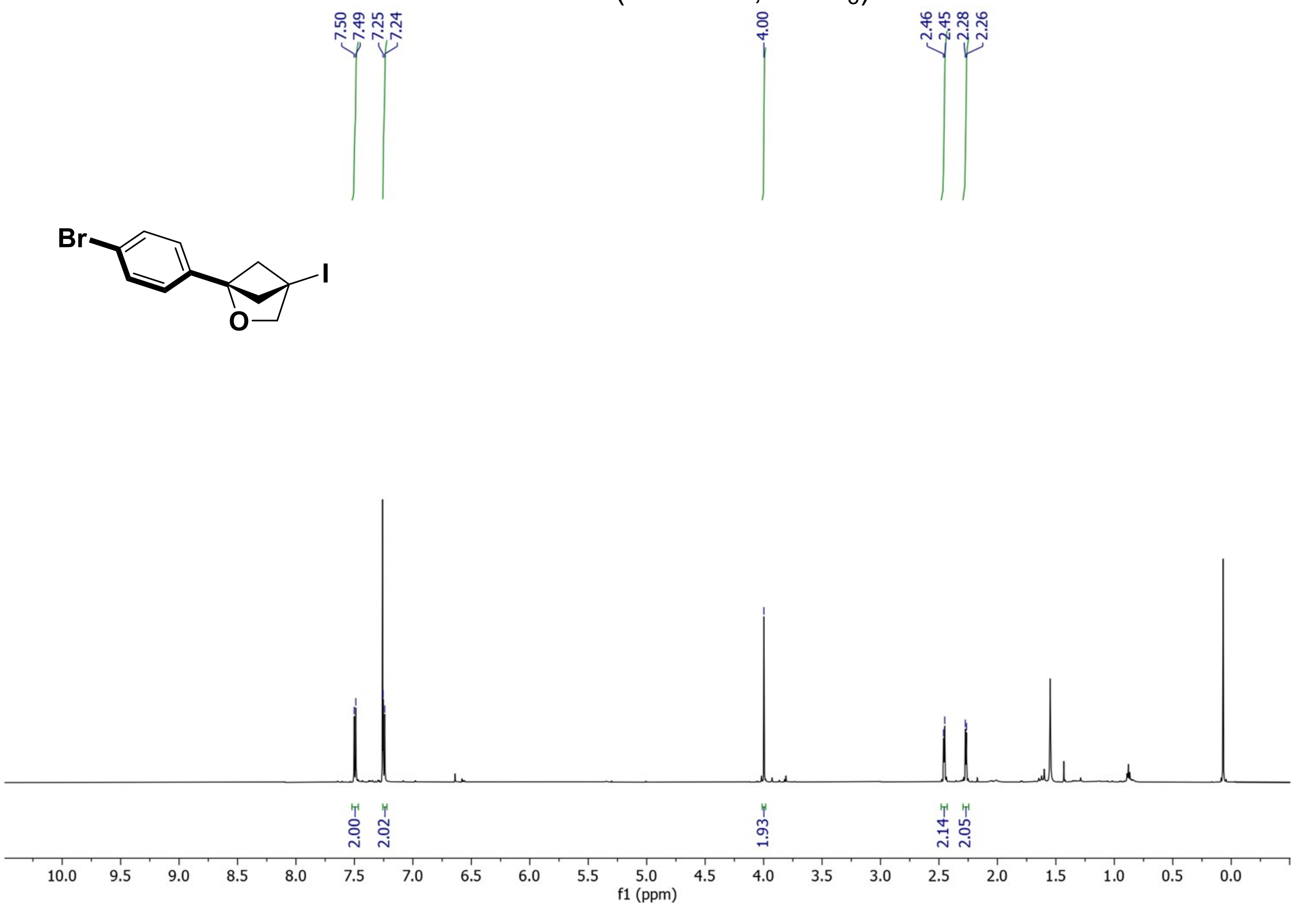

3ne ^{13}C NMR (151 MHz, $CDCl_3$)

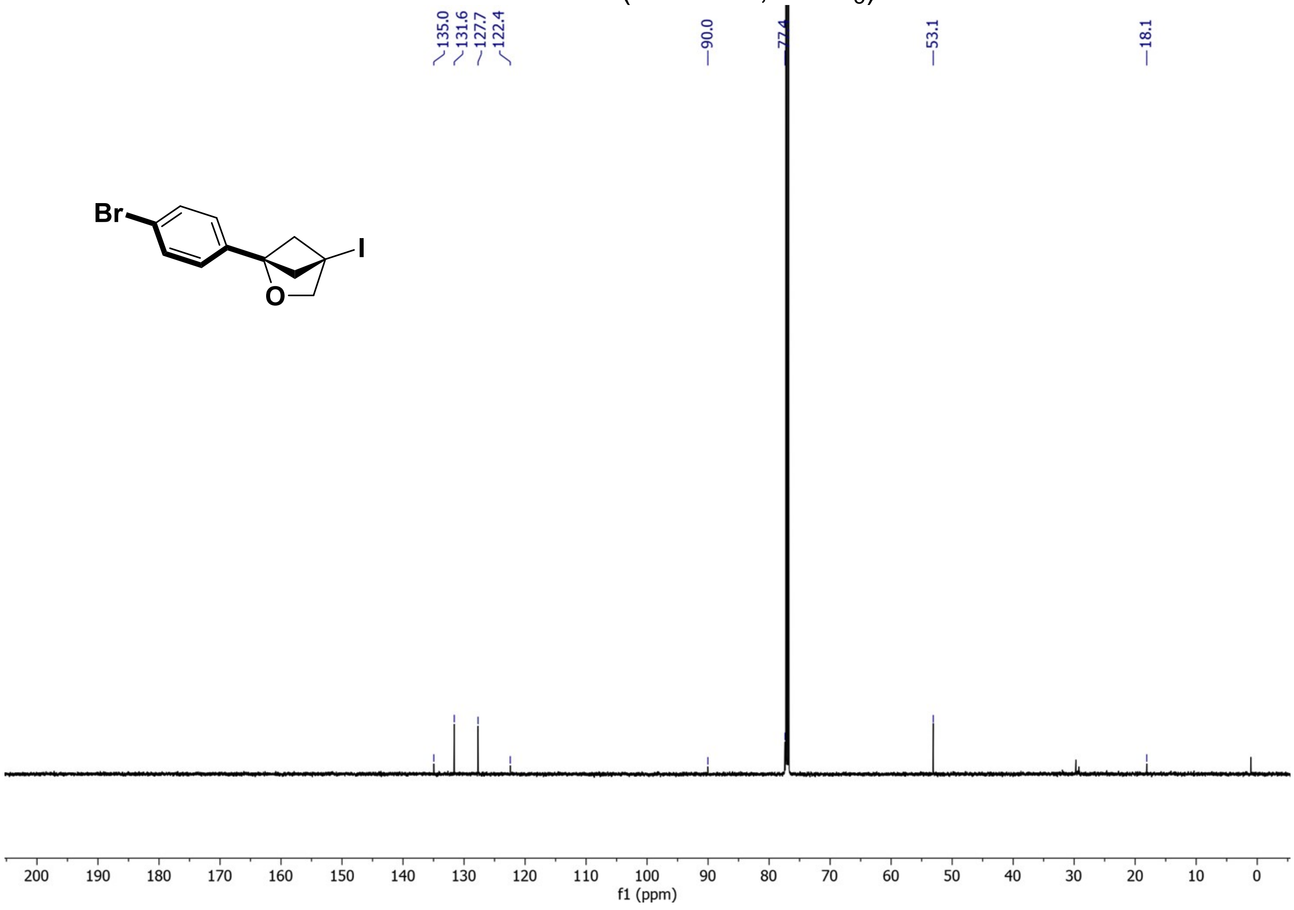

3oe ^{1}H NMR (600 MHz, $CDCl_3$)

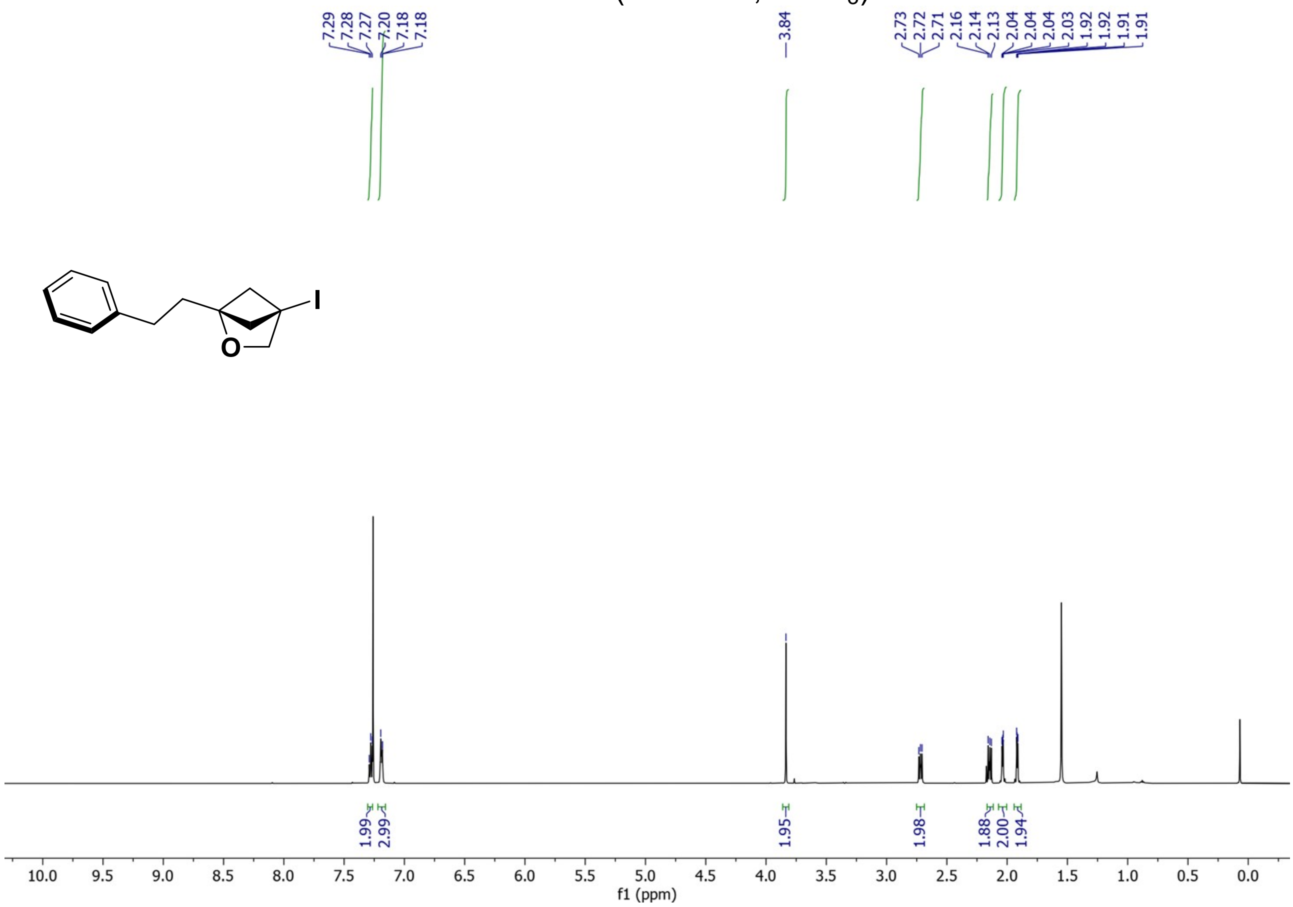

3oe ^{13}C NMR (151 MHz, $CDCl_3$)

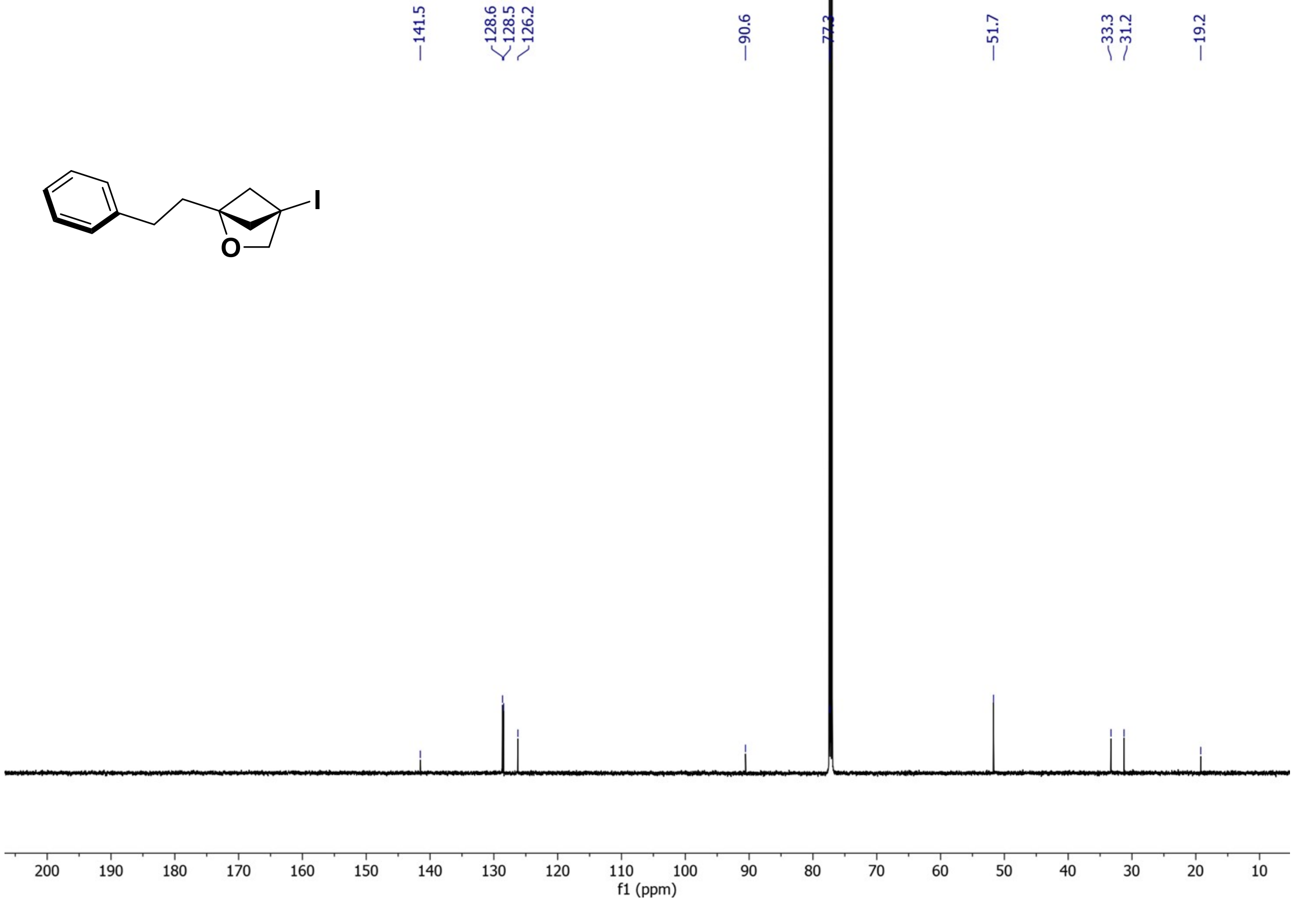

3xe ^{1}H NMR (600 MHz, $CDCl_3$)

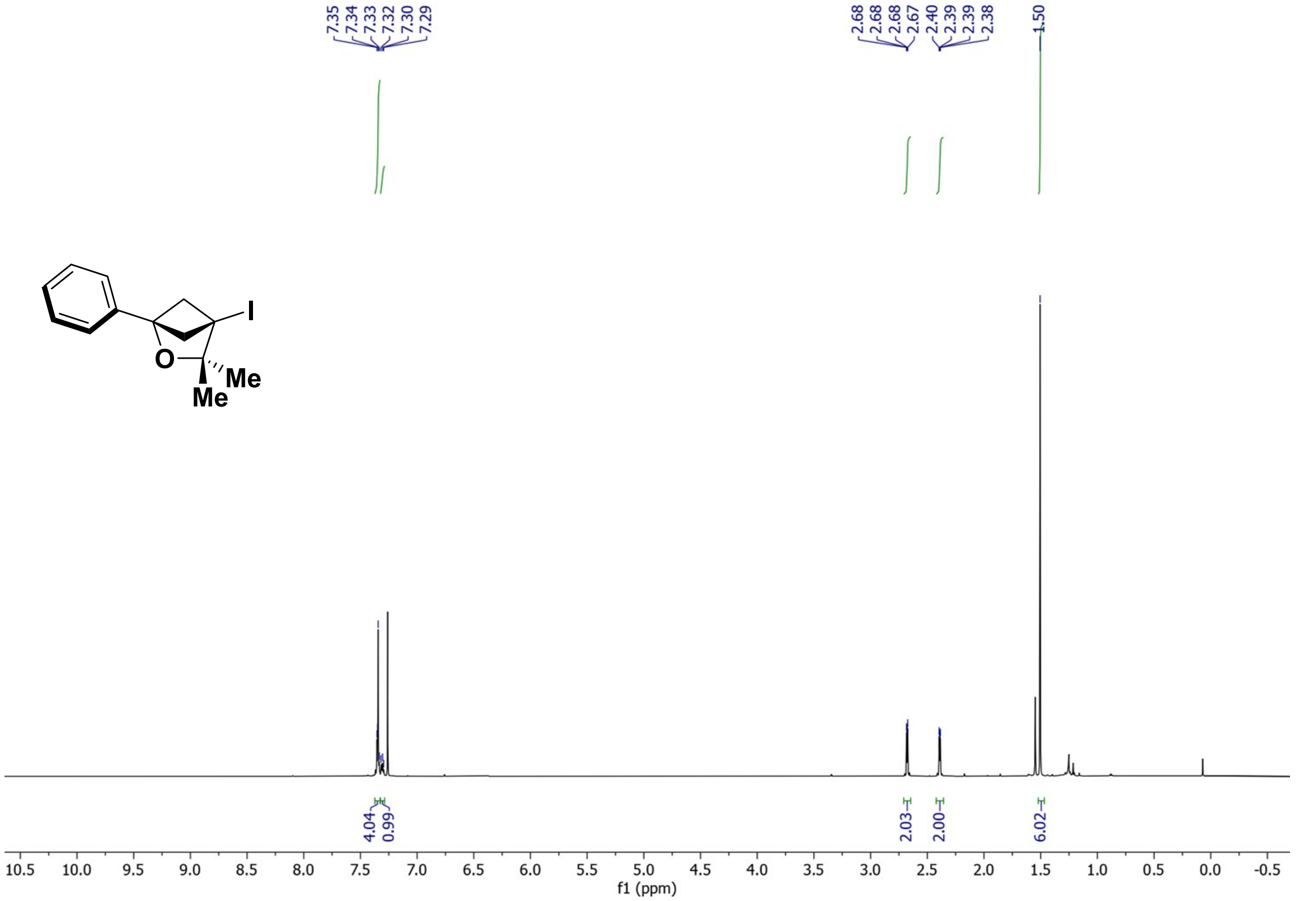

3xe ^{13}C NMR (151 MHz, $CDCl_3$)

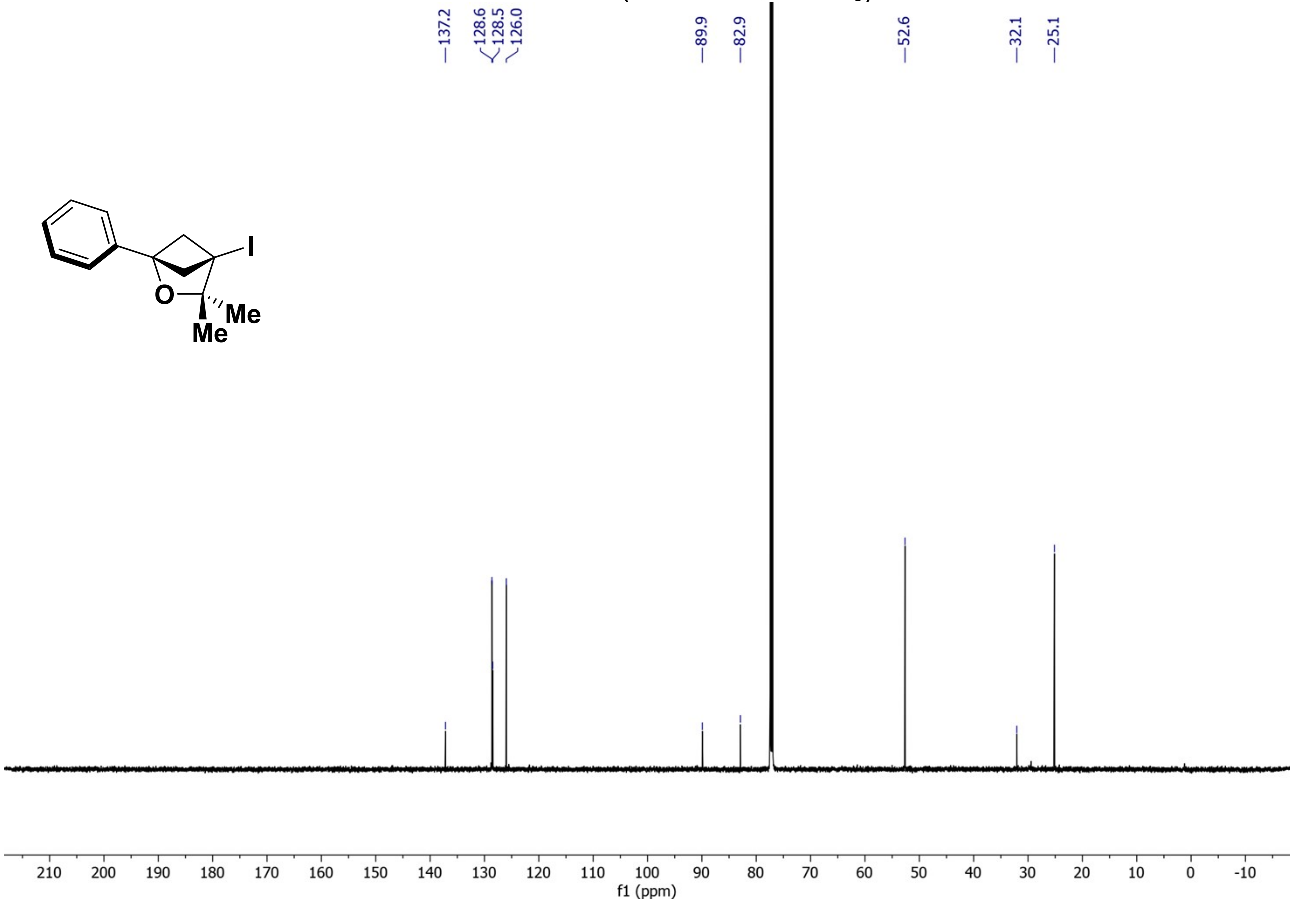

3ye ^{1}H NMR (600 MHz, $CDCl_3$)

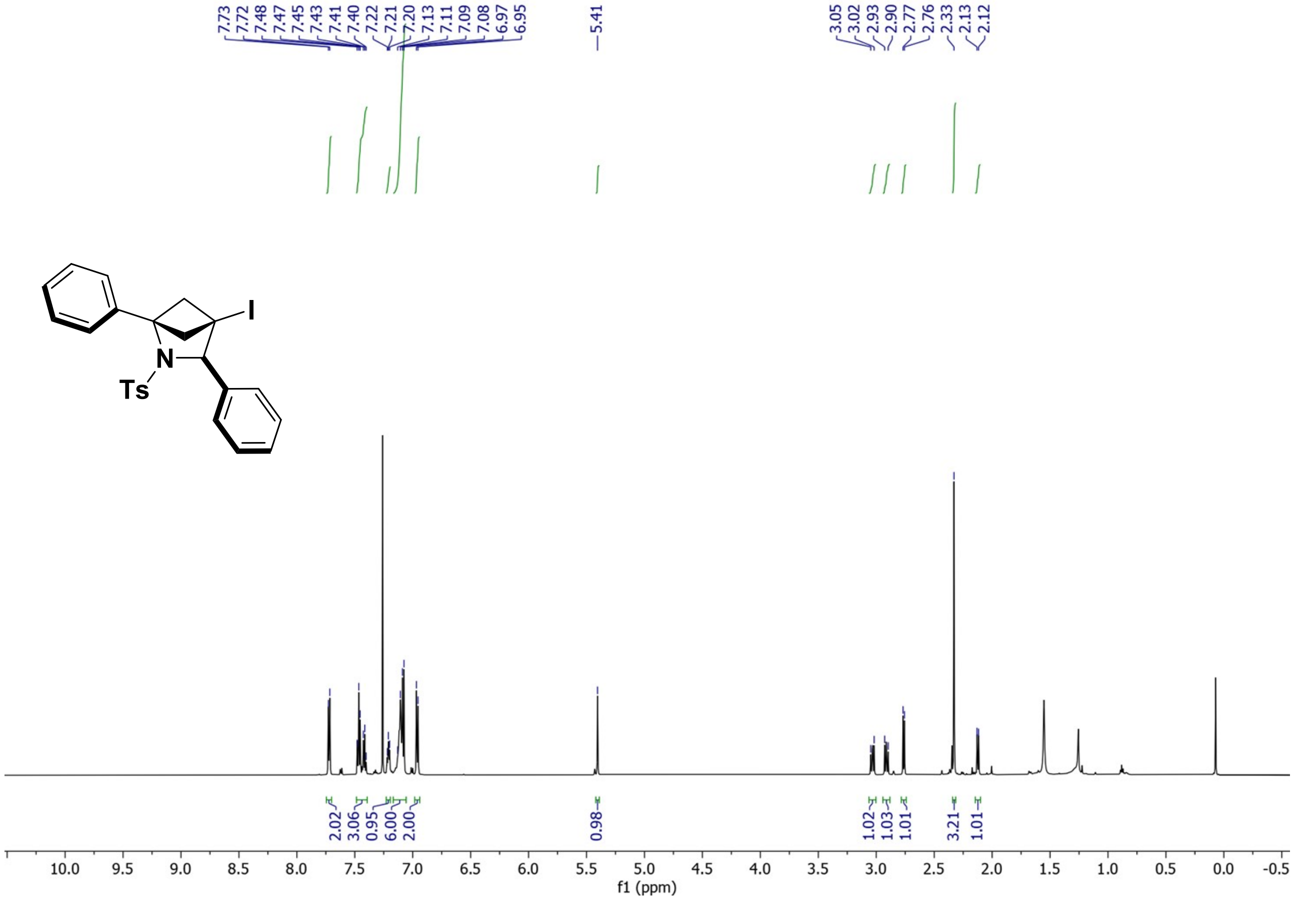

3ye ^{13}C NMR (151 MHz, $CDCl_3$)

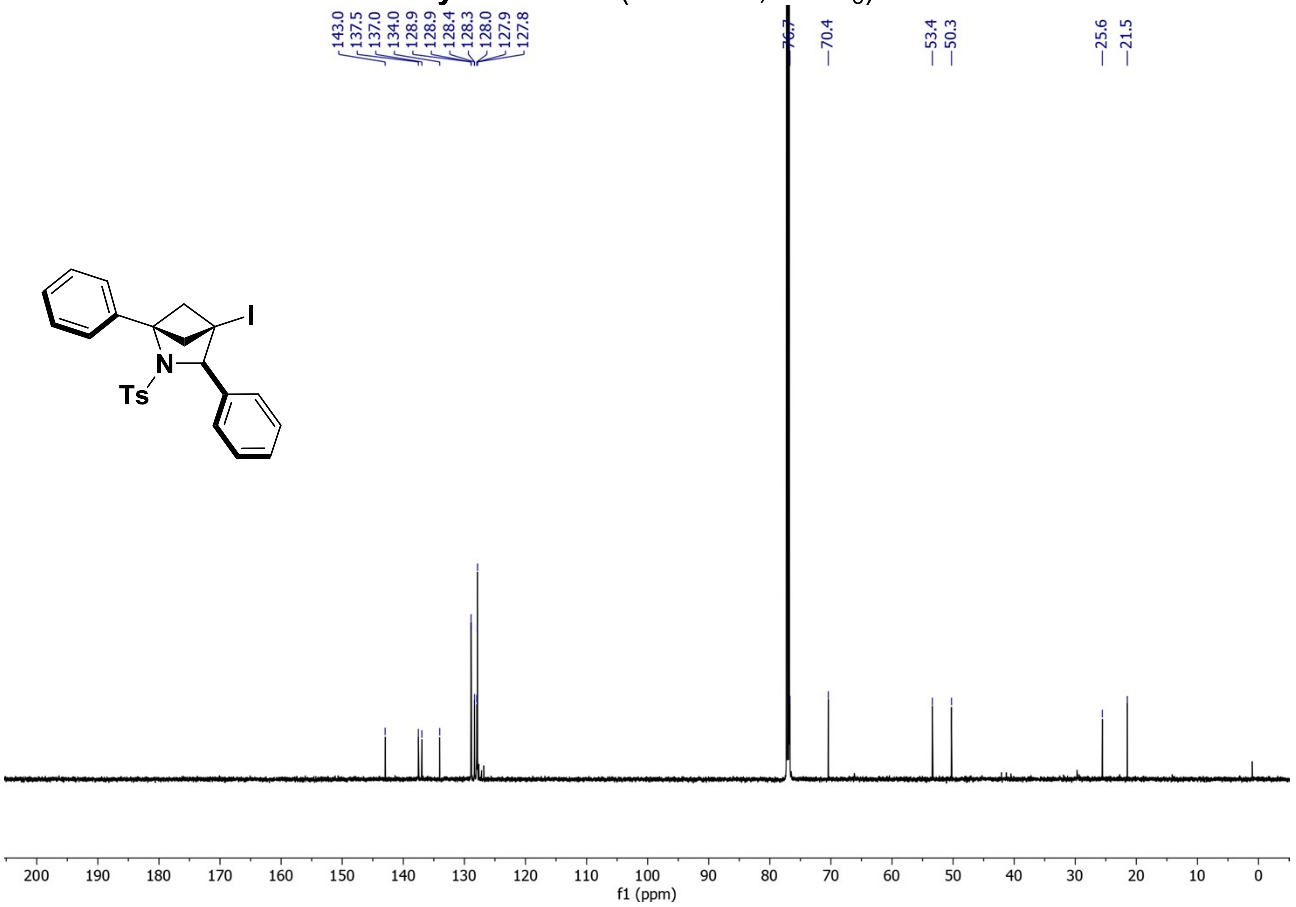

3af ^{1}H NMR (600 MHz, $CDCl_3$)

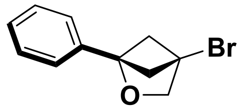

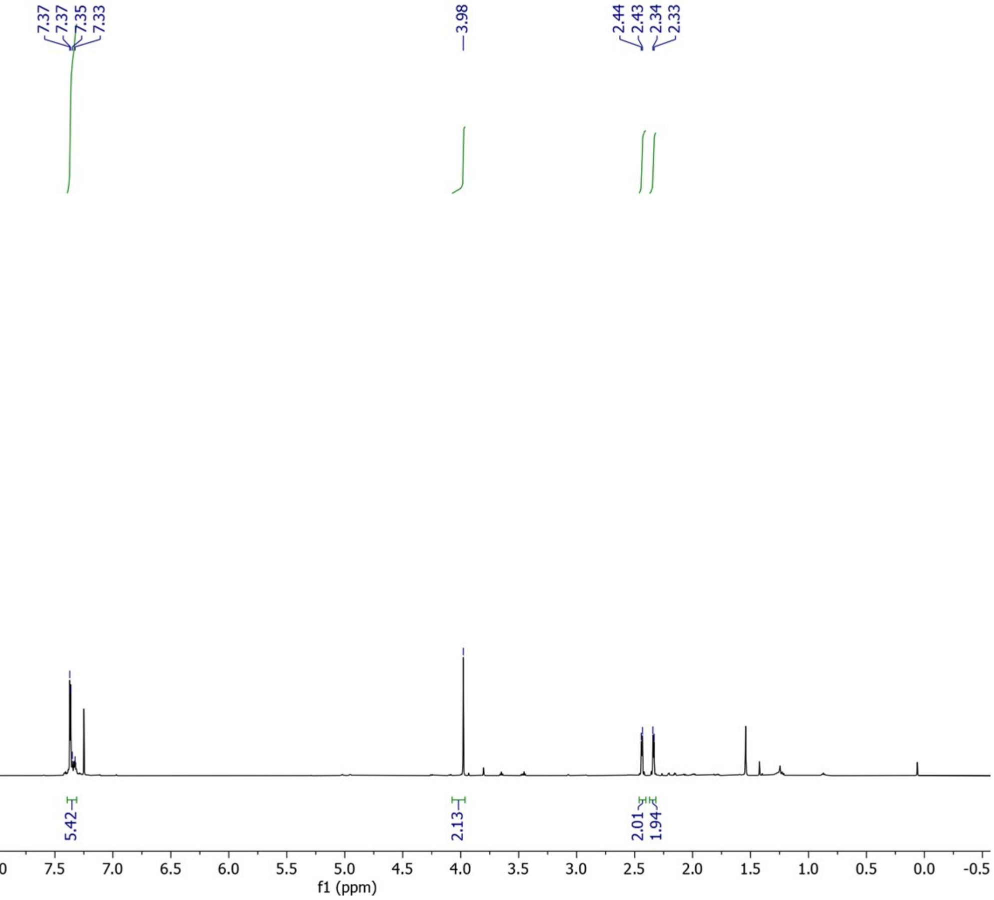

3af ^{13}C NMR (151 MHz, $CDCl_3$)

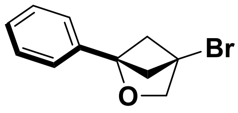

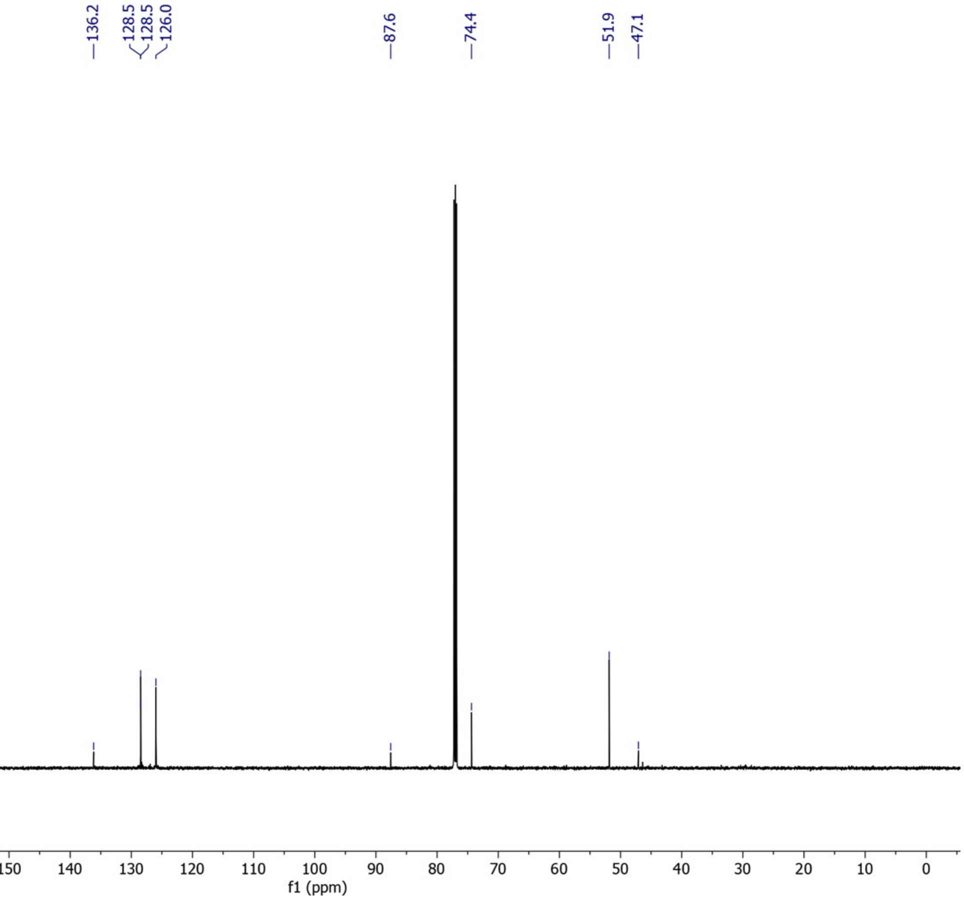

3kf ^{1}H NMR (600 MHz, $CDCl_3$)

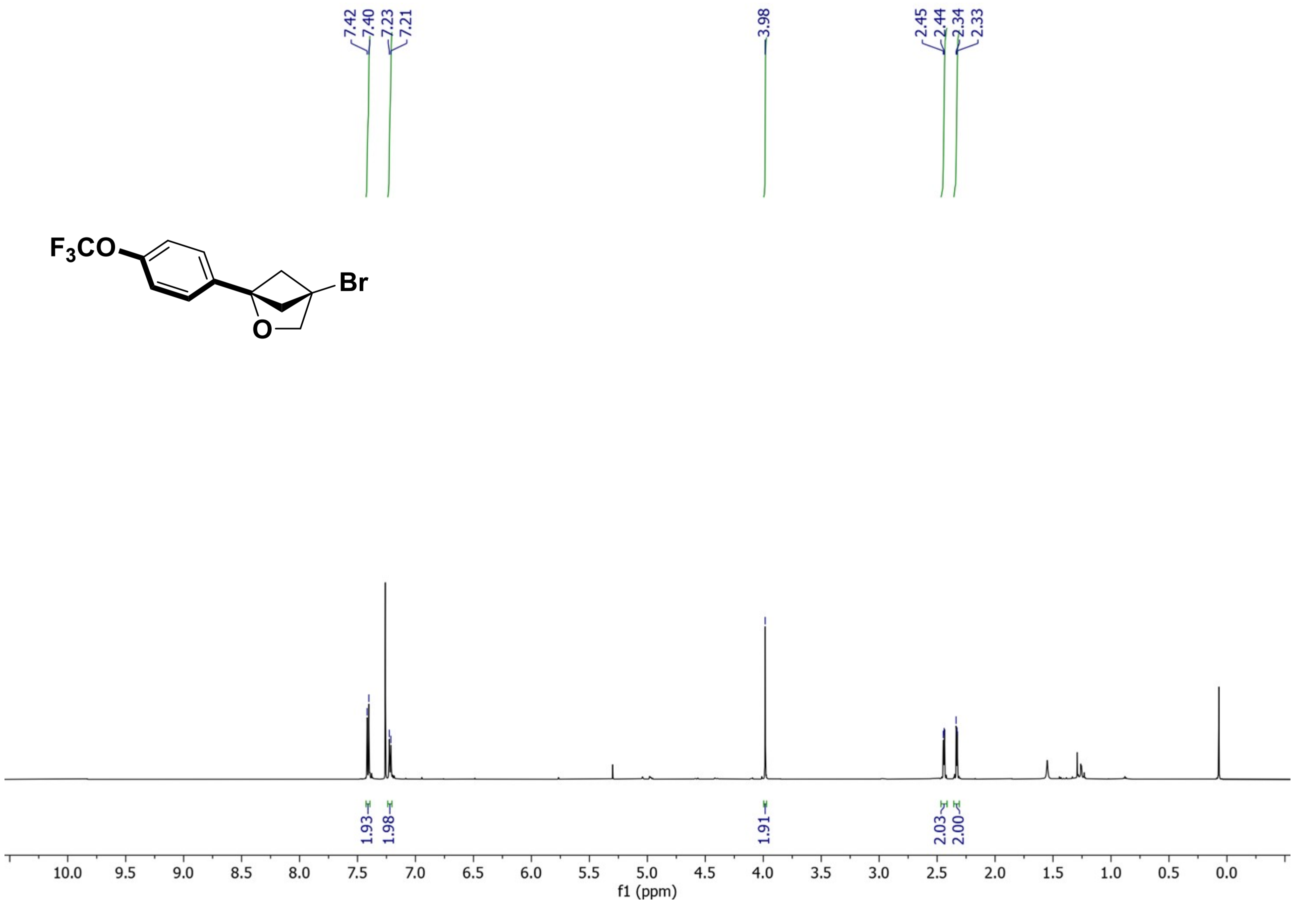

3kf ^{13}C NMR (151 MHz, $CDCl_3$)

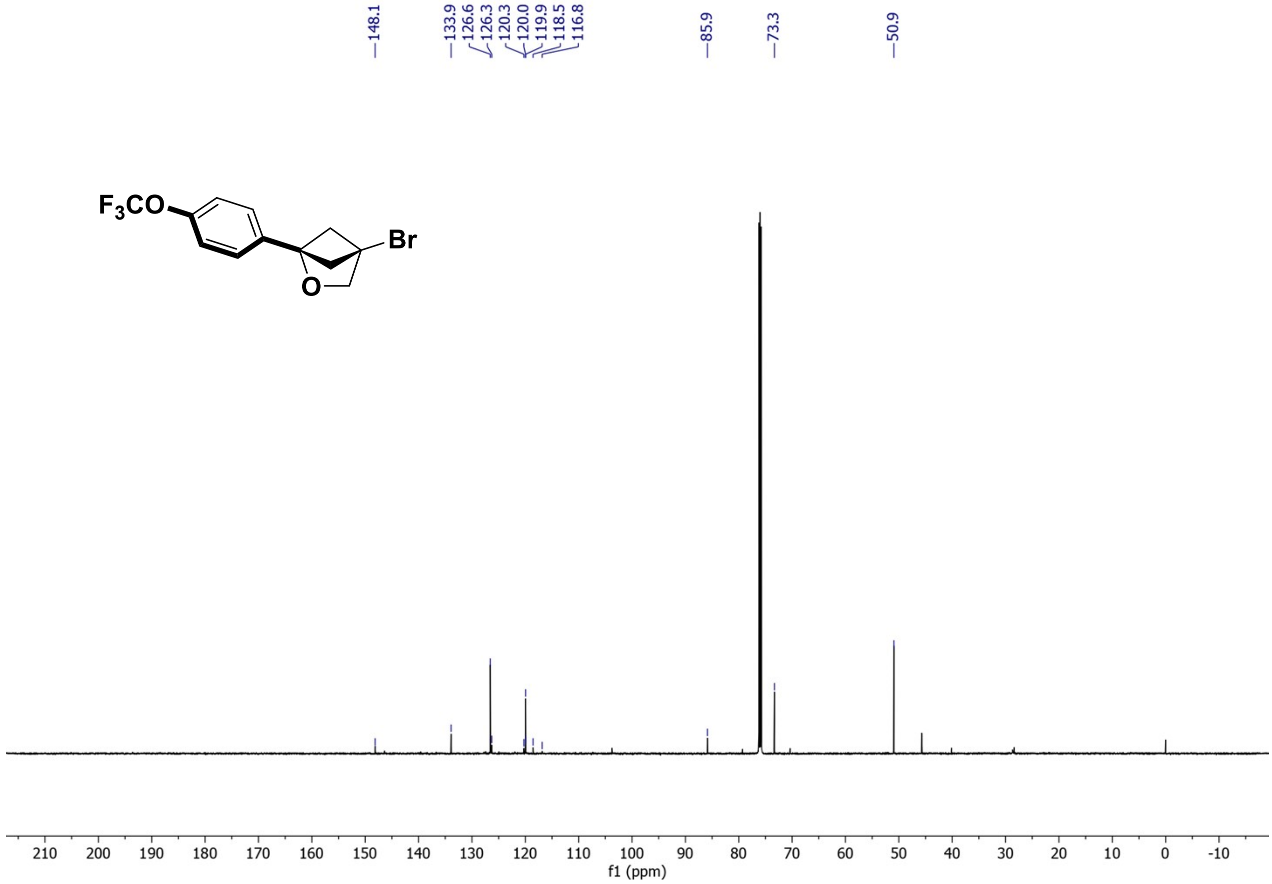

3kf ^{19}F NMR (576 MHz, $CDCl_3$)

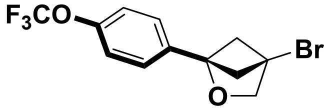

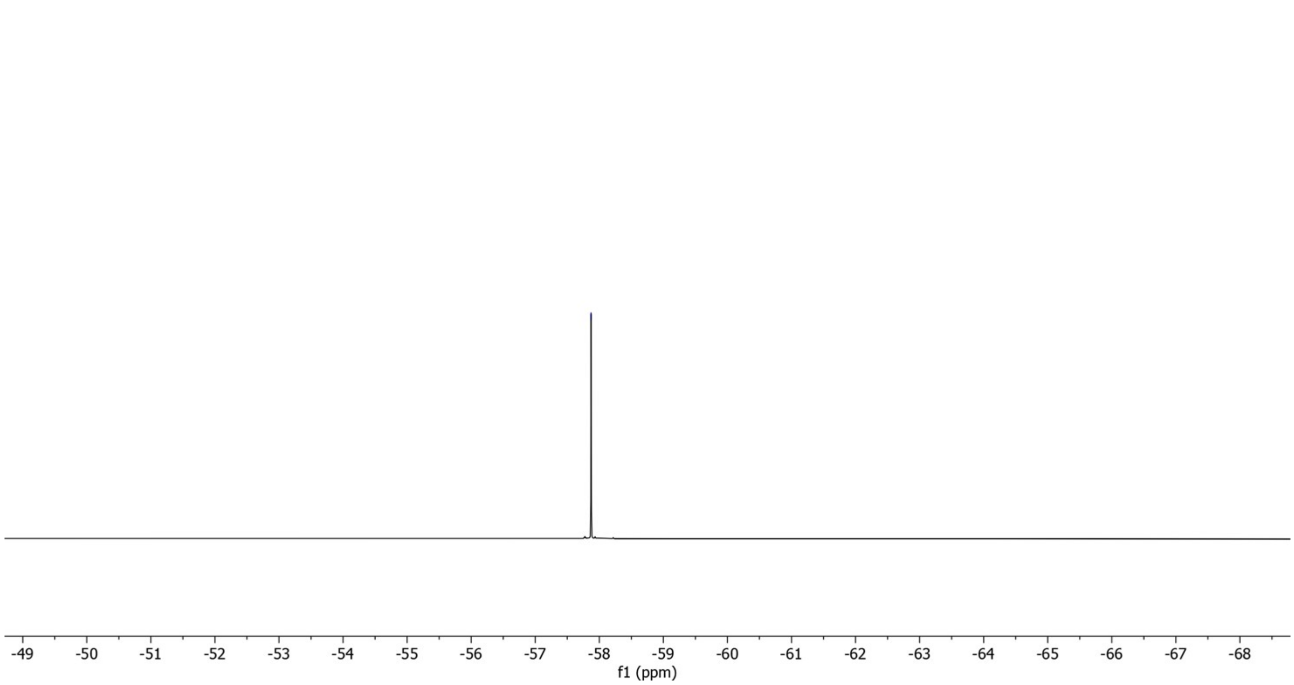

3ag ^{1}H NMR (600 MHz, $CDCl_3$)

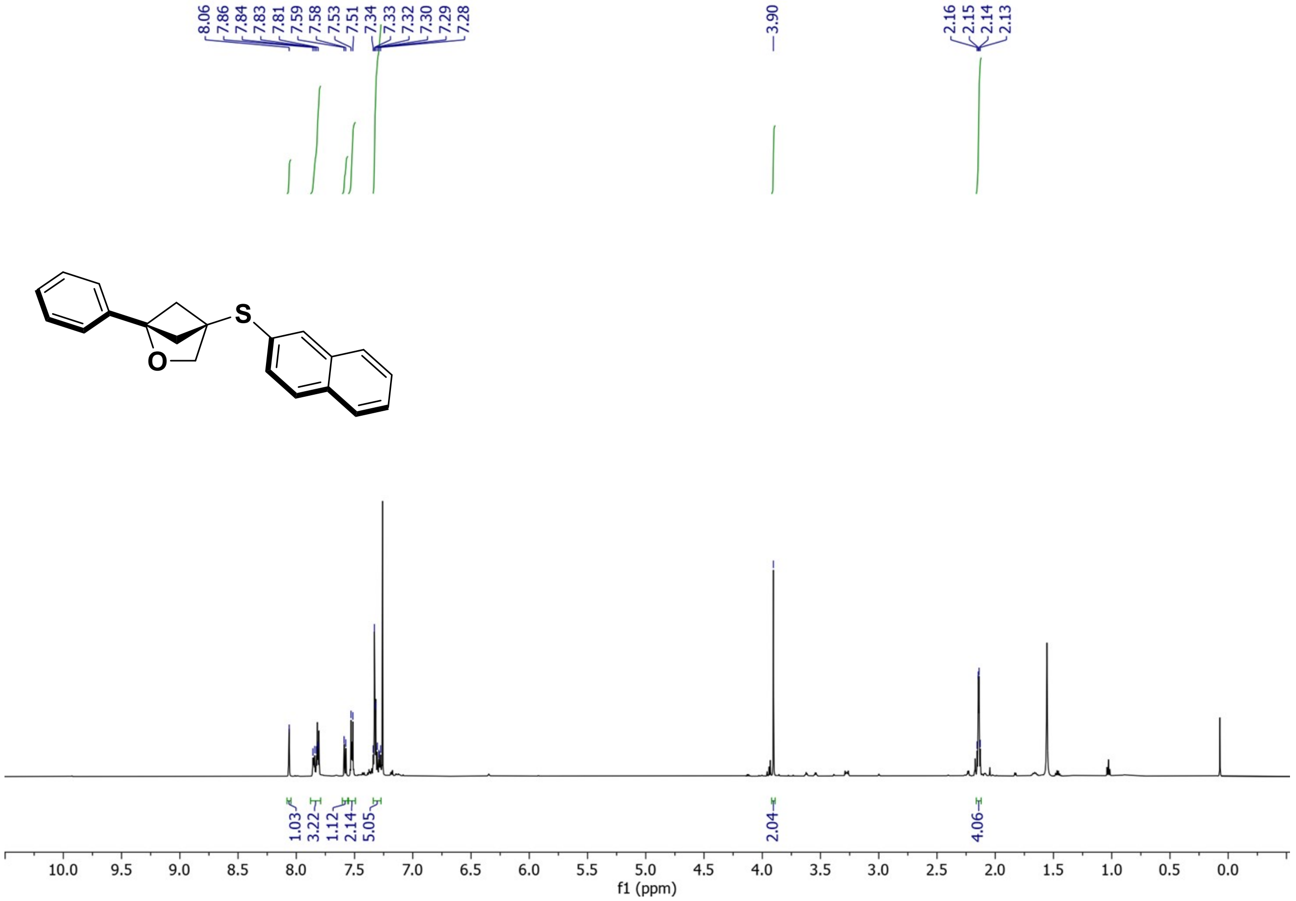

3ag ^{13}C NMR (151 MHz, $CDCl_3$)

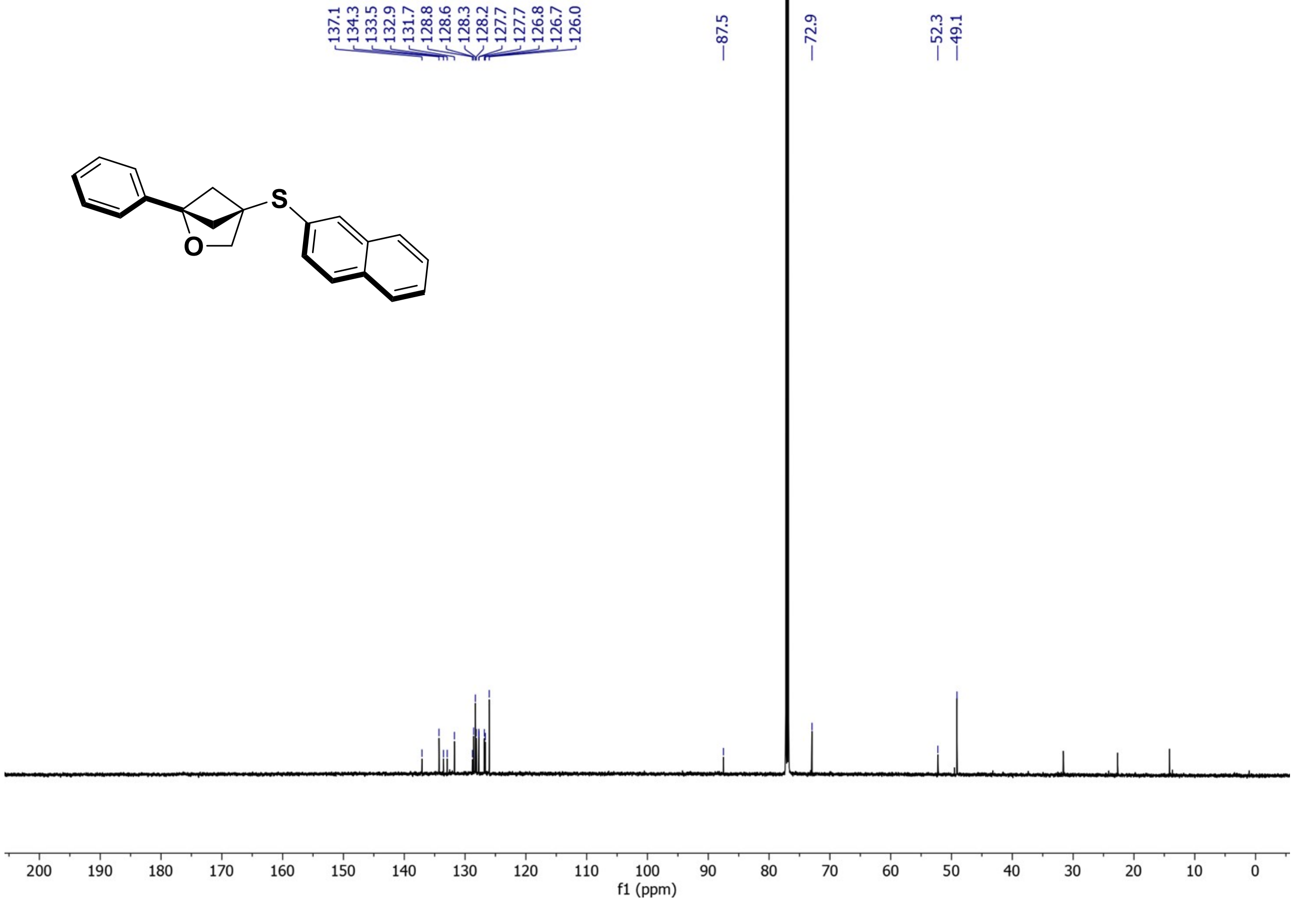

3ah ^{1}H NMR (600 MHz, $CDCl_3$)

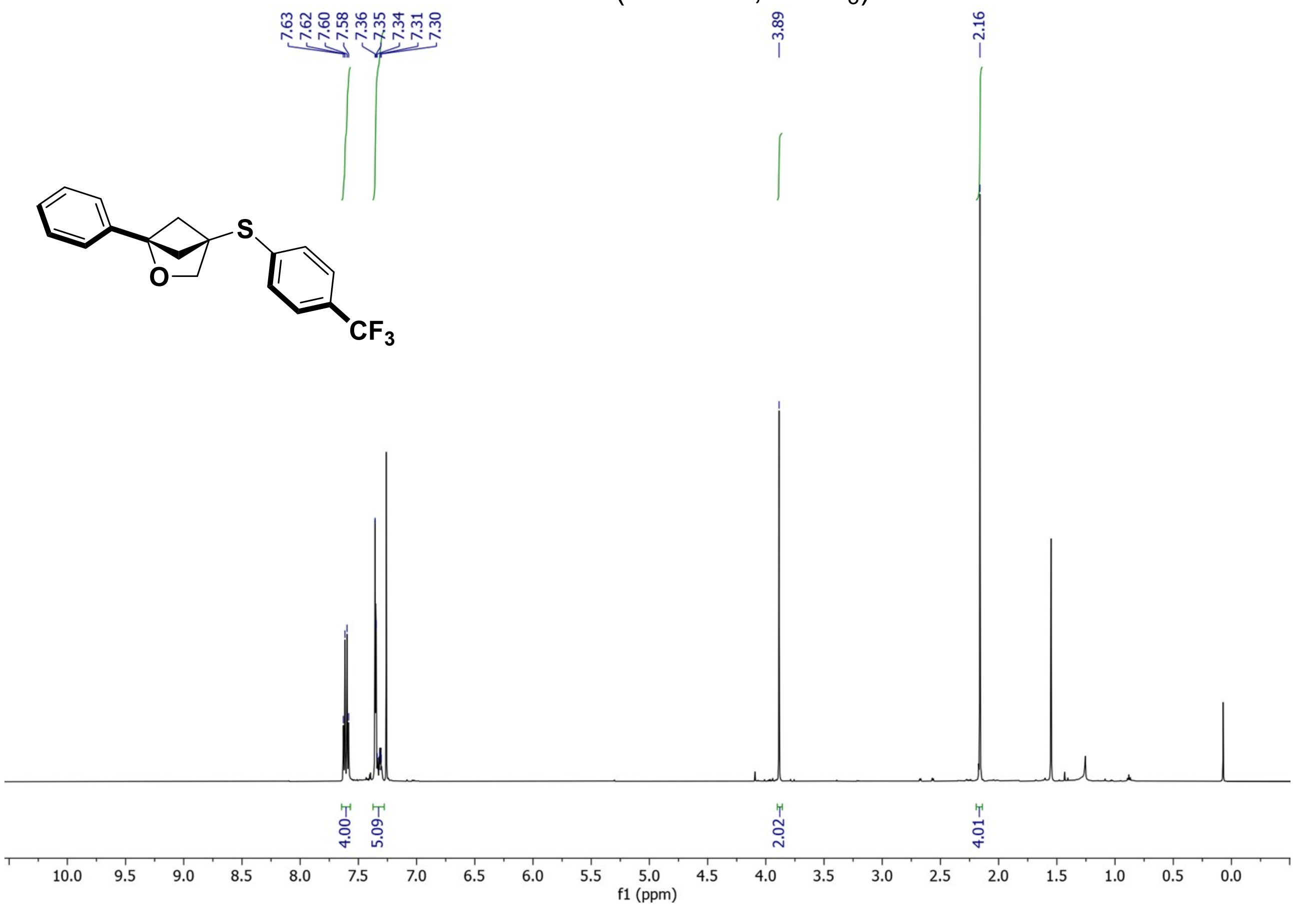

3ah ^{13}C NMR (151 MHz, $CDCl_3$)

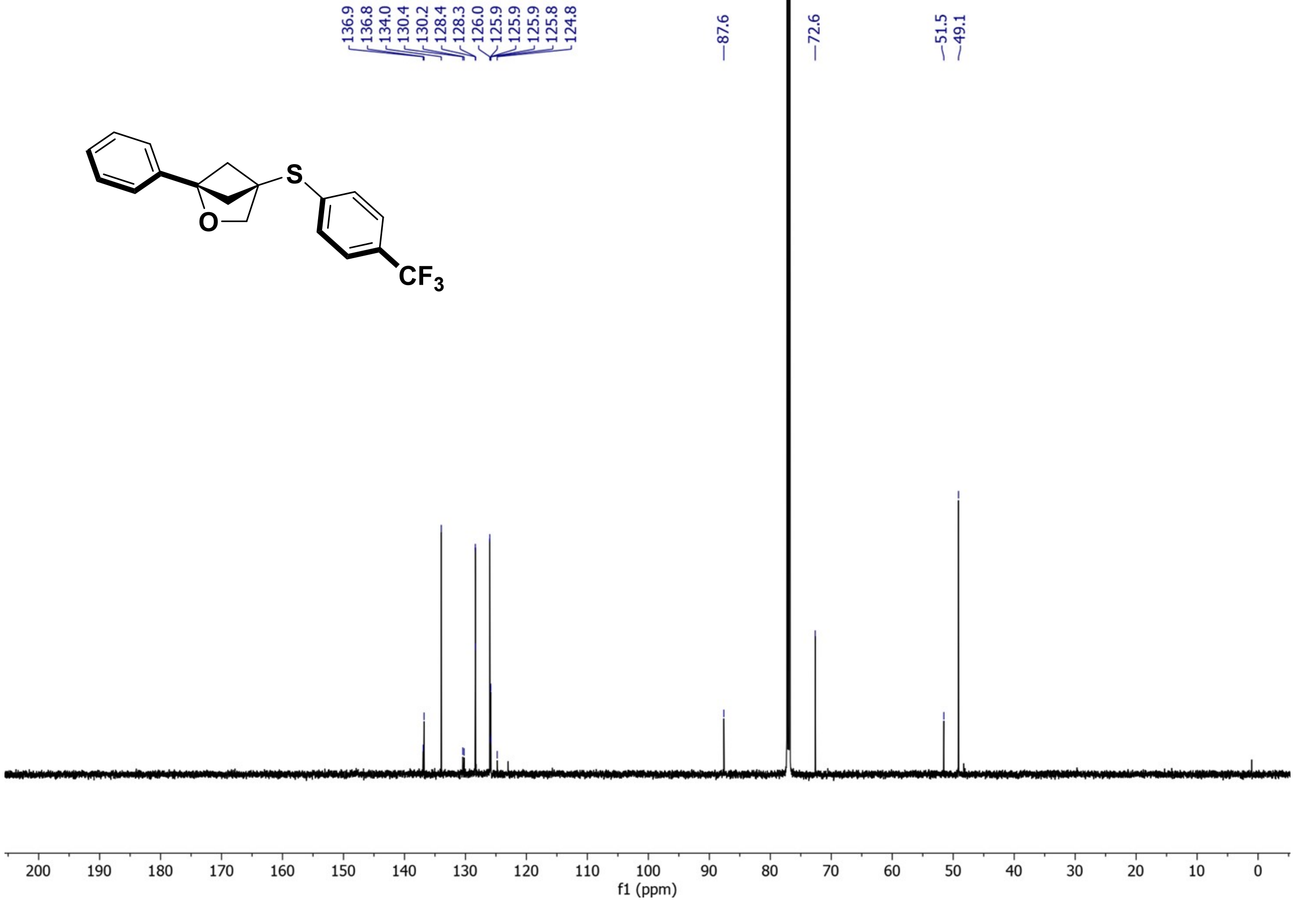

3ah ^{19}F NMR (565 MHz, $CDCl_3$)

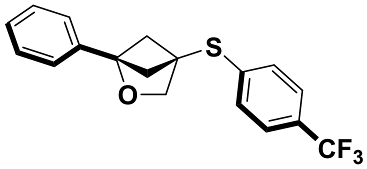

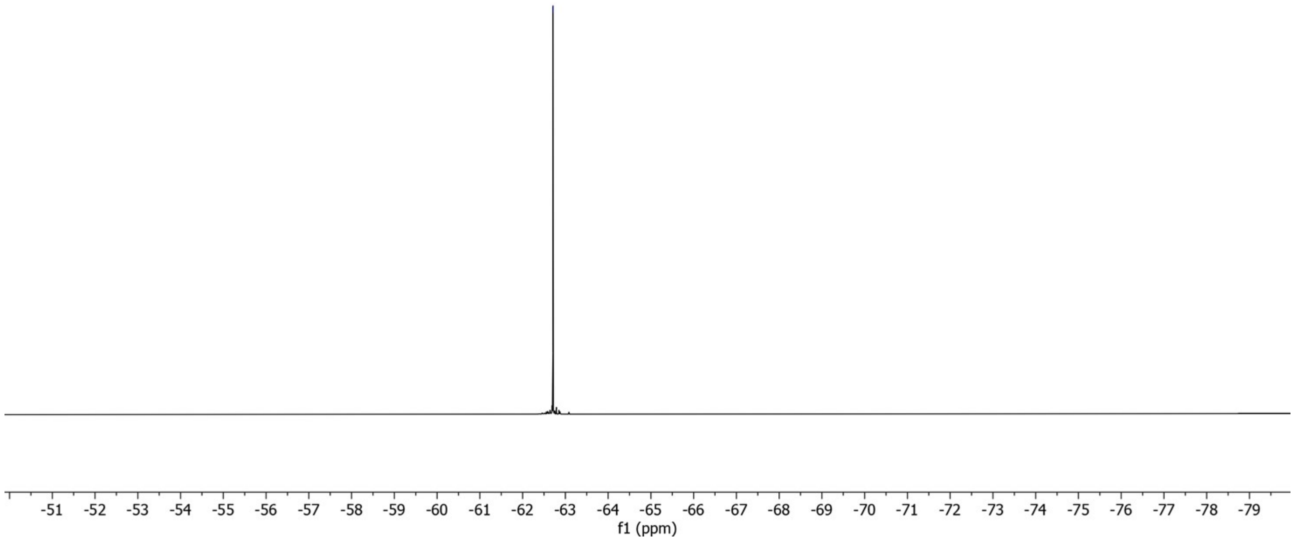

3ai ^{1}H NMR (600 MHz, $CDCl_3$)

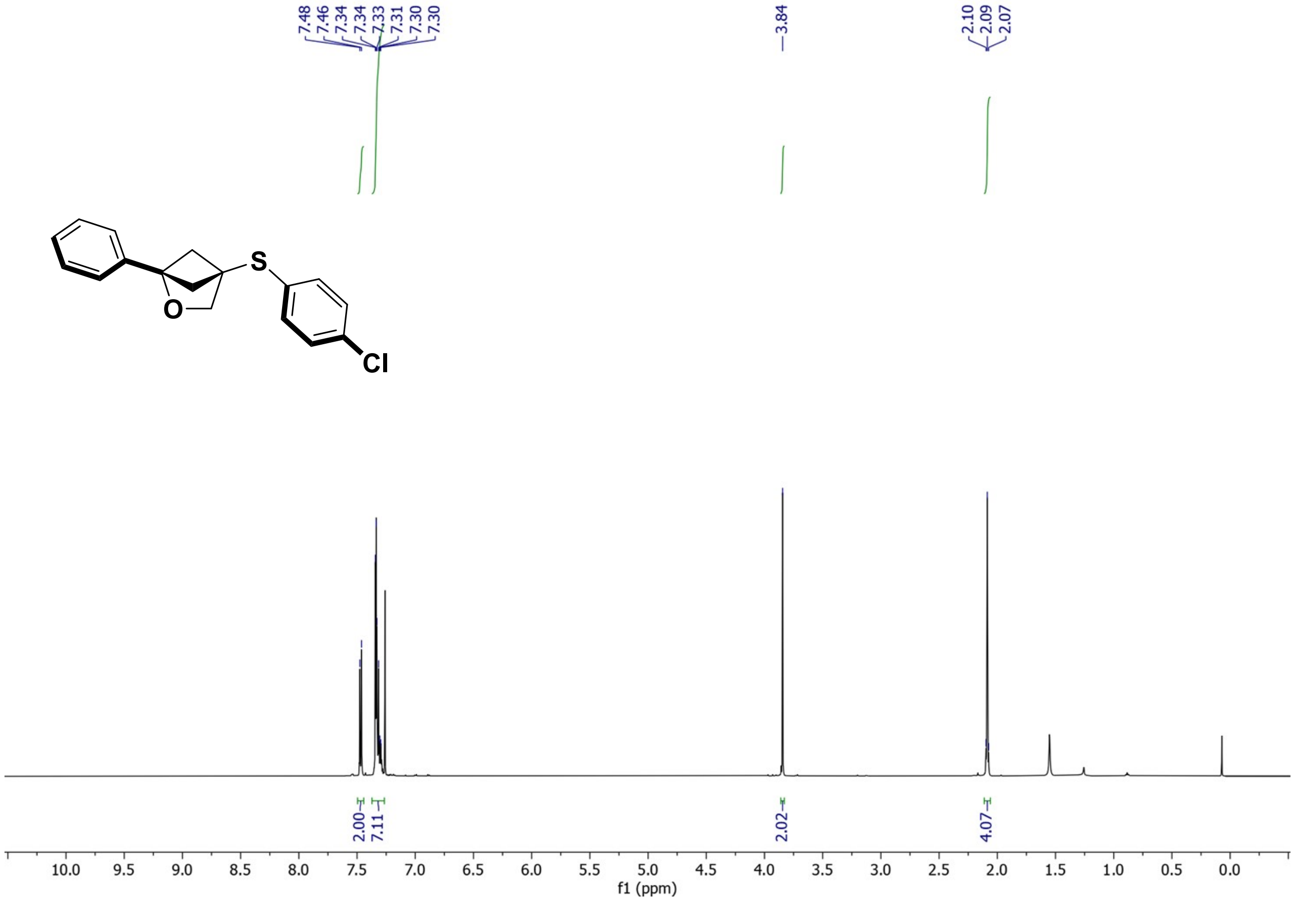

3ai ^{13}C NMR (151 MHz, $CDCl_3$)

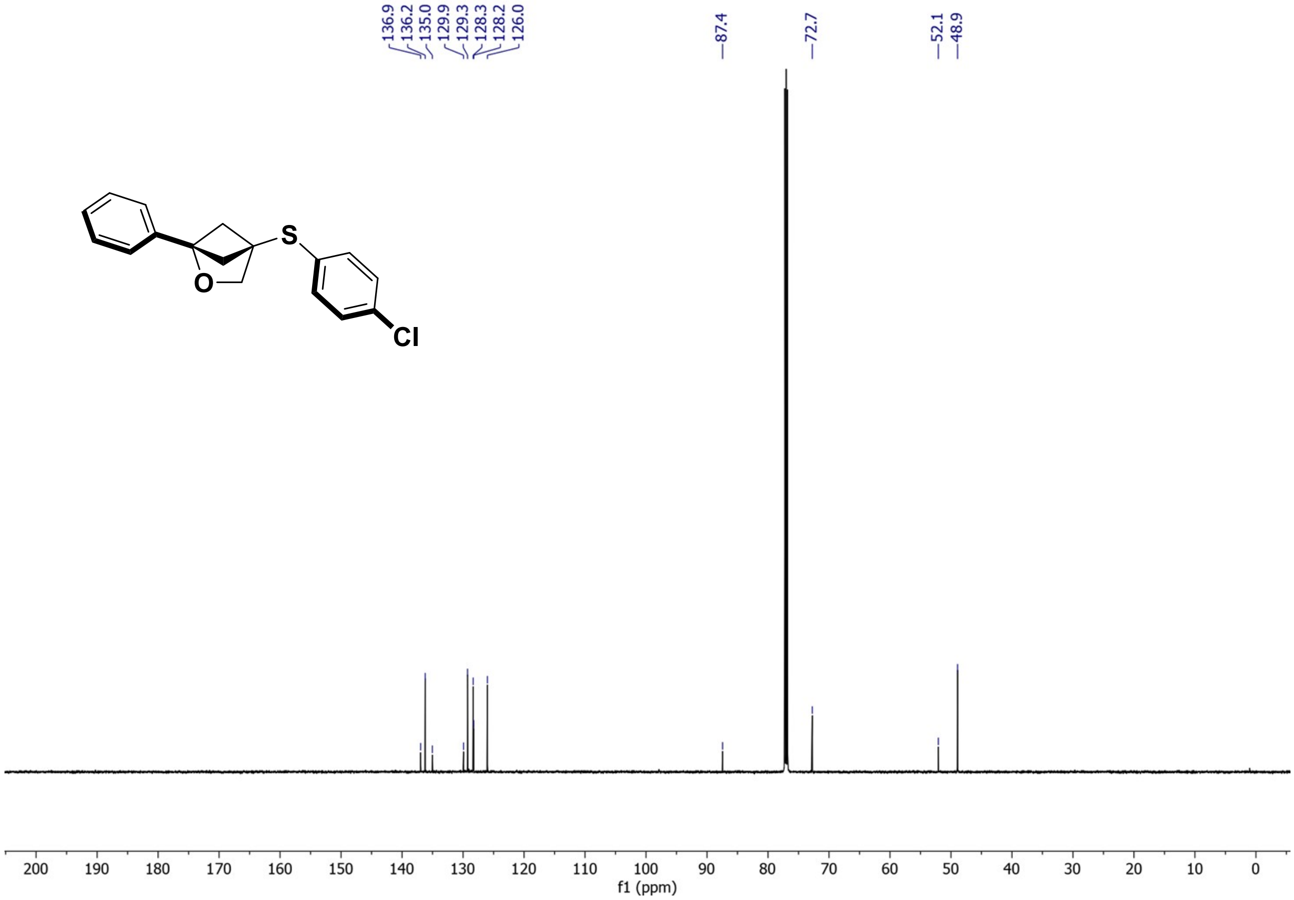

3aj ^{1}**H NMR** (600 MHz, $CDCl_3$)

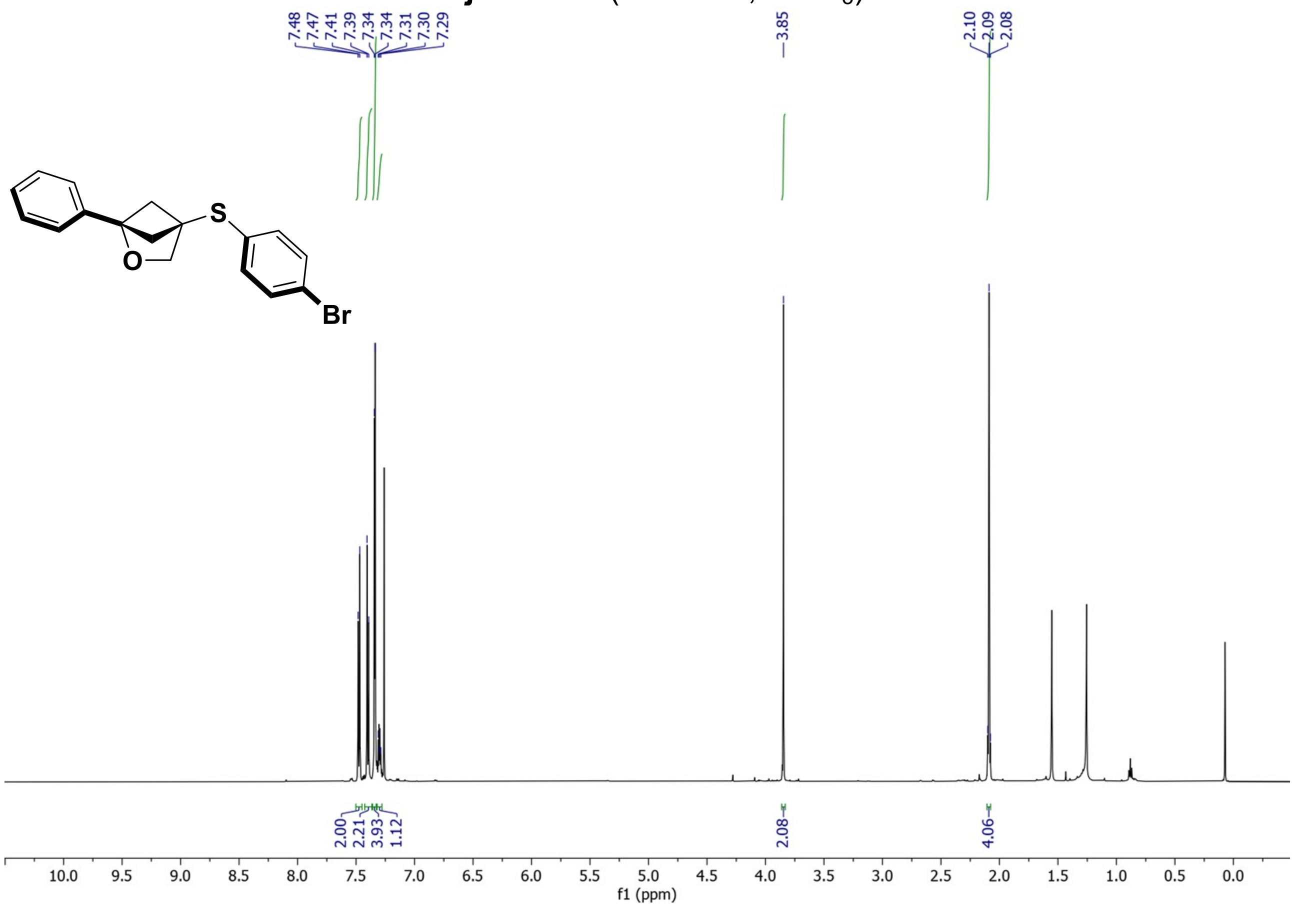

3aj ^{13}**C NMR** (151 MHz, $CDCl_3$)

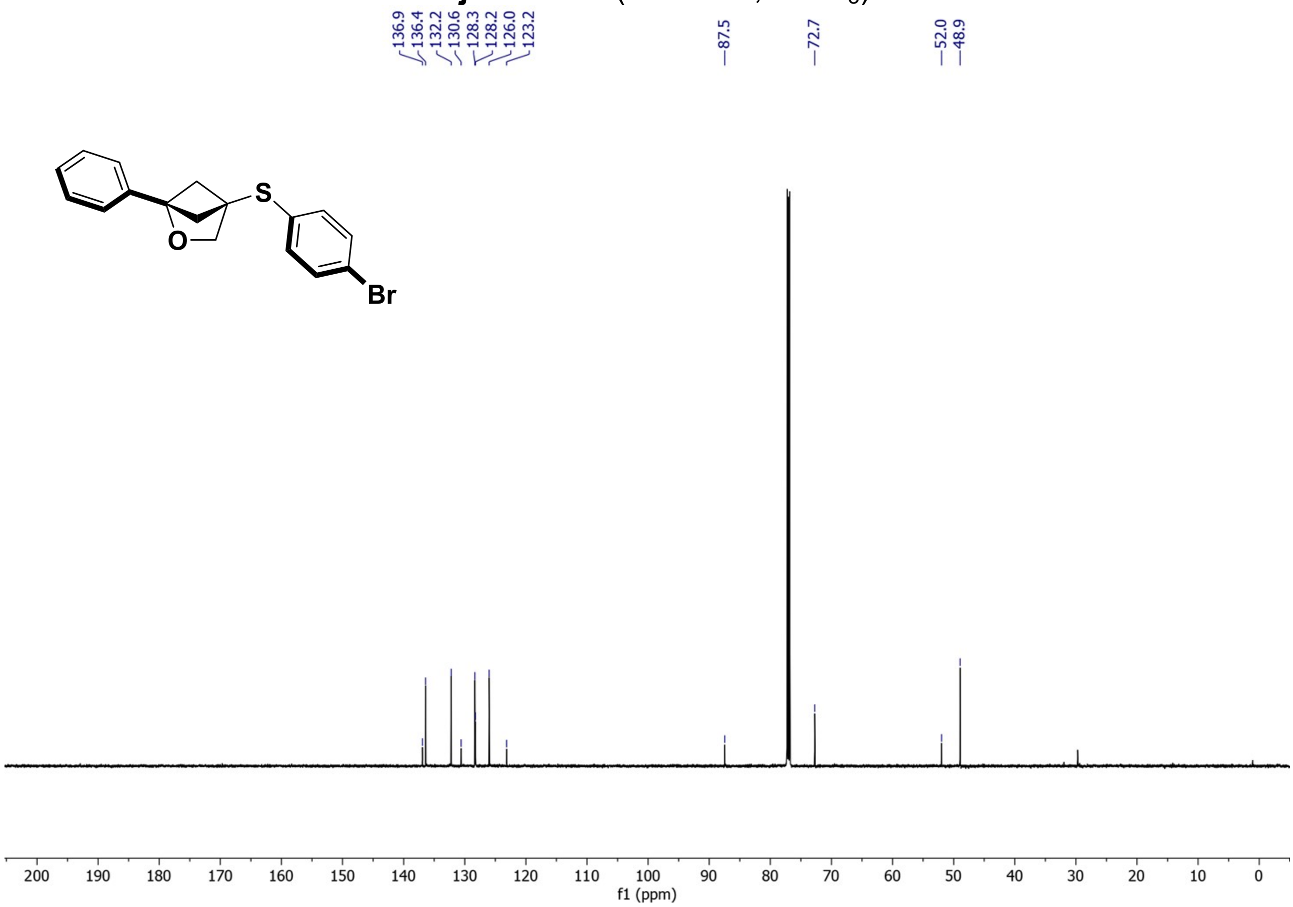

***d*$_4$-3aa ^{1}H NMR** (600 MHz, $CDCl_3$)

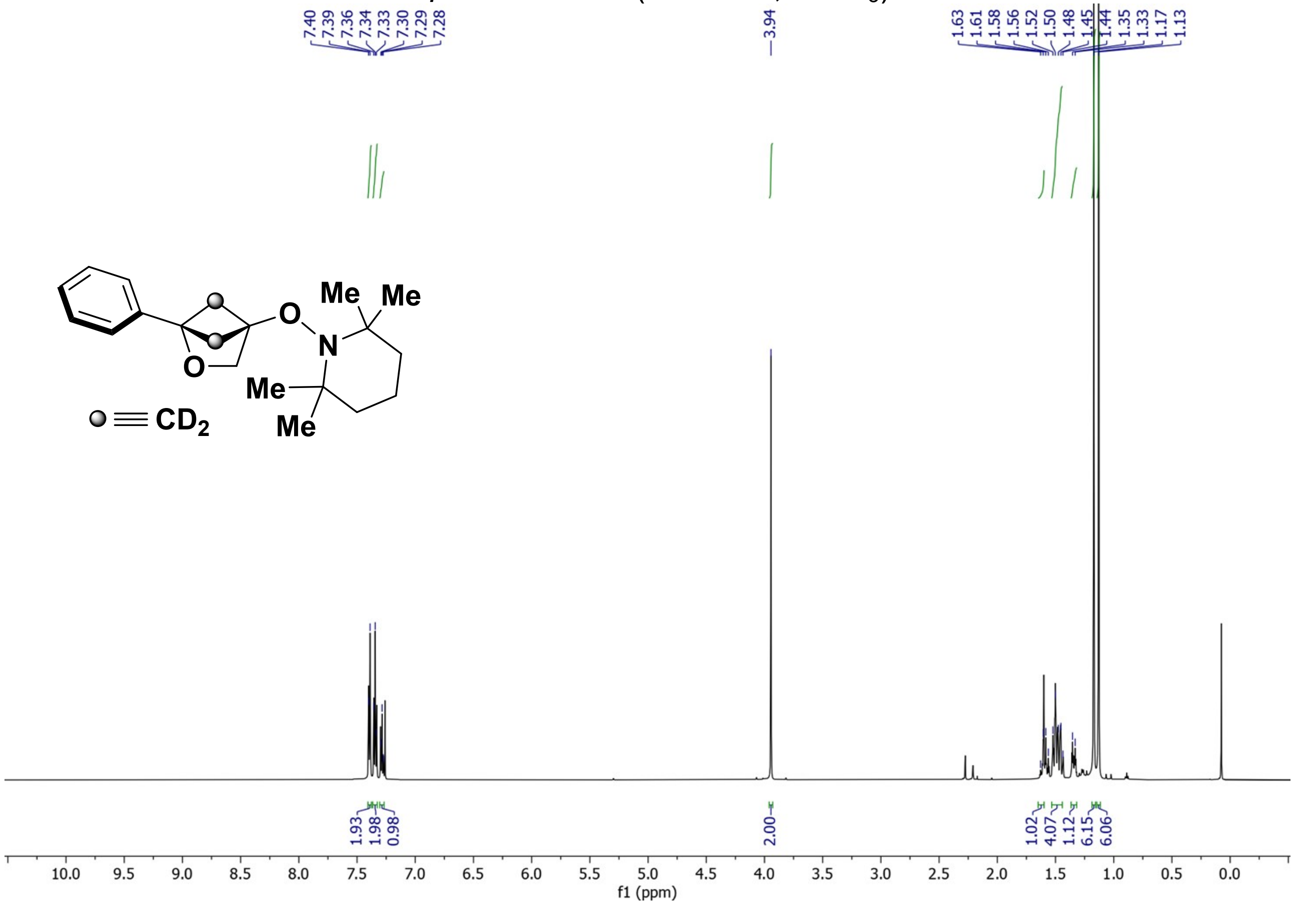

***d*$_4$-3aa ^{13}C NMR** (151 MHz, $CDCl_3$)

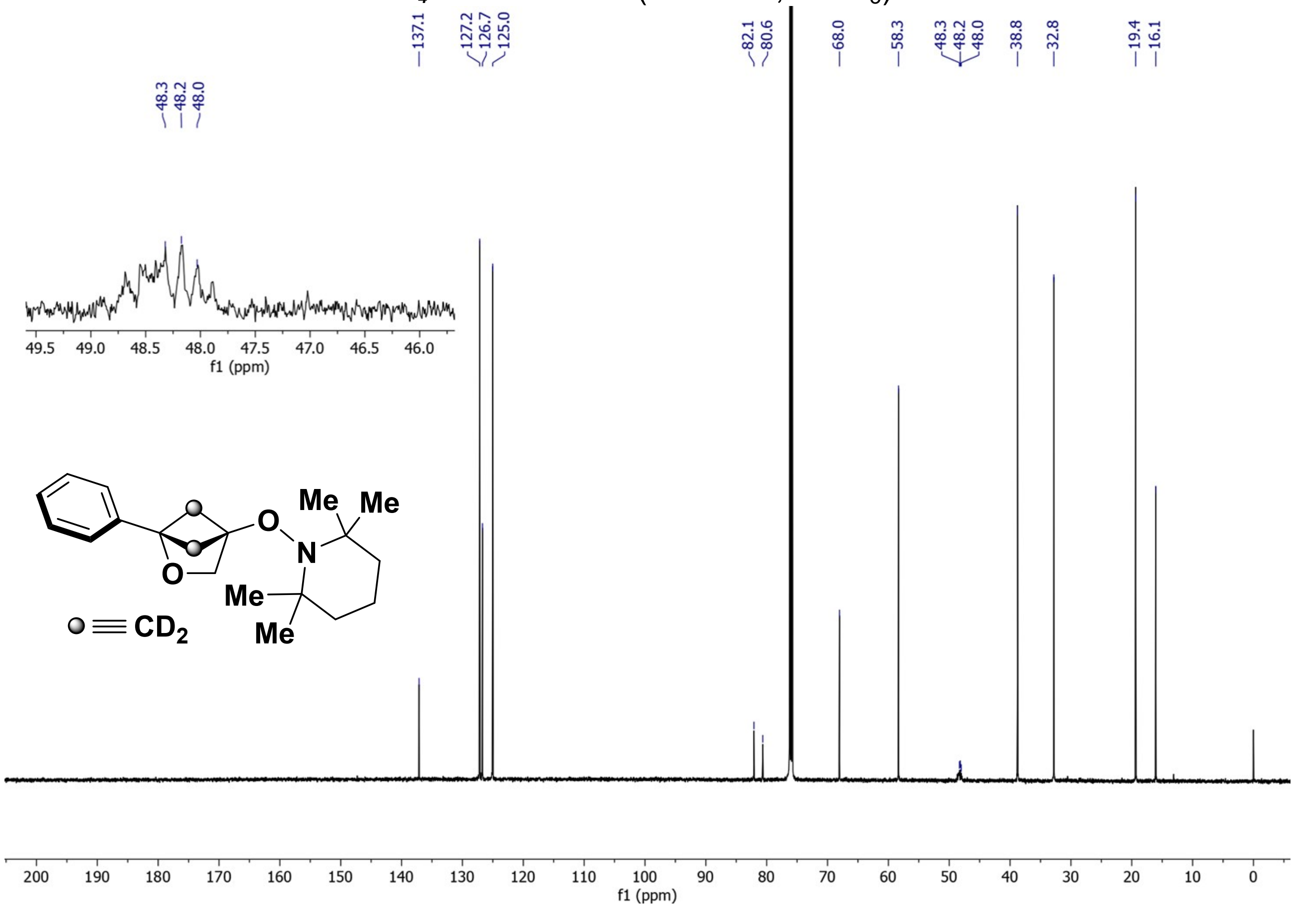

4aa ¹H NMR (600 MHz, $CDCl_3$)

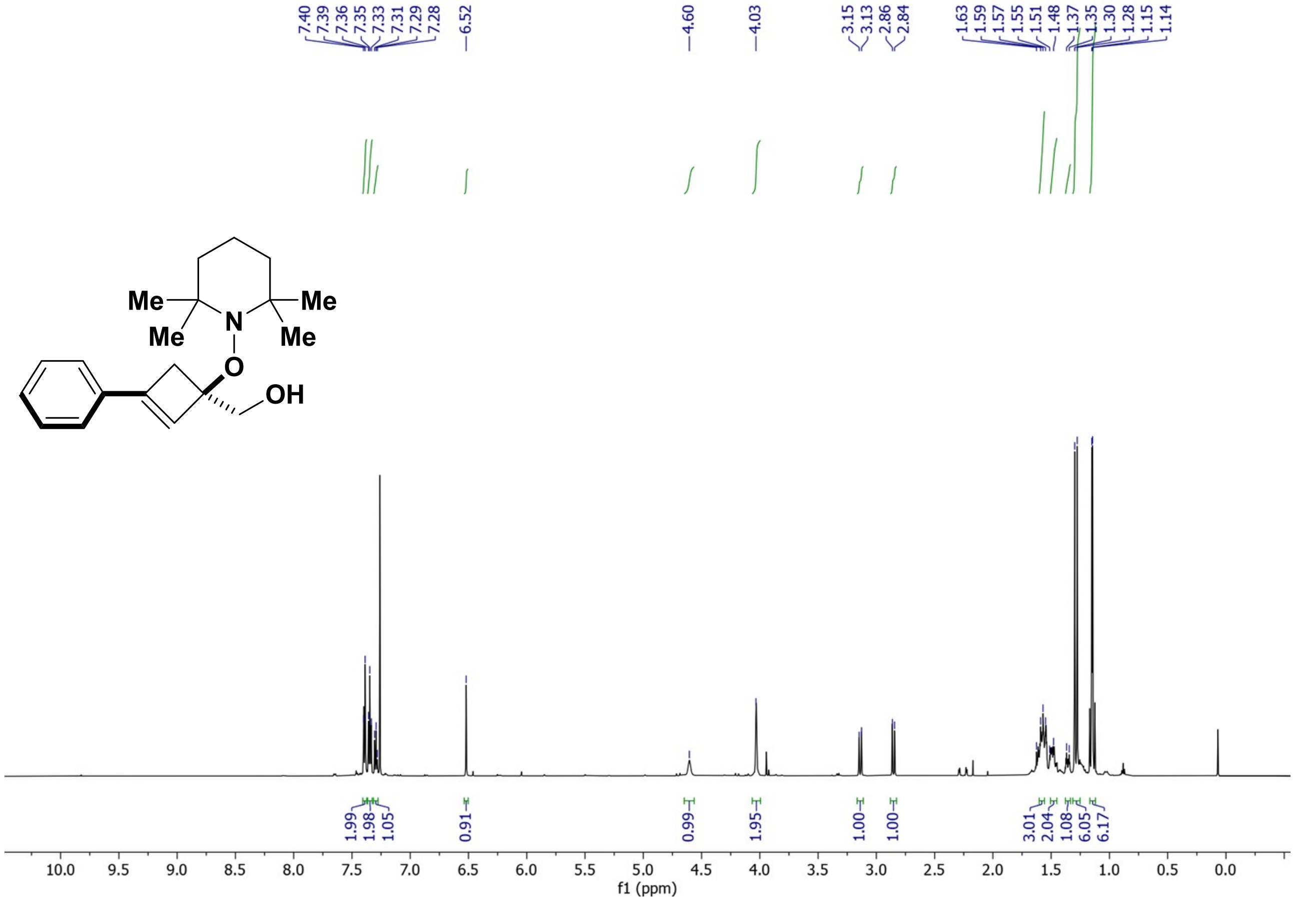

4aa ¹³C NMR (151 MHz, $CDCl_3$)

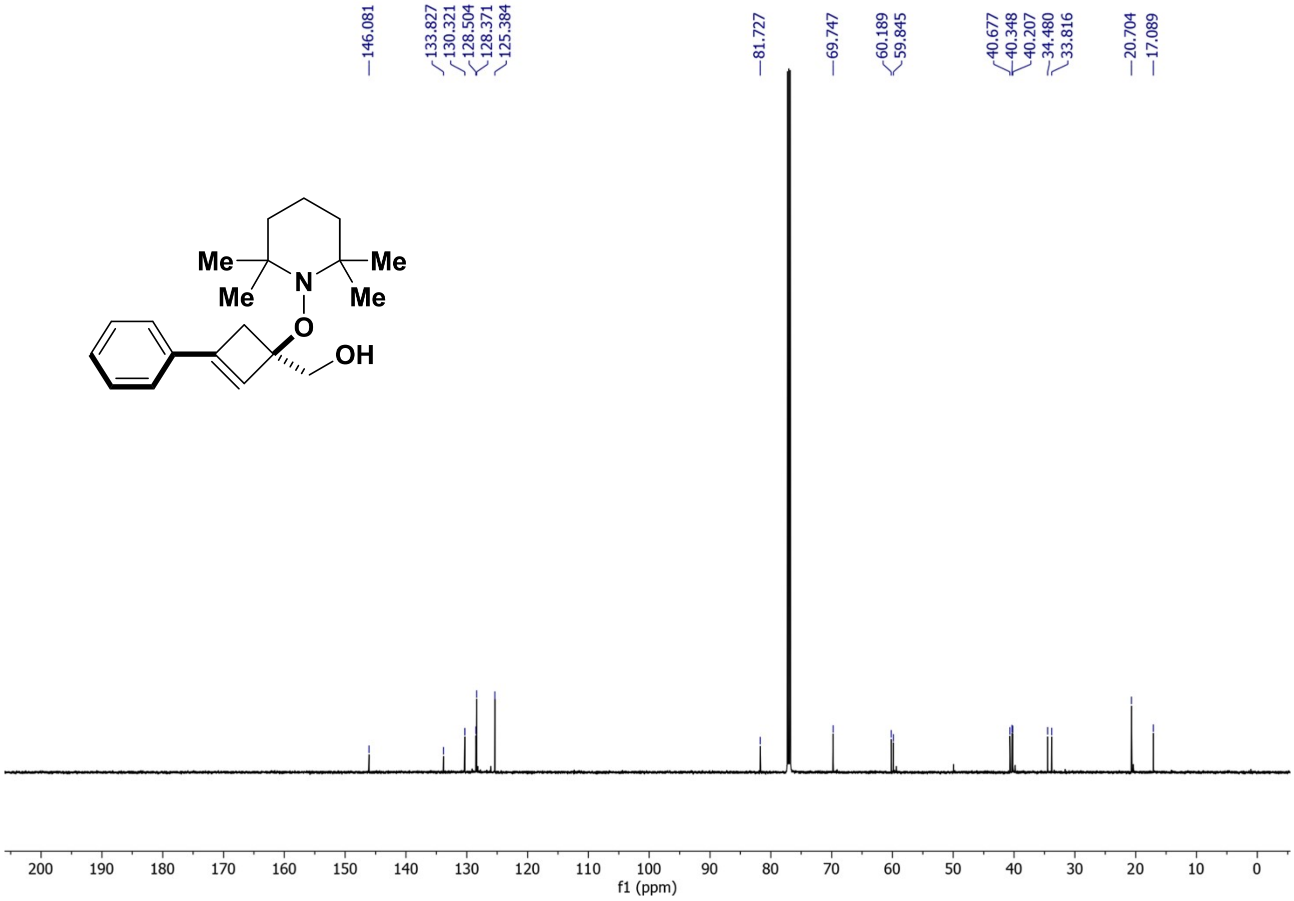

6 ^{1}H NMR (600 MHz, $CDCl_3$)

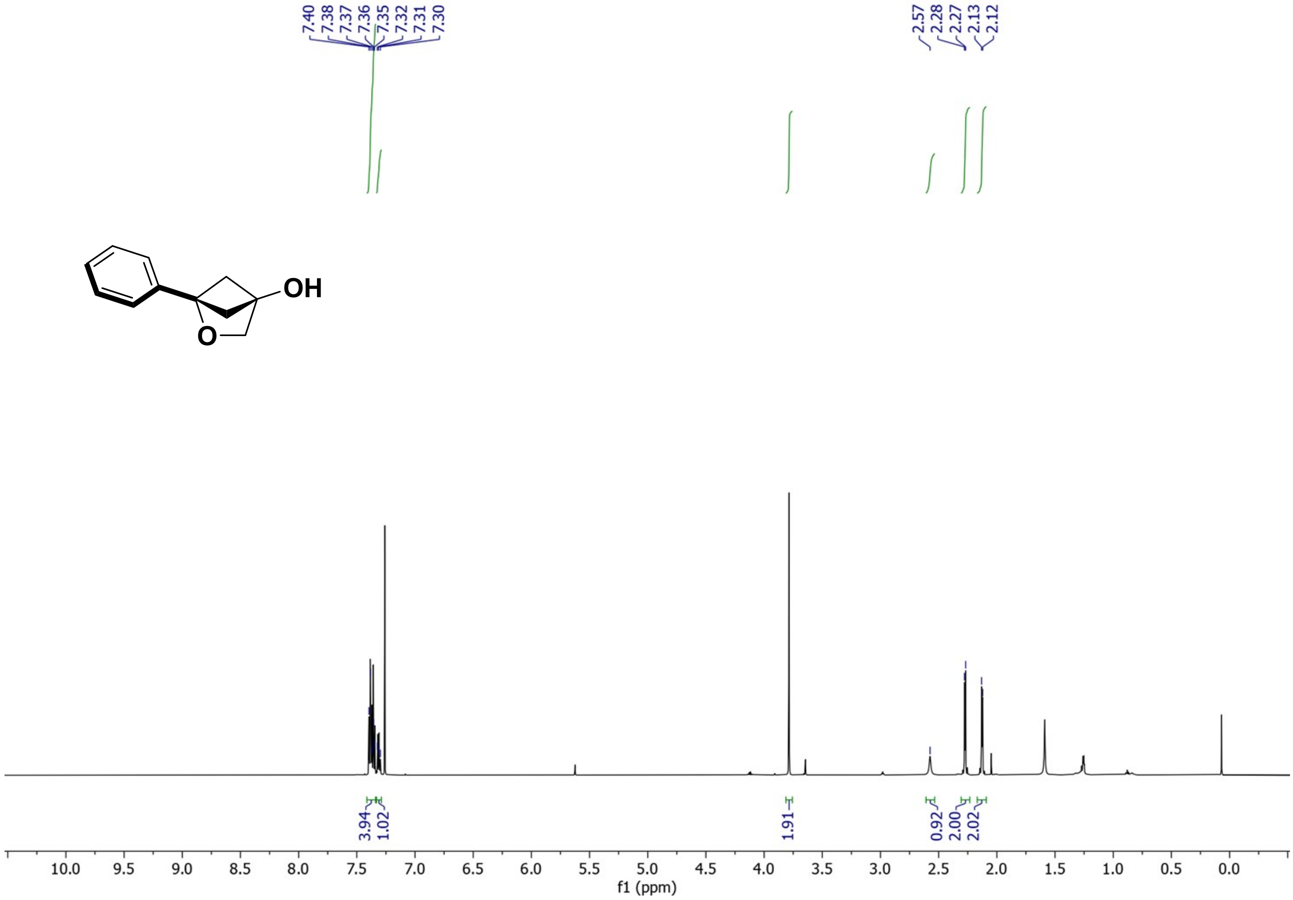

6 ^{13}C NMR (151 MHz, $CDCl_3$)

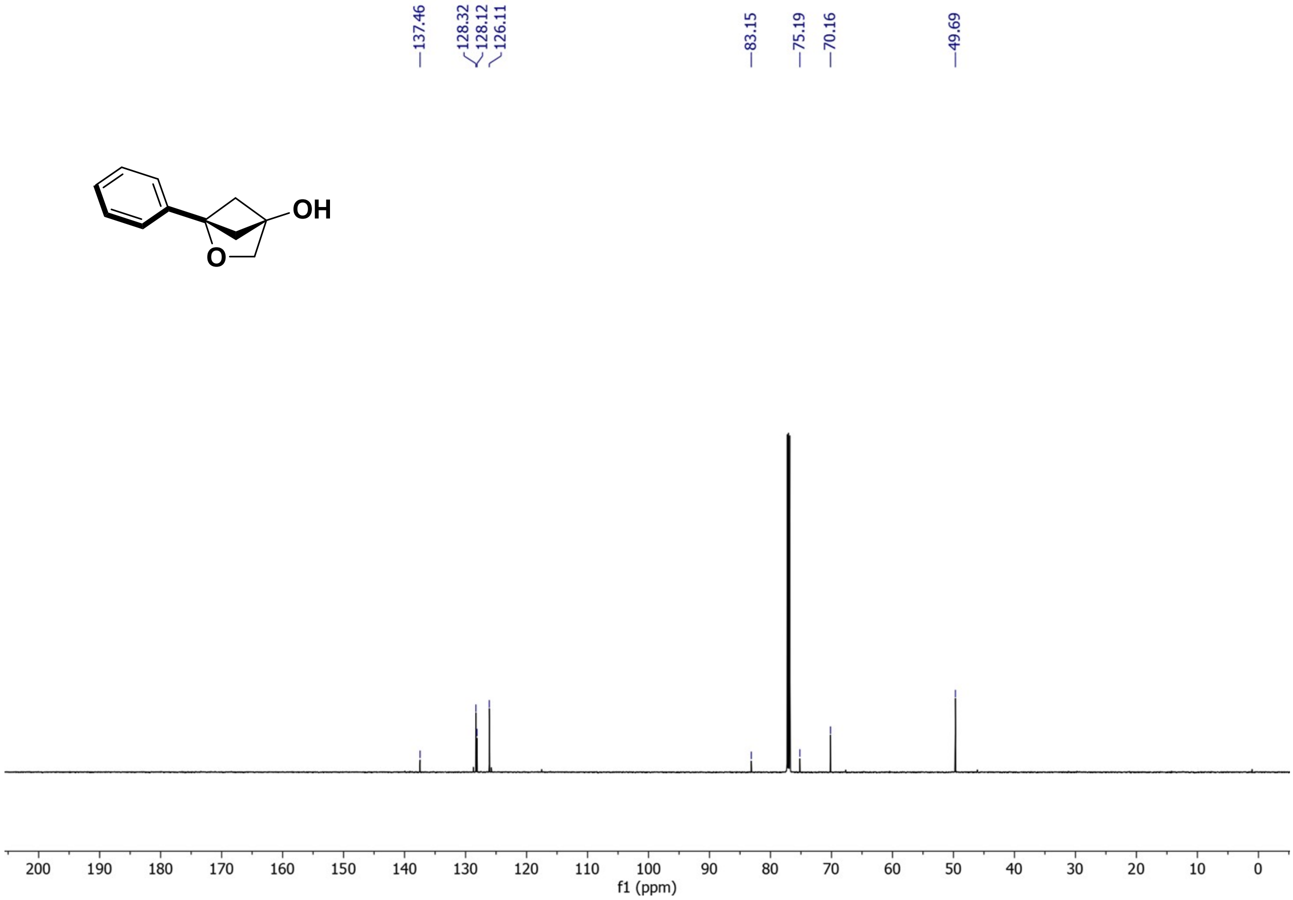

7 ^{1}H NMR (600 MHz, $CDCl_3$)

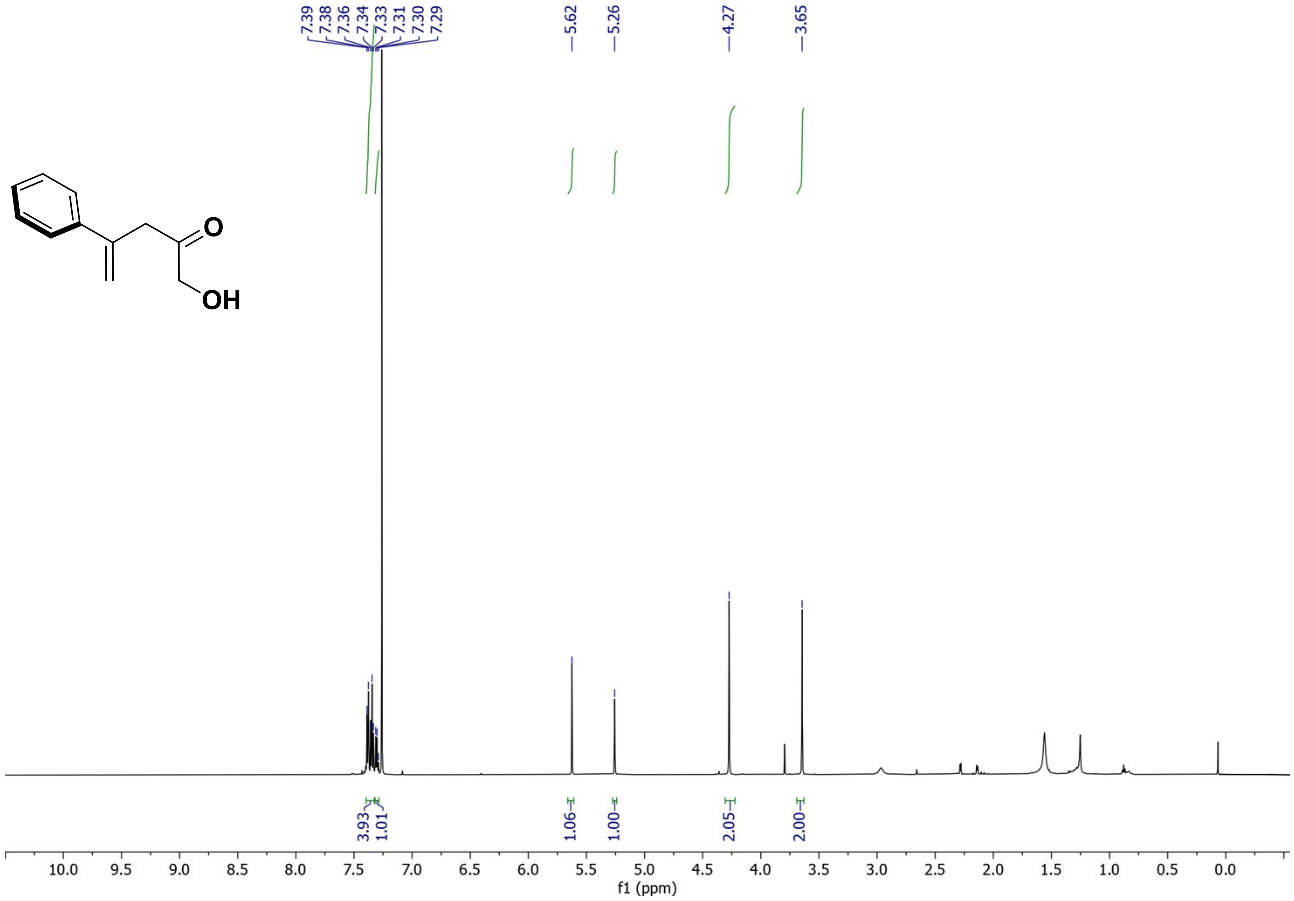

7 ^{13}C NMR (151 MHz, $CDCl_3$)

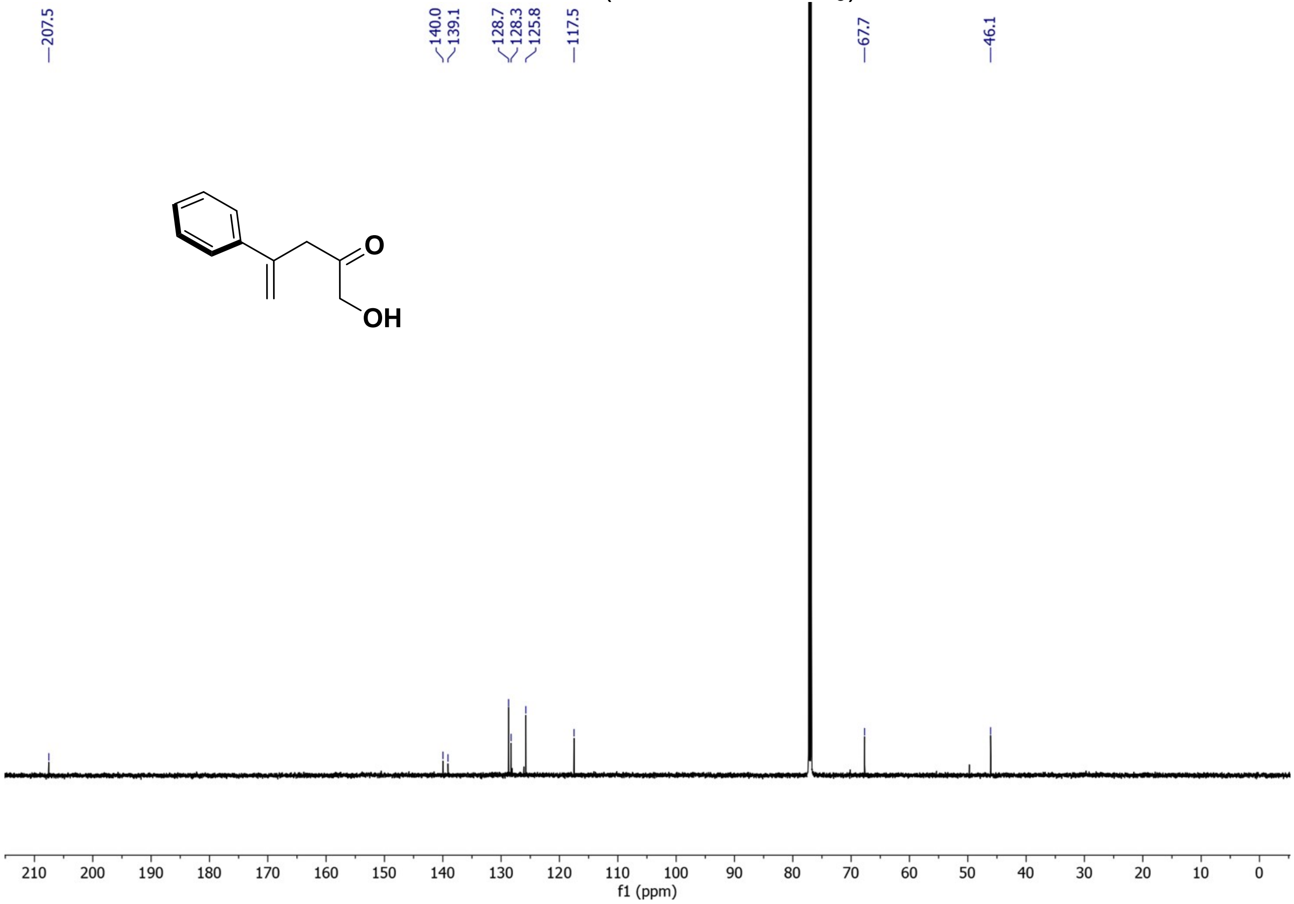

8 ^{1}H NMR (600 MHz, $CDCl_3$)

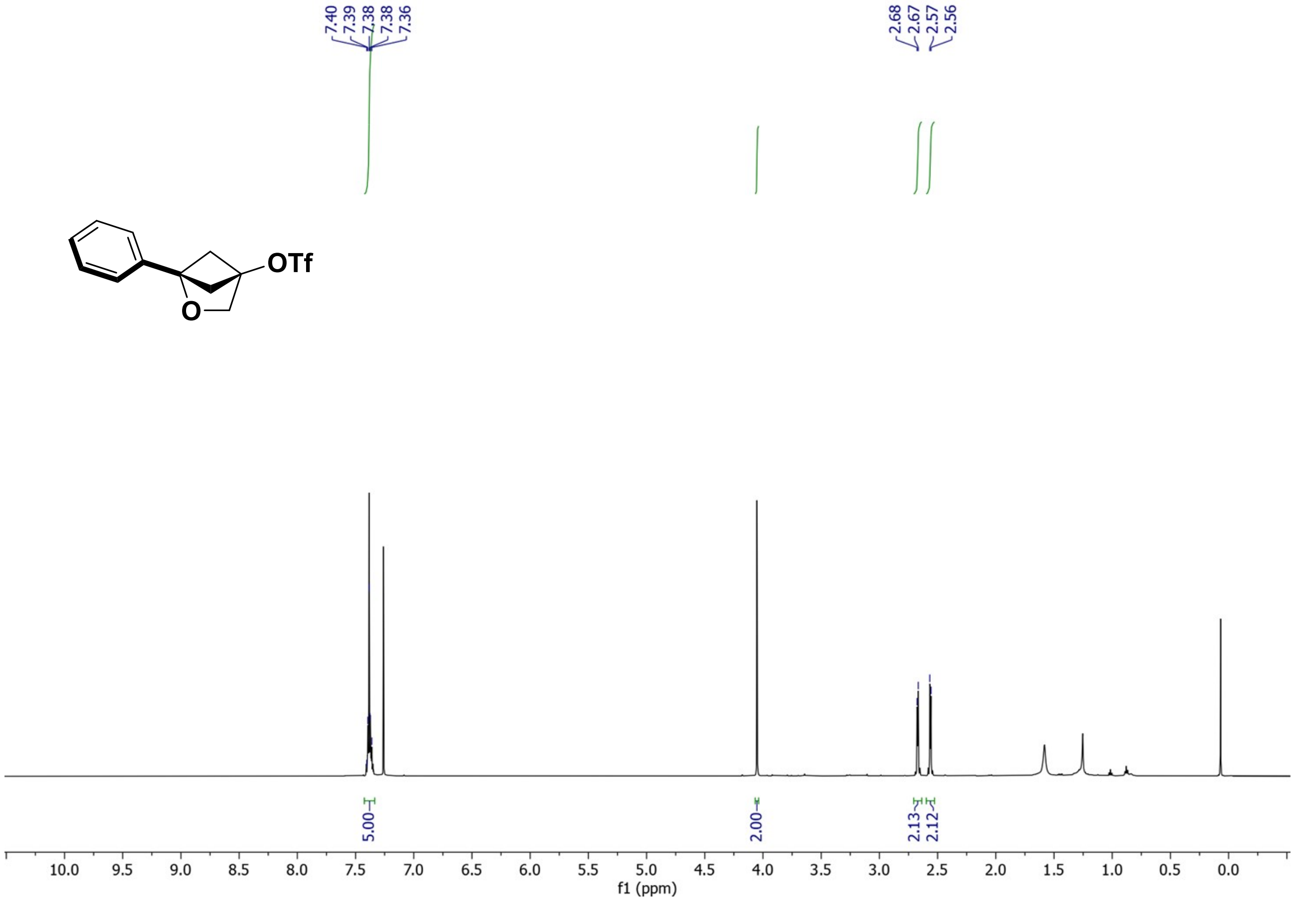

8 ^{13}C NMR (151 MHz, $CDCl_3$)

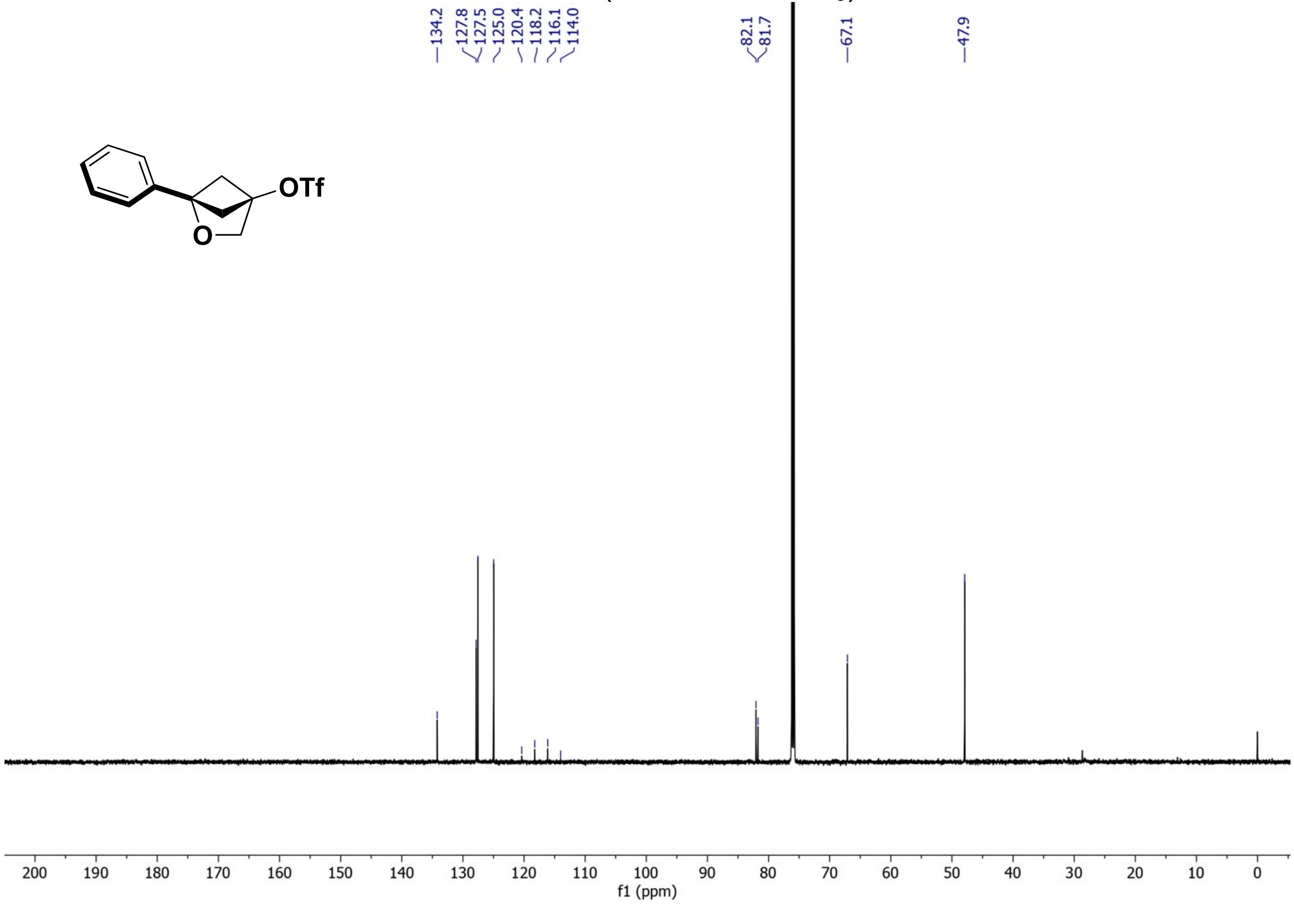

8 ¹⁹F NMR (565 MHz, $CDCl_3$)

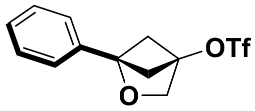

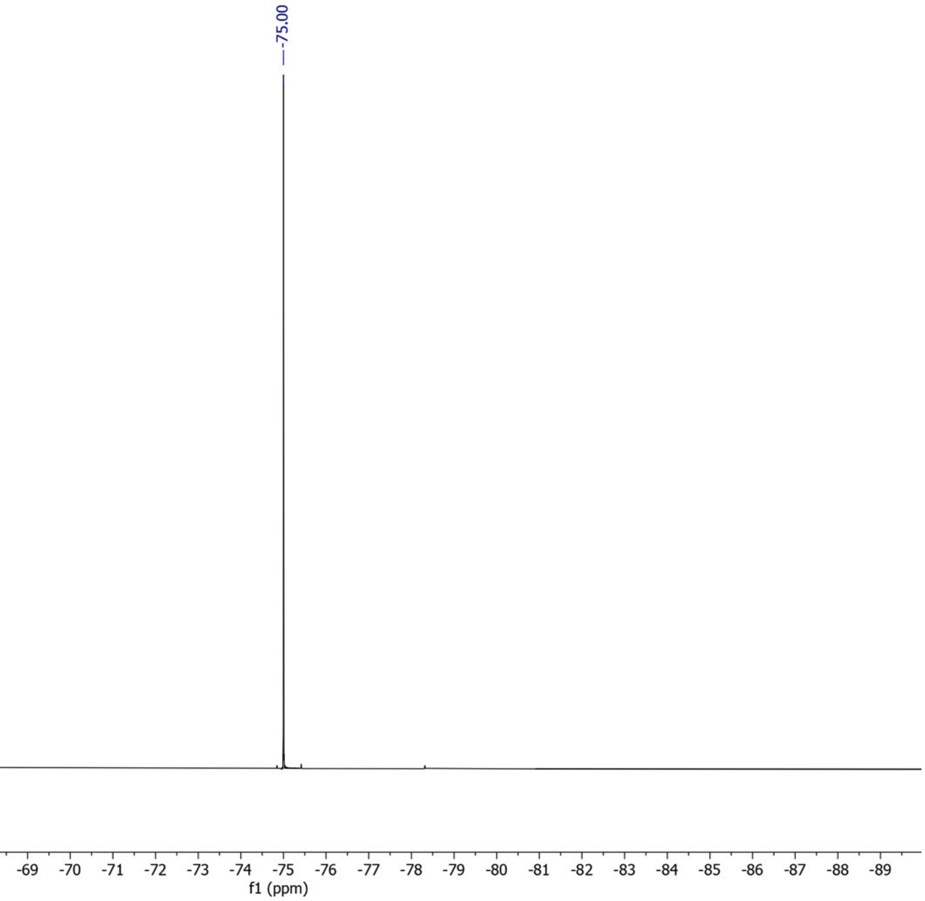

9 ^{1}H NMR (600 MHz, $CDCl_3$)

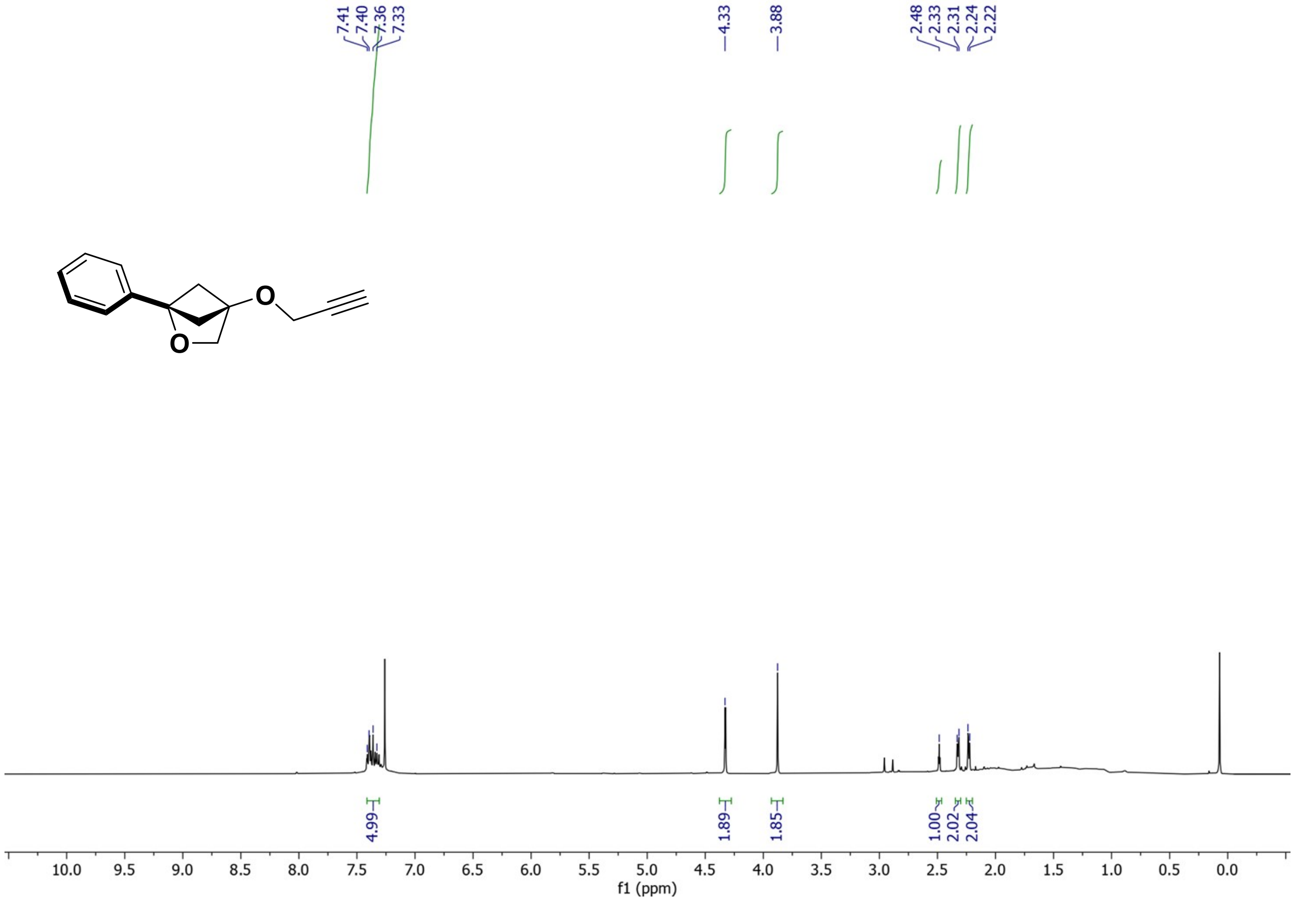

9 ^{13}C NMR (151 MHz, $CDCl_3$)

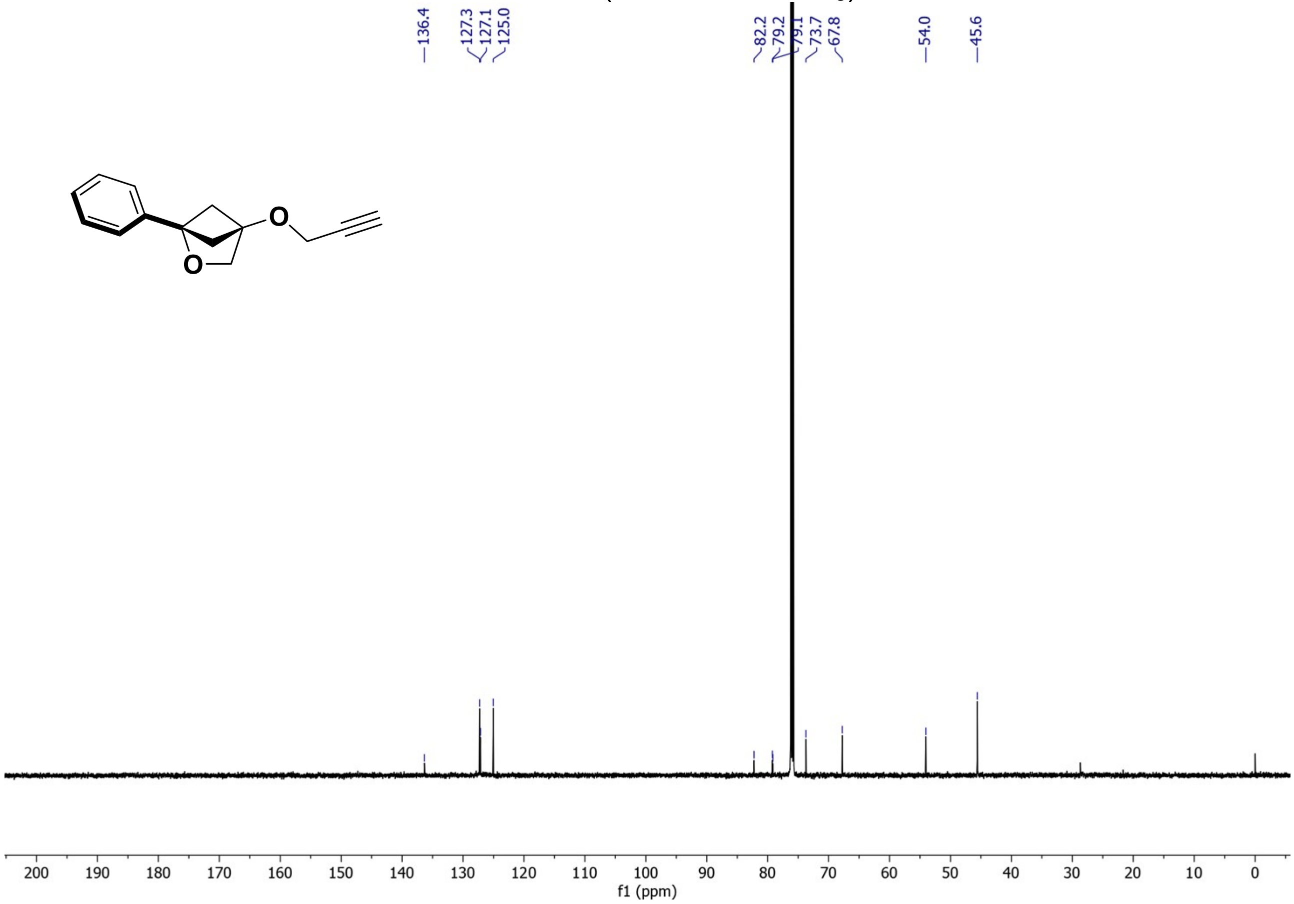

10 ^{1}H NMR (600 MHz, $CDCl_3$)

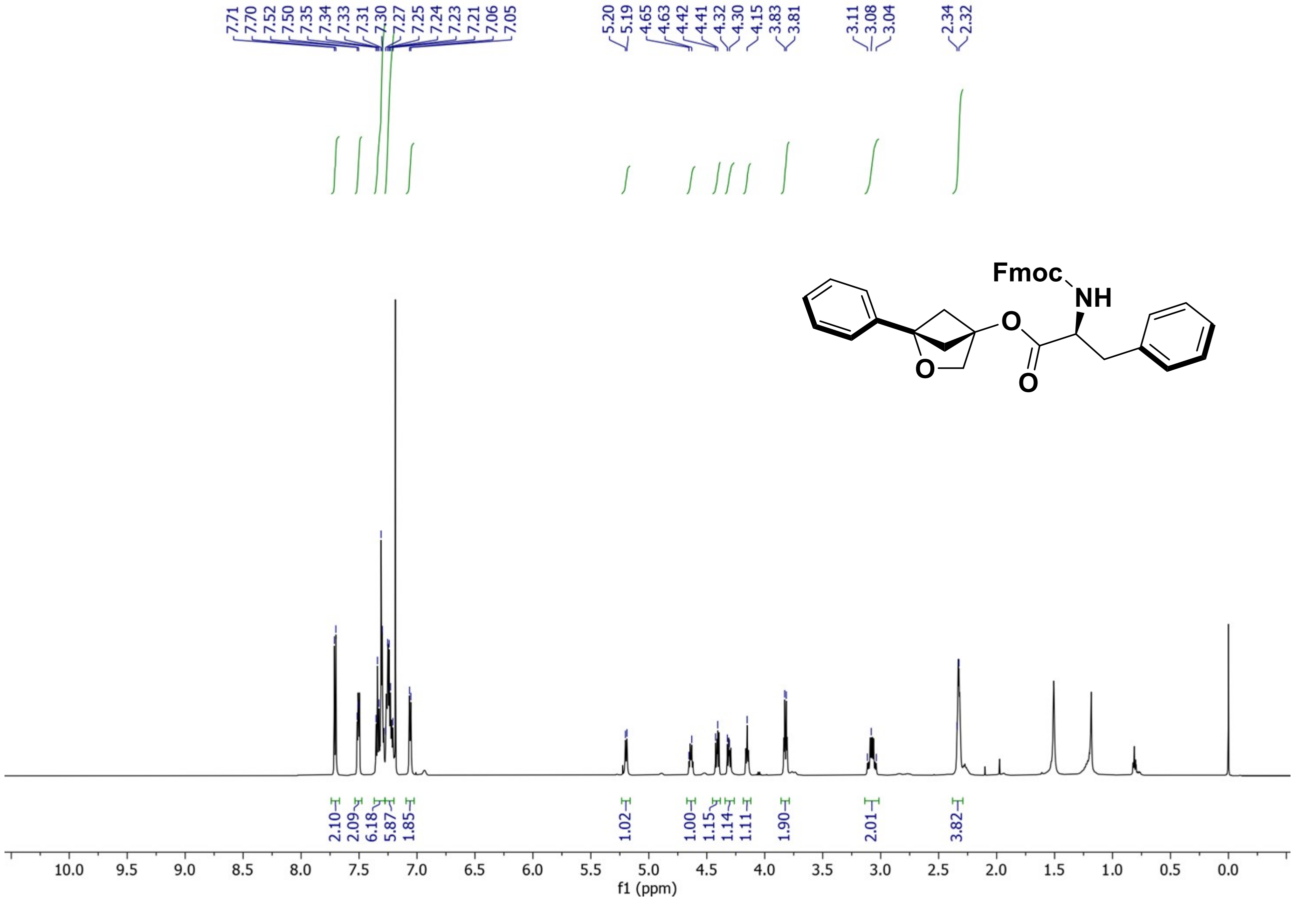

10 ^{13}C NMR (151 MHz, $CDCl_3$)

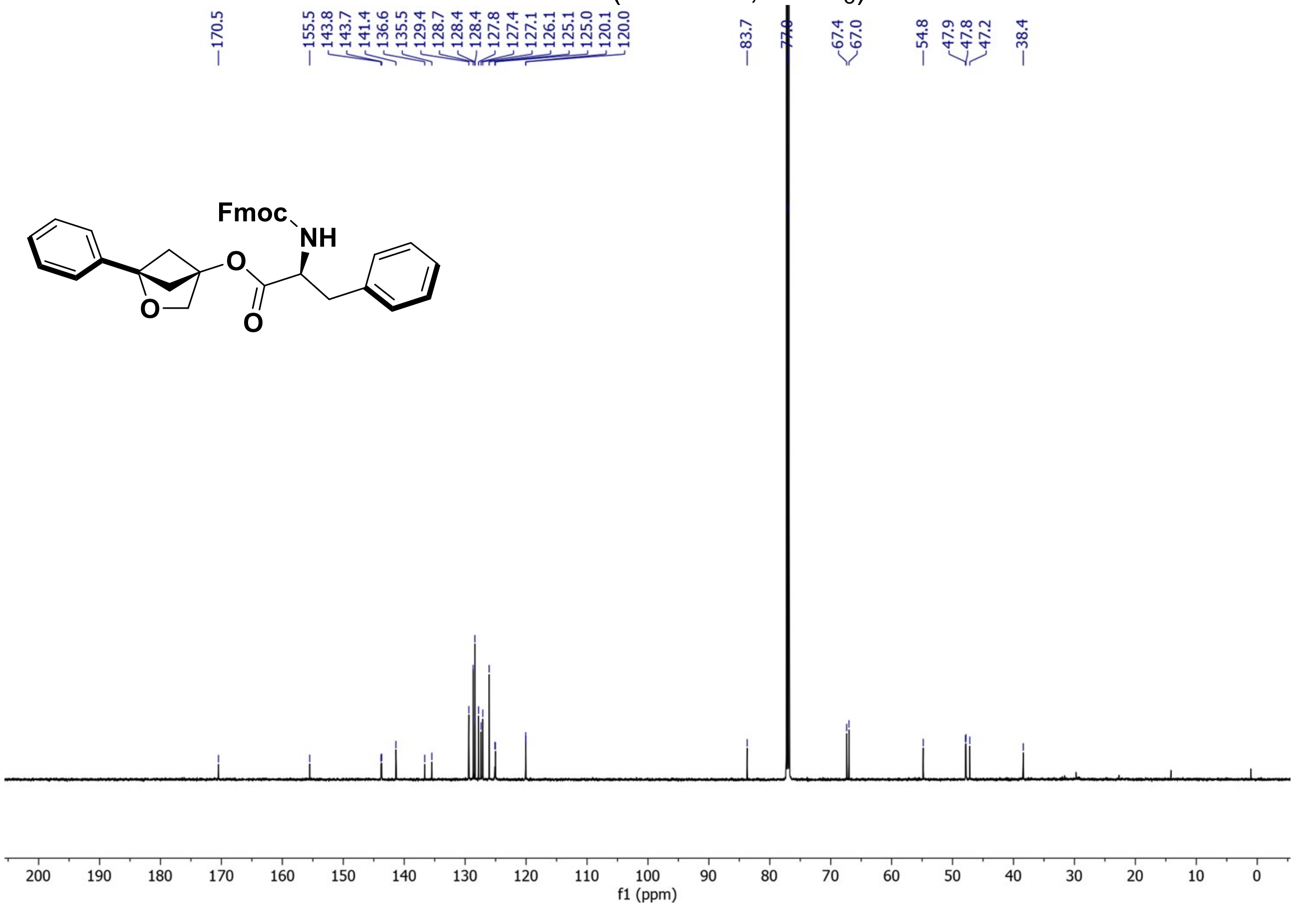

11 ^{1}H NMR (600 MHz, $CDCl_3$)

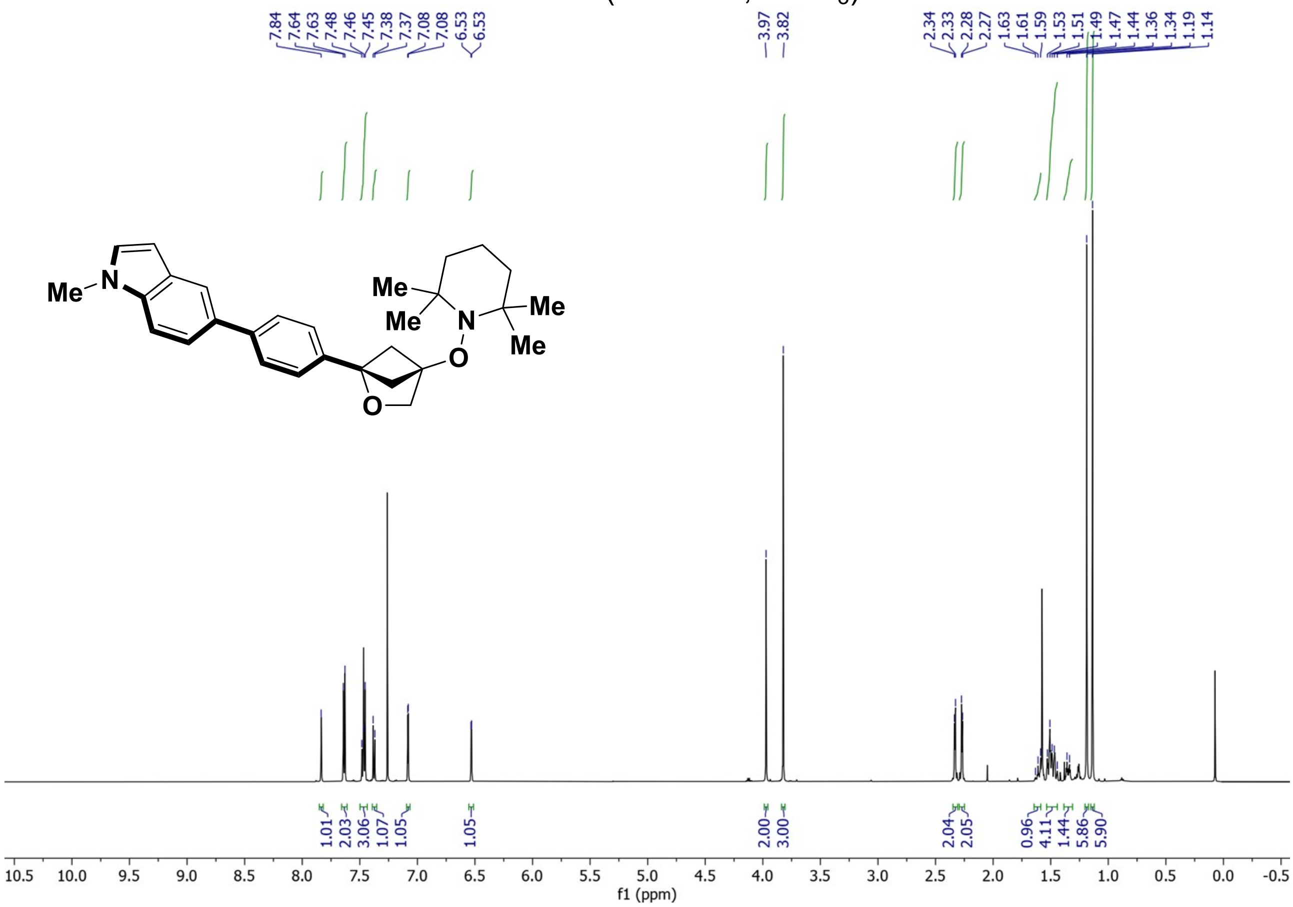

11 ^{13}C NMR (151 MHz, $CDCl_3$)

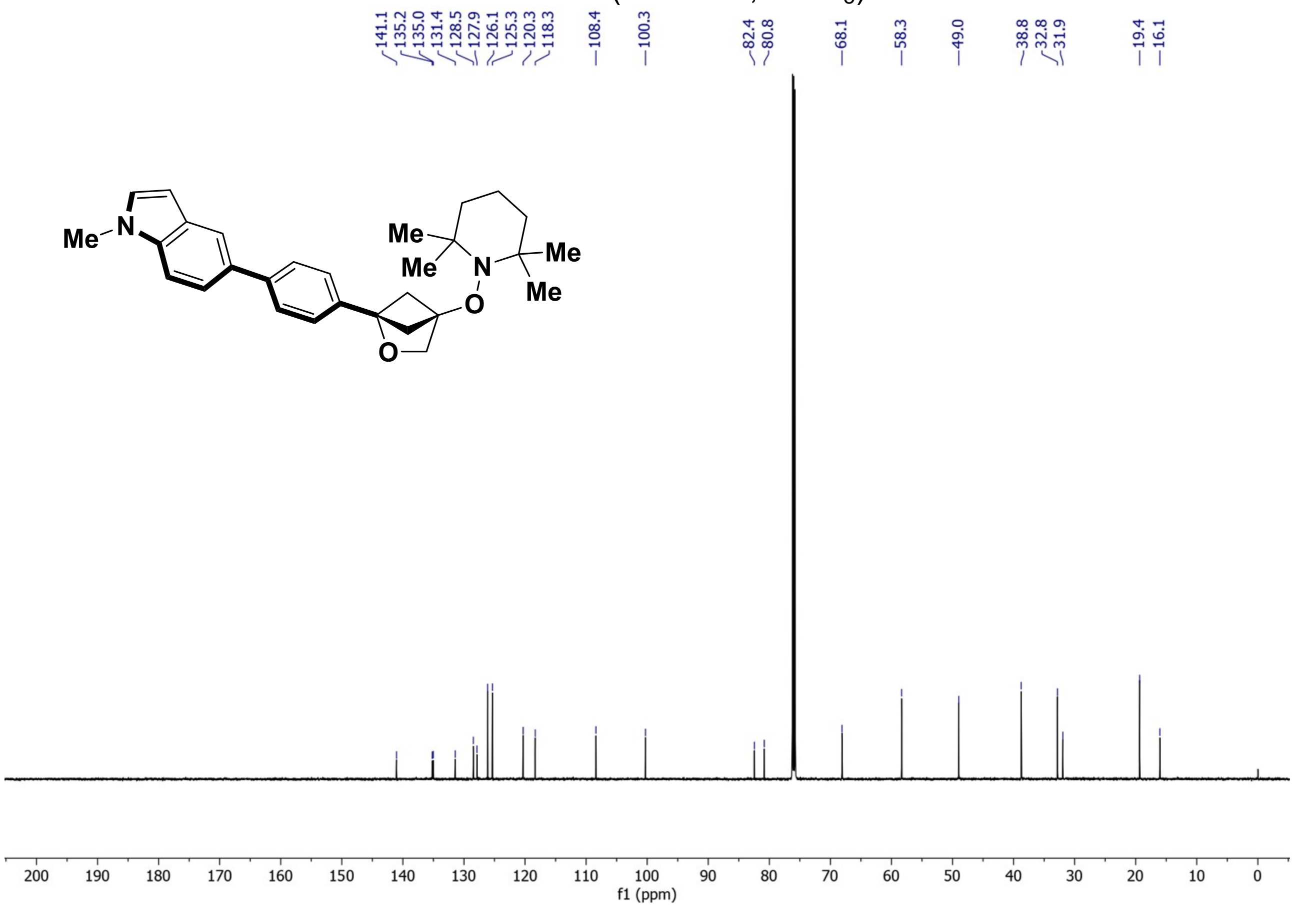

9. Cartesian Coordinates

Reaction with TEMPO 2a

FP$^+$

SCF Energy:

ZPE-corrected Energy: -984.699746

ΔU: -984.678465

ΔH: -984.677521

ΔG: -984.748562

Num. Imaginary Frequencies: 0

C	-2.174207	-0.012054	-0.039309
C	-1.208869	-1.042757	-0.606774
C	-0.147580	-0.018537	-0.120163
C	-1.196921	1.088524	-0.425215
C	-3.643885	0.003664	-0.115705
H	-1.217387	-2.048346	-0.192653
H	-1.276975	-1.058568	-1.691548
C	-0.245431	-0.145629	1.398563
O	1.105235	0.011286	-0.685692
H	-1.259977	1.293486	-1.491264
H	-1.188698	2.010923	0.152470
C	-4.319502	1.212517	-0.249262
C	-5.703226	1.226515	-0.321672
C	-6.411287	0.035495	-0.256626
C	-5.739353	-1.172941	-0.122539

C	-4.357617	-1.191088	-0.054230
H	-3.764317	2.140460	-0.304513
H	-6.227756	2.166405	-0.430727
H	-7.492096	0.047105	-0.314119
H	-6.294001	-2.100667	-0.075680
H	-3.831094	-2.131871	0.046253
H	0.104938	0.713728	1.957727
H	0.122779	-1.073179	1.817977
O	-1.735819	-0.201834	1.482781
H	-2.104228	0.496074	2.045496
N	2.148773	-0.018855	0.302868
C	2.931700	-1.272185	0.035116
C	3.747577	-1.163701	-1.261617
C	3.830563	-1.576539	1.240457
C	1.960954	-2.448487	-0.086492
C	4.587744	0.103787	-1.333259
H	4.377811	-2.051621	-1.350375
H	3.054564	-1.179427	-2.107838
C	3.707367	1.329724	-1.135359
H	5.383473	0.078786	-0.584441
H	5.085343	0.160991	-2.303015
C	2.888603	1.280069	0.163173
H	4.308162	2.242165	-1.132564
H	3.013804	1.408087	-1.977572
C	3.775303	1.493783	1.396240
C	1.876955	2.427465	0.153845
H	4.121179	-2.626554	1.199825

H	4.746257	-0.994342	1.260943
H	3.290331	-1.407552	2.172465
H	2.538278	-3.358895	-0.249700
H	1.386209	-2.586623	0.831071
H	1.277759	-2.333316	-0.924475
H	4.048196	2.548020	1.448451
H	3.232550	1.235790	2.306219
H	4.700200	0.926723	1.373275
H	2.421038	3.371333	0.113213
H	1.219204	2.386304	-0.710960
H	1.274384	2.435196	1.064236

FP+tau

SCF Energy:

ZPE-corrected Energy: -984.736175

ΔU: -984.715246

ΔH: -984.714301

ΔG: -984.785033

Num. Imaginary Frequencies: 0

C	2.118970	-0.013092	0.525191
C	1.110139	1.135399	0.286502
C	0.181851	0.077422	0.915077
C	0.988967	-1.001214	0.159675
C	3.476502	-0.044446	-0.080278
H	1.267852	2.067910	0.824619
H	0.946254	1.301220	-0.773972

C	0.786448	-0.040411	2.314708
O	-1.225448	0.160661	1.048647
H	0.810022	-1.023515	-0.913682
H	1.041621	-2.002034	0.580903
C	3.892373	-1.120755	-0.852685
C	5.165632	-1.136104	-1.405487
C	6.030666	-0.075347	-1.185534
C	5.620154	1.003146	-0.411941
C	4.348499	1.019016	0.136400
H	3.220706	-1.952520	-1.026048
H	5.480628	-1.978952	-2.007336
H	7.023655	-0.086527	-1.616599
H	6.292216	1.834055	-0.239334
H	4.027864	1.860884	0.738098
H	0.473691	-0.951170	2.828315
H	0.575926	0.831468	2.936034
O	2.170261	-0.099368	1.964799
N	-2.002134	0.012652	-0.108809
C	-2.739440	1.337816	-0.420255
C	-3.607236	1.077200	-1.654433
C	-3.541613	1.812898	0.783964
C	-1.675622	2.372410	-0.758602
C	-4.521769	-0.128489	-1.530315
H	-4.179546	1.988699	-1.829426
H	-2.954362	0.946645	-2.523171
C	-3.698169	-1.374207	-1.254937
H	-5.269122	0.025960	-0.750561

H	-5.072517	-0.259492	-2.461400
C	-2.837537	-1.287727	0.007598
H	-4.337499	-2.249352	-1.135250
H	-3.050009	-1.576686	-2.113006
C	-3.660456	-1.295681	1.289292
C	-1.852320	-2.448527	0.036362
H	-1.282180	-2.471947	0.962826
H	-2.427791	-3.371132	-0.024067
H	-1.171338	-2.432862	-0.815208
H	-2.185382	3.273132	-1.097518
H	-1.072992	2.634133	0.108483
H	-1.028187	2.040600	-1.570947
H	-3.788306	2.859872	0.612923
H	-4.474857	1.276739	0.918857
H	-2.957839	1.752370	1.700100
H	-3.067345	-0.980618	2.145279
H	-4.556160	-0.686559	1.230613
H	-3.975974	-2.323939	1.461598
H	-1.368820	-0.141126	-0.898840

Int1

SCF Energy:

ZPE-corrected Energy: -984.878566

ΔU: -984.856232

ΔH: -984.855287

ΔG: -984.929268

S^2 before (0.7919) and after higher multiplicity projection (0.7512)

Num. Imaginary Frequencies: 0

C	2.355129	-0.053317	-0.200993
C	1.310104	1.004382	-0.014009
C	0.316481	-0.145422	0.329798
C	1.347269	-1.162564	-0.227274
C	3.753423	-0.028614	-0.239016
H	1.464293	1.771441	0.747753
H	1.050718	1.493596	-0.957255
C	0.169528	-0.341105	1.833296
O	-0.886502	-0.151940	-0.429001
H	1.081078	-1.476655	-1.242027
H	1.536074	-2.055074	0.375852
C	4.504980	-1.220969	-0.382059
C	5.883292	-1.188425	-0.415551
C	6.566491	0.022070	-0.309764
C	5.846254	1.207388	-0.169693
C	4.467744	1.190367	-0.134549
H	3.983005	-2.166554	-0.465849
H	6.438554	-2.111953	-0.525281

H	7.648272	0.041573	-0.336796
H	6.372712	2.150466	-0.087756
H	3.916491	2.116549	-0.025851
H	-0.262796	-1.326172	2.026523
H	1.176618	-0.344017	2.255453
O	-0.559896	0.667786	2.489995
H	-1.426549	0.625542	2.054070
N	-2.106572	0.020756	0.297366
C	-2.730249	1.290959	-0.221982
C	-3.234002	1.128975	-1.663299
C	-3.861670	1.733770	0.715969
C	-1.683824	2.405971	-0.195122
C	-4.123359	-0.089986	-1.854030
H	-3.758732	2.043048	-1.951613
H	-2.365080	1.039203	-2.321055
C	-3.400571	-1.340373	-1.375605
H	-5.067808	0.035635	-1.318196
H	-4.384600	-0.195397	-2.908774
C	-2.904919	-1.238553	0.075195
H	-4.049297	-2.215756	-1.457023
H	-2.536666	-1.522692	-2.021243
C	-4.073070	-1.299287	1.069185
C	-2.035650	-2.460856	0.375949
H	-4.397456	-2.336550	1.158585
H	-3.755432	-0.958092	2.055211
H	-4.940422	-0.721930	0.765365
H	-2.609492	-3.355368	0.129918

H	-1.121865	-2.473708	-0.212632
H	-1.783063	-2.515580	1.435005
H	-2.171833	3.348930	-0.443405
H	-1.226984	2.506205	0.790159
H	-0.900520	2.233347	-0.926566
H	-4.094564	2.778178	0.507660
H	-4.781552	1.172812	0.590698
H	-3.556199	1.660447	1.760894

Int1$^+$

SCF Energy:

ZPE-corrected Energy: -984.705824

ΔU: -984.683467

ΔH: -984.682523

ΔG: -984.756008

Num. Imaginary Frequencies: 0

C	2.346926	-0.052839	-0.255056
C	1.309254	0.988012	-0.077229
C	0.318308	-0.161667	0.292188
C	1.354675	-1.152057	-0.307623
C	3.731985	-0.029825	-0.239938
H	1.491379	1.806573	0.616511
H	1.065795	1.398878	-1.062411
C	0.209015	-0.357693	1.801024
O	-0.879823	-0.171559	-0.447111
H	1.102608	-1.363524	-1.352432

H	1.580896	-2.087967	0.201993
C	4.461660	-1.234711	-0.385035
C	5.833285	-1.202272	-0.402513
C	6.490972	0.020747	-0.272821
C	5.791964	1.218753	-0.126936
C	4.419986	1.200768	-0.107933
H	3.930874	-2.172030	-0.484072
H	6.402765	-2.113890	-0.513796
H	7.573383	0.040508	-0.283918
H	6.330050	2.150637	-0.027919
H	3.858211	2.118136	0.005491
H	-0.242300	-1.331879	2.000627
H	1.222858	-0.387441	2.207108
O	-0.474963	0.672965	2.461725
H	-1.363839	0.638613	2.074429
N	-2.087556	0.016877	0.301855
C	-2.703612	1.294440	-0.212283
C	-3.245026	1.131823	-1.639496
C	-3.803540	1.756244	0.752914
C	-1.641021	2.394832	-0.218712
C	-4.154842	-0.075570	-1.802421
H	-3.763954	2.051863	-1.918227
H	-2.394707	1.027854	-2.319296
C	-3.434599	-1.333231	-1.340405
H	-5.083084	0.063374	-1.242549
H	-4.444642	-0.180339	-2.849493
C	-2.902444	-1.236453	0.097382

H	-4.094704	-2.201270	-1.403514
H	-2.589907	-1.526834	-2.008002
C	-4.044864	-1.280964	1.121358
C	-2.039441	-2.467939	0.377687
H	-4.380014	-2.313807	1.219329
H	-3.698348	-0.943844	2.098885
H	-4.911914	-0.692953	0.838380
H	-2.632871	-3.355221	0.154557
H	-1.146974	-2.501250	-0.242353
H	-1.754709	-2.523438	1.428342
H	-2.124350	3.344678	-0.447105
H	-1.148789	2.489680	0.749958
H	-0.890152	2.219387	-0.983755
H	-4.036437	2.798872	0.536893
H	-4.729460	1.199038	0.662081
H	-3.468317	1.695821	1.789449

TEMPO$^+$

SCF Energy:

ZPE-corrected Energy: -483.310621

ΔU: -483.298859

ΔH: -483.297915

ΔG: -483.346548

Num. Imaginary Frequencies: 0

O	0.000000	-1.931759	-0.359810
N	0.000000	-0.780474	-0.126714
C	1.342298	-0.077385	0.021235
C	1.242303	1.306127	-0.636304
C	1.634673	0.009503	1.528509
C	2.391756	-0.939404	-0.661296
C	0.000000	2.087618	-0.250831
H	2.149734	1.841388	-0.355329
H	1.268798	1.175660	-1.720542
C	-1.242303	1.306127	-0.636304
H	-0.000000	2.331521	0.812589
H	-0.000000	3.038763	-0.782371
C	-1.342298	-0.077385	0.021235
H	-2.149734	1.841388	-0.355329
H	-1.268798	1.175660	-1.720542
C	-1.634673	0.009503	1.528509
C	-2.391756	-0.939404	-0.661296
H	-3.325215	-0.378765	-0.641962
H	-2.131652	-1.136290	-1.700745

H	-2.547829	-1.883490	-0.143713
H	-2.678554	0.306850	1.616882
H	-1.513588	-0.958432	2.012705
H	-1.029567	0.750734	2.039957
H	3.325215	-0.378765	-0.641962
H	2.547829	-1.883490	-0.143713
H	2.131652	-1.136290	-1.700745
H	2.678554	0.306850	1.616882
H	1.029567	0.750734	2.039957
H	1.513588	-0.958432	2.012705

TEMPO 2a

SCF Energy:

ZPE-corrected Energy: -483.502341

ΔU: -483.490559

ΔH: -483.489615

ΔG: -483.538777

S^2 before (0.7548) and after higher multiplicity projection (0.7500)

Num. Imaginary Frequencies: 0

O	0.000000	-2.017559	-0.091952
N	0.000000	-0.751198	-0.198354
C	1.316156	-0.075908	-0.022414
C	1.235428	1.375890	-0.505054
C	1.726355	-0.148594	1.453840
C	2.342664	-0.825384	-0.869170
C	0.000000	2.110449	-0.015839
H	2.148633	1.882463	-0.186598
H	1.234097	1.380758	-1.599328
C	-1.235428	1.375890	-0.505054
H	0.000000	2.192101	1.073886
H	0.000000	3.131021	-0.401846
C	-1.316156	-0.075908	-0.022414
H	-2.148633	1.882463	-0.186598
H	-1.234097	1.380758	-1.599328
C	-1.726355	-0.148593	1.453840
C	-2.342664	-0.825384	-0.869170
H	-3.291050	-0.287206	-0.831871

H	-2.017629	-0.879829	-1.909246
H	-2.501233	-1.836137	-0.499021
H	-2.757457	0.191807	1.560006
H	-1.663196	-1.177573	1.808234
H	-1.098946	0.476208	2.088152
H	3.291050	-0.287206	-0.831871
H	2.501233	-1.836137	-0.499021
H	2.017629	-0.879829	-1.909246
H	2.757457	0.191807	1.560006
H	1.098946	0.476208	2.088152
H	1.663196	-1.177573	1.808234

TS1

SCF Energy:

ZPE-corrected Energy: -984.826829

ΔU: -984.803801

ΔH: -984.802857

ΔG: -984.878379

S^2 before (0.8413) and after higher multiplicity projection (0.7531)

Num. Imaginary Frequencies: 1

Imaginary Frequency: -913.0831

C	2.007502	0.767324	-0.560622
C	3.400886	0.434323	-0.491142
C	0.880344	-0.170314	-0.859159
C	1.238657	1.628174	0.395888
H	0.072611	0.209126	-1.497946
H	1.111175	-1.209167	-1.069041
C	0.705519	0.230423	0.568662
H	0.549107	2.333606	-0.059337
H	1.765116	2.067616	1.241238
C	1.206870	-0.567991	1.732195
H	0.485431	-0.439322	2.546854
O	1.340173	-1.930423	1.383206
H	2.169375	-0.160901	2.068787
O	-1.207455	0.156483	1.034819
N	-2.091078	0.079762	0.061904
C	4.303013	1.219262	0.248933
C	5.646313	0.893650	0.311928

C	6.136216	-0.216455	-0.364912
C	5.260948	-0.996990	-1.112869
C	3.917703	-0.679163	-1.178991
H	3.944638	2.097163	0.770958
H	6.318890	1.514177	0.891404
H	7.188129	-0.466850	-0.316826
H	5.632248	-1.860486	-1.651130
H	3.253433	-1.293212	-1.773535
H	1.577520	-2.420558	2.172121
C	-2.945597	1.295198	-0.045026
C	-3.894941	1.153450	-1.239182
C	-3.729169	1.556339	1.250918
C	-2.042755	2.498729	-0.298285
C	-4.655868	-0.159114	-1.253445
H	-4.578298	2.005538	-1.226131
H	-3.307289	1.229164	-2.159852
C	-3.657287	-1.300930	-1.265361
H	-5.320942	-0.231913	-0.389344
H	-5.293012	-0.211810	-2.138358
C	-2.681201	-1.283611	-0.083675
H	-4.163775	-2.268811	-1.265674
H	-3.077238	-1.244983	-2.192008
C	-3.374787	-1.731233	1.211731
C	-1.558572	-2.270119	-0.391496
H	-1.985639	-3.267981	-0.509051
H	-1.060904	-2.003661	-1.323941
H	-0.816165	-2.298936	0.403321

H	-2.660003	3.373954	-0.508341
H	-1.428079	2.715569	0.573109
H	-1.395079	2.319501	-1.157670
H	-3.618878	-2.792944	1.147099
H	-2.706662	-1.582564	2.060105
H	-4.299760	-1.190186	1.401590
H	-4.155022	2.560907	1.222427
H	-4.548052	0.854754	1.396469
H	-3.059828	1.490689	2.108631

TS1^{+}

TS1+

SCF Energy:

ZPE-corrected Energy: -984.667632

ΔU: -984.667632

ΔH: -984.644041

ΔG: -984.718037

Num. Imaginary Frequencies: 1

Imaginary Frequency: -383.3537

C	-2.192909	0.216198	-1.206768
C	-3.448865	0.143606	-0.494731
C	-1.231769	1.324634	-1.211098
C	-1.256468	-0.886907	-1.459632
H	-0.729372	1.533197	-2.156494
H	-1.447407	2.213439	-0.630020
C	-0.599810	0.117803	-0.522469
H	-0.751429	-0.889482	-2.426649
H	-1.494346	-1.875420	-1.087743
C	-0.544269	-0.041641	0.962540
H	-1.010626	0.816402	1.454050
O	-1.056732	-1.259390	1.443654
H	0.522141	-0.026371	1.243431
O	1.193855	0.175295	-1.182884

N	2.110418	0.074186	-0.341225
C	-4.095983	-1.088514	-0.329629
C	-5.297807	-1.159360	0.348239
C	-5.875055	-0.004888	0.861082
C	-5.252792	1.224742	0.687225
C	-4.050316	1.302526	0.011810
H	-3.661625	-1.989255	-0.741604
H	-5.789767	-2.114636	0.472345
H	-6.817175	-0.062424	1.390515
H	-5.709692	2.124389	1.076999
H	-3.580096	2.265919	-0.129630
H	-2.012335	-1.192328	1.518126
C	2.753899	1.348985	0.128127
C	3.399689	1.111201	1.495907
C	3.783525	1.792750	-0.920776
C	1.690260	2.435508	0.246615
C	4.231494	-0.158266	1.568678
H	4.004944	1.988326	1.729932
H	2.608681	1.060766	2.250232
C	3.369539	-1.361595	1.225156
H	5.094271	-0.095957	0.902062
H	4.630916	-0.272794	2.576844
C	2.729344	-1.283071	-0.163018
H	3.952124	-2.283229	1.266401
H	2.573489	-1.454706	1.970155
C	3.759507	-1.502366	-1.280345
C	1.646116	-2.348366	-0.290912
H	2.111728	-3.317050	-0.110137
H	0.851458	-2.219451	0.441831
H	1.213697	-2.363425	-1.289900
H	2.176128	3.338630	0.614717
H	1.235837	2.663731	-0.715550
H	0.912661	2.170853	0.961021
H	4.067477	-2.548133	-1.265039
H	3.316002	-1.289239	-2.253237
H	4.650548	-0.891692	-1.160650
H	4.116074	2.801368	-0.673996
H	4.660416	1.151339	-0.948930
H	3.331394	1.812813	-1.912673

TS2⁺

SCF Energy:

ZPE-corrected Energy: -984.699259

ΔU: -984.677937

ΔH: -984.676993

ΔG: -984.748226

Num. Imaginary Frequencies: 1

Imaginary Frequency: -240.8264

C	-2.209560	-0.000298	-0.215851
C	-1.205484	-1.066075	-0.524930
C	-0.162919	-0.014980	-0.039823
C	-1.196068	1.073417	-0.461320
C	-3.642496	0.003473	-0.203222
H	-1.284627	-2.035902	-0.041304
H	-1.142014	-1.178660	-1.608538
C	-0.249569	-0.060880	1.483853
O	1.083560	-0.011420	-0.631148
H	-1.130356	1.247828	-1.536558
H	-1.264092	2.018423	0.071742
C	-4.338261	1.216951	-0.212623
C	-5.719241	1.216158	-0.227318
C	-6.407908	0.009141	-0.228701
C	-5.723517	-1.200923	-0.218492
C	-4.343102	-1.207331	-0.205551
H	-3.792845	2.151964	-0.213601
H	-6.261983	2.151446	-0.238999

H	-7.490286	0.011089	-0.240022
H	-6.270273	-2.133960	-0.222275
H	-3.800791	-2.143893	-0.199139
H	0.156959	0.823524	1.963843
H	0.190601	-0.954218	1.913611
O	-1.683882	-0.130201	1.663392
H	-2.008730	0.625677	2.166171
N	2.157308	-0.004207	0.321422
C	2.925392	-1.271535	0.082050
C	3.700008	-1.224186	-1.243680
C	3.861452	-1.530716	1.269603
C	1.945752	-2.446242	0.041156
C	4.541637	0.034942	-1.398006
H	4.323975	-2.118249	-1.314158
H	2.979943	-1.273050	-2.065755
C	3.672034	1.272452	-1.225574
H	5.360953	0.038921	-0.674598
H	5.008082	0.047331	-2.384778
C	2.896548	1.282723	0.100265
H	4.275401	2.181442	-1.283340
H	2.950938	1.316449	-2.046993
C	3.825349	1.545774	1.292360
C	1.889304	2.433892	0.075420
H	4.151877	-2.581598	1.260357
H	4.776816	-0.948261	1.240175
H	3.349777	-1.325806	2.210498
H	2.513694	-3.367963	-0.088422

H	1.388152	-2.532420	0.975336
H	1.246096	-2.368318	-0.787547
H	4.103292	2.600068	1.290670
H	3.312751	1.328636	2.230067
H	4.747291	0.974066	1.262636
H	2.434796	3.373171	-0.019032
H	1.206202	2.361454	-0.767448
H	1.313716	2.478672	1.001800

Reaction with Thiols (PhSH)

BCB

SCF Energy:

ZPE-corrected Energy: -501.360674

ΔU: -501.349977

ΔH: -501.349033

ΔG: -501.398161

Num. Imaginary Frequencies: 0

C	2.067934	-0.309145	-0.052742
C	2.341391	0.613689	1.079324
C	1.699545	-1.750651	-0.011758
C	1.800761	-0.048013	-1.492646
H	2.231591	-2.459937	-0.643768
H	1.409221	-2.143982	0.957540
C	0.778698	-0.754812	-0.648323
H	2.338710	-0.611348	-2.254087
H	1.585786	0.981647	-1.756026
C	-0.606767	-0.397012	-0.335936
O	1.801302	1.889085	0.782138
C	-1.299379	-1.029648	0.698046
C	-2.599425	-0.663361	1.010180
C	-3.239917	0.335226	0.290158
C	-2.566825	0.959108	-0.750926
C	-1.266824	0.594082	-1.063900
H	-0.820325	-1.819829	1.262128

H	-3.116309	-1.165875	1.818462
H	-4.256623	0.617691	0.531741
H	-3.058345	1.732551	-1.328191
H	-0.757247	1.081565	-1.885397
H	3.421712	0.685240	1.251062
H	1.888135	0.195300	1.987301
H	2.068127	2.497505	1.473266

Int1

SCF Energy:

ZPE-corrected Energy: -1131.13572

ΔU: -1131.11848

ΔH: -1131.11754

ΔG: -1131.18365

S^2 before (0.7911) and after higher multiplicity projection (0.7511)

Num. Imaginary Frequencies: 0

C	-1.694721	0.201109	0.075214
C	-1.020126	1.448601	0.560920
C	0.318745	0.860002	0.025870
C	-0.413581	-0.469434	-0.320417
C	-3.038435	-0.185393	-0.009456
H	-1.320013	2.389409	0.092152
H	-1.052119	1.588485	1.646301
C	0.821781	1.551089	-1.224039
S	1.549929	0.765983	1.364565
H	-0.124712	-1.313630	0.310864
H	-0.328901	-0.785692	-1.364975
C	-3.404150	-1.445577	-0.540339
C	-4.729257	-1.819591	-0.622391
C	-5.735538	-0.961343	-0.182545
C	-5.396306	0.283744	0.344097
C	-4.075380	0.670973	0.432138
H	-2.628264	-2.119087	-0.884183
H	-4.988693	-2.788168	-1.031919

H	-6.773674	-1.259984	-0.249184
H	-6.175349	0.953327	0.687443
H	-3.820693	1.640461	0.843295
C	2.835286	-0.194264	0.603671
C	3.839494	0.433452	-0.129311
C	4.845746	-0.317036	-0.717946
C	4.859898	-1.697497	-0.573244
C	3.866091	-2.326508	0.162631
C	2.857026	-1.578176	0.751397
H	3.820844	1.510020	-0.241635
H	5.621340	0.177954	-1.288401
H	5.647492	-2.282282	-1.031194
H	3.875943	-3.402417	0.281978
H	2.085035	-2.066843	1.331807
H	1.643471	0.969114	-1.657406
H	-0.000345	1.555549	-1.951095
O	1.229195	2.868438	-0.924990
H	1.466775	3.305642	-1.744467

Int1$^+$

SCF Energy:

ZPE-corrected Energy: -1130.95998

ΔU: -1130.94278

ΔH: -1130.94183

ΔG: -1131.00740

Num. Imaginary Frequencies: 0

C	-1.701503	0.130423	0.209146
C	-1.030530	1.371591	0.669518
C	0.303574	0.799051	0.111850
C	-0.421095	-0.566377	-0.068819
C	-3.029495	-0.216783	0.031433
H	-1.389384	2.315898	0.259929
H	-1.059347	1.437530	1.762407
C	0.673556	1.402552	-1.230499
S	1.623787	0.861657	1.356398
H	-0.193432	-1.273107	0.734934
H	-0.332208	-1.089662	-1.021482
C	-3.367329	-1.497697	-0.470480
C	-4.686383	-1.844518	-0.617232
C	-5.678375	-0.926650	-0.271767
C	-5.368012	0.340686	0.221511
C	-4.053067	0.700854	0.373303
H	-2.581739	-2.193503	-0.732989
H	-4.959031	-2.818443	-0.997856
H	-6.717780	-1.204988	-0.391275
H	-6.159442	1.029843	0.479131
H	-3.789532	1.678865	0.753397
C	2.883653	-0.112154	0.571117
C	3.812604	0.493694	-0.270730
C	4.799181	-0.268427	-0.876885
C	4.867292	-1.634447	-0.640099
C	3.948872	-2.238952	0.205920
C	2.959242	-1.480414	0.813505

H	3.752193	1.559099	-0.452598
H	5.517308	0.206363	-1.532940
H	5.640160	-2.227490	-1.112045
H	4.003484	-3.302766	0.397880
H	2.249304	-1.948227	1.483369
H	1.502299	0.833802	-1.665725
H	-0.186863	1.296592	-1.904406
O	1.006347	2.757205	-1.052193
H	1.199524	3.141451	-1.909304

Int2⁺

SCF Energy:

ZPE-corrected Energy: -1130.95618

ΔU: -1130.94051

ΔH: -1130.93957

ΔG: -1131.00101

Num. Imaginary Frequencies: 0

C	-1.481323	0.321773	0.026297
C	-0.906491	1.611203	0.603387
C	0.444695	0.936814	0.247078
C	-0.253873	-0.418553	0.545428
C	-2.883176	-0.126356	0.011738
H	-1.164202	2.555947	0.130808
H	-1.075017	1.645250	1.676583
C	0.418889	0.982444	-1.273143
S	1.948812	1.488078	1.036077
H	-0.344465	-0.598064	1.613612
H	0.084802	-1.312823	0.026308
C	-3.178734	-1.480843	0.127899
C	-4.498349	-1.902794	0.114749
C	-5.520144	-0.973564	-0.015301
C	-5.227306	0.379516	-0.129845
C	-3.910901	0.804976	-0.115188
H	-2.378882	-2.202833	0.235329
H	-4.728648	-2.955644	0.208664
H	-6.550914	-1.303753	-0.023512

H	-6.026560	1.102192	-0.226702
H	-3.679668	1.859313	-0.200426
C	3.080705	0.262102	0.413768
C	3.903028	0.560344	-0.667368
C	4.786064	-0.395597	-1.147359
C	4.847007	-1.647296	-0.552326
C	4.029443	-1.943347	0.529567
C	3.150865	-0.989065	1.018605
H	3.848622	1.536804	-1.131310
H	5.425120	-0.160815	-1.988657
H	5.535540	-2.392428	-0.929547
H	4.079472	-2.917207	0.999087
H	2.520776	-1.212032	1.870053
H	1.060295	0.254317	-1.761455
H	0.528921	1.973819	-1.700170
O	-1.002672	0.580230	-1.472786
H	-1.097212	-0.213672	-2.021520

PhS^{+}

SCF Energy:

ZPE-corrected Energy: -629.486612

ΔU: -629.480855

ΔH: -629.479911

ΔG: -629.516104

Num. Imaginary Frequencies: 0

S	0.000000	-0.000000	2.222755
C	0.000000	-0.000000	0.599464
C	-0.000000	1.251396	-0.138686
C	-0.000000	1.235764	-1.496768
C	-0.000000	0.000000	-2.167360
C	-0.000000	-1.235764	-1.496768
C	-0.000000	-1.251396	-0.138686
H	-0.000000	2.174225	0.425004
H	-0.000000	2.154054	-2.065332
H	-0.000000	0.000000	-3.250597
H	-0.000000	-2.154054	-2.065332
H	-0.000000	-2.174225	0.425004

PhS-

SCF Energy:

ZPE-corrected Energy: -629.891587

ΔU: -629.886136

ΔH: -629.885192

ΔG: -629.920653

Num. Imaginary Frequencies: 0

S	0.000000	0.000000	2.334939
C	0.000000	0.000000	0.578840
C	-0.000000	1.193918	-0.161954
C	-0.000000	1.194026	-1.548443
C	-0.000000	0.000000	-2.258501
C	-0.000000	-1.194026	-1.548443
C	-0.000000	-1.193918	-0.161954
H	-0.000000	2.137727	0.371212
H	-0.000000	2.139777	-2.078795
H	-0.000000	0.000000	-3.341124
H	-0.000000	-2.139777	-2.078795
H	-0.000000	-2.137727	0.371212

PhS

SCF Energy:

ZPE-corrected Energy: -629.725727

ΔU: -629.720229

ΔH: -629.719285

ΔG: -629.755488

S^2 before (0.7794) and after higher multiplicity projection (0.7506)

Num. Imaginary Frequencies: 0

S	0.000000	-0.000000	2.282968
C	0.000000	-0.000000	0.575553
C	0.000000	1.213920	-0.146072

C	0.000000	1.208473	-1.524629
C	-0.000000	0.000000	-2.216735
C	-0.000000	-1.208473	-1.524629
C	-0.000000	-1.213920	-0.146072
H	0.000000	2.146716	0.402413
H	0.000000	2.143740	-2.068850
H	-0.000000	0.000000	-3.299092
H	-0.000000	-2.143740	-2.068850
H	-0.000000	-2.146716	0.402413

TS1

SCF Energy:

ZPE-corrected Energy: -1131.08482

ΔU: -1131.06735

ΔH: -1131.06641

ΔG: -1131.13379

S^2 before (0.7858) and after higher multiplicity projection (0.7510)

Num. Imaginary Frequencies: 1

Imaginary Frequency: -386.2439

C	1.011239	-0.621422	-0.005002
C	2.460302	-0.720950	0.025546
C	0.136710	-0.000137	1.017555
C	0.150483	-0.394750	-1.193053
H	-0.813237	-0.492104	1.227514
H	0.600169	0.448311	1.888032
C	0.169604	0.786291	-0.265464
H	-0.793776	-0.937108	-1.234671
H	0.625004	-0.280605	-2.162380
C	0.996527	2.014855	-0.518982
H	0.406698	2.737238	-1.091203
O	1.400150	2.559264	0.719765
H	1.867887	1.744353	-1.131245
S	-2.158465	1.859109	-0.411473
C	-3.063799	0.411497	-0.109525
C	3.193245	-0.935118	-1.146875
C	4.575432	-1.017265	-1.112654

C	5.254660	-0.896021	0.091254
C	4.538064	-0.697919	1.264786
C	3.157706	-0.613959	1.234547
H	2.679694	-1.049147	-2.092320
H	5.124072	-1.181966	-2.031319
H	6.334623	-0.963836	0.117113
H	5.058759	-0.611990	2.210086
H	2.612334	-0.470445	2.157953
H	1.948680	3.326426	0.546977
C	-3.421991	0.045583	1.197203
C	-4.125222	-1.122039	1.437227
C	-4.484815	-1.951106	0.381847
C	-4.139017	-1.603625	-0.918213
C	-3.436574	-0.436686	-1.163762
H	-3.139535	0.691175	2.019002
H	-4.393128	-1.388271	2.451888
H	-5.033755	-2.864531	0.571821
H	-4.418111	-2.245920	-1.743917
H	-3.167472	-0.164821	-2.176498

TS1$^+$

SCF Energy:

ZPE-corrected Energy: -1130.84965

ΔU: -1130.83234

ΔH: -1130.83140

ΔG: -1130.89669

Num. Imaginary Frequencies: 1

Imaginary Frequency: -110.4806

C	-1.564742	-1.311488	-0.385575
C	-0.170249	-1.727072	-0.250156
C	-2.771109	-1.466402	0.498493
C	-2.311353	-0.821902	-1.588947
H	-3.541173	-2.171706	0.183293
H	-2.617965	-1.417835	1.571819
C	-2.608097	-0.197452	-0.269009
H	-3.050874	-1.493588	-2.026190
H	-1.771006	-0.213289	-2.307572
C	-2.884069	1.245356	0.008375
H	-2.886487	1.451725	1.076581
O	-4.178284	1.582020	-0.456630
H	-2.125511	1.875625	-0.467389
S	-0.269684	0.972024	1.771791
C	0.912135	1.486058	0.768166
C	0.740458	-1.515396	-1.287582
C	2.028021	-2.031836	-1.215951
C	2.429742	-2.754709	-0.105411
C	1.535189	-2.954845	0.941112
C	0.248576	-2.450575	0.868535
H	0.433804	-0.970275	-2.170620
H	2.715430	-1.865031	-2.035154
H	3.430803	-3.161922	-0.051348
H	1.839616	-3.519254	1.813125
H	-0.447218	-2.634063	1.677018

H	-4.195115	1.481376	-1.411720
C	0.815142	2.765348	0.105087
C	1.829284	3.191736	-0.692185
C	2.970511	2.382127	-0.852534
C	3.102782	1.138700	-0.232490
C	2.087417	0.674664	0.555223
H	-0.070218	3.363565	0.271189
H	1.775848	4.146345	-1.195201
H	3.775511	2.740100	-1.482233
H	3.993904	0.548093	-0.387497
H	2.144029	-0.282280	1.055380

TS2⁺

SCF Energy:

ZPE-corrected Energy: -1130.95511

ΔU: -1130.93955

ΔH: -1130.93861

ΔG: -1130.99973

Num. Imaginary Frequencies: 1

Imaginary Frequency: -240.1828

C	-1.535135	-0.303213	-0.215520
C	-0.911909	-1.624623	-0.556009
C	0.425763	-0.923014	-0.188341
C	-0.270376	0.413021	-0.582345
C	-2.896122	0.127048	-0.104987
H	-1.219992	-2.518191	-0.020443
H	-0.991993	-1.776252	-1.633412
C	0.434567	-0.913664	1.335191
S	1.920672	-1.501676	-0.986015
H	-0.251490	0.550041	-1.664160
H	-0.003851	1.339085	-0.079482
C	-3.195288	1.493325	-0.115537
C	-4.509933	1.907483	-0.032219
C	-5.524898	0.963179	0.065344
C	-5.234620	-0.396558	0.077383
C	-3.922973	-0.817897	-0.006856
H	-2.396890	2.219941	-0.194493
H	-4.748190	2.962221	-0.044030

H	-6.554649	1.290279	0.130682
H	-6.033496	-1.121627	0.151482
H	-3.686394	-1.873897	0.001538
C	3.073279	-0.274817	-0.406886
C	3.892090	-0.548654	0.683582
C	4.792606	0.407525	1.128988
C	4.875925	1.635982	0.489609
C	4.062707	1.907797	-0.601710
C	3.166002	0.952730	-1.055501
H	3.820722	-1.506843	1.181931
H	5.428288	0.191244	1.977801
H	5.578569	2.381305	0.839461
H	4.130140	2.862932	-1.106257
H	2.539964	1.156956	-1.914577
H	1.126928	-0.182452	1.746889
H	0.624086	-1.898493	1.756001
O	-0.930803	-0.545902	1.630347
H	-0.976188	0.283890	2.119417

Reaction with TBABr 2f

Br^+

SCF Energy:

ZPE-corrected Energy: -2573.78467

ΔU: -2573.78326

ΔH: -2573.78231

ΔG: -2573.80085

Num. Imaginary Frequencies: 0

Br	0.000000	0.000000	0.000000

Br-

SCF Energy:

ZPE-corrected Energy: -2574.39930

ΔU: -2574.39789

ΔH: -2574.39694

ΔG: -2574.41548

Num. Imaginary Frequencies: 0

Br	0.000000	0.000000	0.000000

Br

SCF Energy:

ZPE-corrected Energy: -2574.17288

ΔU: -2574.17147

ΔH: -2574.17052

ΔG: -2574.18971

S^2 before (0.7526) and after higher multiplicity projection (0.7500)

Num. Imaginary Frequencies: 0

Br	0.000000	0.000000	0.000000

Int1

SCF Energy:

ZPE-corrected Energy: -3075.59486

ΔU: -3075.58237

ΔH: -3075.58143

ΔG: -3075.63622

S^2 before (0.7914) and after higher multiplicity projection (0.7511)

Num. Imaginary Frequencies: 0

C	0.723687	-0.108200	-0.016526
C	-0.328414	0.597261	-0.821411
C	-1.339646	0.150920	0.263062
C	-0.272475	-0.784636	0.879699
C	2.123549	-0.084484	-0.042283
H	-0.236248	1.678158	-0.948404
H	-0.508364	0.147899	-1.801236
C	-1.754546	1.255052	1.206973
Br	-2.935001	-0.768162	-0.424205
H	-0.433259	-1.839612	0.644100
H	-0.134173	-0.691039	1.960405
C	2.885003	-0.838808	0.882130
C	4.263572	-0.810043	0.849775
C	4.933312	-0.036138	-0.096174
C	4.201613	0.713166	-1.015689
C	2.822728	0.693970	-0.995501
H	2.372655	-1.444160	1.620126
H	4.828848	-1.394342	1.565325

H	6.015218	-0.017696	-0.116997
H	4.718611	1.315241	-1.752692
H	2.261724	1.278572	-1.714573
H	-2.339161	0.831778	2.030589
H	-0.831663	1.675905	1.627684
O	-2.479224	2.239150	0.506528
H	-2.705671	2.943401	1.116306

Int1^{+}

SCF Energy:

ZPE-corrected Energy: -3075.41640

ΔU: -3075.40398

ΔH: -3075.40304

ΔG: -3075.45696

Num. Imaginary Frequencies: 0

C	0.731663	-0.116217	-0.019433
C	-0.316944	0.589412	-0.801054
C	-1.339862	0.134665	0.269430
C	-0.259980	-0.817535	0.838184
C	2.112519	-0.080365	-0.037369
H	-0.197722	1.660891	-0.961145
H	-0.454001	0.105433	-1.772048
C	-1.720201	1.233353	1.237728
Br	-2.927263	-0.746699	-0.430476
H	-0.382804	-1.849708	0.497981
H	-0.088893	-0.826752	1.914785

C	2.856192	-0.870561	0.875013
C	4.226603	-0.845082	0.835134
C	4.869900	-0.037011	-0.102999
C	4.158029	0.749283	-1.009436
C	2.787218	0.733520	-0.982013
H	2.336764	-1.489578	1.594093
H	4.807275	-1.442485	1.523215
H	5.952095	-0.018812	-0.127346
H	4.686130	1.364590	-1.723608
H	2.214698	1.335235	-1.675062
H	-2.320297	0.810594	2.049289
H	-0.791220	1.625192	1.674372
O	-2.408764	2.236996	0.537280
H	-2.668545	2.923136	1.154802

TS2+

SCF Energy:

ZPE-corrected Energy: -3075.40980

ΔU: -3075.39911

ΔH: -3075.39817

ΔG: -3075.44764

Num. Imaginary Frequencies: 1

Imaginary Frequency: -245.1506

C	0.534422	-0.011730	-0.075910
C	-0.490049	1.000462	-0.510991
C	-1.490710	0.024191	0.150561
C	-0.489828	-1.110467	-0.165739
C	1.966689	-0.021294	-0.137463
H	-0.422312	2.024517	-0.155519
H	-0.566949	0.970650	-1.597643
C	-1.347549	0.273780	1.644393
Br	-3.308217	-0.071330	-0.435224
H	-0.566421	-1.424247	-1.207031
H	-0.426236	-1.975922	0.488623
C	2.659574	-1.230800	-0.025326
C	4.037984	-1.240502	-0.109522
C	4.725290	-0.048042	-0.300780
C	4.042493	1.157939	-0.411321
C	2.664666	1.175366	-0.329915
H	2.115146	-2.154801	0.121390
H	4.579436	-2.172984	-0.028248

H	5.805629	-0.058420	-0.366660
H	4.588272	2.079217	-0.562198
H	2.124236	2.109079	-0.415077
H	-1.732216	-0.544108	2.248451
H	-1.776023	1.222585	1.957357
O	0.092376	0.364739	1.754487
H	0.448122	-0.310355	2.345597

Reaction with TBAI 2e

Int1

SCF Energy:

ZPE-corrected Energy: -799.162157

ΔU: -799.149556

ΔH: -799.148611

ΔG: -799.204154

S^2 before (0.7913) and after higher multiplicity projection (0.7511)

Num. Imaginary Frequencies: 0

C	1.168946	-0.035207	0.028341
C	0.132576	0.595606	-0.854331
C	-0.870947	0.378574	0.309027
C	0.167835	-0.509008	1.041004
C	2.567182	-0.093147	-0.028812
H	0.277184	1.638636	-1.146054
H	-0.091390	0.016213	-1.753498
C	-1.178581	1.640441	1.082781
I	-2.709141	-0.624001	-0.234797
H	-0.044939	-1.579432	0.984344
H	0.344208	-0.251786	2.089468
C	3.314199	-0.734267	0.988170
C	4.690968	-0.787334	0.925051
C	5.373078	-0.209857	-0.144316
C	4.655617	0.425087	-1.156466

C	3.278651	0.486016	-1.106616
H	2.792069	-1.186933	1.822461
H	5.245025	-1.282392	1.713019
H	6.453488	-0.254966	-0.188914
H	5.182278	0.874197	-1.989461
H	2.728728	0.980672	-1.898089
H	-1.737297	1.390913	1.991127
H	-0.213255	2.067687	1.387927
O	-1.887593	2.546350	0.269611
H	-2.048037	3.347028	0.772024

Int1⁺

SCF Energy:

ZPE-corrected Energy: -798.984061

ΔU: -798.971546

ΔH: -798.970602

ΔG: -799.025121

Num. Imaginary Frequencies: 0

C	1.178715	-0.070706	0.014632
C	0.144576	0.572941	-0.835926
C	-0.866119	0.335652	0.317156
C	0.177291	-0.602071	0.976630
C	2.559763	-0.100102	-0.030825
H	0.313972	1.600722	-1.156682
H	-0.041380	-0.040155	-1.722455
C	-1.107638	1.581664	1.144198

I	-2.720681	-0.584884	-0.245520
H	-0.005191	-1.661094	0.772442
H	0.379313	-0.487743	2.041813
C	3.289192	-0.796229	0.965406
C	4.658040	-0.842367	0.897424
C	5.313842	-0.197924	-0.152021
C	4.616092	0.495554	-1.141341
C	3.246935	0.549707	-1.086778
H	2.760424	-1.289408	1.769889
H	5.227708	-1.370773	1.648319
H	6.394768	-0.235918	-0.199049
H	5.153757	0.985232	-1.940509
H	2.684835	1.082614	-1.841989
H	-1.676141	1.321909	2.042652
H	-0.127089	1.960363	1.467142
O	-1.773280	2.533072	0.354152
H	-1.946788	3.311802	0.886407

I^+

SCF Energy:

ZPE-corrected Energy: -297.426678

ΔU: -297.425262

ΔH: -297.424317

ΔG: -297.443526

Num. Imaginary Frequencies: 0

I	0.000000	0.000000	0.000000

I^-

SCF Energy:

ZPE-corrected Energy: -297.972281

ΔU: -297.970865

ΔH: -297.969921

ΔG: -297.989129

Num. Imaginary Frequencies: 0

I	0.000000	0.000000	0.000000

I

SCF Energy:

ZPE-corrected Energy: -297.761123

ΔU: -297.759707

ΔH: -297.758763

ΔG: -297.778626

S^2 before (0.7525) and after higher multiplicity projection (0.7500)

Num. Imaginary Frequencies: 0

I	0.000000	0.000000	0.000000

TS2$^+$

SCF Energy:

ZPE-corrected Energy: -798.978829

ΔU: -798.967945

ΔH: -798.967001

ΔG: -799.017442

Num. Imaginary Frequencies: 1

Imaginary Frequency: -259.1589

C	1.037266	-0.013164	-0.039891
C	0.004319	0.895300	-0.644568
C	-0.984449	0.100031	0.246010
C	0.004733	-1.088299	0.154887
C	2.466119	-0.053148	-0.137762
H	0.094805	1.971036	-0.522925
H	-0.109430	0.635987	-1.697296
C	-0.785410	0.668089	1.643684
I	-3.013192	-0.120236	-0.297743
H	-0.110026	-1.630384	-0.784358
H	0.091431	-1.785338	0.984417
C	3.153759	-1.221886	0.204858
C	4.528997	-1.266352	0.088578
C	5.218712	-0.147617	-0.362885
C	4.541322	1.017608	-0.704406
C	3.166462	1.068565	-0.593859
H	2.606294	-2.088057	0.553997
H	5.066181	-2.168450	0.347389

H	6.296821	-0.184511	-0.451869
H	5.089391	1.880663	-1.056731
H	2.629602	1.970249	-0.858377
H	-1.154737	0.008830	2.425744
H	-1.197532	1.667877	1.756784
O	0.657760	0.764770	1.694098
H	1.023307	0.221681	2.402927

10. References

[1] R. Giovanelli, G. Gallorini, Y. Huang, A. Brunetti, K. D. Nikopoulos, M. Monari, C. Silva Lòpez, S. Kiriakidi, G. Bertuzzi, M. Bandini, “Dual Au(I) Catalysis in Regioselective Cycloaddition of Bicyclo[1.1.0]butanes with Allenes” *ACS Catal.* 2025, **15**, 15698.

[2] Y. Wang, L. Zheng, X. Liu, C. Zhang, Y. Li, Q. Li, X. Zhang, W. Lin, C. Liu, "Iodine(I)-Induced Skeletal Rearrangement of α-Bicyclobutanyl Alcohols and Amines: A Universal Approach to 2-Oxabicyclo[2.1.1]hexanes and 2-Azabicyclo[2.1.1]hexanes" *Angew. Chem. Int. Ed.*, 2025, **65**, e23632.

[3] J.-D. Chai, M. Head-Gordon, *Phys. Chem. Chem. Phys.*, **2008**, *10*, 6615-20.

[4] F. Weigend, *Phys. Chem. Chem. Phys.*, **2006**, *8*, 1057-1065.

[5] F. Weigend, R. Ahlrichs, *Phys. Chem. Chem. Phys.*, **2005**, *7*, 3297-3305.

[6] J. Tomasi, B. Mennucci, R. Cammi, *Chem. Rev.*, **2005**, *105*, 2999–3093.

[7] Gaussian 16, Revision C.01, M. J. Frisch, G. W. Trucks, H. B. Schlegel, G. E. Scuseria, M. A. Robb, J. R. Cheeseman, G. Scalmani, V. Barone, G. A. Petersson, H. Nakatsuji, X. Li, M. Caricato, A. V. Marenich, J. Bloino, B. G. Janesko, R. Gomperts, B. Mennucci, H. P. Hratchian, J. V. Ortiz, A. F. Izmaylov, J. L. Sonnenberg, D. Williams-Young, F. Ding, F. Lipparini, F. Egidi, J. Goings, B. Peng, A. Petrone, T. Henderson, D. Ranasinghe, V. G. Zakrzewski, J. Gao, N. Rega, G. Zheng, W. Liang, M. Hada, M. Ehara, K. Toyota, R. Fukuda, J. Hasegawa, M. Ishida, T. Nakajima, Y. Honda, O. Kitao, H. Nakai, T. Vreven, K. Throssell, J. A. Montgomery, Jr., J. E. Peralta, F. Ogliaro, M. J. Bearpark, J. J. Heyd, E. N. Brothers, K. N. Kudin, V. N. Staroverov, T. A. Keith, R. Kobayashi, J. Normand, K. Raghavachari, A. P. Rendell, J. C. Burant, S. S. Iyengar, J. Tomasi, M. Cossi, J. M. Millam, M. Klene, C. Adamo, R. Cammi, J. W. Ochterski, R. L. Martin, K. Morokuma, O. Farkas, J. B. Foresman, and D. J. Fox, Gaussian, Inc., Wallingford CT, 2016

8 H. B. Schlegel and J. J. W. McDouall, “Do You Have SCF Stability and Convergence Problems?,” in *Computational Advances in Organic Chemistry: Molecular Structure and Reactivity*, Dordrecht: Springer Netherlands, 1991, 167–185.

[9] R. Bauernschmitt, R. Ahlrichs, *J. Chem. Phys.*, **1996**, *104*, 9047–9052.

[10] R. Seeger, J. A. Pople, *J. Chem. Phys.*, **1977**, 66, 3045–3050.

[11] H.-K. Fan, S. Yang, J.-h. Li, Q.-Q. Teng, M. Chen, “BaO-Catalyzed *α*-Deuteration of Ketones, Sulfones, Sulfoxides, and Nitriles” *Eur. J. Org. Chem.* 2022, e202201218.

[12] Prepared following the procedure reported in ref. 2.

[13] See for example a) S. Yang, W. Denny, “A New Short Synthesis of 3-Substituted 5-Amino-1-(chloromethyl)-1,2-dihydro-3*H*-benzo[*e*]indoles (Amino-CBIs)” *J. Org. Chem.* 2002, **67**, 8958; b) A. J. Smaligo, J. Wu, N. R. Burton, A. S. Hacker, A. C. Shaikh, J. C. Quintana, R. Wang, C. Xie, O. Kwon, “Oxodealkenylative Cleavage of Alkene C(sp^3)–C(sp^2) Bonds: A Practical Method for Introducing Carbonyls into Chiral Pool Materials” *Angew. Chem. Int. Ed.* 2020, **59**, 1211.

[14] a) APEX3 Software Package V2019; Bruker AXS Inc.: Madison, WI, 2019; b) Bruker SAINT, v8.40A: Part of the APEX3 Software Package V2019; Bruker AXS Inc.: Madison, WI, 2019; c) Bruker SADABS V2016/2: Part of the APEX3 Software Package V2019; Bruker AXS Inc.: Madison, WI, 2019.

[15] a) G. M. Sheldrick, “SHELXT − Integrated Space-Group and Crystal-Structure Determination” *Acta Crystallogr., Sect. A: Found. Adv.* 2015, **71**, 3; b) G. M. Sheldrick, “Crystal Structure Refinement with SHELXL” *Acta Crystallogr., Sect. C: Cryst. Struct. Commun.* 2015, **71**, 3.

[16] C. F. Macrae, I. Sovago, S. J. Cottrell, P. T. A. Galek, P. McCabe, E. Pidcock, M. Platings, G. P. Shields, J. S. Stevens, M. Towler P. A. Wood, “*Mercury 4.0*: from visualization to analysis, design and prediction” *J. Appl. Cryst.* 2020, **53**, 226.

[17] As a possible explanation to the higher energy demand for **TS1** and **TS1’** in the TEMPO-pathway with respect to the ones regarding bromide, iodide or thiolate, we suggest the higher stability and greater steric encumbrance of both the radical and the cationic forms of TEMPO compared to halo (and thio) radicals or halonium (and thionium) cations. Moreo
